# Benefits and harms of the human papillomavirus (HPV) vaccines: comparison of trial data from clinical study reports with corresponding trial register entries and journal publications

**DOI:** 10.1186/s13643-020-01300-1

**Published:** 2020-02-28

**Authors:** Lars Jørgensen, Peter C. Gøtzsche, Tom Jefferson

**Affiliations:** Nordic Cochrane Centre, Rigshospitalet 7811, Blegdamsvej 9, 2100 Copenhagen, Denmark

**Keywords:** Human papillomavirus vaccine, Randomized clinical trial, Clinical study report, Trial register entry, Journal publication, Meta-analysis, Systematic review, Risk of bias

## Abstract

**Background:**

No study has looked at differences of pooled estimates—such as meta-analyses—of corresponding study documents of the same intervention. In this study, we compared meta-analyses of human papillomavirus (HPV) vaccine trial data from clinical study reports with trial data from corresponding trial register entries and journal publications.

**Methods:**

We obtained clinical study reports from the European Medicines Agency and GlaxoSmithKline, corresponding trial register entries from ClinicalTrials.gov and corresponding journal publications via the Cochrane Collaboration’s Central Register of Controlled Trials, Google Scholar and PubMed. Two researchers extracted data. We compared reporting of trial design aspects and 20 prespecified benefit and harm outcomes extracted from each study document type. Risk ratios were calculated with the random effects inverse variance method.

**Results:**

We included study documents from 22 randomized clinical trials and 2 follow-up studies with 95,670 healthy participants and non-HPV vaccine comparators (placebo, HPV vaccine adjuvants and hepatitis vaccines). We obtained 24 clinical study reports, 24 corresponding trial register entries and 23 corresponding journal publications; the median number of pages was 1351 (range 357 to 11,456), 32 (range 11 to 167) and 11 (range 7 to 83), respectively. All 24 (100%) clinical study reports, no (0%) trial register entries and 9 (39%) journal publications reported on all six major design-related biases defined by the Cochrane Handbook version 2011. The clinical study reports reported more inclusion criteria (mean 7.0 vs. 5.8 [trial register entries] and 4.0 [journal publications]) and exclusion criteria (mean 17.8 vs. 11.7 and 5.0) but fewer primary outcomes (mean 1.6 vs. 3.5 and 1.2) and secondary outcomes (mean 8.8 vs. 13.0 and 3.2) than the trial register entries. Results were posted for 19 trial register entries (79%). Compared to the clinical study reports, the trial register entries and journal publications contained 3% and 44% of the seven assessed benefit data points (6879 vs. 230 and 3015) and 38% and 31% of the 13 assessed harm data points (167,550 vs. 64,143 and 51,899). No meta-analysis estimate differed significantly when we compared pooled risk ratio estimates of corresponding study document data as ratios of relative risk.

**Conclusion:**

There were no significant differences in the meta-analysis estimates of the assessed outcomes from corresponding study documents. The clinical study reports were the superior study documents in terms of the quantity and the quality of the data they contained and should be used as primary data sources in systematic reviews.

**Systematic review registration:**

The protocol for our comparison is registered on PROSPERO as an addendum to our systematic review of the benefits and harms of the HPV vaccines: https://www.crd.york.ac.uk/PROSPEROFILES/56093_PROTOCOL_20180320.pdf: CRD42017056093. Our systematic review protocol was registered on PROSPERO on January 2017: https://www.crd.york.ac.uk/PROSPEROFILES/56093_PROTOCOL_20170030.pdf. Two protocol amendments were registered on PROSPERO on November 2017: https://www.crd.york.ac.uk/PROSPEROFILES/56093_PROTOCOL_20171116.pdf. Our index of the HPV vaccine studies was published in *Systematic Reviews* on January 2018: 10.1186/s13643-018-0675-z. A description of the challenges obtaining the data was published on September 2018: 10.1136/bmj.k3694.

## Background

Since 1995, the pharmaceutical industry has written structured clinical study reports of randomized clinical trials following international guidelines to document their products’ benefits and harms when applying for marketing approval [1]. Clinical study reports are usually confidential documents, but can be requested or downloaded from the European Medicines Agency (EMA) [2], ClinicalStudyDataRequest.com (CSDR), GlaxoSmithKline’s trial register website and, in the future, possibly from the US Food and Drug Administration (FDA) [3]. Publicly available trial data mainly come from biomedical journal publications and trial register entries such as those on ClinicalTrials.gov. The intention of ClinicalTrials.gov is that all studies publish all results and that those who do not publish results within 12 months of trial completion are fined. According to fdaaa.trialstracker.net, 32% of studies on ClinicalTrials.gov have no results posted and no fines have been issued. Clinical study reports usually have all prespecified data included or include amendments. There can be important differences in results from published [4] and unpublished [5] versions of corresponding study documents. Clinical study reports include highly detailed information on all aspects of a trial [6] and are on average about 2000 pages long [7], but it can be difficult to obtain complete and unredacted clinical study reports [8].

We carried out a systematic review of the human papillomavirus (HPV) vaccines’ clinical study reports [9] based on an index we constructed of 206 HPV vaccine studies [10]. As of July 2017, 62% (92/149) of the completed studies were not published in journal publications and 48% (71/147) of the completed studies on ClinicalTrials.gov had no study results posted [10]. Systematic reviewers often only use journal publications and trial registers for their reviews, which may increase the risk of using a data set influenced by selective outcome reporting.

To our knowledge, no study has looked at differences of pooled estimates—such as meta-analyses—of corresponding study documents of the same intervention. Our primary aim in this study was to compare meta-analyses of HPV vaccine data from clinical study reports with data from corresponding trial register entries and journal publications. Our secondary aim was to compare the reporting of study design aspects of the corresponding study documents.

## Methods

We compared corresponding HPV vaccine study documents of clinical study reports, trial register entries and journal publications to investigate the degree of reporting bias for prespecified outcomes and the reporting of trial design aspects; see our protocol on PROSPERO [11] (registered as ‘Protocol amendment no. 3’ for our systematic review of the HPV vaccines [9]).

Clinical study reports were obtained from EMA and GlaxoSmithKline [9]. We identified the clinical study reports’ corresponding trial register entries on ClinicalTrials.gov and corresponding primary journal publications from our published index of the HPV vaccine studies. The search strings used to identify the studies are available in the index publication [10]. We assessed all identified journal publications for a study (including supplementary documents and errata) for eligible information and chose the primary publication that corresponded to the clinical study report for our comparison. We did not check for eligible information in additional trial registers (such as the EU Clinical Trials Register) or letters to the editors.

Data extraction and comparison of the study documents were carried out by two researchers (LJ extracted the data; TJ checked the extractions; and PCG arbitrated). For each study document, the following data were compared: study ID, number of pages, date of document, time from study completion to publication in a journal, result availability, protocol availability (including pre-specification of outcomes and inclusion of a statistical analysis plan), reporting of PICO criteria (participants, interventions, comparisons and outcomes) and reporting of six major design-related biases defined by the Cochrane Handbook (version 2011) for the Cochrane risk of bias tool [12] (random sequence generation, allocation concealment, blinding of outcome assessors, blinding of personnel, blinding of participants and loss to follow-up). We collected these data, as they are important to evaluate a study’s internal and external validity. We did not include the Cochrane risk of bias tool domain ‘selective outcome reporting’, since we compared this domain quantitatively between corresponding documents.

For each study document, we extracted and compared data on the outcomes we assessed in our systematic review [9]. As our review contained 166 meta-analyses, we only compared the 20 most clinically relevant outcomes (or statistically significant outcomes with a *p* value ≤ 0.05; noted in parentheses). Benefit outcomes included all-cause mortality, HPV-related cancer mortality, HPV-related cancer incidence, HPV-related carcinoma in situ, HPV-related moderate intraepithelial neoplasia, HPV-related moderate intraepithelial neoplasia or worse and HPV-related treatment procedures. Harm outcomes include fatal harms, serious harms (including those judged as ‘definitely associated’ with postural orthostatic tachycardia syndrome [POTS] and complex regional pain syndrome [CRPS; see our systematic review protocol amendment [13] for these two post hoc exploratory analyses] and the nervous system disorders that were Medical Dictionary for Regulatory Activities [MedDRA] classified in this system organ class), new-onset diseases (including back pain, vaginal infection and the vascular disorders that were MedDRA classified in this system organ class) and general harms (including fatigue, headache and myalgia). Histological outcomes were assessed irrespective of involved HPV types. The most aggregated data account (participants with events over the total number of participants) was used for the meta-analyses, and the most detailed harm account of MedDRA preferred terms was used for event comparisons. For example, if harms were registered separately per harm, we would count the separate harms and summarize them as a total number of harms. For all GlaxoSmithKline clinical study reports and for serious harms for Merck clinical study reports, we pooled MedDRA preferred terms in their respective system organ classes. A participant could potentially be included more than once in a separate analysis (e.g. if a participant experienced both serious ‘headache’ and serious ‘dizziness’, the participant would be counted twice in the MedDRA system organ class analysis of serious nervous system disorders); we therefore consider the MedDRA system organ class analyses exploratory.

Merck Sharp & Dohme did not provide a formal definition for its new-onset disease category—new medical history—but described the category as ‘all new reported diagnoses’ in the clinical study report of trial V501-019. Although ‘new medical history’ was not explicitly mentioned in the trial register entries and journal publications, we included eligible new reported diagnoses not reported as serious or general harms in this category.

For our meta-analyses, we used the intention to treat principle. Risk ratios (RRs) were calculated with the random effects inverse variance method. Random effects estimates were compared to fixed effect estimates, as the former method may weigh small trials unduly if there is considerable heterogeneity between trials [12].

## Results

We included study documents from 22 randomized clinical trials and 2 follow-up studies and obtained 24 clinical study reports, 24 corresponding trial register entries and 23 corresponding primary journal publications (for the remaining journal publication—HPV-003, of 61 participants—the manufacturer confirmed that no journal publication had been published [10]). See Additional file 1 for our study’s PRISMA statement.

### Characteristics of included studies

The 24 included studies investigated four different HPV vaccines: Cervarix™, Gardasil™, Gardasil 9™ and an HPV type 16 vaccine, and included 95,670 healthy participants (79,102 females and 16,568 males) aged 8 to 72. One (4%) study used a saline placebo comparator, but its participants had been HPV vaccinated before randomization. Fourteen (58%) studies used vaccine adjuvants as comparators: amorphous aluminium hydroxyphosphate sulphate (AAHS), aluminium hydroxide (Al[OH]_3_) or carrier solution. Nine (38%) studies used hepatitis vaccine comparators: Aimmugen™, Engerix™, Havrix™ or Twinrix Paediatric™.

### Characteristics of included study documents

Nearly all study documents (70/72) reported data from study start to completion, except for the clinical study report and journal publication of study HPV-040 that described interim analyses. The median number of pages in the clinical study reports was 1351 (range 357 to 11,456) (see Table 1). For four studies (HPV-008, HPV-013, HPV-015 and HPV-040), we obtained clinical study reports from both EMA and GlaxoSmithKline (we did not account for duplicate pages). EMA’s clinical study reports were only 22% of the length of the corresponding GlaxoSmithKline reports (5316 vs. 23,645 pages). After transformation to PDFs, the median number of pages in the trial register entries was 32 (range 11 to 167). Results were posted on ClinicalTrials.gov for 19 studies (79%) but were not posted for 5 studies: HPV-001, HPV-003, HPV-013, HPV-033 and HPV-035. The median number of pages in the journal publications—including supplementary appendices—was 11 (range 7 to 83). Twelve (52%) journal publications contained supplementary appendices. The mean time from study completion to journal publication was 2.3 years (see Table 1).

**Table 1:** Comparison of HPV vaccine clinical study reports with trial register entries and journal publications: date and availability of clinical study reports, trial registry report results and journal publications

Manufacturer	Clinical study report			Trial register entries from ClinicalTrials.gov				Journal publication		
	Study ID	*N* = pages^a^	Date of report	NCT ID	*N* = pages	Results posted	Date results posted	Reference	*N* = pages	Date published
**GlaxoSmithKline**	HPV-001	5813	November 13, 2004	NCT00689741	19	No	Not applicable	Harper DM et al [14]	10	November 13, 2004
	HPV-003	799	April 13, 2003	NCT00263744	12	No	Not applicable	Not published	Not applicable	Not applicable
	HPV-008	11,456	July 1, 2009	NCT00122681	132	Yes	January 20, 2010	Paavonen J et al. [15]	25	July 25, 2009
	HPV-013	8323	December 1, 2005	NCT00196924	12	No	September 20, 2005	Medina DM et al. [16]	8	May 1, 2010
	HPV-015	6290	March 31, 2015	NCT00294047	136	Yes	March 27, 2012	Skinner S et al. [17]	20	December 20, 2014
	HPV-023	936	November 12, 2009	NCT00518336	167	Yes	October 25, 2011	Naud PS et al. [18]	19	June 19, 2014
	HPV-029	1543	June 9, 2009	NCT00578227	50	Yes	January 6, 2010	Pedersen C et al. [19]	9	January 1, 2012
	HPV-030	1351	June 17, 2010	NCT00652938	52	Yes	August 31, 2010	Schmeink CE et al. [20]	8	November 15, 2011
	HPV-031	476	December4, 2013	NCT00344032	25	Yes	December 15, 2009	Bhatla N et al. [21]	10	February 4, 2010
	HPV-032	2912	November 1, 2008	NCT00316693	30	Yes	December 16, 2009	Konno R et al. [22]	9	July 4, 2010
	HPV-033	587	March 27, 2007	NCT00290277	11	No	Not applicable	Kim YJ et al. [23]	8	August 1, 2010
	HPV-035	451	June 9, 2008	NCT00306241	14	No	March 23, 2006	Ngan HY et al. [24]	9	June 15, 2010
	HPV-038	957	August 5, 2009	NCT00485732	28	Yes	December 17, 2009	Kim SC et al. [25]	9	June 30, 2011
	HPV-040	2892	April 13, 2016	NCT00534638	45	Yes	January 26, 2016	Lehtinen M et al. [26]	14	March 3, 2015
	HPV-058	1745	May 28, 2012	NCT00996125	22	Yes	June 27, 2012	Zhu F et al. [27]	17	July 1, 2014
	HPV-063	1474	July 19, 2013	NCT00929526	41	Yes	October 15, 2012	Konno R et al. [28]	19	July 1, 2014
	HPV-069	819	June 6, 2013	NCT01277042	32	Yes	December 3, 2013	Zhu F et al. [27]	17	July 1, 2014
**Merck Sharp & Dohme**	V501-005	357	March 8, 2005	NCT00365378	28	Yes	April 9, 2010	Koutsky LA et al. [29]	7	November 21, 2002
	V501-013	1797	November 12, 2007	NCT00092521	48	Yes	November 20, 2009	Garland SM [30]	30	May 10, 2007
	V501-015	713	November 13, 2007	NCT00092534	45	Yes	November 26, 2009	The FUTURE II Study Group [31]	36	May 10, 2007
	V501-018	1014	August 8, 2005	NCT00092547	60	Yes	May 4, 2010	Reisinger KS et al. [32]	11	August 18, 2014
	V501-019	2645	November 17, 2009	NCT00090220	83	Yes	February 1, 2010	Muñoz N et al. [33]	9	June 6, 2009
	V501-020	2595	January 27, 2010	NCT00090285	32	Yes	November 19, 2009	Giuliano AR et al. [34]	76	February 3, 2011
	V503-006	467	June 10, 2011	NCT01047345	33	Yes	December 22, 2014	Garland SM et al. [35]	83	November 27, 2015
**Total pages**		**58,412**			**1157**				**463**	

^a^A page was defined as one A4 PDF page regardless of the number of words or characters per page

### Inclusion of protocols

Ten clinical study reports (42%), no trial register entries (0%) and 2 journal publications (9%) included protocols. All 12 protocols listed prespecified outcomes and contained statistical analysis plans (see Table 2). The GlaxoSmithKline trial register entries contained protocol hyperlinks to ClinicalStudyDataRequest.com, but the protocols were not freely available and had to be requested. We did not request the protocols, as this required us to sign a data sharing agreement, which would restrict our ability to publish our results.

**Table 2:** Comparison of HPV vaccine clinical study reports with trial register entries and journal publications: inclusion of protocol and reporting of trial design aspects including PICO criteria

Inclusion of protocol and reporting of trial design aspects including PICO criteria	Clinical study reports: *N* = 24	Trial register entries: *N* = 24	Publications: *N* = 23
Protocol			
Included in study document	10 (42%)	0 (0%)	2 (9%)
- Prespecified outcomes	10 (100%)	Not applicable	2 (100%)
- Included statistical analysis plan	10 (100%)	Not applicable	2 (100%)
Reporting of six major design-related biases defined by the Cochrane Handbook^a^			
Randomization method was explicitly specified	24 (100%)	0 (0%)	22 (96%)
Allocation concealment was explicitly specified	24 (100%)	0 (0%)	17 (74%)
Blinding of outcome assessors was explicitly specified	24 (100%)	23 (96%)	17 (74%)
Blinding of personnel was explicitly specified	24 (100%)	11 (46%)	12 (52%)
Blinding of participants was explicitly specified	24 (100%)	23 (96%)	12 (52%)
Loss to follow-up (attrition) was explicitly accounted for	24 (100%)	20 (83%)	23 (100%)
Population			
Specified inclusion criteria	24 (100%)	24 (100%)	22 (96%)
- Mean number of inclusion criteria	7.0	5.8	4.0
Specified exclusion criteria	24 (100%)	24 (100%)	20 (87%)
- Mean number of exclusion criteria	17.8	11.7	5.0
Intervention			
Specified HPV vaccine antigens	24 (100%)	18 (75%)	23 (100%)
Specified HPV vaccine adjuvants	24 (100%)	8 (33%)	23 (100%)
Specified dose	24 (100%)	6 (25%)	21 (91%)
Comparator			
Specified content	24 (100%)	8 (33%)	23 (100%)
Specified dose	24 (100%)	6 (25%)	21 (91%)
Reported active comparator as a ‘placebo’^b^	14 (58%)	13 (54%)	17 (74%)
Outcomes			
Primary outcomes explicitly specified	24 (100%)	24 (100%)	18 (78%)
- Mean number of primary outcomes	1.6	3.5	1.2
Secondary outcomes explicitly specified	24 (100%)	24 (100%)	14 (61%)
- Mean number of secondary outcomes	8.8	13.0	3.2

^a^Cochrane Handbook: http://training.cochrane.org/handbook

^b^Active comparators included amorphous aluminium hydroxyphosphate sulphate (AAHS), aluminium hydroxide (Al[OH]_3_), carrier solution and hepatitis vaccines (Aimmugen™, Engerix-B™, Havrix™ and Twinrix Paediatric™)

### Reporting of major design-related biases

All 24 (100%) clinical study reports, no (0%) trial register entries and 9 (39%) journal publications reported explicitly on all six domains to be assessed for bias according to the Cochrane Handbook version 2011 [12] (see Table 2).

### Reporting of PICO criteria

Compared to the trial register entries and journal publications, the clinical study reports reported on average more inclusion criteria (mean 7.0 vs. 5.8 and 4.0, respectively) and exclusion criteria (mean 17.8 vs. 11.7 and 5.0) (see Table 2). As an example, while 20 (83%) clinical study reports reported that participants with immunological disorders were excluded, only 12 (50%) trial register entries and 9 (39%) journal publications reported this criterion. All clinical study reports and journal publications specified the intervention and comparator contents (including antigens, adjuvants and doses), whereas only 18 (75%) and 8 (33%) trial register entries specified these. Active comparators (AAHS, Al[OH]_3_ and carrier solution) were referred to as ‘placebos’ in 14 (58%) clinical study reports, 13 (54%) trial register entries and 17 (74%) journal publications. The mean number of reported primary outcomes was higher in the trial register entries (3.5) than in the clinical study reports (1.6) and the journal publications (1.2). This was also the case for secondary outcomes (13.0 vs. 8.8 and 3.2) (see Table 2).

### Meta-analyses of benefits

Of our seven prespecified benefit outcomes from the clinical study reports, the trial register entries included data for 2 (29%) and the journal publications for 6 (86%) (see Table 3 and Additional file 2). Compared to the clinical study reports, the trial register entries and journal publications contained 3% and 44% of the assessed benefit data points (6879 vs. 230 and 3015). Due to the lack of data in the trial register entries and journal publications, it was only possible to calculate the ratios of relative risk for half (10/21) of the prespecified benefit comparisons (see Table 4). The meta-analysis risk ratio estimates from corresponding study documents did not differ much (see Table 3), and the ratios of relative risk differences that could be calculated was not statistically significant (see Table 4).

**Table 3:** Comparison of HPV vaccine clinical study reports with trial register entries and journal publications: results of benefit and harm meta-analyses of intention to treat analyses irrespective of HPV type

Results of benefits and harms meta-analyses of intention to treat analyses irrespective of HPV type^a^	Clinical study reports			Trial register entries			Journal publications		
	HPV vaccine (*n* = 47,075)	Comparator (*n* = 48,595)	Risk ratio^e^ [95% CI]	HPV vaccine (*n* = 47,075)	Comparator (*n* = 48,595)	Risk ratio^e^ [95% CI]	HPV vaccine (*n* = 47,044^f^)	Comparator (*n* = 48,565^f^)	Risk ratio^e^ [95% CI]
Benefits									
All-cause mortality	45	38	1.19 [0.65, 2.19]	39	31	1.30 [0.73, 2.30]	35	28	1.20 [0.51, 2.80]
HPV-related cancer mortality	2	1	1.44 [0.23, 9.12]	0	0	Not applicable	0	0	Not applicable
HPV-related cancer incidence	7	3	1.68 [0.51, 5.49]	0	0	Not applicable	1	0	3.01 [0.12, 73.85]
HPV-related carcinoma in situ	367	490	0.73 [0.53, 1.00]	0	0	Not applicable	212	247	0.85 [0.61, 1.19]
HPV-related moderate intraepithelial neoplasia	538	763	0.81 [0.59, 1.11]	0	0	Not applicable	251	308	0.82 [0.69, 0.96]
HPV-related moderate intraepithelial neoplasia or worse	952	1239	0.78 [0.66, 0.91]	0	0	Not applicable	665	848	0.77 [0.65, 0.92]
HPV-related treatment procedures	1018	1416	0.71 [0.63, 0.80]	76	84	0.90 [0.66, 1.22]	180	240	0.75 [0.62, 0.91]
Total reported benefit data points	2929	3950	Not applicable	115	115	Not applicable	1344	1671	Not applicable
Harms									
Participants with fatal harms	45	38	1.19 [0.65, 2.19]	39	31	1.30 [0.73, 2.30]	35	28	1.20 [0.51, 2.80]
Total number of fatal harms or MedDRA classified fatal harms	79	51	Not applicable	39	31	Not applicable	35	28	Not applicable
Participants with serious harms	1404	1357	1.01 [0.94, 1.08]	1398	1349	1.01 [0.94, 1.09]	1241	1234	1.01 [0.93, 1.09]
Total number of serious harms or MedDRA classified serious harms	1741	1628	Not applicable	1763	1636	Not applicable	1255	1249	Not applicable
- Judged ‘definitely associated’ with CRPS^b^	95	57	1.54 [1.11, 2.14]	88	55	1.52 [1.08, 2.12]	9	2	1.94 [0.57, 6.57]
- Judged ‘definitely associated’ with POTS^b^	56	26	1.92 [1.21, 3.07]	52	23	2.00 [1.23, 3.25]	6	2	1.79 [0.45, 7.22]
- Nervous system disorders	72	46	1.49 [1.02, 2.16]	69	45	1.47 [1.01, 2.15]	12	7	1.45 [0.53, 3.94]
Participants with new-onset diseases^c^	14,258	14,014	0.99 [0.97, 1.02]	4874	4779	1.02 [0.95, 1.10]	4740	4801	1.00 [0.92, 1.09]
Total number of new-onset diseases or MedDRA classified new-onset diseases	47,474	46,662	Not applicable	9972	8673	Not applicable	4740	4801	Not applicable
- Back pain	397	336	1.15 [1.00, 1.33]	68	63	1.08 [0.77, 1.52]	0	0	Not applicable
- Vaginal infection	369	420	0.87 [0.76, 1.00]	0	0	Not applicable	0	0	Not applicable
- Vascular disorders	234	294	0.80 [0.67, 0.94]	0	0	Not applicable	0	0	Not applicable
Participants with general harms^d^	13,248	12,394	1.07 [1.03, 1.11]	3522	3468	1.07 [1.00, 1.15]	8457	7697	1.05 [1.01, 1.10]
Total number of general harms or MedDRA classified general harms	37,999	31,916	Not applicable	22,236	19,793	Not applicable	21,001	18,790	Not applicable
- Fatigue	4933	4489	1.13 [1.08, 1.18]	4255	3901	1.13 [1.07, 1.19]	2343	2210	1.15 [1.04, 1.26]
- Headache	5561	5246	1.06 [1.02, 1.11]	4934	4587	1.07 [1.03, 1.12]	2443	2372	1.08 [1.01, 1.16]
- Myalgia	3989	3047	1.41 [1.24, 1.60]	3508	2688	1.44 [1.21, 1.71]	1868	1193	1.57 [1.23, 2.01]
Total reported MedDRA classified data points	87,293	80,257	Not applicable	34,010	30,133	Not applicable	27,031	24,868	Not applicable

^a^See Additional file 2 for the meta-analyses. It was not feasible to present this summary table for the 16 subgroups that the 24 included studies comprised (based on age-group, gender, type of HPV vaccine and comparator)

^b^We asked a physician with clinical expertise in complex regional pain syndrome (CRPS) and postural orthostatic tachycardia syndrome (POTS) to assess the reported MedDRA preferred terms as ‘definitely,’ ‘probably,’ ‘probably not’ or ‘definitely not’ associated with the syndromes. We sent an Excel sheet to the physician with all the reported MedDRA terms. The physician was blinded, as the Excel sheet contained no outcome data. When the physician had assessed all the MedDRA terms, we synthesized the data for those MedDRA terms that the physician judged ‘definitely’ associated with POTS or CRPS

^c^New-onset diseases were compiled of the harm categories ‘medically significant conditions’ (for Cervarix) and ‘new medical history’ (for Gardasil, Gardasil 9 and the HPV 16 vaccine). GlaxoSmithKline defined ‘medically significant conditions’ as ‘Adverse events prompting emergency room or physician visits that are not (1) related to common diseases or (2) routine visits for physical examination or vaccination, or SAEs [serious adverse events] that are not related to common diseases. Serious adverse events related to common diseases were reported but are not classified as medically significant conditions for analysis purposes. Common diseases include: upper respiratory infections, sinusitis, pharyngitis, gastroenteritis, urinary tract infections, cervicovaginal yeast infections, menstrual cycle abnormalities and injury’. Merck Sharp & Dohme did not provide a formal definition for ‘new medical history’ but described the category as ‘all new reported diagnoses’ in the clinical study report of study V501-019

^d^General harms was compiled of the harm categories ‘solicited general symptoms’, ‘unsolicited general symptoms’ (for Cervarix) and ‘systemic adverse experiences’ (for Gardasil, Gardasil 9 and the HPV 16 vaccine). GlaxoSmithKline defined ‘solicited’ general adverse events as ‘Adverse events to be recorded as endpoints in the clinical study. The presence/occurrence/intensity of these events is actively solicited from the subject or an observer during a specified post-vaccination follow-up period’. GlaxoSmithKline defined ‘unsolicited’ general adverse event as ‘Any AE [adverse event] reported in addition to those solicited during the clinical study. Also, any “solicited” symptom with onset outside the specified period of follow-up for solicited symptoms was reported as an unsolicited AE’. Merck Sharp & Dohme defined ‘systemic adverse event’ as ‘…any systemic clinical adverse event that developed on the day of vaccination or during the 14 days after vaccination was recorded on the VRC [vaccination report card] along with the date it started and the last date it was present’

^e^Risk ratios were calculated with the random effects inverse variance method

^f^The numbers of participants for ‘HPV vaccine’ and ‘comparator’ in the journal publication column were subtracted by 31 and 30 participants, respectively, as no journal publication existed for trial HPV-003 that included 31 and 30 participants

**Table 4:** Comparison of HPV vaccine clinical study reports with trial register entries and journal publications: ratio of relative risk differences of results of benefits and harms

Ratios of relative risk (RRR) of results of benefits and harms^a^	RRR of clinical study reports vs. trial register entries	RRR of clinical study reports vs. journal publications	RRR of trial register entries vs. journal publications
Benefits			
All-cause mortality	0.95 [0.41, 2.18]	1.03 [0.36, 2.92]	1.08 [0.39, 3.02]
- HPV-related cancer mortality	Not applicable^d^	Not applicable	Not applicable
HPV-related cancer incidence	Not applicable	0.55 [0.02, 17.13]	Not applicable
HPV-related carcinoma in situ	Not applicable	0.85 [0.54, 1.36]	Not applicable
HPV-related moderate intraepithelial neoplasia	Not applicable	0.98 [0.69, 1.41]	Not applicable
HPV-related moderate intraepithelial neoplasia or worse	Not applicable	1.02 [0.80, 1.28]	Not applicable
HPV-related treatment procedures	0.79 [0.57, 1.09]	0.95 [0.76, 1.19]	1.20 [0.84, 1.72]
Harms			
Fatal harms	0.95 [0.41, 2.18]	1.03 [0.36, 2.92]	1.08 [0.39, 3.02]
Serious harms	1.00 [0.90, 1.11]	1.00 [0.90, 1.11]	1.00 [0.93, 1.09]
- Judged ‘definitely associated’ with CRPS^b^	1.01 [0.63, 1.62]	0.79 [0.22, 2.81]	0.78 [0.22, 2.78]
- Judged ‘definitely associated’ with POTS^c^	0.96 [0.49, 1.88]	1.07 [0.25, 4.64]	1.12 [0.26, 4.86]
- Nervous system disorders	1.01 [0.60, 1.73]	1.03 [0.35, 3.00]	1.01 [0.35, 2.96]
New-onset diseases	0.97 [0.90, 1.05]	0.99 [0.91, 1.08]	1.02 [0.92, 1.09]
- Back pain	1.06 [0.73, 1.54]	Not applicable	Not applicable
- Vaginal infection	Not applicable	Not applicable	Not applicable
- Vascular disorders	Not applicable	Not applicable	Not applicable
General harms	1.00 [0.92, 1.08]	1.02 [0.96, 1.08]	1.02 [0.94, 1.11]
- Fatigue	1.00 [0.93, 1.07]	0.98 [0.88, 1.09]	0.98 [0.88, 1.09]
- Headache	0.99 [0.93, 1.06]	0.98 [0.91, 1.06]	0.99 [0.91, 1.07]
- Myalgia	0.98 [0.79, 1.21]	0.90 [0.68, 1.18]	0.92 [0.68, 1.24]

^a^Relative risk ratio differences were calculated as a risk ratio calculated with the random effects inverse variance method vs. a risk ratio calculated with the random effects inverse variance method (see Table 3)

^b^CRPS: complex regional pain syndrome (see Table 3)

^c^POTS: postural orthostatic tachycardia syndrome (see Table 3)

^d^Not applicable: when no data were available for the outcome in one (or both) of the compared study document groups (see Table 3)

### Meta-analyses of harms

Of our 13 prespecified harm outcomes from the clinical study reports, the trial register entries included data for 11 (85%) and the journal publications for 10 (77%) (see Tables 3 and 4 and Additional file 2). Compared to the clinical study reports, the trial register entries and journal publications contained 38% and 31% of the assessed harm data points (167,550 vs. 64,143 and 51,899). It was only possible to calculate the ratios of relative risk for 80% (31/39) of the prespecified harm comparisons (see Table 4). The meta-analysis risk ratio estimates did not differ much (see Table 3), and the ratio of relative risk differences that could be calculated was not statistically significant (see Table 4).

### Random effects vs. fixed effect analyses

We found similar results with the fixed effect model but with narrower confidence intervals, as the between-trial variance is not included in this model.

### Subgroup analyses

When we excluded the studies that had no results posted on their corresponding trial register entries (HPV-001, HPV-003, HPV-013, HPV-033 and HPV-035) from the clinical study report meta-analyses, the results did not differ significantly.

### Study document differences

**Fig. 1:**
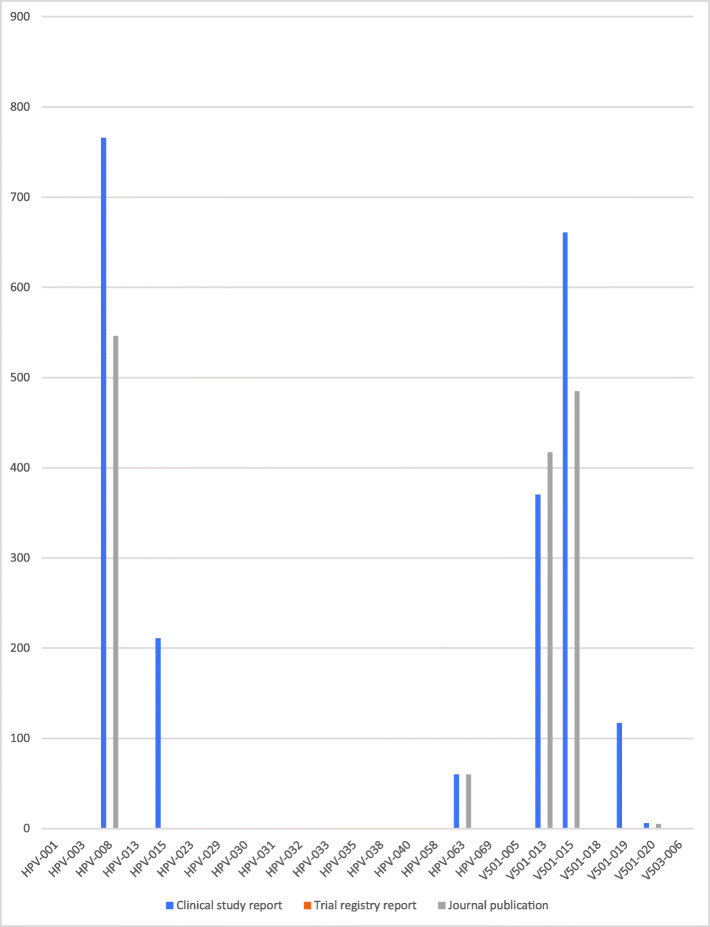
Comparison of HPV vaccine study documents: number of reported cases of HPV-related moderate intraepithelial neoplasia or worse

**Fig. 2:**
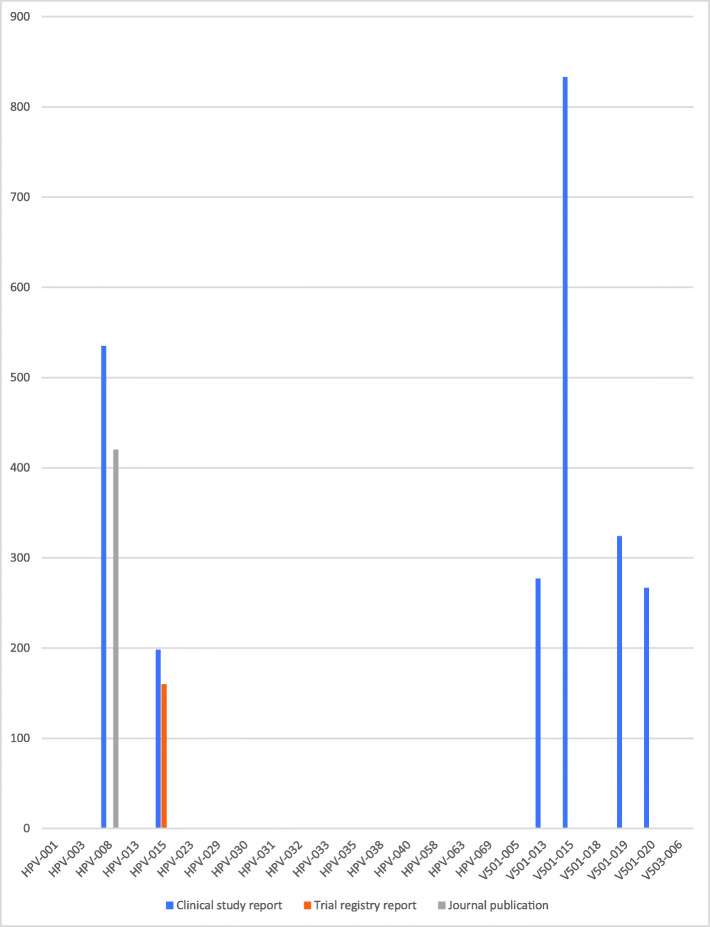
Comparison of HPV vaccine study documents: number of reported cases of HPV-related referral procedures

**Fig. 3:**
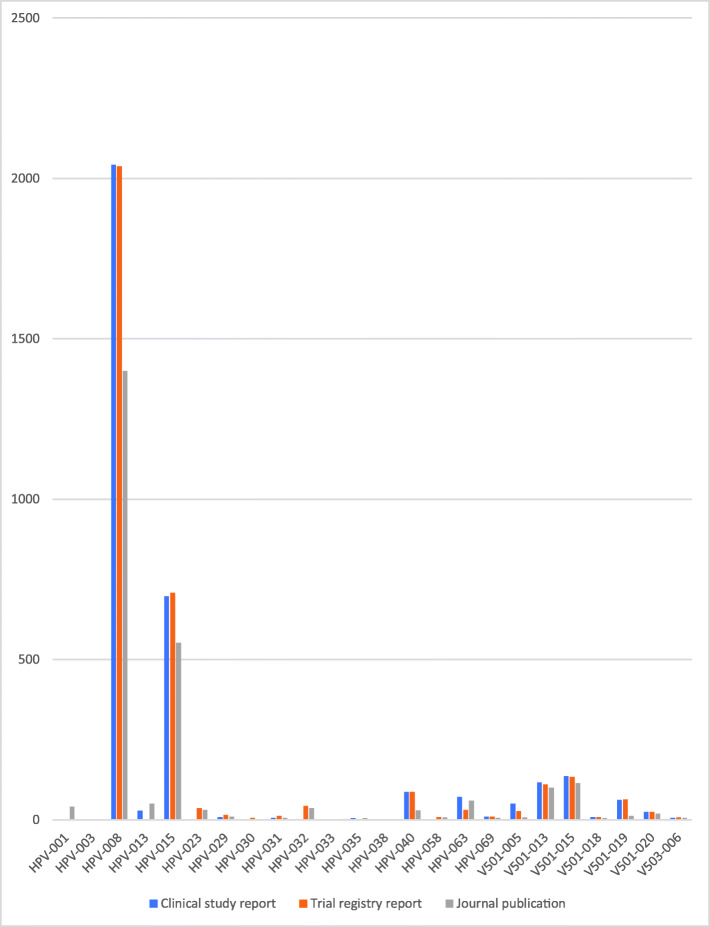
Comparison of HPV vaccine study documents: number of reported serious harms

**Fig. 4:**
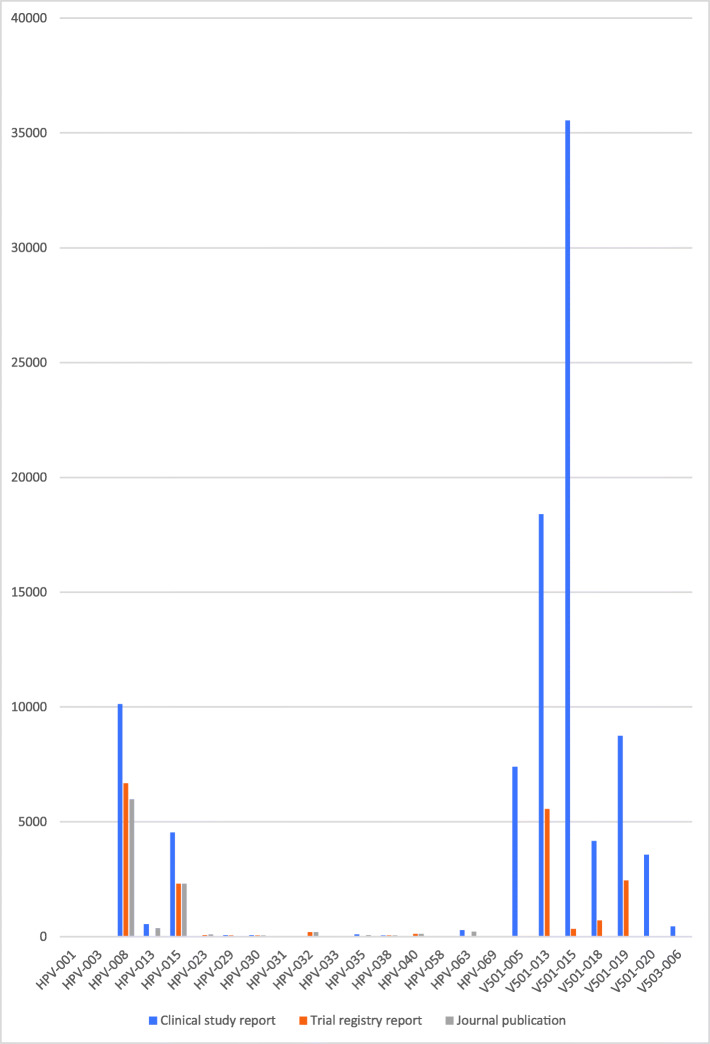
Comparison of HPV vaccine study documents: number of reported new-onset diseases

**Fig. 5:**
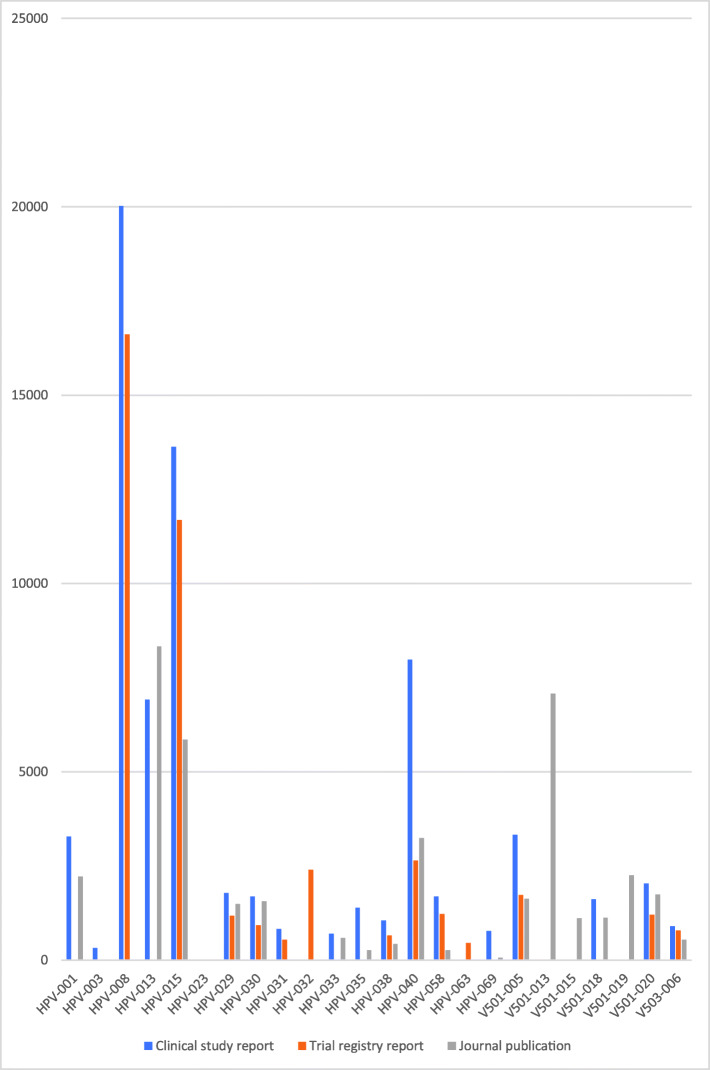
Comparison of HPV vaccine study documents: number of reported general harms

There were substantial differences between the amount of data in the three study document types (see Figs. 1, 2, 3, 4 and 5). For example, the journal publication for V501-013 included more cases of HPV-related moderate intraepithelial neoplasia or worse compared to its clinical study report (417 vs. 370; see Fig. 1). The trial register entry for HPV-015 reported fewer HPV-related treatment procedures than the clinical study report (160 vs. 198; see Fig. 2). The trial registry entry of HPV-040 reported 10 deaths (five in each group), whereas the clinical study report reported ‘no deaths considered as possibly related to vaccination according to the investigator (up to 30 April 2011)’, and the journal publication reported ‘No deaths had been reported at the time of this interim analysis (up to April 2011)’. Compared to the corresponding clinical study report, the journal publication of HPV-008 only contained an aggregate total number of serious harms (1400), whereas the clinical study report contained all individual serious harms classified with MedDRA preferred terms (2043). Only the trial register entries and journal publications for HPV-023 and HPV-032 included serious harms classified with MedDRA preferred terms (see Fig. 3). No journal publication of Merck Sharp & Dohme studies included their new-onset disease category: ‘new medical history’ (V501-005 to V503-006). Merck Sharp & Dohme did not provide a formal definition for ‘new medical history’ but described the category as ‘all new reported diagnoses’ in the clinical study report of trial V501-019. Although not mentioned as an explicit category, the trial register entries reported fewer events of new diagnoses than the clinical study reports (e.g. for V501-015: 329 vs. 35,546; see Fig. 4). Only the trial registry report of HPV-032 and the journal publication of V501-013 included general harms (see Fig. 5).

## Discussion

There were on average 50 and 121 times more pages in the clinical study reports than in their corresponding trial register entries and journal publications. This was likely a main reason why the clinical study reports were superior at reporting trial design aspects. If our systematic review of clinical study reports [9] had relied on trial register entries or journal publications, it would have had no data for a quarter of our prespecified outcomes (11/40). Although the inclusion of clinical study reports led to significantly more eligible and available data, no changes in the direction of available results occurred when comparing the risk ratios of corresponding meta-analyses as ratios of relative risks. This may have several explanations. First, GlaxoSmithKline might be more transparent than other pharmaceutical companies [36], so corresponding study documents from GlaxoSmithKline could be more consistent compared to corresponding study documents from other companies [37–40]. Second, we used the random effects model, but more risk ratios had narrower confidence intervals with a fixed effect model. Third, there were low event numbers for several outcomes; differences in low event numbers may be overestimated when using risk ratios [12]. Finally, the studies were designed with a lack of placebo controls and incomplete reporting of harms [8] and the trial register entries and journal publications only included very few of the assessed data points (from 3% to 44%) compared to the clinical study reports. This may have skewed some of our comparison results towards being false-negative and led to an underestimation of harms caused by the HPV vaccines. Major study design features such as the use of active comparators and the reporting format of harms are not affected by the number of pages in a study document, but the vast increase in the amount of detail in clinical study reports allows for a more complete understanding that might impact conclusions. We have expanded on the issues of the lack of placebo controls and incomplete harms reporting elsewhere [8].

### Strengths and limitations

Our comparison included 71 of 72 primary study documents (except for the journal publication of trial HPV-003 with 61 participants, which does not exist). Nearly all corresponding study documents (70/72) reported data from initiation to completion. To our knowledge, our study is the first with the aim of comparing meta-analyses from different study document data. The majority of study document comparison studies have mainly looked at harms [37–40]; we looked at both benefits and harms.

We did not obtain a single complete and unredacted clinical study report, so the included reports are less useful than complete and unredacted ones. We did not prespecify comparisons of clinical study reports obtained from different sources (i.e. EMA vs. GlaxoSmithKline), and we only prespecified ClinicalTrials.gov register entries for inclusion, as these are intended to have detailed summaries uploaded within 12 months of a study’s completion. We considered it appropriate to only compare a clinical study report with a single corresponding primary register entry and a single corresponding primary journal publication. A comparison that included all published information would become very complex and, in our view, less useful for researchers conducting systematic reviews.

As the clinical study reports were incomplete and often redacted, some eligible data may have been left out. We have described these issues elsewhere [8]. Cervarix™ clinical study reports obtained from EMA were a fifth of the length of the reports that we downloaded from GlaxoSmithKline’s trial register. Merck Sharp & Dohme clinical study reports (of Gardasil™, Gardasil 9™ and the HPV type 16 vaccine) were only obtained from EMA. These consisted of 9588 pages for seven trials. Thus, potentially 40,000 pages remain undisclosed for our comparison of Merck Sharp & Dohme clinical study reports [8].

Only 12 of 71 study documents contained the study protocol. We believe that all study publications should include the study protocol, as readers otherwise are less able to evaluate whether selective outcome reporting, protocol amendments or post hoc analyses were present in the study publication.

It was not possible to compare meta-analyses of per-protocol and intention to treat populations, as we had prespecified [11]. In the trial register entries and journal publications, per-protocol benefit outcomes were not reported irrespective of HPV type and harm results were not reported for per-protocol populations. Differences might have been more marked for these comparisons. For example, in the journal publication for HPV-015, it was stated that ‘Few cases of CIN2^+^ (moderate cervical intraepithelial neoplasia or worse) were recorded’ for the per-protocol population for CIN2^+^ related to HPV types 16 and 18 (25 vs. 34), but the corresponding clinical study report reported four times as many CIN2^+^ cases for the intention to treat population irrespective of HPV type (103 vs. 108).

The lower amount of data points in journal publications might be due to space restrictions, but in many biomedical journals, it is possible to include large electronic appendices. As there is no space restriction on ClinicalTrials.gov [41], the lower amount of data points was likely due to incomplete reporting.

Journal publications for five studies (HPV-031, HPV-035, HPV-040, HPV-058 and HPV-069) only included figures with graphs of general harms without exact numbers. We could calculate the absolute numbers from the percentages of general harms that were provided for four of the five journal publications (HPV-035, HPV-040, HPV-058 and HPV-069).

No journal publication of Merck Sharp & Dohme mentioned ‘new medical history’—a category used in all seven Merck clinical study reports. Merck Sharp & Dohme described ‘new medical history’ as ‘all new reported diagnoses’.

Some data in the trial register entries and journal publications were not comparable for our prespecified outcomes; for example, whereas the clinical study reports had reported an aggregate number of participants experiencing ‘solicited and unsolicited’ harms, the trial register entries and journal publications only reported general harms as ‘solicited’ and ‘unsolicited’ harms and that on a MedDRA preferred term and total level, respectively. We decided to compare such data as number of events but excluded non-aggregated data from the meta-analyses, as the data would constitute a considerable risk of counting participants more than once in an analysis (e.g. for trial register entries for GlaxoSmithKline studies, we only used ‘unsolicited’ events for general harms, as these were reported aggregately). For trial register entries for Merck studies, general harms were reported aggregately with local harms. We had not prespecified local harms as an outcome, so we did not use these data.

Since a journal publication page usually has a higher word and character count than a clinical study report page (that usually has a higher word count than a trial register PDF page), it may have been more appropriate to compare the word count of the study documents instead of the number of pages. As we received clinical study reports both from EMA and GlaxoSmithKline for some clinical study reports, some of the pages were duplicates and the median number of pages was therefore overestimated to some extent.

### Similar studies

Our study supplements earlier studies that found reporting bias from clinical study reports to trial register entries and journal publications [38–40, 42]. Golder et al. performed a systematic review of 11 comparison studies that compared the number of harms in corresponding published and unpublished study documents [37]. They found that 62% (mean) of the harms and 2–100% of the serious harms would have been missed if the comparison studies had relied on journal publications. Similarly, our systematic review of the HPV vaccines of clinical study reports would have missed 62% of the assessed harm data points if it had relied on trial register entries and 69% of the harms if it had relied on journal publications. Our systematic review would have included 1% more serious harms classified with MedDRA preferred terms if it had relied on trial registers but missed 26% serious harms classified with MedDRA preferred terms if it was based on journal publications. It would also have missed 97% of the benefit data points if it had relied on trial register entries and 56% if it had relied on journal publications.

We found a mean time from trial completion to journal publication of 2.3 years. This is similar to a study by Sreekrishnan et al.—from 2018, of 2000 neurology studies—that found a mean time to publication of 2.2 years [43], but less similar to a study by Ross et al.—from 2013, of 1336 clinical trials—that found a mean time to publication of 1.8 years [44].

## Conclusion

There were no significant differences in the meta-analysis estimates of the assessed outcomes from corresponding study documents. The clinical study reports were the superior study documents in terms of the quantity and the quality of the data they contained and should be used as primary data sources in systematic reviews; trial register entries and journal publications should be used concomitantly with clinical study reports, as some data may only be available in trial register entries or journal publications. A systematic review of the HPV vaccines would have had considerably less information and data included if it relied on trial register entries and journal publications instead of clinical study reports. A full data set would be expected to be available from case report forms and individual participant data, but there are regulatory barriers that need to be lifted before independent researchers can access such data [8]. Corresponding study documents ought to use consistent terminology and provide all aggregate and individual benefits and harms data. To test our results’ generalizability, we recommend that other researchers replicate and expand on our method of comparison for other interventions.

## Supplementary information

**Additional file 1.** Word document: Comparison of HPV vaccine study documents: PRISMA 2009 checklist.

**Additional file 2.** PDF document: Comparison of HPV vaccine study documents: meta-analyses.

## Data Availability

The datasets generated and analysed during our study are available from the corresponding author (LJ) upon request.

## Abbreviations

AAHS: Amorphous aluminium hydroxyphosphate sulphate
Al(OH)_3_: Aluminium hydroxide
CRPS: Chronic regional pain syndrome
EMA: European Medicines Agency
FDA: Food and Drug Administration
GSK: GlaxoSmithKline
HPV: Human papillomavirus
ICH: International Council for Harmonisation of Technical Requirements for Pharmaceuticals for Human Use
MedDRA: Medical Dictionary for Regulatory Activities
Merck: Merck & Co., Inc. or Merck Sharp & Dohme outside the USA and Canada
PICO: Patients, interventions, comparisons and outcomes
POTS: Postural orthostatic tachycardia syndrome
PRISMA: Preferred Reporting Items for Systematic Reviews and Meta-Analyses
PROSPERO: International Prospective Register of Systematic Reviews
