# Supplementary material for: Benefits and harms of the human papillomavirus (HPV) vaccines: comparison of trial data from clinical study reports with corresponding trial register entries and journal publications
Source: Syst Rev. 2020 Feb 28;9:42. doi: 10.1186/s13643-020-01300-1 (PMC7047365; doi:10.1186/s13643-020-01300-1)
Supplement: Supplementary file 2 — Additional file 2. PDF document: Comparison of HPV vaccine study documents: meta-analyses. [file 13643_2020_1300_MOESM2_ESM.pdf]

Additional file 2

Comparison of HPV vaccine study documents: meta-analyses

Table of contents

1. Clinical study reports 2

2. Trial register entries 22

3. Journal publications 42

1. Clinical study reports

1.1. All-cause mortality/deaths*: intention to treat analysis

Study or Subgroup	HPV vaccine Events	HPV vaccine Total	Control Events	Control Total	Weight	Risk Ratio IV, Random, 95% CI
**1.1.1 All-cause mortality/deaths: Cervarix vs. Al(OH)3 (females, age 24-72; follow-up until 36 months)**						
HPV-015: Cervarix vs. Al(OH)3 (f, 24-72; 36)	13	2882	5	2871	18.7%	2.59 [0.92, 7.26]
**Subtotal (95% CI)**		**2882**		**2871**	**18.7%**	**2.59 [0.92, 7.26]**
Total events	13		5			
Heterogeneity: Not applicable						
Test for overall effect: Z = 1.81 (P = 0.07)						
**1.1.2 All-cause mortality/deaths: Cervarix vs. Aimmugen: hepatitis A vaccine (females, age 19-25; follow-up until 7 months)**						
HPV-032: Cervarix vs. Aimmugen (f, 19-25; 7)	1	519	0	521	3.3%	3.01 [0.12, 73.76]
**Subtotal (95% CI)**		**519**		**521**	**3.3%**	**3.01 [0.12, 73.76]**
Total events	1		0			
Heterogeneity: Not applicable						
Test for overall effect: Z = 0.68 (P = 0.50)						
**1.1.3 All-cause mortality/deaths: Cervarix vs. Engerix: hepatitis B vaccine (females, age 26-46; follow-up until 12 months)**						
HPV-069: Cervarix vs. Engerix (f, 26-46; 12)	1	606	0	606	3.3%	3.00 [0.12, 73.50]
**Subtotal (95% CI)**		**606**		**606**	**3.3%**	**3.00 [0.12, 73.50]**
Total events	1		0			
Heterogeneity: Not applicable						
Test for overall effect: Z = 0.67 (P = 0.50)						
**1.1.4 All-cause mortality/deaths: Cervarix vs. Havrix: hepatitis A vaccine (females, age 15-25; follow-up until 48 months)**						
HPV-008: Cervarix vs. Havrix (f, 15-25; 48)	10	9319	13	9325	23.2%	0.77 [0.34, 1.75]
**Subtotal (95% CI)**		**9319**		**9325**	**23.2%**	**0.77 [0.34, 1.75]**
Total events	10		13			
Heterogeneity: Not applicable						
Test for overall effect: Z = 0.62 (P = 0.53)						
**1.1.5 All-cause mortality/deaths: Gardasil vs. AAHS (females, age 15-26; follow-up until 45 months)**						
V501-013: Gardasil vs. AAHS (f, 16-24; 45)	2	2723	3	2732	9.0%	0.67 [0.11, 4.00]
V501-015: Gardasil vs. AAHS (f, 15-26; 36)	7	6087	5	6080	16.6%	1.40 [0.44, 4.40]
**Subtotal (95% CI)**		**8810**		**8812**	**25.6%**	**1.13 [0.43, 2.96]**
Total events	9		8			
Heterogeneity: Tau² = 0.00; Chi² = 0.46, df = 1 (P = 0.50); I² = 0%						
Test for overall effect: Z = 0.24 (P = 0.81)						
**1.1.6 All-cause mortality/deaths: Gardasil vs. AAHS (females, age 21-46; follow-up until 48 months)**						
V501-019: Gardasil vs. AAHS (f, 21-46; 48)	7	1911	1	1908	7.0%	6.99 [0.86, 56.75]
**Subtotal (95% CI)**		**1911**		**1908**	**7.0%**	**6.99 [0.86, 56.75]**
Total events	7		1			
Heterogeneity: Not applicable						
Test for overall effect: Z = 1.82 (P = 0.07)						
**1.1.7 All-cause mortality/deaths: Gardasil vs. AAHS (males, age 15-27; follow-up until 36 months)**						
V501-020: Gardasil vs. AAHS (m, 15-27; 36)	3	2032	10	2033	14.4%	0.30 [0.08, 1.09]
**Subtotal (95% CI)**		**2032**		**2033**	**14.4%**	**0.30 [0.08, 1.09]**
Total events	3		10			
Heterogeneity: Not applicable						
Test for overall effect: Z = 1.83 (P = 0.07)						
**1.1.8 All-cause mortality/deaths: HPV 16 vaccine vs. AAHS (females, age 16-25; follow-up until 48 months)**						
V501-005: HPV 16 vaccine vs. AAHS (f, 16-25; 48)	1	1204	1	1205	4.3%	1.00 [0.06, 15.98]
**Subtotal (95% CI)**		**1204**		**1205**	**4.3%**	**1.00 [0.06, 15.98]**
Total events	1		1			
Heterogeneity: Not applicable						
Test for overall effect: Z = 0.00 (P = 1.00)						
**Total (95% CI)**		**27283**		**27281**	**100.0%**	**1.19 [0.65, 2.19]**
Total events	45		38			
Heterogeneity: Tau² = 0.24; Chi² = 11.49, df = 8 (P = 0.18); I² = 30%						
Test for overall effect: Z = 0.56 (P = 0.58)						
Test for subgroup differences: Chi² = 11.02, df = 7 (P = 0.14), I² = 36.5%						

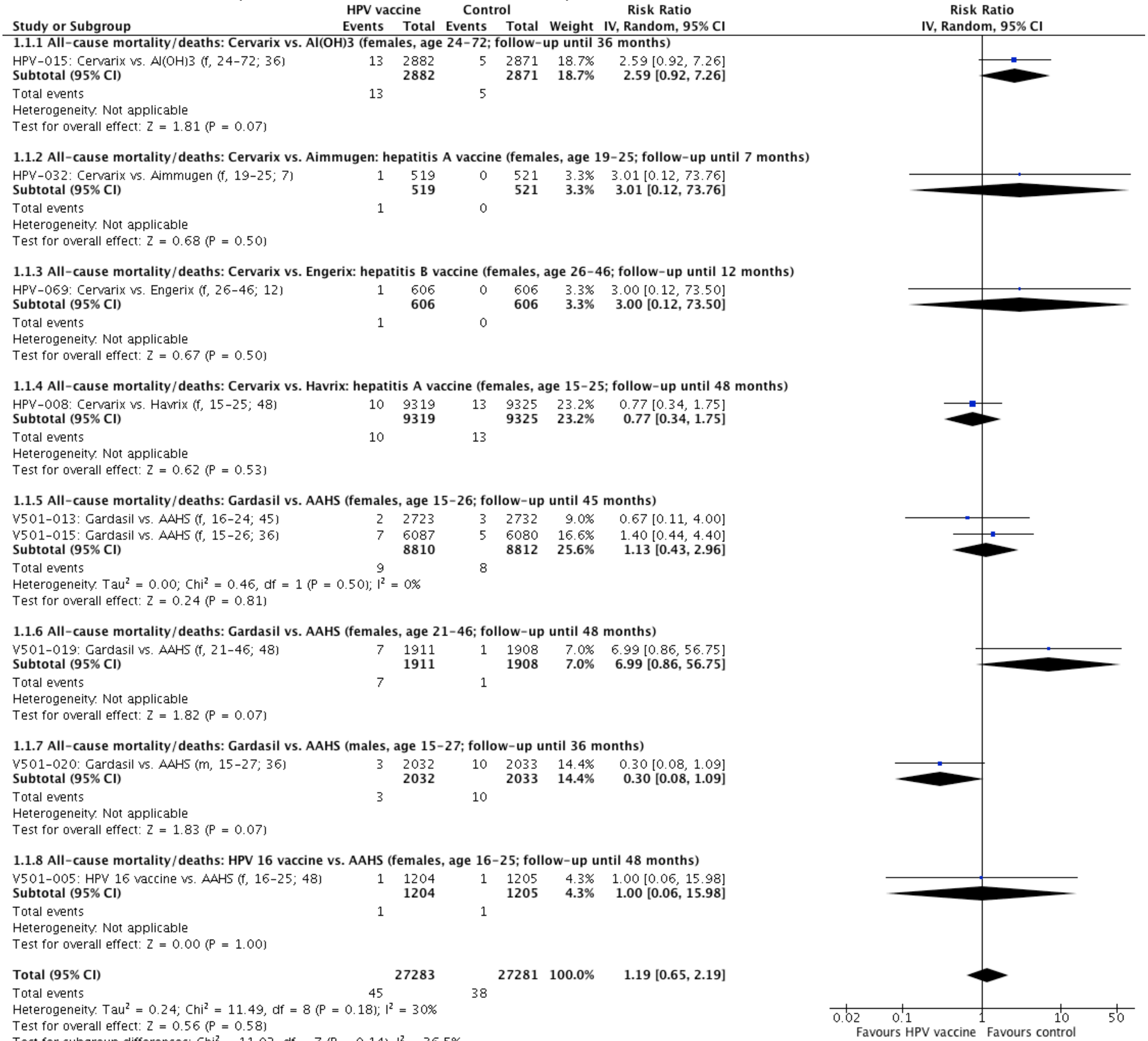

*1.1. Risk ratio for GlaxoSmithKline studies (i.e., HPV-0xx): 1.43 [0.65, 3.15]; risk ratio for Merck Sharp & Dohme studies (i.e., V50x-xxx): 1.08 [0.40, 2.96].

1.2. Mortality/deaths from HPV-related cancers (anal, cervical, oropharyngeal, penile, vaginal and vulvar) irrespective of HPV type*: intention to treat analysis

	HPV vaccine		Control			Risk Ratio
**Study or Subgroup**	**Events**	**Total**	**Events**	**Total**	**Weight**	**IV, Random, 95% CI**
**1.2.1 Cervical cancer: Cervarix (females, age 24-72; follow-up until 36 months)**						
HPV-015: Cervarix vs. Al(OH)3 (f, 24-72; 36)	1	2882	0	2871	33.3%	2.99 [0.12, 73.33]
**Subtotal (95% CI)**		**2882**		**2871**	**33.3%**	**2.99 [0.12, 73.33]**
Total events	1		0			
Heterogeneity: Not applicable						
Test for overall effect: Z = 0.67 (P = 0.50)						
**1.2.2 Oropharyngeal cancer: Cervarix (females, age 24-72; follow-up until 36 months)**						
HPV-015: Cervarix vs. Al(OH)3 (f, 24-72; 36)	0	2882	1	2871	33.3%	0.33 [0.01, 8.15]
**Subtotal (95% CI)**		**2882**		**2871**	**33.3%**	**0.33 [0.01, 8.15]**
Total events	0		1			
Heterogeneity: Not applicable						
Test for overall effect: Z = 0.68 (P = 0.50)						
**1.2.3 Oropharyngeal cancer: Gardasil (females, age 21-46; follow-up until 48 months)**						
V501-019: Gardasil vs. AAHS (f, 21-46; 48)	1	1911	0	1908	33.3%	3.00 [0.12, 73.48]
**Subtotal (95% CI)**		**1911**		**1908**	**33.3%**	**3.00 [0.12, 73.48]**
Total events	1		0			
Heterogeneity: Not applicable						
Test for overall effect: Z = 0.67 (P = 0.50)						
**Total (95% CI)**		**7675**		**7650**	**100.0%**	**1.44 [0.23, 9.12]**
Total events	2		1			
Heterogeneity: Tau² = 0.00; Chi² = 1.21, df = 2 (P = 0.55); I² = 0%						
Test for overall effect: Z = 0.39 (P = 0.70)						
Test for subgroup differences: Chi² = 1.21, df = 2 (P = 0.55), I² = 0%						

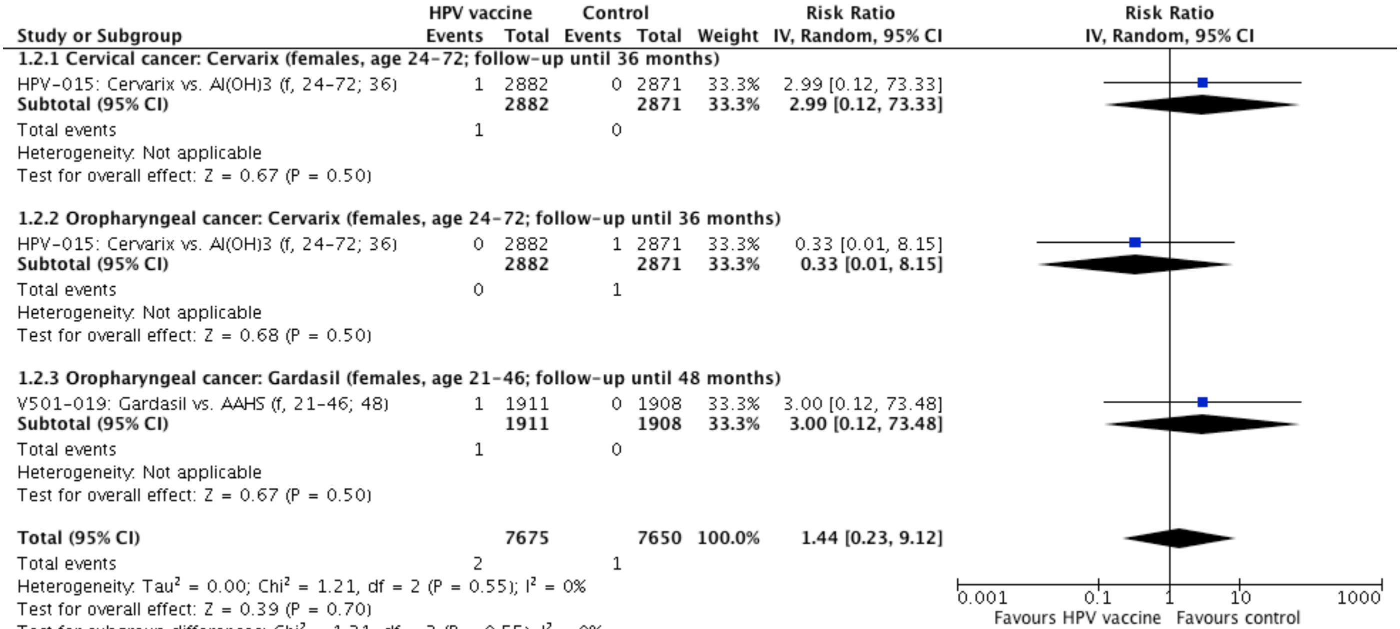

*1.2. Risk ratio for GlaxoSmithKline studies (i.e., HPV-0xx): 1.00 [0.10, 9.57]; risk ratio for Merck Sharp & Dohme studies (i.e., V50x-xxx): 3.00 [0.12, 73.48].

1.3. Incidence of HPV-related cancers (anal, cervical, oropharyngeal, penile, vaginal and vulvar) irrespective of HPV type*: intention to treat analysis

Study or Subgroup	HPV vaccine Events	HPV vaccine Total	Control Events	Control Total	Weight	Risk Ratio IV, Random, 95% CI
**1.3.1 Cervical cancer: Cervarix (females, age 24-72; follow-up until 36 months)**						
HPV-015: Cervarix vs. Al(OH)3 (f, 24-72; 36)	3	2882	0	2871	16.0%	6.97 [0.36, 134.94]
**Subtotal (95% CI)**		**2882**		**2871**	**16.0%**	**6.97 [0.36, 134.94]**
Total events	3		0			
Heterogeneity: Not applicable						
Test for overall effect: Z = 1.28 (P = 0.20)						
**1.3.2 Cervical cancer: Gardasil (females, age 21-46; follow-up until 48 months)**						
V501-019: Gardasil vs. AAHS (f, 21-46; 48)	0	1911	2	1908	15.3%	0.20 [0.01, 4.16]
**Subtotal (95% CI)**		**1911**		**1908**	**15.3%**	**0.20 [0.01, 4.16]**
Total events	0		2			
Heterogeneity: Not applicable						
Test for overall effect: Z = 1.04 (P = 0.30)						
**1.3.3 Oropharyngeal cancer: Cervarix (females, age 24-72; follow-up until 36 months)**						
HPV-015: Cervarix vs. Al(OH)3 (f, 24-72; 36)	0	2882	1	2871	13.7%	0.33 [0.01, 8.15]
**Subtotal (95% CI)**		**2882**		**2871**	**13.7%**	**0.33 [0.01, 8.15]**
Total events	0		1			
Heterogeneity: Not applicable						
Test for overall effect: Z = 0.68 (P = 0.50)						
**1.3.4 Oropharyngeal cancer: Gardasil (females, age 21-46; follow-up until 48 months)**						
V501-019: Gardasil vs. AAHS (f, 21-46; 48)	1	1911	0	1908	13.7%	3.00 [0.12, 73.48]
**Subtotal (95% CI)**		**1911**		**1908**	**13.7%**	**3.00 [0.12, 73.48]**
Total events	1		0			
Heterogeneity: Not applicable						
Test for overall effect: Z = 0.67 (P = 0.50)						
**1.3.5 Vaginal cancer: Cervarix (females, age 24-72; follow-up until 36 months)**						
HPV-015: Cervarix vs. Al(OH)3 (f, 24-72; 36)	1	2882	0	2871	13.7%	2.99 [0.12, 73.33]
**Subtotal (95% CI)**		**2882**		**2871**	**13.7%**	**2.99 [0.12, 73.33]**
Total events	1		0			
Heterogeneity: Not applicable						
Test for overall effect: Z = 0.67 (P = 0.50)						
**1.3.6 Vulvar cancer: Cervarix (females, age 15-25; follow-up until 48 months)**						
HPV-008: Cervarix vs. Havrix (f, 15-25; 48)	1	9319	0	9325	13.7%	3.00 [0.12, 73.68]
**Subtotal (95% CI)**		**9319**		**9325**	**13.7%**	**3.00 [0.12, 73.68]**
Total events	1		0			
Heterogeneity: Not applicable						
Test for overall effect: Z = 0.67 (P = 0.50)						
**1.3.7 Vulvar cancer: Gardasil (females, age 15-26; follow-up until 45 months)**						
V501-013: Gardasil vs. AAHS (f, 16-24; 45)	1	2723	0	2732	13.7%	3.01 [0.12, 73.85]
**Subtotal (95% CI)**		**2723**		**2732**	**13.7%**	**3.01 [0.12, 73.85]**
Total events	1		0			
Heterogeneity: Not applicable						
Test for overall effect: Z = 0.67 (P = 0.50)						
**Total (95% CI)**		**24510**		**24486**	**100.0%**	**1.68 [0.51, 5.49]**
Total events	7		3			
Heterogeneity: $Tau^2 = 0.00$; $Chi^2 = 4.27$, df = 6 (P = 0.64); $I^2 = 0\%$						
Test for overall effect: Z = 0.85 (P = 0.39)						
Test for subgroup differences: $Chi^2 = 4.27$, df = 6 (P = 0.64), $I^2 = 0\%$						

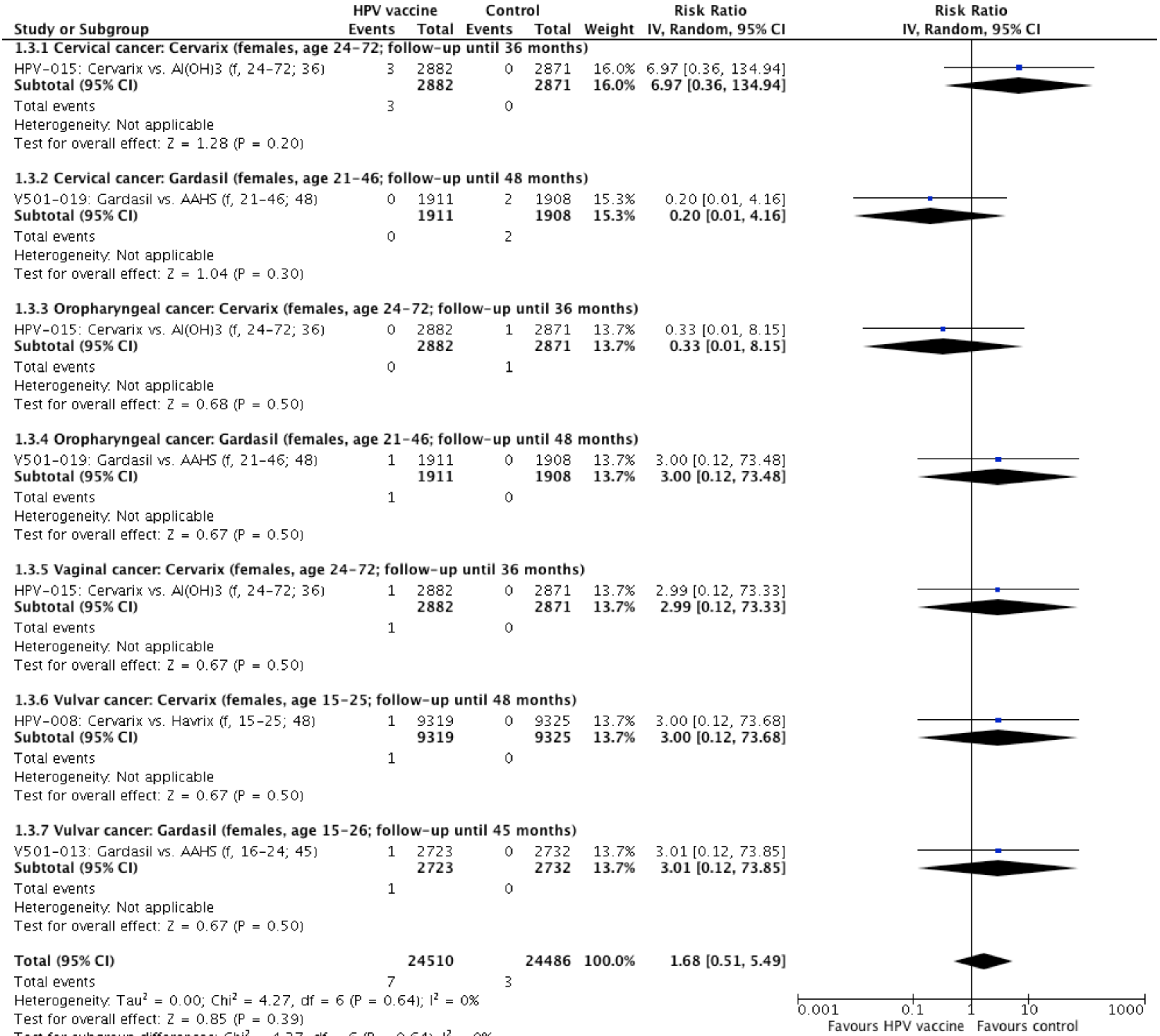

*1.3. Risk ratio for GlaxoSmithKline studies (i.e., HPV-0xx): 2.24 [0.47, 10.74]; risk ratio for Merck Sharp & Dohme studies (i.e., V50x-xxx): 1.14 [0.19, 6.99]. There were no reported cases of anal or penile cancer.

1.4. Incidence of HPV-related carcinoma in situ (anal intraepithelial neoplasia grade 3 [AIN3], cervical adenocarcinoma in situ [AIS], cervical intraepithelial neoplasia grade 3 [CIN3], penile intraepithelial neoplasia grade 3 [PIN3], vaginal intraepithelial neoplasia grade 3 [VIN3] and vulvar intraepithelial neoplasia grade 3 [VaIN3]) irrespective of HPV type*: intention to treat analysis

Study or Subgroup	HPV vaccine Events	HPV vaccine Total	Control Events	Control Total	Weight	Risk Ratio IV, Random, 95% CI
**1.4.1 AIS: Cervarix (females, age 15-25; follow-up until 48 months)**						
HPV-008: Cervarix vs. Havrix (f, 15-25; 48)	3	9319	13	9325	5.2%	0.23 [0.07, 0.81]
**Subtotal (95% CI)**		**9319**		**9325**	**5.2%**	**0.23 [0.07, 0.81]**
Total events	3		13			
Heterogeneity: Not applicable						
Test for overall effect: Z = 2.29 (P = 0.02)						
**1.4.2 AIS: Gardasil (females, age 15-26; follow-up until 45 months)**						
V501-013: Gardasil vs. AAHS (f, 16-24; 45)	1	2723	6	2732	2.1%	0.17 [0.02, 1.39]
V501-015: Gardasil vs. AAHS (f, 15-26; 36)	5	6087	10	6080	6.6%	0.50 [0.17, 1.46]
**Subtotal (95% CI)**		**8810**		**8812**	**8.7%**	**0.40 [0.15, 1.04]**
Total events	6		16			
Heterogeneity: $Tau^2 = 0.00$; $Chi^2 = 0.82$, df = 1 (P = 0.37); $I^2 = 0\%$						
Test for overall effect: Z = 1.88 (P = 0.06)						
**1.4.3 AIS: Gardasil (females, age 21-46; follow-up until 48 months)**						
V501-019: Gardasil vs. AAHS (f, 21-46; 48)	0	1911	2	1908	1.1%	0.20 [0.01, 4.16]
**Subtotal (95% CI)**		**1911**		**1908**	**1.1%**	**0.20 [0.01, 4.16]**
Total events	0		2			
Heterogeneity: Not applicable						
Test for overall effect: Z = 1.04 (P = 0.30)						
**1.4.4 CIN3: Cervarix (females, age 15-25; follow-up until 48 months)**						
HPV-008: Cervarix vs. Havrix (f, 15-25; 48)	83	9319	145	9325	22.6%	0.57 [0.44, 0.75]
**Subtotal (95% CI)**		**9319**		**9325**	**22.6%**	**0.57 [0.44, 0.75]**
Total events	83		145			
Heterogeneity: Not applicable						
Test for overall effect: Z = 4.07 (P < 0.0001)						
**1.4.5 CIN3: Gardasil (females, age 15-26; follow-up until 45 months)**						
V501-013: Gardasil vs. AAHS (f, 16-24; 45)	84	2723	76	2732	21.5%	1.11 [0.82, 1.51]
V501-015: Gardasil vs. AAHS (f, 15-26; 36)	153	6087	208	6080	24.1%	0.73 [0.60, 0.90]
**Subtotal (95% CI)**		**8810**		**8812**	**45.7%**	**0.89 [0.59, 1.33]**
Total events	237		284			
Heterogeneity: $Tau^2 = 0.07$; $Chi^2 = 4.79$, df = 1 (P = 0.03); $I^2 = 79\%$						
Test for overall effect: Z = 0.58 (P = 0.56)						
**1.4.6 CIN3: Gardasil (females, age 21-46; follow-up until 48 months)**						
V501-019: Gardasil vs. AAHS (f, 21-46; 48)	38	1911	30	1908	16.8%	1.26 [0.79, 2.03]
**Subtotal (95% CI)**		**1911**		**1908**	**16.8%**	**1.26 [0.79, 2.03]**
Total events	38		30			
Heterogeneity: Not applicable						
Test for overall effect: Z = 0.97 (P = 0.33)						
**Total (95% CI)**		**40080**		**40090**	**100.0%**	**0.73 [0.53, 1.00]**
Total events	367		490			
Heterogeneity: $Tau^2 = 0.10$; $Chi^2 = 21.43$, df = 7 (P = 0.003); $I^2 = 67\%$						
Test for overall effect: Z = 1.94 (P = 0.05)						
Test for subgroup differences: $Chi^2 = 14.44$, df = 5 (P = 0.01), $I^2 = 65.4\%$						

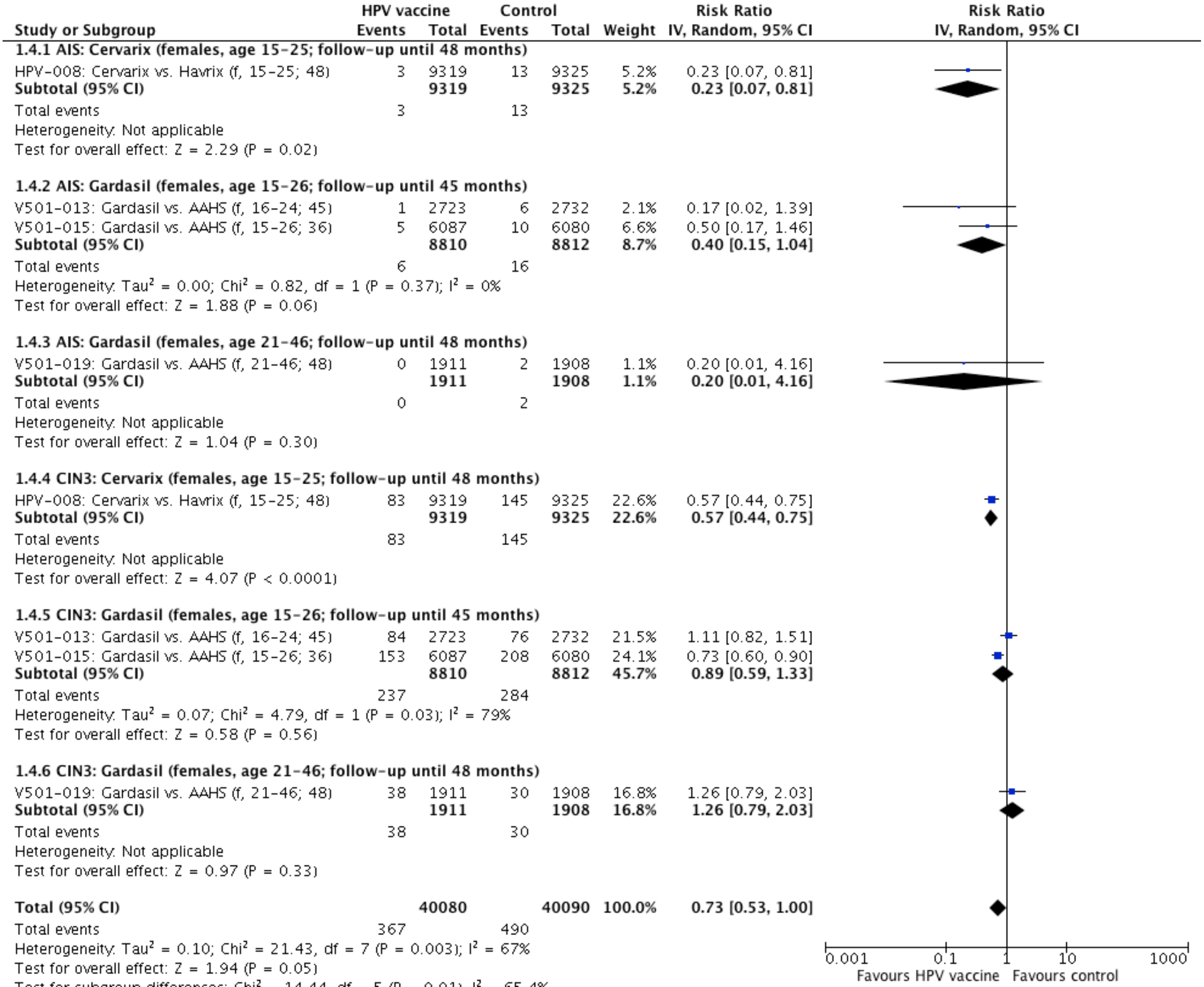

*1.4. Risk ratio for GlaxoSmithKline studies (i.e., HPV-0xx): **0.45 [0.21, 0.99]**; risk ratio for Merck Sharp & Dohme studies (i.e., V50x-xxx): 0.87 [0.62, 1.22]. There were no reports of AIN3, PIN3, VIN3 or VaIN3 irrespective of HPV type.

1.5. Incidence of HPV-related moderate intraepithelial neoplasia (anal intraepithelial neoplasia grade 2 [AIN2], cervical intraepithelial neoplasia grade 2 [CIN2], penile intraepithelial neoplasia grade 2 [PIN2], vaginal intraepithelial neoplasia grade 2 [VIN2] and vulvar intraepithelial neoplasia grade 2 [VaIN2]) irrespective of HPV type*: intention to treat analysis

	HPV vaccine		Control			Risk Ratio
**Study or Subgroup**	**Events**	**Total**	**Events**	**Total**	**Weight**	**IV, Random, 95% CI**
**1.5.1 CIN2: Cervarix (females, age 15-25; follow-up until 48 months)**						
HPV-008: Cervarix vs. Havrix (f, 15-25; 48)	201	9319	370	9325	28.2%	0.54 [0.46, 0.64]
**Subtotal (95% CI)**		**9319**		**9325**	**28.2%**	**0.54 [0.46, 0.64]**
Total events	201		370			
Heterogeneity: Not applicable						
Test for overall effect: Z = 7.06 (P < 0.00001)						
**1.5.2 CIN2: Gardasil (females, age 15-26; follow-up until 45 months)**						
V501-013: Gardasil vs. AAHS (f, 16-24; 45)	110	2723	125	2732	25.7%	0.88 [0.69, 1.13]
V501-015: Gardasil vs. AAHS (f, 15-26; 36)	185	6087	238	6080	27.6%	0.78 [0.64, 0.94]
**Subtotal (95% CI)**		**8810**		**8812**	**53.3%**	**0.81 [0.70, 0.95]**
Total events	295		363			
Heterogeneity: $Tau^2 = 0.00$; $Chi^2 = 0.64$, df = 1 (P = 0.42); $I^2 = 0\%$						
Test for overall effect: Z = 2.68 (P = 0.007)						
**1.5.3 CIN2: Gardasil (females, age 21-46; follow-up until 48 months)**						
V501-019: Gardasil vs. AAHS (f, 21-46; 48)	42	1911	30	1908	18.5%	1.40 [0.88, 2.22]
**Subtotal (95% CI)**		**1911**		**1908**	**18.5%**	**1.40 [0.88, 2.22]**
Total events	42		30			
Heterogeneity: Not applicable						
Test for overall effect: Z = 1.41 (P = 0.16)						
**Total (95% CI)**		**20040**		**20045**	**100.0%**	**0.81 [0.59, 1.11]**
Total events	538		763			
Heterogeneity: $Tau^2 = 0.09$; $Chi^2 = 21.50$, df = 3 (P < 0.0001); $I^2 = 86\%$						
Test for overall effect: Z = 1.31 (P = 0.19)						
Test for subgroup differences: $Chi^2 = 20.86$, df = 2 (P < 0.0001), $I^2 = 90.4\%$						

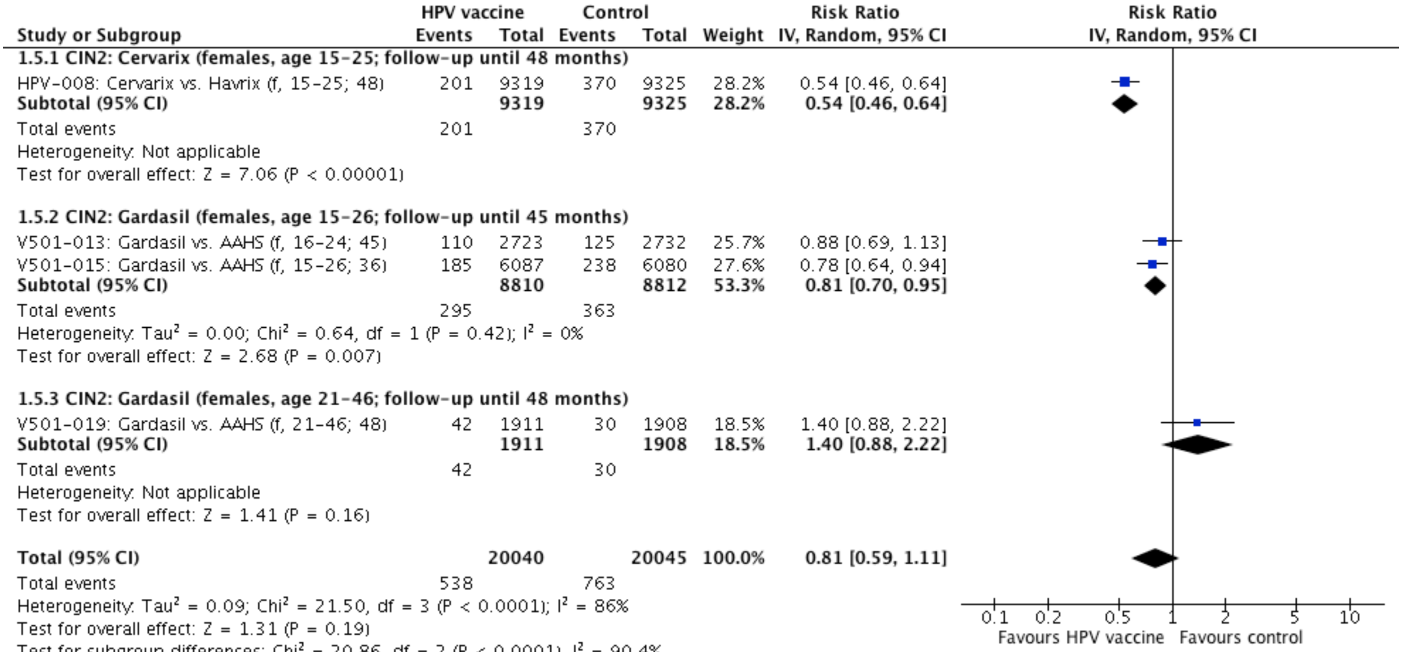

*1.5. Risk ratio for GlaxoSmithKline studies (i.e., HPV-0xx): **0.54 [0.46, 0.64]**; risk ratio for Merck Sharp & Dohme studies (i.e., V50x-xxx): 0.92 [0.70, 1.19]. There were no reports of AIN2, PIN2, VIN2 or VaIN2 irrespective of HPV type.

1.6. Incidence of HPV-related moderate intraepithelial neoplasia or worse (AIN2⁺, CIN2⁺, PIN2⁺, VIN2⁺, VaIN2⁺) irrespective of HPV type*: intention to treat analysis

Study or Subgroup	HPV vaccine Events	HPV vaccine Total	Control Events	Control Total	Weight	Risk Ratio IV, Random, 95% CI
**1.6.1 CIN2+: Cervarix (females, age 15-25; follow-up until 48 months)**						
HPV-008: Cervarix vs. Havrix (f, 15-25; 48)	287	9319	428	9325	18.0%	0.67 [0.58, 0.78]
**Subtotal (95% CI)**		**9319**		**9325**	**18.0%**	**0.67 [0.58, 0.78]**
Total events	287		428			
Heterogeneity: Not applicable						
Test for overall effect: Z = 5.33 (P < 0.00001)						
**1.6.2 CIN2+: Cervarix (females, age 22-29; follow-up until 12 months)**						
HPV-063: Cervarix vs. Aimmugen (f, 22-29; 12)	19	464	41	463	6.4%	0.46 [0.27, 0.78]
**Subtotal (95% CI)**		**464**		**463**	**6.4%**	**0.46 [0.27, 0.78]**
Total events	19		41			
Heterogeneity: Not applicable						
Test for overall effect: Z = 2.86 (P = 0.004)						
**1.6.3 CIN2+: Cervarix (females, age 24-72; follow-up until 36 months)**						
HPV-015: Cervarix vs. Al(OH)3 (f, 24-72; 36)	103	2882	108	2871	13.4%	0.95 [0.73, 1.24]
**Subtotal (95% CI)**		**2882**		**2871**	**13.4%**	**0.95 [0.73, 1.24]**
Total events	103		108			
Heterogeneity: Not applicable						
Test for overall effect: Z = 0.38 (P = 0.70)						
**1.6.4 CIN2+: Gardasil (females, age 15-26; follow-up until 45 months)**						
V501-013: Gardasil vs. AAHS (f, 16-24; 45)	152	2723	166	2732	15.4%	0.92 [0.74, 1.14]
V501-015: Gardasil vs. AAHS (f, 15-26; 36)	269	6087	350	6080	17.7%	0.77 [0.66, 0.90]
**Subtotal (95% CI)**		**8810**		**8812**	**33.1%**	**0.83 [0.70, 0.98]**
Total events	421		516			
Heterogeneity: Tau² = 0.01; Chi² = 1.78, df = 1 (P = 0.18); I² = 44%						
Test for overall effect: Z = 2.15 (P = 0.03)						
**1.6.5 CIN2+: Gardasil (females, age 21-46; follow-up until 48 months)**						
V501-019: Gardasil vs. AAHS (f, 21-46; 48)	62	1911	51	1908	10.1%	1.21 [0.84, 1.75]
**Subtotal (95% CI)**		**1911**		**1908**	**10.1%**	**1.21 [0.84, 1.75]**
Total events	62		51			
Heterogeneity: Not applicable						
Test for overall effect: Z = 1.04 (P = 0.30)						
**1.6.6 PIN2+: Gardasil (males, age 15-27; follow-up until 36 months)**						
V501-020: Gardasil vs. AAHS (m, 15-27; 36)	3	2032	3	2033	1.0%	1.00 [0.20, 4.95]
**Subtotal (95% CI)**		**2032**		**2033**	**1.0%**	**1.00 [0.20, 4.95]**
Total events	3		3			
Heterogeneity: Not applicable						
Test for overall effect: Z = 0.00 (P = 1.00)						
**1.6.7 VIN2+: Gardasil (females, age 15-26; follow-up until 45 months)**						
V501-013: Gardasil vs. AAHS (f, 16-24; 45)	12	2723	15	2732	3.7%	0.80 [0.38, 1.71]
V501-015: Gardasil vs. AAHS (f, 15-26; 36)	6	6087	21	6080	2.7%	0.29 [0.12, 0.71]
**Subtotal (95% CI)**		**8810**		**8812**	**6.4%**	**0.49 [0.18, 1.36]**
Total events	18		36			
Heterogeneity: Tau² = 0.35; Chi² = 2.94, df = 1 (P = 0.09); I² = 66%						
Test for overall effect: Z = 1.37 (P = 0.17)						
**1.6.8 VaIN2+: Gardasil (females, age 15-26; follow-up until 45 months)**						
V501-013: Gardasil vs. AAHS (f, 16-24; 45)	8	2723	17	2732	3.1%	0.47 [0.20, 1.09]
V501-015: Gardasil vs. AAHS (f, 15-26; 36)	6	6087	9	6080	2.1%	0.67 [0.24, 1.87]
**Subtotal (95% CI)**		**8810**		**8812**	**5.2%**	**0.54 [0.28, 1.04]**
Total events	14		26			
Heterogeneity: Tau² = 0.00; Chi² = 0.26, df = 1 (P = 0.61); I² = 0%						
Test for overall effect: Z = 1.85 (P = 0.06)						
**1.6.9 VaIN2+: Gardasil (females, age 21-46; follow-up until 48 months)**						
V501-019: Gardasil vs. AAHS (f, 21-46; 48)	3	1911	1	1908	0.5%	3.00 [0.31, 28.77]
**Subtotal (95% CI)**		**1911**		**1908**	**0.5%**	**3.00 [0.31, 28.77]**
Total events	3		1			
Heterogeneity: Not applicable						
Test for overall effect: Z = 0.95 (P = 0.34)						
**1.6.10 VIN2+ or VaIN2+: Cervarix (females, age 15-25; follow-up until 48 months)**						
HPV-008: Cervarix vs. Havrix (f, 15-25; 48)	22	9319	29	9325	6.0%	0.76 [0.44, 1.32]
**Subtotal (95% CI)**		**9319**		**9325**	**6.0%**	**0.76 [0.44, 1.32]**
Total events	22		29			
Heterogeneity: Not applicable						
Test for overall effect: Z = 0.98 (P = 0.33)						
**Total (95% CI)**		**54268**		**54269**	**100.0%**	**0.78 [0.66, 0.91]**
Total events	952		1239			
Heterogeneity: Tau² = 0.03; Chi² = 25.46, df = 12 (P = 0.01); I² = 53%						
Test for overall effect: Z = 3.11 (P = 0.002)						
Test for subgroup differences: Chi² = 19.34, df = 9 (P = 0.02), I² = 53.5%						

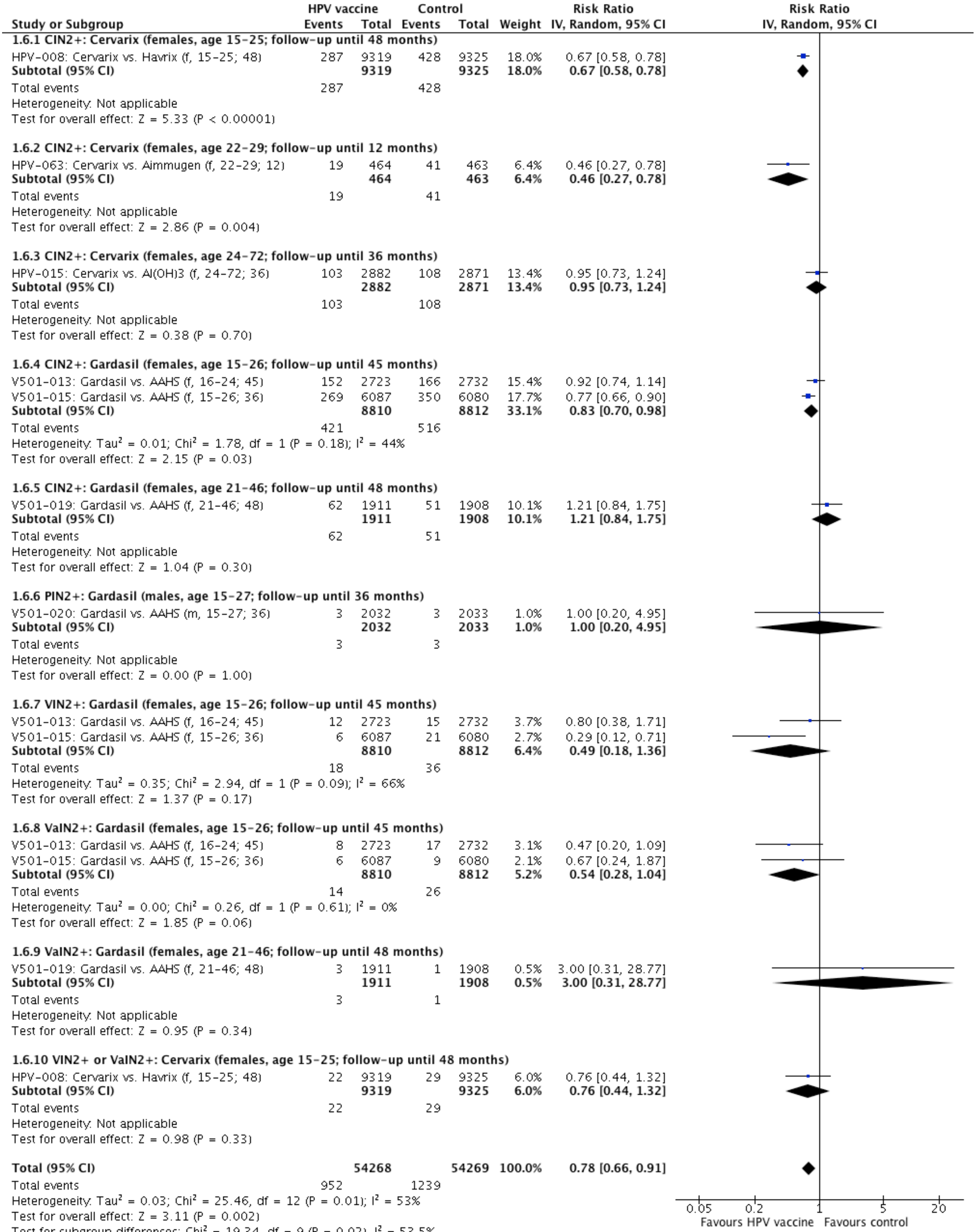

*1.6. Risk ratio for GlaxoSmithKline studies (i.e., HPV-0xx): **0.72 [0.55, 0.93]**; risk ratio for Merck Sharp & Dohme studies (i.e., V50x-xxx): 0.82 [0.66, 1.02]. There were no reports of AIN2⁺ irrespective of HPV type.

1.7. Number of treatment procedures (both surgical and non-surgical treatment) due to HPV-related diseases irrespective of HPV type*: intention to treat analysis

Study or Subgroup	HPV vaccine Events	HPV vaccine Total	Control Events	Control Total	Weight	Risk Ratio IV, Random, 95% CI
**8.4.1 Cervical procedure: Cervarix (females, age 15–25; follow-up until 48 months)**						
HPV-008: Cervarix vs. Havrix (f, 15–25; 48)	212	9319	323	9325	18.4%	0.66 [0.55, 0.78]
**Subtotal (95% CI)**		**9319**		**9325**	**18.4%**	**0.66 [0.55, 0.78]**
Total events	212		323			
Heterogeneity: Not applicable						
Test for overall effect: Z = 4.82 (P < 0.00001)						
**8.4.2 Cervical procedure: Cervarix (females, age 24–72; follow-up until 36 months)**						
HPV-015: Cervarix vs. Al(OH)3 (f, 24–72; 36)	88	2882	110	2871	11.3%	0.80 [0.61, 1.05]
**Subtotal (95% CI)**		**2882**		**2871**	**11.3%**	**0.80 [0.61, 1.05]**
Total events	88		110			
Heterogeneity: Not applicable						
Test for overall effect: Z = 1.61 (P = 0.11)						
**8.4.3 Cervical procedure: Gardasil (females, age 15–26; follow-up until 45 months)**						
V501-013: Gardasil vs. AAHS (f, 16–24; 45)	55	2723	94	2732	8.9%	0.59 [0.42, 0.82]
V501-015: Gardasil vs. AAHS (f, 15–26; 36)	361	6087	472	6080	21.9%	0.76 [0.67, 0.87]
**Subtotal (95% CI)**		**8810**		**8812**	**30.8%**	**0.70 [0.55, 0.89]**
Total events	416		566			
Heterogeneity: Tau^2 = 0.02; Chi^2 = 2.13, df = 1 (P = 0.14); I^2 = 53%						
Test for overall effect: Z = 2.88 (P = 0.004)						
**8.4.4 Cervical procedure: Gardasil (females, age 21–46; follow-up until 48 months)**						
V501-019: Gardasil vs. AAHS (f, 21–46; 48)	128	1911	140	1908	13.9%	0.91 [0.72, 1.15]
**Subtotal (95% CI)**		**1911**		**1908**	**13.9%**	**0.91 [0.72, 1.15]**
Total events	128		140			
Heterogeneity: Not applicable						
Test for overall effect: Z = 0.77 (P = 0.44)						
**8.4.5 External genital lesion (EGL) procedure: Gardasil (females, age 16–24; follow-up until 45 months)**						
V501-013: Gardasil vs. AAHS (f, 16–24; 45)	43	2723	85	2732	7.7%	0.51 [0.35, 0.73]
**Subtotal (95% CI)**		**2723**		**2732**	**7.7%**	**0.51 [0.35, 0.73]**
Total events	43		85			
Heterogeneity: Not applicable						
Test for overall effect: Z = 3.66 (P = 0.0002)						
**8.4.6 External genital lesion (EGL) procedure: Gardasil (females, age 21–46; follow-up until 48 months)**						
V501-019: Gardasil vs. AAHS (f, 21–46; 48)	26	1911	30	1908	4.3%	0.87 [0.51, 1.46]
**Subtotal (95% CI)**		**1911**		**1908**	**4.3%**	**0.87 [0.51, 1.46]**
Total events	26		30			
Heterogeneity: Not applicable						
Test for overall effect: Z = 0.54 (P = 0.59)						
**8.4.7 External genital lesion (EGL) procedure: Gardasil (males, age 15–27; follow-up until 36 months)**						
V501-020: Gardasil vs. AAHS (m, 15–27; 36)	105	2032	162	2033	13.5%	0.65 [0.51, 0.82]
**Subtotal (95% CI)**		**2032**		**2033**	**13.5%**	**0.65 [0.51, 0.82]**
Total events	105		162			
Heterogeneity: Not applicable						
Test for overall effect: Z = 3.57 (P = 0.0004)						
**Total (95% CI)**		**29588**		**29589**	**100.0%**	**0.71 [0.63, 0.80]**
Total events	1018		1416			
Heterogeneity: Tau^2 = 0.01; Chi^2 = 12.72, df = 7 (P = 0.08); I^2 = 45%						
Test for overall effect: Z = 5.70 (P < 0.00001)						
Test for subgroup differences: Chi^2 = 10.43, df = 6 (P = 0.11), I^2 = 42.4%						

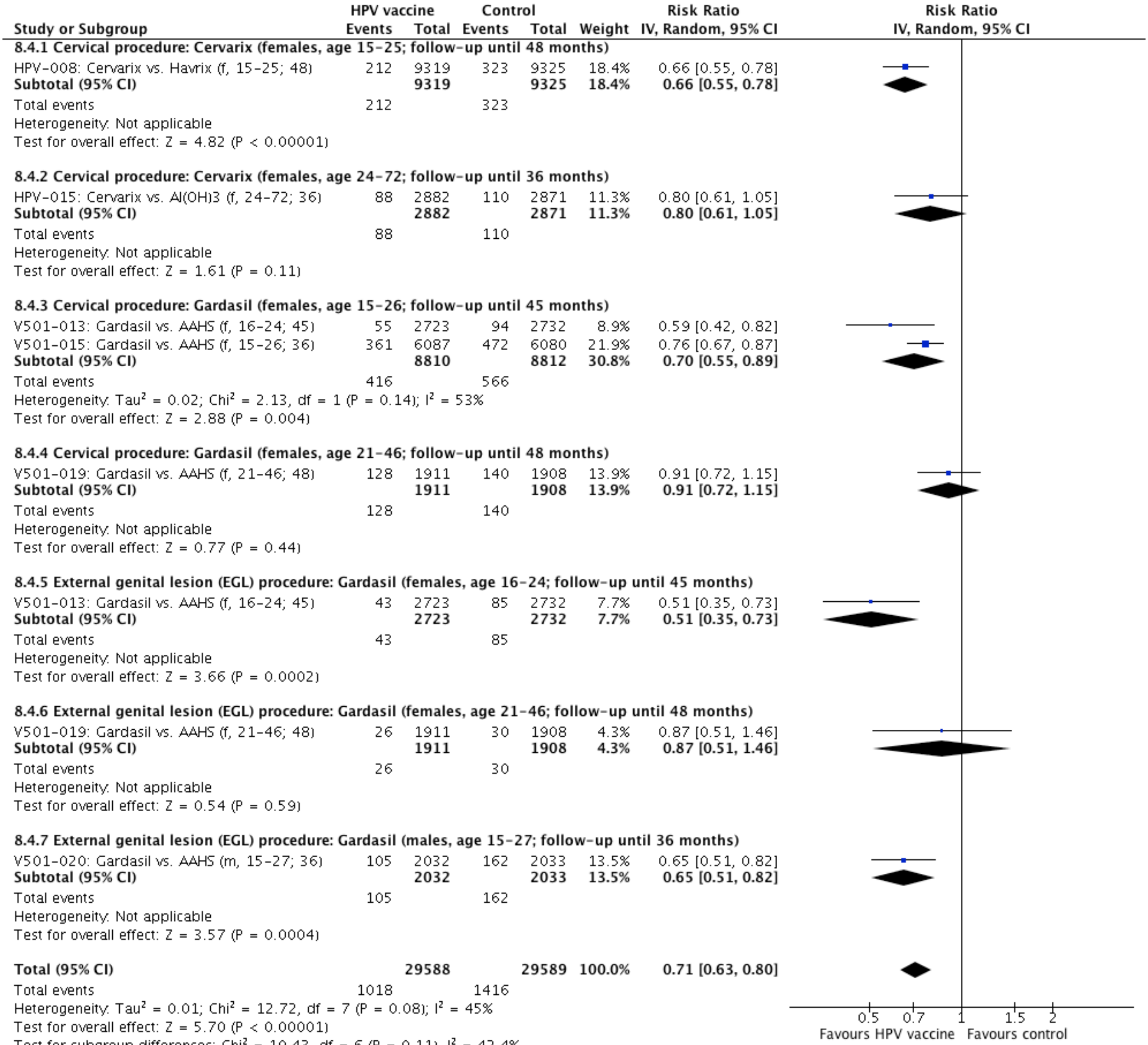

*1.7. Risk ratio for GlaxoSmithKline studies (i.e., HPV-0xx): **0.70 [0.59, 0.84]**; risk ratio for Merck Sharp & Dohme studies (i.e., V50x-xxx): **0.71 [0.60, 0.83]**. Only cervical procedure: 844 in the HPV vaccine group vs. 1,139 in the control group, risk ratio **0.74 [0.65, 0.84]**; only EGL procedure: 174 vs. 277, risk ratio **0.63 [0.50, 0.80]**.

1.8. Fatal harms*: intention to treat analysis

Study or Subgroup	HPV vaccine Events	HPV vaccine Total	Control Events	Control Total	Weight	Risk Ratio IV, Random, 95% CI
**9.1.1 Fatal harms: Cervarix vs. Al(OH)3 (females, age 24-72; follow-up until 36 months)**						
HPV-015: Cervarix vs. Al(OH)3 (f, 24-72; 36)	13	2882	5	2871	18.7%	2.59 [0.92, 7.26]
**Subtotal (95% CI)**		**2882**		**2871**	**18.7%**	**2.59 [0.92, 7.26]**
Total events	13		5			
Heterogeneity: Not applicable						
Test for overall effect: Z = 1.81 (P = 0.07)						
**9.1.2 Fatal harms: Cervarix vs. Aimmugen: hepatitis A vaccine (females, age 19-25; follow-up until 7 months)**						
HPV-032: Cervarix vs. Aimmugen (f, 19-25; 7)	1	519	0	521	3.3%	3.01 [0.12, 73.76]
**Subtotal (95% CI)**		**519**		**521**	**3.3%**	**3.01 [0.12, 73.76]**
Total events	1		0			
Heterogeneity: Not applicable						
Test for overall effect: Z = 0.68 (P = 0.50)						
**9.1.3 Fatal harms: Cervarix vs. Engerix: hepatitis B vaccine (females, age 26-46; follow-up until 12 months)**						
HPV-069: Cervarix vs. Engerix (f, 26-46; 12)	1	606	0	606	3.3%	3.00 [0.12, 73.50]
**Subtotal (95% CI)**		**606**		**606**	**3.3%**	**3.00 [0.12, 73.50]**
Total events	1		0			
Heterogeneity: Not applicable						
Test for overall effect: Z = 0.67 (P = 0.50)						
**9.1.4 Fatal harms: Cervarix vs. Havrix: hepatitis A vaccine (females, age 15-25; follow-up until 48 months)**						
HPV-008: Cervarix vs. Havrix (f, 15-25; 48)	10	9319	13	9325	23.2%	0.77 [0.34, 1.75]
**Subtotal (95% CI)**		**9319**		**9325**	**23.2%**	**0.77 [0.34, 1.75]**
Total events	10		13			
Heterogeneity: Not applicable						
Test for overall effect: Z = 0.62 (P = 0.53)						
**9.1.5 Fatal harms: Gardasil vs. AAHS (females, age 15-26; follow-up until 45 months)**						
V501-013: Gardasil vs. AAHS (f, 16-24; 45)	2	2723	3	2732	9.0%	0.67 [0.11, 4.00]
V501-015: Gardasil vs. AAHS (f, 15-26; 36)	7	6087	5	6080	16.6%	1.40 [0.44, 4.40]
**Subtotal (95% CI)**		**8810**		**8812**	**25.6%**	**1.13 [0.43, 2.96]**
Total events	9		8			
Heterogeneity: $Tau^2 = 0.00$; $Chi^2 = 0.46$, df = 1 (P = 0.50); $I^2 = 0\%$						
Test for overall effect: Z = 0.24 (P = 0.81)						
**9.1.6 Fatal harms: Gardasil vs. AAHS (females, age 21-46; follow-up until 48 months)**						
V501-019: Gardasil vs. AAHS (f, 21-46; 48)	7	1911	1	1908	7.0%	6.99 [0.86, 56.75]
**Subtotal (95% CI)**		**1911**		**1908**	**7.0%**	**6.99 [0.86, 56.75]**
Total events	7		1			
Heterogeneity: Not applicable						
Test for overall effect: Z = 1.82 (P = 0.07)						
**9.1.7 Fatal harms: Gardasil vs. AAHS (males, age 15-27; follow-up until 36 months)**						
V501-020: Gardasil vs. AAHS (m, 15-27; 36)	3	2032	10	2033	14.4%	0.30 [0.08, 1.09]
**Subtotal (95% CI)**		**2032**		**2033**	**14.4%**	**0.30 [0.08, 1.09]**
Total events	3		10			
Heterogeneity: Not applicable						
Test for overall effect: Z = 1.83 (P = 0.07)						
**9.1.8 Fatal harms: HPV 16 vaccine vs. AAHS (females, age 16-25; follow-up until 48 months)**						
V501-005: HPV 16 vaccine vs. AAHS (f, 16-25; 48)	1	1204	1	1205	4.3%	1.00 [0.06, 15.98]
**Subtotal (95% CI)**		**1204**		**1205**	**4.3%**	**1.00 [0.06, 15.98]**
Total events	1		1			
Heterogeneity: Not applicable						
Test for overall effect: Z = 0.00 (P = 1.00)						
**Total (95% CI)**		**27283**		**27281**	**100.0%**	**1.19 [0.65, 2.19]**
Total events	45		38			
Heterogeneity: $Tau^2 = 0.24$; $Chi^2 = 11.49$, df = 8 (P = 0.18); $I^2 = 30\%$						
Test for overall effect: Z = 0.56 (P = 0.58)						
Test for subgroup differences: $Chi^2 = 11.02$, df = 7 (P = 0.14), $I^2 = 36.5\%$						

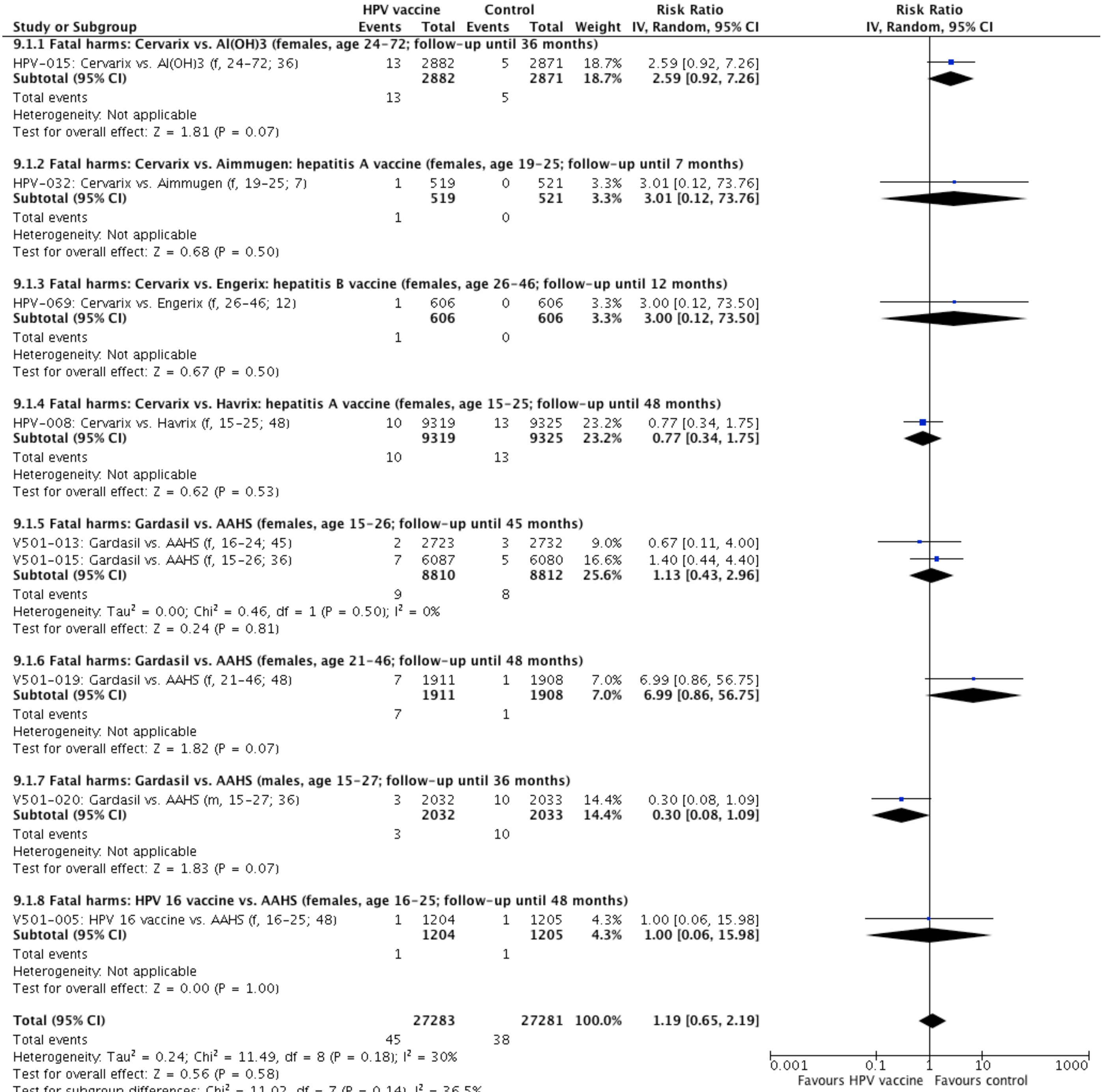

*1.8. Risk ratio for GlaxoSmithKline studies (i.e., HPV-0xx): 1.43 [0.65, 3.15]; risk ratio for Merck Sharp & Dohme studies (i.e., V50x-xxx): 1.08 [0.40, 2.96]. The most common fatal serious harms were: 'road traffic accident' (five in the HPV vaccine group and seven in the control group, risk ratio 0.77 [0.24, 2.46]); 'completed suicide' (four and eight, risk ratio 0.58 [0.15, 2.19]); 'cardiorespiratory arrest' (three and two, risk ratio 0.99 [0.13, 7.65]); 'gunshot wound' (two and three, risk ratio 0.74 [0.09, 5.85]); and 'homicide' (two and two, risk ratio 0.95 [0.14, 6.50]). The fatal serious harms most increased by the HPV vaccines were: 'cardiac arrest' (two in the HPV vaccine group and none in the control group, risk ratio 3.00 [0.31, 28.82]); 'traumatic intracranial haemorrhage' (two and none, risk ratio 3.00 [0.31, 28.82]); 'systemic lupus erythematosus' (two and none, risk ratio 3.00 [0.31, 28.82]); 'metastases to lung' (two and none, risk ratio 3.00 [0.31, 28.82]); and 'renal failure acute' (two and none, risk ratio 3.00 [0.31, 28.82]). The fatal serious harms most decreased by the HPV vaccines were: 'completed suicide' (four in the HPV vaccine group and eight in the control group, risk ratio 0.58 [0.15, 2.19]); and 'road traffic accident' (five and seven, risk ratio 0.77 [0.24, 2.46]).

1.9. Serious harms*: intention to treat analysis

Study or Subgroup	HPV vaccine Events	HPV vaccine Total	Control Events	Control Total	Weight	Risk Ratio IV, Random, 95% CI
**10.1.1 Serious harms: Cervarix vs. Al(OH)3 (females, age 9-17; follow-up until 12 months)**						
HPV-058: Cervarix vs. Al(OH)3 (f, 9-17; 12)	5	374	2	376	0.2%	2.51 [0.49, 12.87]
**Subtotal (95% CI)**		**374**		**376**	**0.2%**	**2.51 [0.49, 12.87]**
Total events	5		2			
Heterogeneity: Not applicable						
Test for overall effect: Z = 1.11 (P = 0.27)						
**10.1.2 Serious harms: Cervarix vs. Al(OH)3 (females, age 15-25; follow-up until 7 months)**						
HPV-001: Cervarix vs. Al(OH)3 (f, 15-26; 27)	22	560	19	553	1.4%	1.14 [0.63, 2.09]
HPV-038: Cervarix vs. Al(OH)3 (f, 15-25; 7)	2	149	1	76	0.1%	1.02 [0.09, 11.07]
**Subtotal (95% CI)**		**709**		**629**	**1.5%**	**1.14 [0.63, 2.04]**
Total events	24		20			
Heterogeneity: Tau² = 0.00; Chi² = 0.01, df = 1 (P = 0.93); I² = 0%						
Test for overall effect: Z = 0.43 (P = 0.67)						
**10.1.3 Serious harms: Cervarix vs. Al(OH)3 (females, age 18-35; follow-up until 12 months)**						
HPV-003: Cervarix vs. Al(OH)3 (f, 18-30; 12)	1	31	0	30	0.1%	2.91 [0.12, 68.66]
HPV-023: Cervarix vs. Al(OH)3 (f, 21-32; 36)	4	222	5	211	0.3%	0.76 [0.21, 2.79]
HPV-031: Cervarix vs. Al(OH)3 (f, 18-35; 7)	2	176	4	178	0.2%	0.51 [0.09, 2.73]
HPV-035: Cervarix vs. Al(OH)3 (f, 18-35; 7)	3	150	1	150	0.1%	3.00 [0.32, 28.52]
**Subtotal (95% CI)**		**579**		**569**	**0.6%**	**0.94 [0.38, 2.30]**
Total events	10		10			
Heterogeneity: Tau² = 0.00; Chi² = 2.13, df = 3 (P = 0.55); I² = 0%						
Test for overall effect: Z = 0.14 (P = 0.89)						
**10.1.4 Serious harms: Cervarix vs. Al(OH)3 (females, age 24-72; follow-up until 36 months)**						
HPV-015: Cervarix vs. Al(OH)3 (f, 24-72; 36)	286	2882	266	2871	20.5%	1.07 [0.91, 1.26]
**Subtotal (95% CI)**		**2882**		**2871**	**20.5%**	**1.07 [0.91, 1.26]**
Total events	286		266			
Heterogeneity: Not applicable						
Test for overall effect: Z = 0.85 (P = 0.40)						
**10.1.5 Serious harms: Cervarix vs. Aimmugen: hepatitis A vaccine (females, age 22-29; follow-up until 12 months)**						
HPV-063: Cervarix vs. Aimmugen (f, 22-29; 12)	26	519	34	521	2.1%	0.77 [0.47, 1.26]
**Subtotal (95% CI)**		**519**		**521**	**2.1%**	**0.77 [0.47, 1.26]**
Total events	26		34			
Heterogeneity: Not applicable						
Test for overall effect: Z = 1.04 (P = 0.30)						
**10.1.6 Serious harms: Cervarix vs. Engerix: hepatitis B vaccine (females and males, age 9-16; follow-up until 72 months)**						
HPV-030: Cervarix vs. Engerix (f, 9-15; 12)	1	247	0	246	0.1%	2.99 [0.12, 72.99]
HPV-040: Cervarix vs. Engerix (f & m, 12-16; 72)	58	2436	25	1267	2.4%	1.21 [0.76, 1.92]
**Subtotal (95% CI)**		**2683**		**1513**	**2.5%**	**1.23 [0.78, 1.95]**
Total events	59		25			
Heterogeneity: Tau² = 0.00; Chi² = 0.30, df = 1 (P = 0.58); I² = 0%						
Test for overall effect: Z = 0.88 (P = 0.38)						
**10.1.7 Serious harms: Cervarix vs. Engerix: hepatitis B vaccine (females, age 26-46; follow-up until 12 months)**						
HPV-069: Cervarix vs. Engerix (f, 26-46; 12)	3	606	3	606	0.2%	1.00 [0.20, 4.93]
**Subtotal (95% CI)**		**606**		**606**	**0.2%**	**1.00 [0.20, 4.93]**
Total events	3		3			
Heterogeneity: Not applicable						
Test for overall effect: Z = 0.00 (P = 1.00)						
**10.1.8 Serious harms: Cervarix vs. Havrix: hepatitis A vaccine (females, age 9-15; follow-up until 12 months)**						
HPV-013: Cervarix vs. Havrix (f, 10-14; 12)	10	1035	13	1032	0.8%	0.77 [0.34, 1.74]
HPV-033: Cervarix vs. Havrix (f, 9-15; 7)	0	160	1	161	0.1%	0.34 [0.01, 8.17]
**Subtotal (95% CI)**		**1195**		**1193**	**0.8%**	**0.73 [0.33, 1.61]**
Total events	10		14			
Heterogeneity: Tau² = 0.00; Chi² = 0.24, df = 1 (P = 0.62); I² = 0%						
Test for overall effect: Z = 0.78 (P = 0.43)						
**10.1.9 Serious harms: Cervarix vs. Havrix: hepatitis A vaccine (females, age 15-25; follow-up until 48 months)**						
HPV-008: Cervarix vs. Havrix (f, 15-25; 48)	835	9319	829	9325	61.5%	1.01 [0.92, 1.10]
**Subtotal (95% CI)**		**9319**		**9325**	**61.5%**	**1.01 [0.92, 1.10]**
Total events	835		829			
Heterogeneity: Not applicable						
Test for overall effect: Z = 0.17 (P = 0.87)						
**10.1.10 Serious harms: Cervarix vs. Twinrix: hepatitis A and B vaccine (females, age 8-15; follow-up until 12 months)**						
HPV-029: Cervarix vs. Twinrix (f, 8-15; 12)	3	270	4	271	0.2%	0.75 [0.17, 3.33]
**Subtotal (95% CI)**		**270**		**271**	**0.2%**	**0.75 [0.17, 3.33]**
Total events	3		4			
Heterogeneity: Not applicable						
Test for overall effect: Z = 0.37 (P = 0.71)						
**10.1.11 Serious harms: Gardasil vs. carrier solution (females, age 9-16; follow-up until 18 months)**						
V501-018: Gardasil vs. carrier solu. (f&m,9-16;18)	6	1184	0	597	0.1%	6.56 [0.37, 116.26]
**Subtotal (95% CI)**		**1184**		**597**	**0.1%**	**6.56 [0.37, 116.26]**
Total events	6		0			
Heterogeneity: Not applicable						
Test for overall effect: Z = 1.28 (P = 0.20)						
**10.1.12 Serious harms: Gardasil vs. AAHS (females, age 15-26; follow-up until 45 months)**						
V501-013: Gardasil vs. AAHS (f, 16-24; 45)	49	2723	45	2732	3.2%	1.09 [0.73, 1.63]
V501-015: Gardasil vs. AAHS (f, 15-26; 36)	46	6087	56	6080	3.4%	0.82 [0.56, 1.21]
**Subtotal (95% CI)**		**8810**		**8812**	**6.6%**	**0.94 [0.71, 1.25]**
Total events	95		101			
Heterogeneity: Tau² = 0.00; Chi² = 1.01, df = 1 (P = 0.31); I² = 1%						
Test for overall effect: Z = 0.41 (P = 0.68)						
**10.1.13 Serious harms: Gardasil vs. AAHS (females, age 21-46; follow-up until 48 months)**						
V501-019: Gardasil vs. AAHS (f, 21-46; 48)	14	1911	16	1908	1.0%	0.87 [0.43, 1.78]
**Subtotal (95% CI)**		**1911**		**1908**	**1.0%**	**0.87 [0.43, 1.78]**
Total events	14		16			
Heterogeneity: Not applicable						
Test for overall effect: Z = 0.37 (P = 0.71)						
**10.1.14 Serious harms: Gardasil vs. AAHS (males, age 15-27; follow-up until 36 months)**						
V501-020: Gardasil vs. AAHS (m, 15-27; 36)	8	2032	11	2033	0.6%	0.73 [0.29, 1.81]
**Subtotal (95% CI)**		**2032**		**2033**	**0.6%**	**0.73 [0.29, 1.81]**
Total events	8		11			
Heterogeneity: Not applicable						
Test for overall effect: Z = 0.69 (P = 0.49)						
**10.1.15 Serious harms: Gardasil 9 vs. saline placebo (females, age 12-26; follow-up until 7 months)**						
V503-006: Gardasil 9 vs. saline (f, 12-26;7)	3	618	3	306	0.2%	0.50 [0.10, 2.44]
**Subtotal (95% CI)**		**618**		**306**	**0.2%**	**0.50 [0.10, 2.44]**
Total events	3		3			
Heterogeneity: Not applicable						
Test for overall effect: Z = 0.86 (P = 0.39)						
**10.1.16 Serious harms: HPV 16 vaccine vs. AAHS (females, age 16-25; follow-up until 48 months)**						
V501-005: HPV 16 vaccine vs. AAHS (f, 16-25; 48)	17	1204	19	1205	1.2%	0.90 [0.47, 1.71]
**Subtotal (95% CI)**		**1204**		**1205**	**1.2%**	**0.90 [0.47, 1.71]**
Total events	17		19			
Heterogeneity: Not applicable						
Test for overall effect: Z = 0.33 (P = 0.74)						
**Total (95% CI)**		**34895**		**32735**	**100.0%**	**1.01 [0.94, 1.08]**
Total events	1404		1357			
Heterogeneity: Tau² = 0.00; Chi² = 11.70, df = 22 (P = 0.96); I² = 0%						
Test for overall effect: Z = 0.26 (P = 0.79)						
Test for subgroup differences: Chi² = 8.00, df = 15 (P = 0.92), I² = 0%						

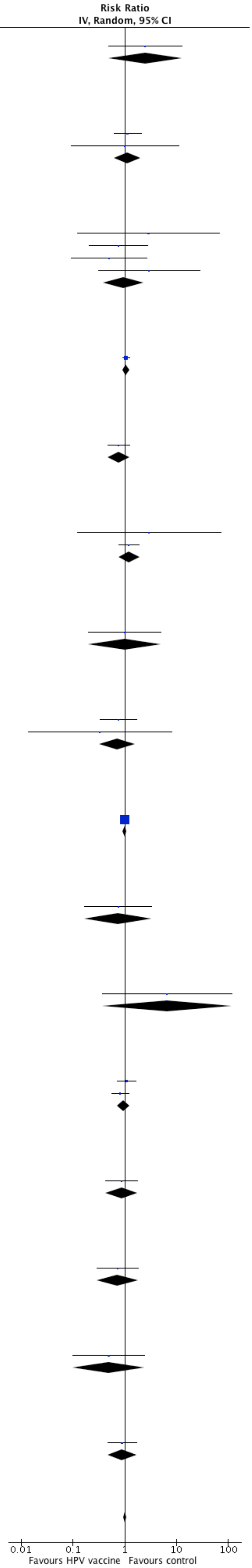

*1.9. Risk ratio for GlaxoSmithKline studies (i.e., HPV-0xx): 1.02 [0.95, 1.10]; risk ratio for Merck Sharp & Dohme studies (i.e., V50x-xxx): 0.91 [0.73, 1.15].

1.10. Serious harms judged as ‘definitely associated’* with chronic regional pain syndrome (CRPS): intention to treat analysis

Study or Subgroup	HPV vaccine Events	HPV vaccine Total	Control Events	Control Total	Weight	Risk Ratio IV, Random, 95% CI
**1.10.1 Serious harms 'definitely associated' with CRPS: Cervarix vs. Al(OH)3 (females, age 18-35; follow-up until 7 months)**						
HPV-035: Cervarix vs. Al(OH)3 (f, 18-35; 7)	4	150	0	150	1.3%	9.00 [0.49, 165.71]
**Subtotal (95% CI)**		**150**		**150**	**1.3%**	**9.00 [0.49, 165.71]**
Total events	4		0			
Heterogeneity: Not applicable						
Test for overall effect: Z = 1.48 (P = 0.14)						
**1.10.2 Serious harms 'definitely associated' with CRPS: Cervarix vs. Al(OH)3 (females, age 24-72; follow-up until 36 months)**						
HPV-015: Cervarix vs. Al(OH)3 (f, 24-72; 36)	15	2882	9	2871	15.9%	1.66 [0.73, 3.79]
**Subtotal (95% CI)**		**2882**		**2871**	**15.9%**	**1.66 [0.73, 3.79]**
Total events	15		9			
Heterogeneity: Not applicable						
Test for overall effect: Z = 1.20 (P = 0.23)						
**1.10.3 Serious harms 'definitely associated' with CRPS: Cervarix vs. Aimmugen: hepatitis A vaccine (females, age 22-29; follow-up until 12 months)**						
HPV-063: Cervarix vs. Aimmugen (f, 22-29; 12)	0	375	1	377	1.1%	0.34 [0.01, 8.20]
**Subtotal (95% CI)**		**375**		**377**	**1.1%**	**0.34 [0.01, 8.20]**
Total events	0		1			
Heterogeneity: Not applicable						
Test for overall effect: Z = 0.67 (P = 0.50)						
**1.10.4 Serious harms 'definitely associated' with CRPS: Cervarix vs. Engerix: hepatitis B vaccine (females and males, age 9-16; follow-up until 72 months)**						
HPV-040: Cervarix vs. Engerix (f & m, 12-16; 72)	3	2436	0	1267	1.2%	3.64 [0.19, 70.46]
**Subtotal (95% CI)**		**2436**		**1267**	**1.2%**	**3.64 [0.19, 70.46]**
Total events	3		0			
Heterogeneity: Not applicable						
Test for overall effect: Z = 0.86 (P = 0.39)						
**1.10.5 Serious harms 'definitely associated' with CRPS: Cervarix vs. Havrix: hepatitis A vaccine (females, age 10-14; follow-up until 12 months)**						
HPV-013: Cervarix vs. Havrix (f, 10-14; 12)	2	1035	2	1032	2.8%	1.00 [0.14, 7.07]
**Subtotal (95% CI)**		**1035**		**1032**	**2.8%**	**1.00 [0.14, 7.07]**
Total events	2		2			
Heterogeneity: Not applicable						
Test for overall effect: Z = 0.00 (P = 1.00)						
**1.10.6 Serious harms 'definitely associated' with CRPS: Cervarix vs. Havrix: hepatitis A vaccine (females, age 15-25; follow-up until 48 months)**						
HPV-008: Cervarix vs. Havrix (f, 15-25; 48)	59	9319	39	9325	66.3%	1.51 [1.01, 2.27]
**Subtotal (95% CI)**		**9319**		**9325**	**66.3%**	**1.51 [1.01, 2.27]**
Total events	59		39			
Heterogeneity: Not applicable						
Test for overall effect: Z = 2.01 (P = 0.04)						
**1.10.7 Serious harms 'definitely associated' with CRPS: Gardasil vs. carrier solution (females and males, age 9-16; follow-up until 18 months)**						
V501-018: Gardasil vs. carrier solu. (f&m,9-16;18)	1	1184	0	597	1.1%	1.51 [0.06, 37.11]
**Subtotal (95% CI)**		**1184**		**597**	**1.1%**	**1.51 [0.06, 37.11]**
Total events	1		0			
Heterogeneity: Not applicable						
Test for overall effect: Z = 0.25 (P = 0.80)						
**1.10.8 Serious harms 'definitely associated' with CRPS: Gardasil vs. AAHS (females, age 15-26; follow-up until 45 months)**						
V501-013: Gardasil vs. AAHS (f, 16-24; 45)	1	2723	2	2732	1.9%	0.50 [0.05, 5.53]
V501-015: Gardasil vs. AAHS (f, 15-26; 36)	4	6087	3	6080	4.8%	1.33 [0.30, 5.95]
**Subtotal (95% CI)**		**8810**		**8812**	**6.7%**	**1.01 [0.28, 3.61]**
Total events	5		5			
Heterogeneity: $Tau^2 = 0.00$; $Chi^2 = 0.46$, df = 1 (P = 0.50); $I^2 = 0\%$						
Test for overall effect: Z = 0.02 (P = 0.98)						
**1.10.9 Serious harms 'definitely associated' with CRPS: Gardasil vs. AAHS (females, age 21-46; follow-up until 48 months)**						
V501-019: Gardasil vs. AAHS (f, 21-46; 48)	4	1911	0	1908	1.3%	8.99 [0.48, 166.79]
**Subtotal (95% CI)**		**1911**		**1908**	**1.3%**	**8.99 [0.48, 166.79]**
Total events	4		0			
Heterogeneity: Not applicable						
Test for overall effect: Z = 1.47 (P = 0.14)						
**1.10.10 Serious harms 'definitely associated' with CRPS: Gardasil vs. AAHS (males, age 15-27; follow-up until 36 months)**						
V501-020: Gardasil vs. AAHS (m, 15-27; 36)	1	2032	0	2033	1.1%	3.00 [0.12, 73.64]
**Subtotal (95% CI)**		**2032**		**2033**	**1.1%**	**3.00 [0.12, 73.64]**
Total events	1		0			
Heterogeneity: Not applicable						
Test for overall effect: Z = 0.67 (P = 0.50)						
**1.10.11 Serious harms 'definitely associated' with CRPS: HPV 16 vaccine vs. AAHS (females, age 16-25; follow-up until 48 months)**						
V501-005: HPV 16 vaccine vs. AAHS (f, 16-25; 48)	1	1204	1	1205	1.4%	1.00 [0.06, 15.98]
**Subtotal (95% CI)**		**1204**		**1205**	**1.4%**	**1.00 [0.06, 15.98]**
Total events	1		1			
Heterogeneity: Not applicable						
Test for overall effect: Z = 0.00 (P = 1.00)						
**Total (95% CI)**		**31338**		**29577**	**100.0%**	**1.54 [1.11, 2.14]**
Total events	95		57			
Heterogeneity: $Tau^2 = 0.00$; $Chi^2 = 5.37$, df = 11 (P = 0.91); $I^2 = 0\%$						
Test for overall effect: Z = 2.58 (P = 0.010)						
Test for subgroup differences: $Chi^2 = 4.92$, df = 10 (P = 0.90), $I^2 = 0\%$						

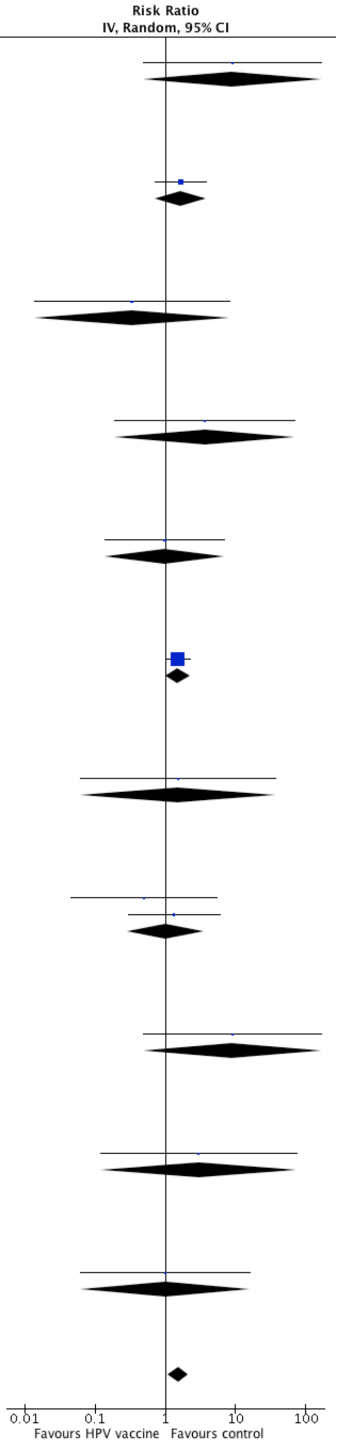

*1.10. Risk ratio for GlaxoSmithKline studies (i.e., HPV-0xx): **1.55 [1.09, 2.20]**; risk ratio for Merck Sharp & Dohme studies (i.e., V50x-xxx): 1.47 [0.56, 3.89]. We asked a physician with clinical expertise in CRPS to assess the reported MedDRA preferred terms as 'definitely,' 'probably,' 'probably not' or 'definitely not' associated with CRPS. We sent an Excel sheet to the physician with all the reported MedDRA terms. The physician was blinded, as the Excel sheet contained no outcome data. When the physician had assessed all the MedDRA terms, we synthesized the data for those MedDRA terms that the physician judged 'definitely' associated with CRPS and compared it to the reported serious harms.

1.11. Serious harms judged as ‘definitely associated’* with postural orthostatic tachycardia syndrome (POTS): intention to treat analysis

Study or Subgroup	HPV vaccine Events	HPV vaccine Total	Control Events	Control Total	Weight	Risk Ratio IV, Random, 95% CI
**1.11.1 Serious harms 'definitely associated' with POTS: Cervarix vs. Al(OH)3 (females, age 18-35; follow-up until 7 months)**						
HPV-035: Cervarix vs. Al(OH)3 (f, 18-35; 7)	3	150	0	150	2.5%	7.00 [0.36, 134.36]
**Subtotal (95% CI)**		**150**		**150**	**2.5%**	**7.00 [0.36, 134.36]**
Total events	3		0			
Heterogeneity: Not applicable						
Test for overall effect: Z = 1.29 (P = 0.20)						
**1.11.2 Serious harms 'definitely associated' with POTS: Cervarix vs. Al(OH)3 (females, age 24-72; follow-up until 36 months)**						
HPV-015: Cervarix vs. Al(OH)3 (f, 24-72; 36)	9	2882	5	2871	18.3%	1.79 [0.60, 5.34]
**Subtotal (95% CI)**		**2882**		**2871**	**18.3%**	**1.79 [0.60, 5.34]**
Total events	9		5			
Heterogeneity: Not applicable						
Test for overall effect: Z = 1.05 (P = 0.29)						
**1.11.3 Serious harms 'definitely associated' with POTS: Cervarix vs. Aimmugen: hepatitis A vaccine (females, age 22-29; follow-up until 12 months)**						
HPV-063: Cervarix vs. Aimmugen (f, 22-29; 12)	0	375	1	377	2.1%	0.34 [0.01, 8.20]
**Subtotal (95% CI)**		**375**		**377**	**2.1%**	**0.34 [0.01, 8.20]**
Total events	0		1			
Heterogeneity: Not applicable						
Test for overall effect: Z = 0.67 (P = 0.50)						
**1.11.4 Serious harms 'definitely associated' with POTS: Cervarix vs. Engerix: hepatitis B vaccine (females and males, age 9-16; follow-up until 72 months)**						
HPV-040: Cervarix vs. Engerix (f & m, 12-16; 72)	3	2436	1	1267	4.3%	1.56 [0.16, 14.99]
**Subtotal (95% CI)**		**2436**		**1267**	**4.3%**	**1.56 [0.16, 14.99]**
Total events	3		1			
Heterogeneity: Not applicable						
Test for overall effect: Z = 0.39 (P = 0.70)						
**1.11.5 Serious harms 'definitely associated' with POTS: Cervarix vs. Havrix: hepatitis A vaccine (females, age 10-14; follow-up until 12 months)**						
HPV-013: Cervarix vs. Havrix (f, 10-14; 12)	0	1035	2	1032	2.4%	0.20 [0.01, 4.15]
**Subtotal (95% CI)**		**1035**		**1032**	**2.4%**	**0.20 [0.01, 4.15]**
Total events	0		2			
Heterogeneity: Not applicable						
Test for overall effect: Z = 1.04 (P = 0.30)						
**1.11.6 Serious harms 'definitely associated' with POTS: Cervarix vs. Havrix: hepatitis A vaccine (females, age 15-25; follow-up until 48 months)**						
HPV-008: Cervarix vs. Havrix (f, 15-25; 48)	28	9319	12	9325	47.9%	2.33 [1.19, 4.59]
**Subtotal (95% CI)**		**9319**		**9325**	**47.9%**	**2.33 [1.19, 4.59]**
Total events	28		12			
Heterogeneity: Not applicable						
Test for overall effect: Z = 2.46 (P = 0.01)						
**1.11.7 Serious harms 'definitely associated' with POTS: Gardasil vs. AAHS (females, age 15-26; follow-up until 45 months)**						
V501-013: Gardasil vs. AAHS (f, 16-24; 45)	3	2723	3	2732	8.5%	1.00 [0.20, 4.97]
V501-015: Gardasil vs. AAHS (f, 15-26; 36)	3	6087	1	6080	4.3%	3.00 [0.31, 28.80]
**Subtotal (95% CI)**		**8810**		**8812**	**12.8%**	**1.44 [0.39, 5.33]**
Total events	6		4			
Heterogeneity: $Tau^2 = 0.00$; $Chi^2 = 0.60$, df = 1 (P = 0.44); $I^2 = 0\%$						
Test for overall effect: Z = 0.55 (P = 0.58)						
**1.11.8 Serious harms 'definitely associated' with POTS: Gardasil vs. AAHS (females, age 21-46; follow-up until 48 months)**						
V501-019: Gardasil vs. AAHS (f, 21-46; 48)	4	1911	0	1908	2.6%	8.99 [0.48, 166.79]
**Subtotal (95% CI)**		**1911**		**1908**	**2.6%**	**8.99 [0.48, 166.79]**
Total events	4		0			
Heterogeneity: Not applicable						
Test for overall effect: Z = 1.47 (P = 0.14)						
**1.11.9 Serious harms 'definitely associated' with POTS: Gardasil vs. AAHS (males, age 15-27; follow-up until 36 months)**						
V501-020: Gardasil vs. AAHS (m, 15-27; 36)	1	2032	0	2033	2.1%	3.00 [0.12, 73.64]
**Subtotal (95% CI)**		**2032**		**2033**	**2.1%**	**3.00 [0.12, 73.64]**
Total events	1		0			
Heterogeneity: Not applicable						
Test for overall effect: Z = 0.67 (P = 0.50)						
**1.11.10 Serious harms 'definitely associated' with POTS: Gardasil 9 vs. saline placebo (females, age 12-26; follow-up until 7 months)**						
V503-006: Gardasil 9 vs. saline (f, 12-26;7)	1	618	0	306	2.1%	1.49 [0.06, 36.42]
**Subtotal (95% CI)**		**618**		**306**	**2.1%**	**1.49 [0.06, 36.42]**
Total events	1		0			
Heterogeneity: Not applicable						
Test for overall effect: Z = 0.24 (P = 0.81)						
**1.11.11 Serious harms 'definitely associated' with POTS: HPV 16 vaccine vs. AAHS (females, age 16-25; follow-up until 48 months)**						
V501-005: HPV 16 vaccine vs. AAHS (f, 16-25; 48)	1	1204	1	1205	2.8%	1.00 [0.06, 15.98]
**Subtotal (95% CI)**		**1204**		**1205**	**2.8%**	**1.00 [0.06, 15.98]**
Total events	1		1			
Heterogeneity: Not applicable						
Test for overall effect: Z = 0.00 (P = 1.00)						
**Total (95% CI)**		**30772**		**29286**	**100.0%**	**1.92 [1.21, 3.07]**
Total events	56		26			
Heterogeneity: $Tau^2 = 0.00$; $Chi^2 = 6.55$, df = 11 (P = 0.83); $I^2 = 0\%$						
Test for overall effect: Z = 2.74 (P = 0.006)						
Test for subgroup differences: $Chi^2 = 5.96$, df = 10 (P = 0.82), $I^2 = 0\%$						

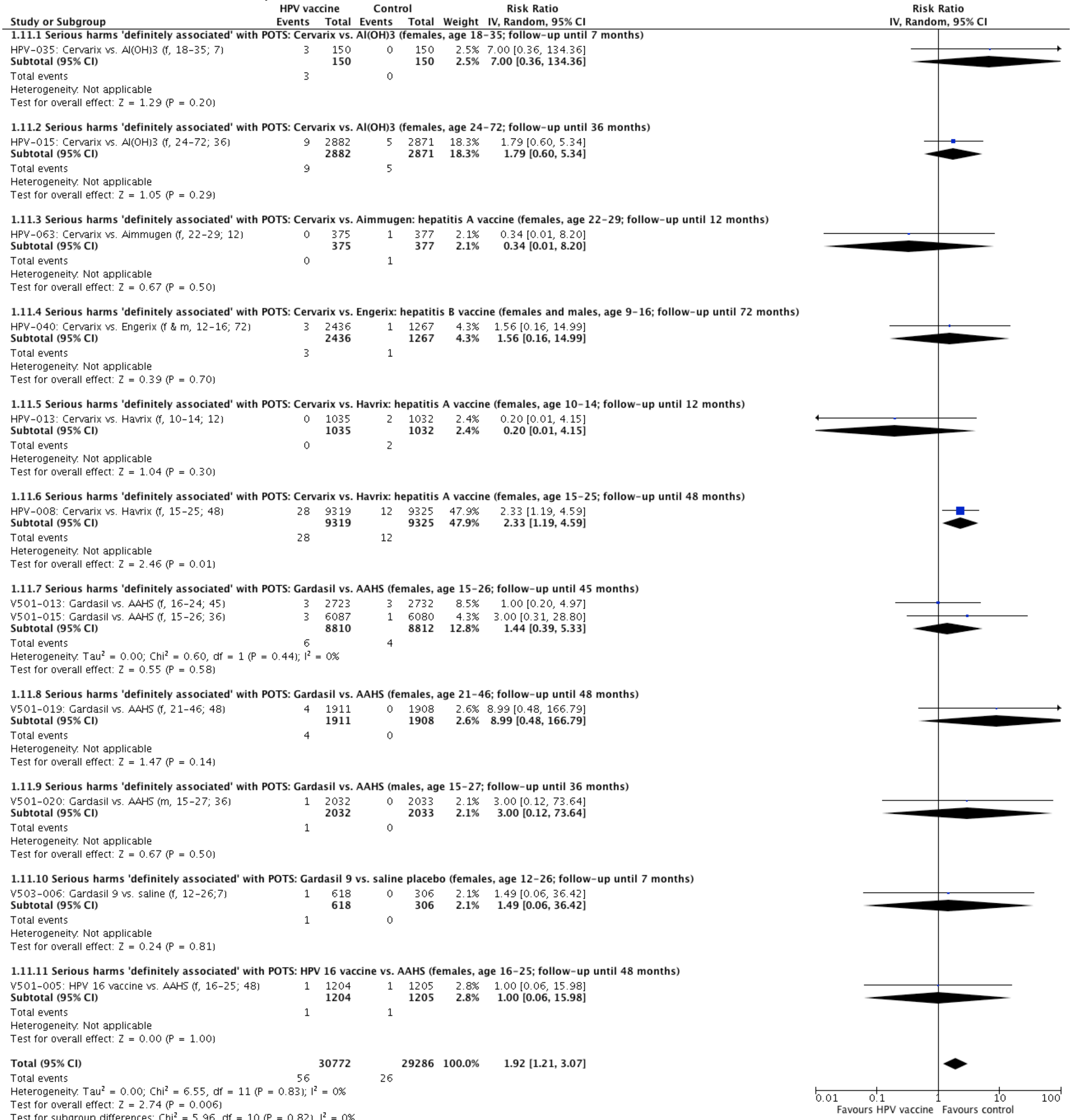

*1.11. Risk ratio for GlaxoSmithKline studies (i.e., HPV-0xx): **1.95 [1.15, 3.32]**; risk ratio for Merck Sharp & Dohme studies (i.e., V50x-xxx): 1.82 [0.68, 4.89]. We asked a physician with clinical expertise in POTS to assess the reported MedDRA preferred terms as 'definitely,' 'probably,' 'probably not' or 'definitely not' associated with POTS. We sent an Excel sheet to the physician with all the reported MedDRA terms. The physician was blinded, as the Excel sheet contained no outcome data. When the physician had assessed all the MedDRA terms, we synthesized the data for those MedDRA terms that the physician judged 'definitely' associated with POTS and compared it to the reported serious harms.

1.12. Serious harms reported within the MedDRA system organ class 'nervous system disorders (10029205)'*: intention to treat analysis

Study or Subgroup	HPV vaccine Events	HPV vaccine Total	Control Events	Control Total	Weight	Risk Ratio IV, Random, 95% CI
**1.12.1 Serious nervous system disorders: Cervarix vs. Al(OH)3 (females, age 18-35; follow-up until 12 months)**						
HPV-003: Cervarix vs. Al(OH)3 (f, 18-30; 12)	1	31	0	30	1.4%	2.91 [0.12, 68.66]
HPV-035: Cervarix vs. Al(OH)3 (f, 18-35; 7)	2	150	0	150	1.5%	5.00 [0.24, 103.28]
**Subtotal (95% CI)**		**181**		**180**	**2.9%**	**3.86 [0.43, 34.36]**
Total events	3		0			
Heterogeneity: Tau² = 0.00; Chi² = 0.06, df = 1 (P = 0.81); I² = 0%						
Test for overall effect: Z = 1.21 (P = 0.23)						
**1.12.2 Serious nervous system disorders: Cervarix vs. Al(OH)3 (females, age 24-72; follow-up until 36 months)**						
HPV-015: Cervarix vs. Al(OH)3 (f, 24-72; 36)	15	2882	11	2871	23.1%	1.36 [0.63, 2.95]
**Subtotal (95% CI)**		**2882**		**2871**	**23.1%**	**1.36 [0.63, 2.95]**
Total events	15		11			
Heterogeneity: Not applicable						
Test for overall effect: Z = 0.77 (P = 0.44)						
**1.12.3 Serious nervous system disorders: Cervarix vs. Aimmugen: hepatitis A vaccine (females, age 22-29; follow-up until 12 months)**						
HPV-063: Cervarix vs. Aimmugen (f, 22-29; 12)	1	375	1	377	1.8%	1.01 [0.06, 16.01]
**Subtotal (95% CI)**		**375**		**377**	**1.8%**	**1.01 [0.06, 16.01]**
Total events	1		1			
Heterogeneity: Not applicable						
Test for overall effect: Z = 0.00 (P = 1.00)						
**1.12.4 Serious nervous system disorders: Cervarix vs. Engerix: hepatitis B vaccine (females and males, age 12-16; follow-up until 72 months)**						
HPV-040: Cervarix vs. Engerix (f & m, 12-16; 72)	2	2436	0	1267	1.5%	2.60 [0.12, 54.15]
**Subtotal (95% CI)**		**2436**		**1267**	**1.5%**	**2.60 [0.12, 54.15]**
Total events	2		0			
Heterogeneity: Not applicable						
Test for overall effect: Z = 0.62 (P = 0.54)						
**1.12.5 Serious nervous system disorders: Cervarix vs. Engerix: hepatitis B vaccine (females, age 26-46; follow-up until 12 months)**						
HPV-069: Cervarix vs. Engerix (f, 26-46; 12)	1	606	0	606	1.4%	3.00 [0.12, 73.50]
**Subtotal (95% CI)**		**606**		**606**	**1.4%**	**3.00 [0.12, 73.50]**
Total events	1		0			
Heterogeneity: Not applicable						
Test for overall effect: Z = 0.67 (P = 0.50)						
**1.12.6 Serious nervous system disorders: Cervarix vs. Havrix: hepatitis A vaccine (females, age 10-14; follow-up until 12 months)**						
HPV-013: Cervarix vs. Havrix (f, 10-14; 12)	0	1035	1	1032	1.4%	0.33 [0.01, 8.15]
**Subtotal (95% CI)**		**1035**		**1032**	**1.4%**	**0.33 [0.01, 8.15]**
Total events	0		1			
Heterogeneity: Not applicable						
Test for overall effect: Z = 0.67 (P = 0.50)						
**1.12.7 Serious nervous system disorders: Cervarix vs. Havrix: hepatitis A vaccine (females, age 15-25; follow-up until 48 months)**						
HPV-008: Cervarix vs. Havrix (f, 15-25; 48)	39	9319	25	9325	55.4%	1.56 [0.95, 2.58]
**Subtotal (95% CI)**		**9319**		**9325**	**55.4%**	**1.56 [0.95, 2.58]**
Total events	39		25			
Heterogeneity: Not applicable						
Test for overall effect: Z = 1.74 (P = 0.08)						
**1.12.8 Serious nervous system disorders: Gardasil vs. AAHS (females, age 15-26; follow-up until 45 months)**						
V501-013: Gardasil vs. AAHS (f, 16-24; 45)	1	2723	5	2732	3.0%	0.20 [0.02, 1.72]
V501-015: Gardasil vs. AAHS (f, 15-26; 36)	4	6087	1	6080	2.9%	4.00 [0.45, 35.74]
**Subtotal (95% CI)**		**8810**		**8812**	**5.9%**	**0.89 [0.05, 16.65]**
Total events	5		6			
Heterogeneity: Tau² = 3.25; Chi² = 3.65, df = 1 (P = 0.06); I² = 73%						
Test for overall effect: Z = 0.08 (P = 0.94)						
**1.12.9 Serious nervous system disorders: Gardasil vs. AAHS (females, age 21-46; follow-up until 48 months)**						
V501-019: Gardasil vs. AAHS (f, 21-46; 48)	3	1911	0	1908	1.6%	6.99 [0.36, 135.21]
**Subtotal (95% CI)**		**1911**		**1908**	**1.6%**	**6.99 [0.36, 135.21]**
Total events	3		0			
Heterogeneity: Not applicable						
Test for overall effect: Z = 1.29 (P = 0.20)						
**1.12.10 Serious nervous system disorders: Gardasil vs. AAHS (males, age 15-27; follow-up until 36 months)**						
V501-020: Gardasil vs. AAHS (m, 15-27; 36)	1	2032	0	2033	1.4%	3.00 [0.12, 73.64]
**Subtotal (95% CI)**		**2032**		**2033**	**1.4%**	**3.00 [0.12, 73.64]**
Total events	1		0			
Heterogeneity: Not applicable						
Test for overall effect: Z = 0.67 (P = 0.50)						
**1.12.11 Serious nervous system disorders: Gardasil 9 vs. saline placebo (females, age 12-26; follow-up until 7 months)**						
V503-006: Gardasil 9 vs. saline (f, 12-26;7)	1	618	1	306	1.8%	0.50 [0.03, 7.89]
**Subtotal (95% CI)**		**618**		**306**	**1.8%**	**0.50 [0.03, 7.89]**
Total events	1		1			
Heterogeneity: Not applicable						
Test for overall effect: Z = 0.50 (P = 0.62)						
**1.12.12 Serious nervous system disorders: HPV 16 vaccine vs. AAHS (females, age 16-25; follow-up until 48 months)**						
V501-005: HPV 16 vaccine vs. AAHS (f, 16-25; 48)	1	1204	1	1205	1.8%	1.00 [0.06, 15.98]
**Subtotal (95% CI)**		**1204**		**1205**	**1.8%**	**1.00 [0.06, 15.98]**
Total events	1		1			
Heterogeneity: Not applicable						
Test for overall effect: Z = 0.00 (P = 1.00)						
**Total (95% CI)**		**31409**		**29922**	**100.0%**	**1.49 [1.02, 2.16]**
Total events	72		46			
Heterogeneity: Tau² = 0.00; Chi² = 8.16, df = 13 (P = 0.83); I² = 0%						
Test for overall effect: Z = 2.08 (P = 0.04)						
Test for subgroup differences: Chi² = 4.07, df = 11 (P = 0.97), I² = 0%						

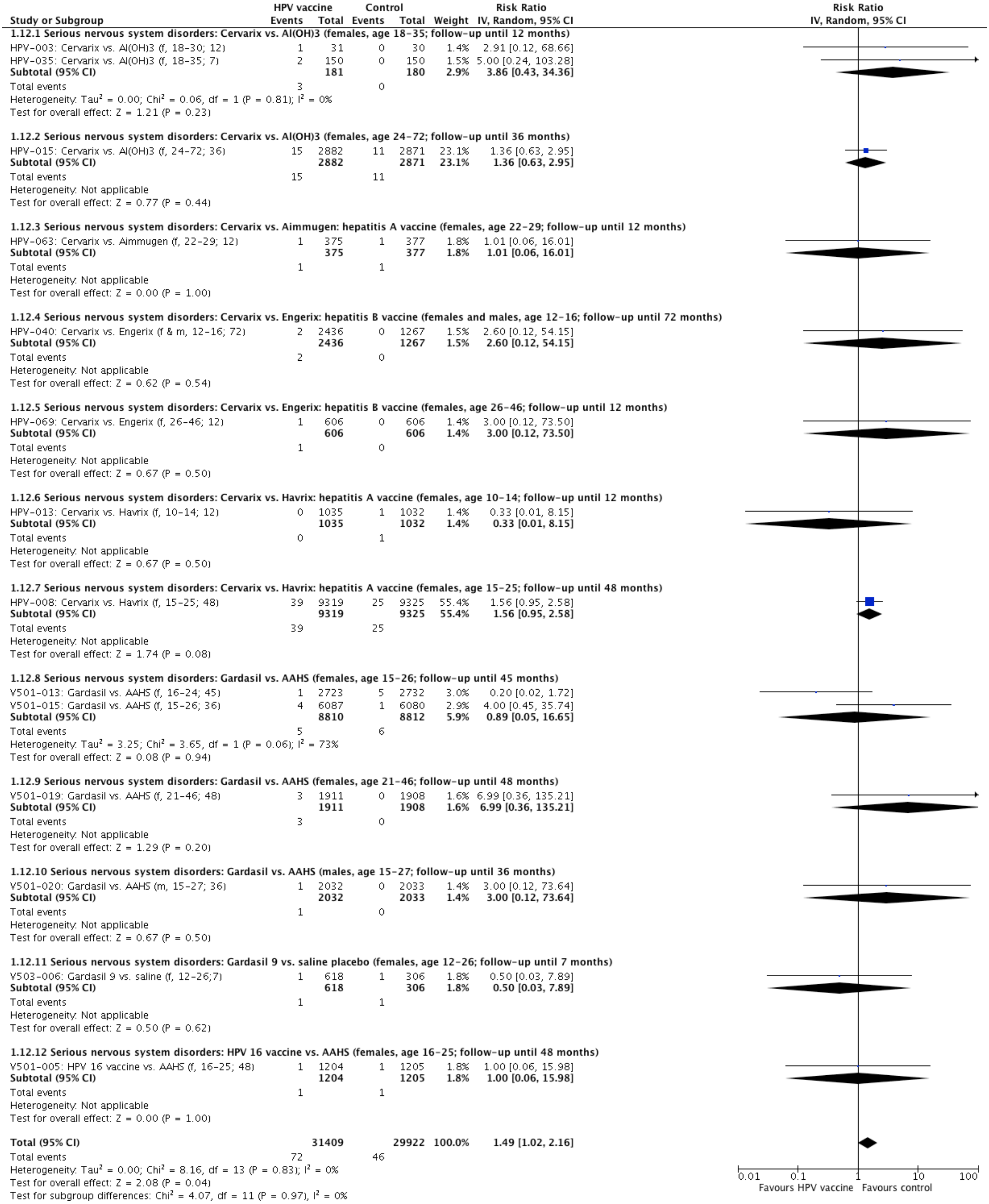

*1.12. Risk ratio for GlaxoSmithKline studies (i.e., HPV-0xx): **1.53 [1.03, 2.28]**; risk ratio for Merck Sharp & Dohme studies (i.e., V50x-xxx): 1.25 [0.39, 3.97].

1.13. New onset diseases ('medically significant conditions' and 'new medical history'*): intention to treat analysis

Study or Subgroup	HPV vaccine Events	HPV vaccine Total	Control Events	Control Total	Weight	Risk Ratio IV, Random, 95% CI
**11.1.1 New onset diseases ('medically significant conditions'): Cervarix vs. Al(OH)3 (females, age 9-17; follow-up until 12 months)**						
HPV-058: Cervarix vs. Al(OH)3 (f, 9-17; 12)	10	374	10	376	0.1%	1.01 [0.42, 2.39]
**Subtotal (95% CI)**		**374**		**376**	**0.1%**	**1.01 [0.42, 2.39]**
Total events	10		10			
Heterogeneity: Not applicable						
Test for overall effect: Z = 0.01 (P = 0.99)						
**11.1.2 New onset diseases ('medically significant conditions'): Cervarix vs. Al(OH)3 (females, age 15-25; follow-up until 7 months)**						
HPV-038: Cervarix vs. Al(OH)3 (f, 15-25; 7)	34	149	10	76	0.2%	1.73 [0.91, 3.32]
**Subtotal (95% CI)**		**149**		**76**	**0.2%**	**1.73 [0.91, 3.32]**
Total events	34		10			
Heterogeneity: Not applicable						
Test for overall effect: Z = 1.66 (P = 0.10)						
**11.1.3 New onset diseases ('medically significant conditions'): Cervarix vs. Al(OH)3 (females, age 18-35; follow-up until 7 months)**						
HPV-031: Cervarix vs. Al(OH)3 (f, 18-35; 7)	13	176	24	178	0.2%	0.55 [0.29, 1.04]
HPV-035: Cervarix vs. Al(OH)3 (f, 18-35; 7)	42	150	24	150	0.4%	1.75 [1.12, 2.74]
**Subtotal (95% CI)**		**326**		**328**	**0.6%**	**1.00 [0.32, 3.13]**
Total events	55		48			
Heterogeneity: Tau² = 0.59; Chi² = 8.46, df = 1 (P = 0.004); I² = 88%						
Test for overall effect: Z = 0.00 (P = 1.00)						
**11.1.4 New onset diseases ('medically significant conditions'): Cervarix vs. Al(OH)3 (females, age 24-72; follow-up until 36 months)**						
HPV-015: Cervarix vs. Al(OH)3 (f, 24-72; 36)	1170	2882	1140	2871	10.4%	1.02 [0.96, 1.09]
**Subtotal (95% CI)**		**2882**		**2871**	**10.4%**	**1.02 [0.96, 1.09]**
Total events	1170		1140			
Heterogeneity: Not applicable						
Test for overall effect: Z = 0.69 (P = 0.49)						
**11.1.5 New onset diseases ('medically significant conditions'): Cervarix vs. Aimmugen: hepatitis A vaccine (females, age 22-29; follow-up until 12 months)**						
HPV-032: Cervarix vs. Aimmugen (f, 19-25; 7)	98	519	115	521	1.3%	0.86 [0.67, 1.09]
**Subtotal (95% CI)**		**519**		**521**	**1.3%**	**0.86 [0.67, 1.09]**
Total events	98		115			
Heterogeneity: Not applicable						
Test for overall effect: Z = 1.27 (P = 0.20)						
**11.1.6 New onset diseases ('medically significant conditions'): Cervarix vs. Engerix: hepatitis B vaccine (females and males, age 9-15; follow-up until 12 months)**						
HPV-030: Cervarix vs. Engerix (f, 9-15; 12)	28	247	22	246	0.3%	1.27 [0.75, 2.15]
HPV-040: Cervarix vs. Engerix (f & m, 12-16; 72)	47	643	76	1047	0.6%	1.01 [0.71, 1.43]
**Subtotal (95% CI)**		**890**		**1293**	**0.9%**	**1.08 [0.81, 1.45]**
Total events	75		98			
Heterogeneity: Tau² = 0.00; Chi² = 0.50, df = 1 (P = 0.48); I² = 0%						
Test for overall effect: Z = 0.52 (P = 0.61)						
**11.1.7 New onset diseases ('medically significant conditions'): Cervarix vs. Engerix: hepatitis B vaccine (females, age 26-46; follow-up until 12 months)**						
HPV-069: Cervarix vs. Engerix (f, 26-46; 12)	5	606	7	606	0.1%	0.71 [0.23, 2.24]
**Subtotal (95% CI)**		**606**		**606**	**0.1%**	**0.71 [0.23, 2.24]**
Total events	5		7			
Heterogeneity: Not applicable						
Test for overall effect: Z = 0.58 (P = 0.56)						
**11.1.8 New onset diseases ('medically significant conditions'): Cervarix vs. Havrix: hepatitis A vaccine (females, age 9-15; follow-up until 12 months)**						
HPV-013: Cervarix vs. Havrix (f, 10-14; 12)	130	1035	160	1032	1.6%	0.81 [0.65, 1.00]
HPV-033: Cervarix vs. Havrix (f, 9-15; 7)	11	160	10	161	0.1%	1.11 [0.48, 2.53]
**Subtotal (95% CI)**		**1195**		**1193**	**1.7%**	**0.83 [0.67, 1.02]**
Total events	141		170			
Heterogeneity: Tau² = 0.00; Chi² = 0.51, df = 1 (P = 0.47); I² = 0%						
Test for overall effect: Z = 1.80 (P = 0.07)						
**11.1.9 New onset diseases ('medically significant conditions'): Cervarix vs. Havrix: hepatitis A vaccine (females, age 15-25; follow-up until 48 months)**						
HPV-008: Cervarix vs. Havrix (f, 15-25; 48)	3298	9319	3378	9325	15.6%	0.98 [0.94, 1.02]
**Subtotal (95% CI)**		**9319**		**9325**	**15.6%**	**0.98 [0.94, 1.02]**
Total events	3298		3378			
Heterogeneity: Not applicable						
Test for overall effect: Z = 1.19 (P = 0.23)						
**11.1.10 New onset diseases ('medically significant conditions'): Cervarix vs. Twinrix: hepatitis A and B vaccine (females, age 8-15; follow-up until 12 months)**						
HPV-029: Cervarix vs. Twinrix (f, 8-15; 12)	23	270	31	271	0.3%	0.74 [0.45, 1.24]
**Subtotal (95% CI)**		**270**		**271**	**0.3%**	**0.74 [0.45, 1.24]**
Total events	23		31			
Heterogeneity: Not applicable						
Test for overall effect: Z = 1.13 (P = 0.26)						
**11.1.11 New onset diseases ('new medical history'): Gardasil vs. carrier solution (females and males, age 9-16; follow-up until 18 months)**						
V501-018: Gardasil vs. carrier solu. (f&m,9-16;18)	520	1184	280	597	5.3%	0.94 [0.84, 1.04]
**Subtotal (95% CI)**		**1184**		**597**	**5.3%**	**0.94 [0.84, 1.04]**
Total events	520		280			
Heterogeneity: Not applicable						
Test for overall effect: Z = 1.20 (P = 0.23)						
**11.1.12 New onset diseases ('new medical history'): Gardasil vs. AAHS (females, age 15-26; follow-up until 45 months)**						
V501-013: Gardasil vs. AAHS (f, 16-24; 45)	2328	2723	2311	2732	19.4%	1.01 [0.99, 1.03]
V501-015: Gardasil vs. AAHS (f, 15-26; 36)	4357	6087	4399	6080	19.4%	0.99 [0.97, 1.01]
**Subtotal (95% CI)**		**8810**		**8812**	**38.8%**	**1.00 [0.98, 1.02]**
Total events	6685		6710			
Heterogeneity: Tau² = 0.00; Chi² = 1.78, df = 1 (P = 0.18); I² = 44%						
Test for overall effect: Z = 0.01 (P = 0.99)						
**11.1.13 New onset diseases ('new medical history'): Gardasil vs. AAHS (females, age 21-46; from 0-7 months)**						
V501-019: Gardasil vs. AAHS (f, 21-46; 48)	756	1911	702	1908	7.8%	1.08 [0.99, 1.17]
**Subtotal (95% CI)**		**1911**		**1908**	**7.8%**	**1.08 [0.99, 1.17]**
Total events	756		702			
Heterogeneity: Not applicable						
Test for overall effect: Z = 1.76 (P = 0.08)						
**11.1.14 New onset diseases ('new medical history'): Gardasil vs. AAHS (females, age 21-46; from 7-48 months)**						
V501-019: Gardasil vs. AAHS (f, 21-46; 48)	958	1911	985	1908		Not estimable
**Subtotal (95% CI)**		**0**		**0**		**Not estimable**
Total events	0		0			
Heterogeneity: Not applicable						
Test for overall effect: Not applicable						
**11.1.15 New onset diseases ('new medical history'): Gardasil vs. AAHS (males, age 15-27; from 0-7 months)**						
V501-020: Gardasil vs. AAHS (m, 15-27; 36)	498	2032	463	2032	5.0%	1.08 [0.96, 1.20]
**Subtotal (95% CI)**		**2032**		**2032**	**5.0%**	**1.08 [0.96, 1.20]**
Total events	498		463			
Heterogeneity: Not applicable						
Test for overall effect: Z = 1.29 (P = 0.20)						
**11.1.16 New onset diseases ('new medical history'): Gardasil vs. AAHS (males, age 15-27; from 7-36 months)**						
V501-020: Gardasil vs. AAHS (m, 15-27; 36)	512	2032	546	2033		Not estimable
**Subtotal (95% CI)**		**0**		**0**		**Not estimable**
Total events	0		0			
Heterogeneity: Not applicable						
Test for overall effect: Not applicable						
**11.1.17 New onset diseases ('new medical history'): Gardasil 9 vs. saline placebo (females, age 12-26; follow-up until 7 months)**						
V503-006: Gardasil 9 vs. saline (f, 12-26;7)	175	618	99	306	1.7%	0.88 [0.71, 1.07]
**Subtotal (95% CI)**		**618**		**306**	**1.7%**	**0.88 [0.71, 1.07]**
Total events	175		99			
Heterogeneity: Not applicable						
Test for overall effect: Z = 1.27 (P = 0.20)						
**11.1.18 New onset diseases ('new medical history'): HPV 16 vaccine vs. AAHS (females, age 16-25; from 0-7 months)**						
V501-005: HPV 16 vaccine vs. AAHS (f, 16-25; 48)	715	1204	753	1205	10.3%	0.95 [0.89, 1.01]
**Subtotal (95% CI)**		**1204**		**1205**	**10.3%**	**0.95 [0.89, 1.01]**
Total events	715		753			
Heterogeneity: Not applicable						
Test for overall effect: Z = 1.56 (P = 0.12)						
**11.1.19 New onset diseases ('new medical history'): HPV 16 vaccine vs. AAHS (females, age 16-25; from 7-48 months)**						
V501-005: HPV 16 vaccine vs. AAHS (f, 16-25; 48)	826	1204	834	1205		Not estimable
**Subtotal (95% CI)**		**0**		**0**		**Not estimable**
Total events	0		0			
Heterogeneity: Not applicable						
Test for overall effect: Not applicable						
**Total (95% CI)**		**32289**		**31720**	**100.0%**	**0.99 [0.97, 1.02]**
Total events	14258		14014			
Heterogeneity: Tau² = 0.00; Chi² = 33.56, df = 19 (P = 0.02); I² = 43%						
Test for overall effect: Z = 0.46 (P = 0.64)						
Test for subgroup differences: Chi² = 21.22, df = 15 (P = 0.13), I² = 29.3%						

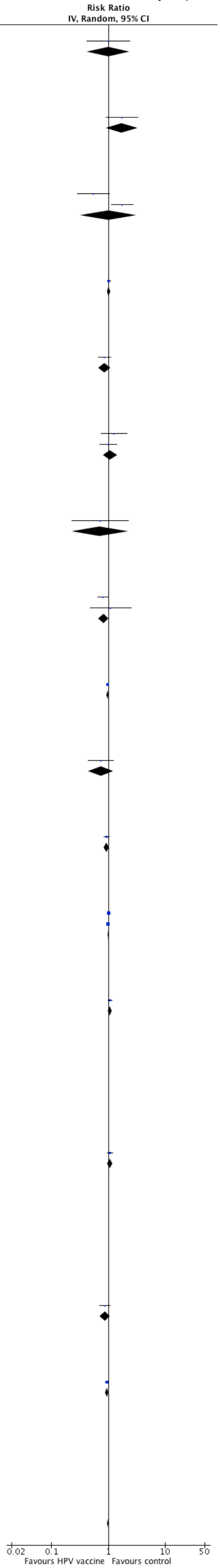

*1.13. Risk ratio for 'medically significant conditions' (GlaxoSmithKline): 0.98 [0.90, 1.06]; risk ratio for 'new medical history' (Merck Sharp & Dohme): 1.00 [0.97, 1.03]; risk ratio for the follow-up periods for the trials V501-005, V501-019 and V501-020 (Merck Sharp & Dohme): 0.98 [0.94, 1.01] (2,296 participants with new medical history in the HPV vaccine group vs. 2,365 participants with new medical history in the control group. The trials V501-005, V501-019 and V501-020 split the reporting of new onset diseases into the vaccination period and the follow-up period. To avoid double counting of participants in the total risk ratio estimate, we only included the new onset diseases reported in the vaccination period for the trials V501-005, V501-019 and V501-020).

1.14. New onset diseases most associated with the HPV vaccines (‘medically significant conditions’*) - ‘back pain’: intention to treat analysis

Study or Subgroup	HPV vaccine Events	HPV vaccine Total	Control Events	Control Total	Weight	Risk Ratio IV, Random, 95% CI
**11.34.1 New onset back pain ('medically significant conditions'): Cervarix vs. Al(OH)3 (females, age 18-35; follow-up until 12 months)**						
HPV-031: Cervarix vs. Al(OH)3 (f, 18-35; 7)	2	176	1	178	0.4%	2.02 [0.19, 22.11]
HPV-035: Cervarix vs. Al(OH)3 (f, 18-35; 7)	2	150	1	150	0.4%	2.00 [0.18, 21.82]
**Subtotal (95% CI)**		**326**		**328**	**0.7%**	**2.01 [0.37, 10.90]**
Total events	4		2			
Heterogeneity: Tau² = 0.00; Chi² = 0.00, df = 1 (P = 0.99); I² = 0%						
Test for overall effect: Z = 0.81 (P = 0.42)						
**11.34.2 New onset back pain ('medically significant conditions'): Cervarix vs. Al(OH)3 (females, age 24-72; follow-up until 36 months)**						
HPV-015: Cervarix vs. Al(OH)3 (f, 24-72; 36)	50	2882	40	2871	12.3%	1.25 [0.82, 1.88]
**Subtotal (95% CI)**		**2882**		**2871**	**12.3%**	**1.25 [0.82, 1.88]**
Total events	50		40			
Heterogeneity: Not applicable						
Test for overall effect: Z = 1.04 (P = 0.30)						
**11.34.3 New onset back pain ('medically significant conditions'): Cervarix vs. Aimmugen: hepatitis A vaccine (females, age 22-29; follow-up until 12 months)**						
HPV-063: Cervarix vs. Aimmugen (f, 22-29; 12)	1	375	1	377	0.3%	1.01 [0.06, 16.01]
**Subtotal (95% CI)**		**375**		**377**	**0.3%**	**1.01 [0.06, 16.01]**
Total events	1		1			
Heterogeneity: Not applicable						
Test for overall effect: Z = 0.00 (P = 1.00)						
**11.34.4 New onset back pain ('medically significant conditions'): Cervarix vs. Havrix: hepatitis A vaccine (females, age 10-14; follow-up until 12 months)**						
HPV-013: Cervarix vs. Havrix (f, 10-14; 12)	3	1035	2	1032	0.7%	1.50 [0.25, 8.93]
**Subtotal (95% CI)**		**1035**		**1032**	**0.7%**	**1.50 [0.25, 8.93]**
Total events	3		2			
Heterogeneity: Not applicable						
Test for overall effect: Z = 0.44 (P = 0.66)						
**11.34.5 New onset back pain ('medically significant conditions'): Cervarix vs. Havrix: hepatitis A vaccine (females, age 15-25; follow-up until 48 months)**						
HPV-008: Cervarix vs. Havrix (f, 15-25; 48)	54	9319	35	9325	11.7%	1.54 [1.01, 2.36]
**Subtotal (95% CI)**		**9319**		**9325**	**11.7%**	**1.54 [1.01, 2.36]**
Total events	54		35			
Heterogeneity: Not applicable						
Test for overall effect: Z = 2.01 (P = 0.04)						
**11.34.6 New onset back pain ('new medical history'): Gardasil vs. carrier solution (females and males, age 9-16; follow-up until 18 months)**						
V501-018: Gardasil vs. carrier solu. (f&m,9-16;18)	13	1184	6	597	2.3%	1.09 [0.42, 2.86]
**Subtotal (95% CI)**		**1184**		**597**	**2.3%**	**1.09 [0.42, 2.86]**
Total events	13		6			
Heterogeneity: Not applicable						
Test for overall effect: Z = 0.18 (P = 0.86)						
**11.34.7 New onset back pain ('new medical history'): Gardasil vs. AAHS (females, age 15-26; follow-up until 45 months)**						
V501-013: Gardasil vs. AAHS (f, 16-24; 45)	59	2723	67	2732	17.5%	0.88 [0.63, 1.25]
V501-015: Gardasil vs. AAHS (f, 15-26; 36)	160	6087	132	6080	40.5%	1.21 [0.96, 1.52]
**Subtotal (95% CI)**		**8810**		**8812**	**58.0%**	**1.06 [0.78, 1.44]**
Total events	219		199			
Heterogeneity: Tau² = 0.03; Chi² = 2.22, df = 1 (P = 0.14); I² = 55%						
Test for overall effect: Z = 0.40 (P = 0.69)						
**11.34.8 New onset back pain ('new medical history'): Gardasil vs. AAHS (females, age 21-46; from 0-7 months)**						
V501-019: Gardasil vs. AAHS (f, 21-46; 48)	19	1911	20	1908	5.4%	0.95 [0.51, 1.77]
**Subtotal (95% CI)**		**1911**		**1908**	**5.4%**	**0.95 [0.51, 1.77]**
Total events	19		20			
Heterogeneity: Not applicable						
Test for overall effect: Z = 0.17 (P = 0.87)						
**11.34.9 New onset back pain ('new medical history'): Gardasil vs. AAHS (females, age 21-46; from 7-48 months)**						
V501-019: Gardasil vs. AAHS (f, 21-46; 48)	26	1911	27	1908	0.0%	0.96 [0.56, 1.64]
**Subtotal (95% CI)**		**0**		**0**		**Not estimable**
Total events	0		0			
Heterogeneity: Not applicable						
Test for overall effect: Not applicable						
**11.34.10 New onset back pain ('new medical history'): Gardasil vs. AAHS (males, age 15-27; from 0-7 months)**						
V501-020: Gardasil vs. AAHS (m, 15-27; 36)	13	2032	15	2032	3.8%	0.87 [0.41, 1.82]
**Subtotal (95% CI)**		**2032**		**2032**	**3.8%**	**0.87 [0.41, 1.82]**
Total events	13		15			
Heterogeneity: Not applicable						
Test for overall effect: Z = 0.38 (P = 0.70)						
**11.34.11 New onset back pain ('new medical history'): Gardasil vs. AAHS (males, age 15-27; from 7-36 months)**						
V501-020: Gardasil vs. AAHS (m, 15-27; 36)	17	2032	25	2033	0.0%	0.68 [0.37, 1.26]
**Subtotal (95% CI)**		**0**		**0**		**Not estimable**
Total events	0		0			
Heterogeneity: Not applicable						
Test for overall effect: Not applicable						
**11.34.12 New onset back pain ('new medical history'): HPV 16 vaccine vs. AAHS (females, age 16-25; from 0-7 months)**						
V501-005: HPV 16 vaccine vs. AAHS (f, 16-25; 48)	17	1204	15	1205	4.4%	1.13 [0.57, 2.26]
**Subtotal (95% CI)**		**1204**		**1205**	**4.4%**	**1.13 [0.57, 2.26]**
Total events	17		15			
Heterogeneity: Not applicable						
Test for overall effect: Z = 0.36 (P = 0.72)						
**11.34.13 New onset back pain ('new medical history'): HPV 16 vaccine vs. AAHS (females, age 16-25; from 7-48 months)**						
V501-005: HPV 16 vaccine vs. AAHS (f, 16-25; 48)	17	1204	19	1205	0.0%	0.90 [0.47, 1.71]
**Subtotal (95% CI)**		**0**		**0**		**Not estimable**
Total events	0		0			
Heterogeneity: Not applicable						
Test for overall effect: Not applicable						
**11.34.14 New onset back pain ('new medical history'): Gardasil 9 vs. saline placebo (females, age 12-26; follow-up until 7 months)**						
V503-006: Gardasil 9 vs. saline (f, 12-26;7)	4	618	1	306	0.4%	1.98 [0.22, 17.64]
**Subtotal (95% CI)**		**618**		**306**	**0.4%**	**1.98 [0.22, 17.64]**
Total events	4		1			
Heterogeneity: Not applicable						
Test for overall effect: Z = 0.61 (P = 0.54)						
**Total (95% CI)**		**29696**		**28793**	**100.0%**	**1.15 [1.00, 1.33]**
Total events	397		336			
Heterogeneity: Tau² = 0.00; Chi² = 6.11, df = 12 (P = 0.91); I² = 0%						
Test for overall effect: Z = 1.94 (P = 0.05)						
Test for subgroup differences: Chi² = 3.90, df = 10 (P = 0.95), I² = 0%						

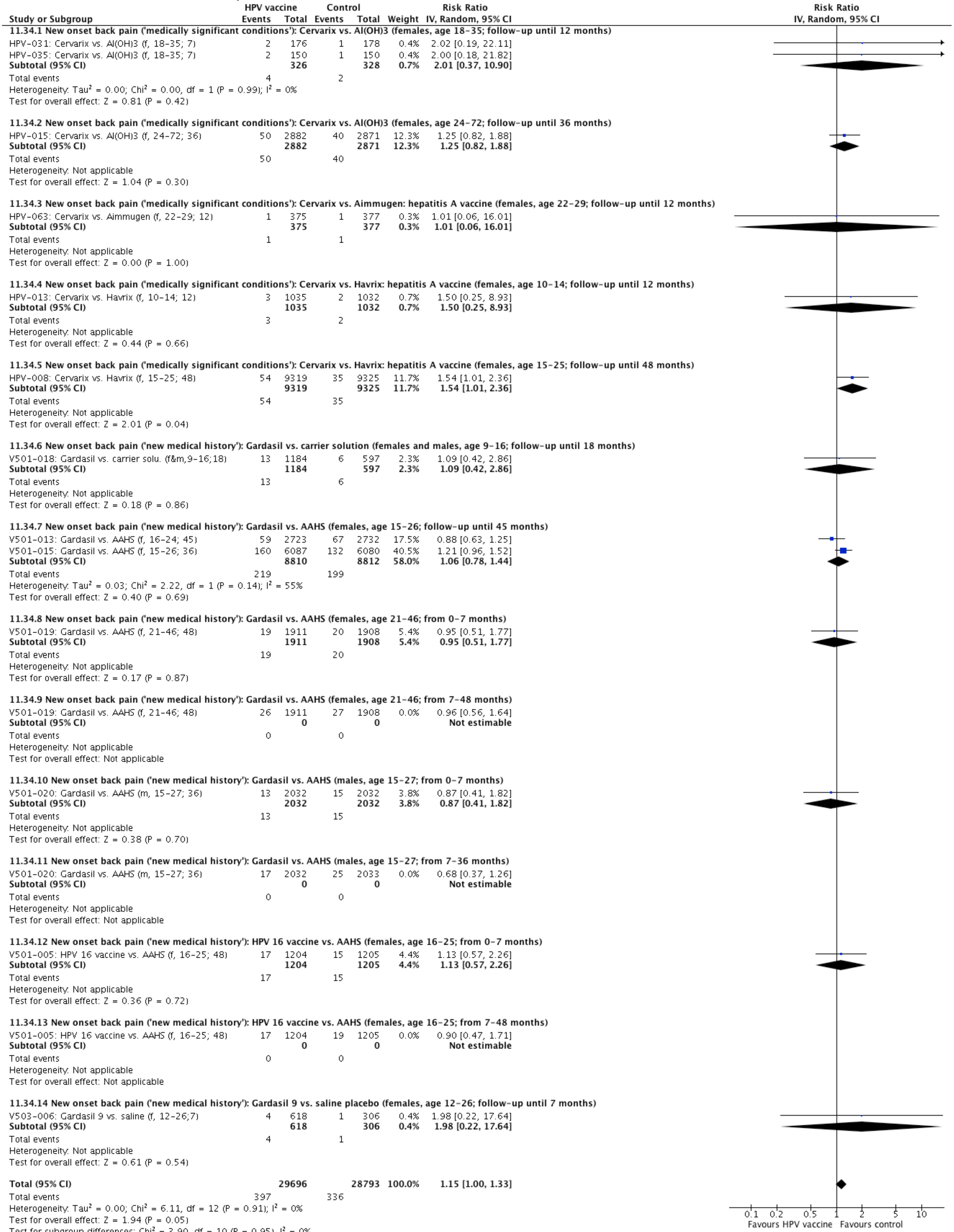

*1.14. Risk ratio for 'medically significant conditions’ (GlaxoSmithKline): **1.40 [1.05, 1.86]**; risk ratio for ‘new medical history’ (Merck Sharp & Dohme): 1.08 [0.91, 1.28]; risk ratio for the follow-up periods for the trials V501-005, V501-019 and V501-020: 0.85 [0.60, 1.19]. The trials V501-005, V501-019 and V501-020 split the reporting of new onset diseases into the vaccination period and the follow-up period. To avoid double counting of participants in the total risk ratio estimate, we only included the new onset diseases reported in the vaccination period for the trials V501-005, V501-019 and V501-020.

1.15. New onset diseases most inversely associated with the HPV vaccines (‘new medical history’*) - ‘vaginal infection’: intention to treat analysis

Study or Subgroup	HPV vaccine Events	HPV vaccine Total	Control Events	Control Total	Weight	Risk Ratio IV, Random, 95% CI
**11.43.1 New onset vaginal infection ('new medical history'): Gardasil vs. carrier solution (females and males, age 9-16; follow-up until 18 months)**						
V501-018: Gardasil vs. carrier solu. (f&m,9-16;18)	3	1184	1	597	0.4%	1.51 [0.16, 14.51]
**Subtotal (95% CI)**		**1184**		**597**	**0.4%**	**1.51 [0.16, 14.51]**
Total events	3		1			
Heterogeneity: Not applicable						
Test for overall effect: Z = 0.36 (P = 0.72)						
**11.43.2 New onset vaginal infection ('new medical history'): Gardasil vs. AAHS (females, age 15-26; follow-up until 45 months)**						
V501-013: Gardasil vs. AAHS (f, 16-24; 45)	134	2723	156	2732	37.1%	0.86 [0.69, 1.08]
V501-015: Gardasil vs. AAHS (f, 15-26; 36)	225	6087	259	6080	61.2%	0.87 [0.73, 1.03]
**Subtotal (95% CI)**		**8810**		**8812**	**98.3%**	**0.87 [0.75, 0.99]**
Total events	359		415			
Heterogeneity: Tau² = 0.00; Chi² = 0.00, df = 1 (P = 0.96); I² = 0%						
Test for overall effect: Z = 2.05 (P = 0.04)						
**11.43.3 New onset vaginal infection ('new medical history'): Gardasil vs. AAHS (females, age 21-46; from 0-7 months)**						
V501-019: Gardasil vs. AAHS (f, 21-46; 48)	6	1911	4	1908	1.2%	1.50 [0.42, 5.30]
**Subtotal (95% CI)**		**1911**		**1908**	**1.2%**	**1.50 [0.42, 5.30]**
Total events	6		4			
Heterogeneity: Not applicable						
Test for overall effect: Z = 0.63 (P = 0.53)						
**11.43.4 New onset vaginal infection ('new medical history'): Gardasil vs. AAHS (females, age 21-46; from 7-48 months)**						
V501-019: Gardasil vs. AAHS (f, 21-46; 48)	8	1911	13	1908	0.0%	0.61 [0.26, 1.48]
**Subtotal (95% CI)**		**0**		**0**		**Not estimable**
Total events	0		0			
Heterogeneity: Not applicable						
Test for overall effect: Not applicable						
**11.43.5 New onset vaginal infection ('new medical history'): Gardasil 9 vs. saline placebo (females, age 12-26; follow-up until 7 months)**						
V503-006: Gardasil 9 vs. saline (f, 12-26;7)	1	618	0	306	0.2%	1.49 [0.06, 36.42]
**Subtotal (95% CI)**		**618**		**306**	**0.2%**	**1.49 [0.06, 36.42]**
Total events	1		0			
Heterogeneity: Not applicable						
Test for overall effect: Z = 0.24 (P = 0.81)						
**Total (95% CI)**		**12523**		**11623**	**100.0%**	**0.87 [0.76, 1.00]**
Total events	369		420			
Heterogeneity: Tau² = 0.00; Chi² = 1.05, df = 4 (P = 0.90); I² = 0%						
Test for overall effect: Z = 1.93 (P = 0.05)						
Test for subgroup differences: Chi² = 1.05, df = 3 (P = 0.79), I² = 0%						

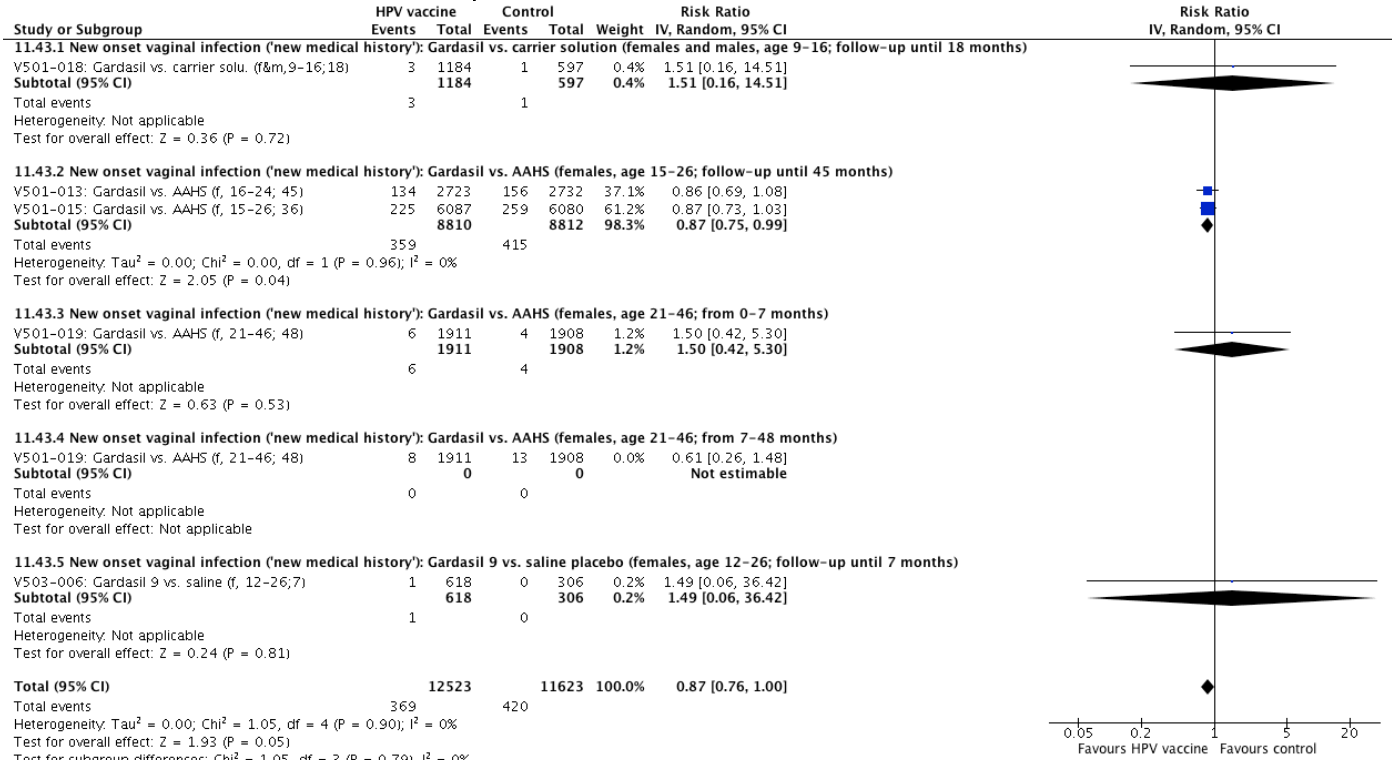

*1.15. Risk ratio for 'medically significant conditions’ (GlaxoSmithKline): not applicable; risk ratio for ‘new medical history’ (Merck Sharp & Dohme): **0.87 [0.76, 1.00]**; risk ratio for the follow-up period for the trial V501-019: 0.61 [0.26, 1.48]. The trial V501-019 split the reporting of new onset diseases into the vaccination period and the follow-up period. To avoid double counting of participants in the total risk ratio estimate, we only included the new onset diseases reported in the vaccination period for the trial V501-019.

1.16. New onset diseases ('medically significant conditions' and 'new medical history'*) reported within the MedDRA system organ class ‘vascular disorders (10047065)': intention to treat analysis

Study or Subgroup	HPV vaccine Events	HPV vaccine Total	Control Events	Control Total	Weight	Risk Ratio IV, Random, 95% CI
**11.27.1 New onset vascular disorders ('medically significant conditions'): Cervarix vs. Al(OH)3 (females, age 24-72; follow-up until 36 months)**						
HPV-015: Cervarix vs. Al(OH)3 (f, 24-72; 36)	65	2882	95	2871	30.4%	0.68 [0.50, 0.93]
**Subtotal (95% CI)**		**2882**		**2871**	**30.4%**	**0.68 [0.50, 0.93]**
Total events	65		95			
Heterogeneity: Not applicable						
Test for overall effect: Z = 2.41 (P = 0.02)						
**11.27.2 New onset vascular disorders ('medically significant conditions'): Cervarix vs. Aimmugen: hepatitis A vaccine (females, age 22-29; follow-up until 12 months)**						
HPV-063: Cervarix vs. Aimmugen (f, 22-29; 12)	1	375	1	377	0.4%	1.01 [0.06, 16.01]
**Subtotal (95% CI)**		**375**		**377**	**0.4%**	**1.01 [0.06, 16.01]**
Total events	1		1			
Heterogeneity: Not applicable						
Test for overall effect: Z = 0.00 (P = 1.00)						
**11.27.3 New onset vascular disorders ('medically significant conditions'): Cervarix vs. Havrix: hepatitis A vaccine (females, age 10-14; follow-up until 12 months)**						
HPV-013: Cervarix vs. Havrix (f, 10-14; 12)	4	1035	2	1032	1.0%	1.99 [0.37, 10.86]
**Subtotal (95% CI)**		**1035**		**1032**	**1.0%**	**1.99 [0.37, 10.86]**
Total events	4		2			
Heterogeneity: Not applicable						
Test for overall effect: Z = 0.80 (P = 0.42)						
**11.27.4 New onset vascular disorders ('medically significant conditions'): Cervarix vs. Havrix: hepatitis A vaccine (females, age 15-25; follow-up until 48 months)**						
HPV-008: Cervarix vs. Havrix (f, 15-25; 48)	41	9319	39	9325	15.4%	1.05 [0.68, 1.63]
**Subtotal (95% CI)**		**9319**		**9325**	**15.4%**	**1.05 [0.68, 1.63]**
Total events	41		39			
Heterogeneity: Not applicable						
Test for overall effect: Z = 0.23 (P = 0.82)						
**11.27.5 New onset vascular disorders ('medically significant conditions'): Cervarix vs. Twinrix: hepatitis A and B vaccine (females, age 8-15; follow-up until 12 months)**						
HPV-029: Cervarix vs. Twinrix (f, 8-15; 12)	0	270	1	271	0.3%	0.33 [0.01, 8.18]
**Subtotal (95% CI)**		**270**		**271**	**0.3%**	**0.33 [0.01, 8.18]**
Total events	0		1			
Heterogeneity: Not applicable						
Test for overall effect: Z = 0.67 (P = 0.50)						
**11.27.6 New onset vascular disorders ('new medical history'): Gardasil vs. carrier solution (females and males, age 9-16; follow-up until 18 months)**						
V501-018: Gardasil vs. carrier solu. (f&m,9-16;18)	1	1184	2	597	0.5%	0.25 [0.02, 2.77]
**Subtotal (95% CI)**		**1184**		**597**	**0.5%**	**0.25 [0.02, 2.77]**
Total events	1		2			
Heterogeneity: Not applicable						
Test for overall effect: Z = 1.13 (P = 0.26)						
**11.27.7 New onset vascular disorders ('new medical history'): Gardasil vs. AAHS (females, age 15-26; follow-up until 45 months)**						
V501-013: Gardasil vs. AAHS (f, 16-24; 45)	36	2723	34	2732	13.6%	1.06 [0.67, 1.69]
V501-015: Gardasil vs. AAHS (f, 15-26; 36)	65	6087	85	6080	28.6%	0.76 [0.55, 1.05]
**Subtotal (95% CI)**		**8810**		**8812**	**42.2%**	**0.86 [0.63, 1.18]**
Total events	101		119			
Heterogeneity: $Tau^2 = 0.01; Chi^2 = 1.31, df = 1 (P = 0.25); I^2 = 23\%$						
Test for overall effect: Z = 0.94 (P = 0.35)						
**11.27.8 New onset vascular disorders ('new medical history'): Gardasil vs. AAHS (females, age 21-46; from 0-7 months)**						
V501-019: Gardasil vs. AAHS (f, 21-46; 48)	9	1911	22	1908	4.9%	0.41 [0.19, 0.88]
**Subtotal (95% CI)**		**1911**		**1908**	**4.9%**	**0.41 [0.19, 0.88]**
Total events	9		22			
Heterogeneity: Not applicable						
Test for overall effect: Z = 2.27 (P = 0.02)						
**11.27.9 New onset vascular disorders ('new medical history'): Gardasil vs. AAHS (females, age 21-46; from 7-48 months)**						
V501-019: Gardasil vs. AAHS (f, 21-46; 48)	45	1911	39	1908	0.0%	1.15 [0.75, 1.76]
**Subtotal (95% CI)**		**0**		**0**		**Not estimable**
Total events	0		0			
Heterogeneity: Not applicable						
Test for overall effect: Not applicable						
**11.27.10 New onset vascular disorders ('new medical history'): Gardasil vs. AAHS (males, age 15-27; from 0-7 months)**						
V501-020: Gardasil vs. AAHS (m, 15-27; 36)	2	2032	3	2032	0.9%	0.67 [0.11, 3.99]
**Subtotal (95% CI)**		**2032**		**2032**	**0.9%**	**0.67 [0.11, 3.99]**
Total events	2		3			
Heterogeneity: Not applicable						
Test for overall effect: Z = 0.44 (P = 0.66)						
**11.27.11 New onset vascular disorders ('new medical history'): Gardasil vs. AAHS (males, age 15-27; from 7-36 months)**						
V501-020: Gardasil vs. AAHS (m, 15-27; 36)	7	2032	12	2033	0.0%	0.58 [0.23, 1.48]
**Subtotal (95% CI)**		**0**		**0**		**Not estimable**
Total events	0		0			
Heterogeneity: Not applicable						
Test for overall effect: Not applicable						
**11.27.12 New onset vascular disorders ('new medical history'): HPV 16 vaccine vs. AAHS (females, age 16-25; from 7-48 months)**						
V501-005: HPV 16 vaccine vs. AAHS (f, 16-25; 48)	10	1204	10	1205	3.9%	1.00 [0.42, 2.40]
**Subtotal (95% CI)**		**1204**		**1205**	**3.9%**	**1.00 [0.42, 2.40]**
Total events	10		10			
Heterogeneity: Not applicable						
Test for overall effect: Z = 0.00 (P = 1.00)						
**Total (95% CI)**		**29022**		**28430**	**100.0%**	**0.80 [0.67, 0.94]**
Total events	234		294			
Heterogeneity: $Tau^2 = 0.00; Chi^2 = 9.54, df = 10 (P = 0.48); I^2 = 0\%$						
Test for overall effect: Z = 2.61 (P = 0.009)						
Test for subgroup differences: $Chi^2 = 8.24, df = 9 (P = 0.51), I^2 = 0\%$						

Risk Ratio IV, Random, 95% CI

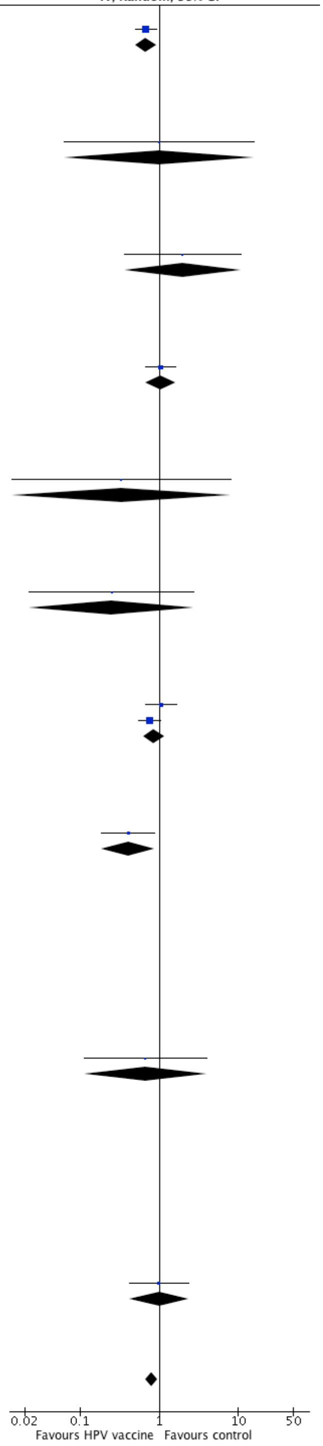

*1.16. Risk ratio for 'medically significant conditions’ (GlaxoSmithKline): 0.80 [0.63, 1.03]; risk ratio for ‘new medical history’ (Merck Sharp & Dohme): 0.78 [0.60, 1.03]; risk ratio for the follow-up periods for the trials V501-019 and V501-020: 0.93 [0.51, 1.73]. The trials V501-019 and V501-020 split the reporting of new onset diseases into the vaccination period and the follow-up period. To avoid double counting of participants in the total risk ratio estimate, we only included the new onset diseases reported in the vaccination period for the trials V501-019 and V501-020.

1.17. General harms ('solicited and unsolicited' and 'systemic adverse events'*): intention to treat analysis

Study or Subgroup	HPV vaccine Events	HPV vaccine Total	Control Events	Control Total	Weight	Risk Ratio IV, Random, 95% CI
**12.1.1 General harms ('solicited' and 'unsolicited'): Cervarix vs. Al(OH)3 (females, age 9-17; follow-up until 12 months)**						
HPV-058: Cervarix vs. Al(OH)3 (f, 9-17; 12)	294	374	275	376	5.3%	1.07 [0.99, 1.17]
**Subtotal (95% CI)**		**374**		**376**	**5.3%**	**1.07 [0.99, 1.17]**
Total events	294		275			
Heterogeneity: Not applicable						
Test for overall effect: Z = 1.75 (P = 0.08)						
**12.1.2 General harms ('solicited' and 'unsolicited'): Cervarix vs. Al(OH)3 (females, age 15-26; follow-up until 27 months)**						
HPV-001: Cervarix vs. Al(OH)3 (f, 15-26; 27)	458	560	462	553	6.3%	0.98 [0.93, 1.03]
HPV-038: Cervarix vs. Al(OH)3 (f, 15-25; 7)	136	149	55	76	3.2%	1.26 [1.09, 1.46]
**Subtotal (95% CI)**		**709**		**629**	**9.6%**	**1.10 [0.86, 1.41]**
Total events	594		517			
Heterogeneity: Tau² = 0.03; Chi² = 10.00, df = 1 (P = 0.002); I² = 90%						
Test for overall effect: Z = 0.76 (P = 0.45)						
**12.1.3 General harms ('solicited' and 'unsolicited'): Cervarix vs. Al(OH)3 (females, age 18-35; follow-up until 12 months)**						
HPV-003: Cervarix vs. Al(OH)3 (f, 18-30; 12)	28	31	29	30	3.6%	0.93 [0.82, 1.07]
HPV-031: Cervarix vs. Al(OH)3 (f, 18-35; 7)	25	176	20	178	0.4%	1.26 [0.73, 2.19]
HPV-035: Cervarix vs. Al(OH)3 (f, 18-35; 7)	136	150	114	150	4.5%	1.19 [1.08, 1.32]
**Subtotal (95% CI)**		**357**		**358**	**8.5%**	**1.08 [0.88, 1.33]**
Total events	189		163			
Heterogeneity: Tau² = 0.02; Chi² = 8.35, df = 2 (P = 0.02); I² = 76%						
Test for overall effect: Z = 0.74 (P = 0.46)						
**12.1.4 General harms ('solicited' and 'unsolicited'): Cervarix vs. Al(OH)3 (females, age 24-72; follow-up until 36 months)**						
HPV-015: Cervarix vs. Al(OH)3 (f, 24-72; 36)	2111	2882	1970	2871	7.0%	1.07 [1.03, 1.10]
**Subtotal (95% CI)**		**2882**		**2871**	**7.0%**	**1.07 [1.03, 1.10]**
Total events	2111		1970			
Heterogeneity: Not applicable						
Test for overall effect: Z = 3.86 (P = 0.0001)						
**12.1.5 General harms ('solicited' and 'unsolicited'): Cervarix vs. Engerix: hepatitis B vaccine (females and males, age 12-16; follow-up until 72 months)**						
HPV-030: Cervarix vs. Engerix (f, 9-15; 12)	202	247	195	246	5.1%	1.03 [0.95, 1.13]
HPV-040: Cervarix vs. Engerix (f & m, 12-16; 72)	484	604	676	1028	6.1%	1.22 [1.15, 1.29]
**Subtotal (95% CI)**		**851**		**1274**	**11.3%**	**1.12 [0.96, 1.32]**
Total events	686		871			
Heterogeneity: Tau² = 0.01; Chi² = 9.62, df = 1 (P = 0.002); I² = 90%						
Test for overall effect: Z = 1.41 (P = 0.16)						
**12.1.6 General harms ('solicited' and 'unsolicited'): Cervarix vs. Engerix: hepatitis B vaccine (females, age 26-46; follow-up until 12 months)**						
HPV-069: Cervarix vs. Engerix (f, 26-46; 12)	202	606	148	606	2.5%	1.36 [1.14, 1.63]
**Subtotal (95% CI)**		**606**		**606**	**2.5%**	**1.36 [1.14, 1.63]**
Total events	202		148			
Heterogeneity: Not applicable						
Test for overall effect: Z = 3.39 (P = 0.0007)						
**12.1.7 General harms ('solicited' and 'unsolicited'): Cervarix vs. Havrix: hepatitis A vaccine (females, age 9-15; follow-up until 12 months)**						
HPV-013: Cervarix vs. Havrix (f, 10-14; 12)	577	1035	468	1032	5.1%	1.23 [1.13, 1.34]
HPV-033: Cervarix vs. Havrix (f, 9-15; 7)	103	160	96	161	2.7%	1.08 [0.91, 1.28]
**Subtotal (95% CI)**		**1195**		**1193**	**7.8%**	**1.18 [1.04, 1.33]**
Total events	680		564			
Heterogeneity: Tau² = 0.00; Chi² = 1.76, df = 1 (P = 0.19); I² = 43%						
Test for overall effect: Z = 2.68 (P = 0.007)						
**12.1.8 General harms ('solicited' and 'unsolicited'): Cervarix vs. Havrix: hepatitis A vaccine (females, age 15-25; follow-up until 48 months)**						
HPV-008: Cervarix vs. Havrix (f, 15-25; 48)	2626	9319	2531	9325	6.6%	1.04 [0.99, 1.09]
**Subtotal (95% CI)**		**9319**		**9325**	**6.6%**	**1.04 [0.99, 1.09]**
Total events	2626		2531			
Heterogeneity: Not applicable						
Test for overall effect: Z = 1.58 (P = 0.11)						
**12.1.9 General harms ('solicited' and 'unsolicited'): Cervarix vs. Twinrix: hepatitis A and B vaccine (females, age 8-15; follow-up until 12 months)**						
HPV-029: Cervarix vs. Twinrix (f, 8-15; 12)	217	270	191	271	4.8%	1.14 [1.03, 1.26]
**Subtotal (95% CI)**		**270**		**271**	**4.8%**	**1.14 [1.03, 1.26]**
Total events	217		191			
Heterogeneity: Not applicable						
Test for overall effect: Z = 2.65 (P = 0.008)						
**12.1.10 General harms ('systemic adverse events'): Gardasil vs. carrier solution (females and males, age 9-16; follow-up until 18 months)**						
V501-018: Gardasil vs. carrier solu. (f&m,9-16;18)	541	1184	260	597	4.3%	1.05 [0.94, 1.17]
**Subtotal (95% CI)**		**1184**		**597**	**4.3%**	**1.05 [0.94, 1.17]**
Total events	541		260			
Heterogeneity: Not applicable						
Test for overall effect: Z = 0.85 (P = 0.39)						
**12.1.11 General harms ('systemic adverse events'): Gardasil vs. AAHS (females, age 15-26; follow-up until 45 months)**						
V501-013: Gardasil vs. AAHS (f, 16-24; 45)	1746	2723	1701	2732	6.8%	1.03 [0.99, 1.07]
V501-015: Gardasil vs. AAHS (f, 15-26; 36)	448	6087	453	6080	3.8%	0.99 [0.87, 1.12]
**Subtotal (95% CI)**		**8810**		**8812**	**10.6%**	**1.03 [0.99, 1.07]**
Total events	2194		2154			
Heterogeneity: Tau² = 0.00; Chi² = 0.38, df = 1 (P = 0.54); I² = 0%						
Test for overall effect: Z = 1.30 (P = 0.20)						
**12.1.12 General harms ('systemic adverse events'): Gardasil vs. AAHS (females, age 21-46; follow-up until 48 months)**						
V501-019: Gardasil vs. AAHS (f, 21-46; 48)	1121	1911	1135	1908	6.4%	0.99 [0.94, 1.04]
**Subtotal (95% CI)**		**1911**		**1908**	**6.4%**	**0.99 [0.94, 1.04]**
Total events	1121		1135			
Heterogeneity: Not applicable						
Test for overall effect: Z = 0.52 (P = 0.60)						
**12.1.13 General harms ('systemic adverse events'): Gardasil vs. AAHS (males, age 15-27; follow-up until 36 months)**						
V501-020: Gardasil vs. AAHS (m, 15-27; 36)	616	2032	613	2033	4.9%	1.01 [0.92, 1.10]
**Subtotal (95% CI)**		**2032**		**2033**	**4.9%**	**1.01 [0.92, 1.10]**
Total events	616		613			
Heterogeneity: Not applicable						
Test for overall effect: Z = 0.11 (P = 0.91)						
**12.1.14 General harms ('systemic adverse events'): Gardasil 9 vs. saline placebo (females, age 12-26; follow-up until 7 months)**						
V503-006: Gardasil 9 vs. saline (f, 12-26;7)	374	618	177	306	4.2%	1.05 [0.93, 1.17]
**Subtotal (95% CI)**		**618**		**306**	**4.2%**	**1.05 [0.93, 1.17]**
Total events	374		177			
Heterogeneity: Not applicable						
Test for overall effect: Z = 0.77 (P = 0.44)						
**12.1.15 General harms ('systemic adverse events'): HPV 16 vaccine vs. AAHS (females, age 16-25; follow-up until 48 months)**						
V501-005: HPV 16 vaccine vs. AAHS (f, 16-25; 48)	803	1204	825	1205	6.3%	0.97 [0.92, 1.03]
**Subtotal (95% CI)**		**1204**		**1205**	**6.3%**	**0.97 [0.92, 1.03]**
Total events	803		825			
Heterogeneity: Not applicable						
Test for overall effect: Z = 0.93 (P = 0.35)						
**Total (95% CI)**		**32322**		**31764**	**100.0%**	**1.07 [1.03, 1.11]**
Total events	13248		12394			
Heterogeneity: Tau² = 0.00; Chi² = 86.35, df = 20 (P < 0.00001); I² = 77%						
Test for overall effect: Z = 3.74 (P = 0.0002)						
Test for subgroup differences: Chi² = 30.88, df = 14 (P = 0.006), I² = 54.7%						

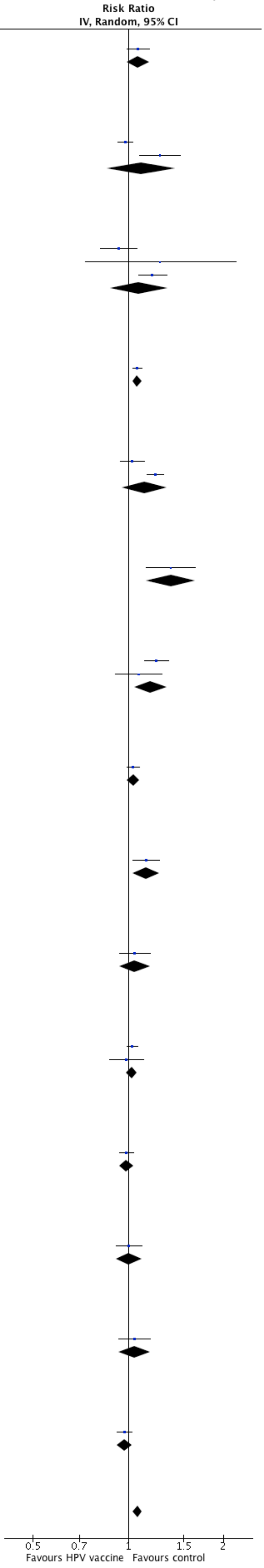

*1.17. Risk ratio for 'solicited and solicited' (GlaxoSmithKline): **1.11 [1.06, 1.16]**; risk ratio for 'systemic adverse events' (Merck Sharp & Dohme): 1.01 [0.98, 1.03]. The total numbers of participants with general harms in GlaxoSmithKline studies were reported as 'solicited [SGAE] and unsolicited [UGAE]' combined.

1.18. General harms most associated with the HPV vaccines ('solicited' and 'unsolicited') - 'fatigue': intention to treat analysis

Study or Subgroup	HPV vaccine Events	HPV vaccine Total	Control Events	Control Total	Weight	Risk Ratio IV, Random, 95% CI
**12.28.1 General fatigue ('solicited'): Cervarix vs. Al(OH)3 (females, age 9-17; follow-up until 12 months)**						
HPV-058: Cervarix vs. Al(OH)3 (f, 9-17; 12)	137	374	126	376	4.3%	1.09 [0.90, 1.33]
**Subtotal (95% CI)**		**374**		**376**	**4.3%**	**1.09 [0.90, 1.33]**
Total events	137		126			
Heterogeneity: Not applicable						
Test for overall effect: Z = 0.89 (P = 0.37)						
**12.28.2 General fatigue ('solicited'): Cervarix vs. Al(OH)3 (females, age 15-26; follow-up until 27 months)**						
HPV-001: Cervarix vs. Al(OH)3 (f, 15-26; 27)	308	560	289	553	10.5%	1.05 [0.94, 1.17]
HPV-038: Cervarix vs. Al(OH)3 (f, 15-25; 7)	103	149	39	76	2.9%	1.35 [1.06, 1.72]
**Subtotal (95% CI)**		**709**		**629**	**13.4%**	**1.16 [0.92, 1.47]**
Total events	411		328			
Heterogeneity: $Tau^2 = 0.02$; $Chi^2 = 3.28$, df = 1 (P = 0.07); $I^2 = 69\%$						
Test for overall effect: Z = 1.24 (P = 0.22)						
**12.28.3 General fatigue ('solicited'): Cervarix vs. Al(OH)3 (females, age 18-35; follow-up until 12 months)**						
HPV-003: Cervarix vs. Al(OH)3 (f, 18-30; 12)	20	31	20	30	1.4%	0.97 [0.67, 1.39]
HPV-031: Cervarix vs. Al(OH)3 (f, 18-35; 7)	84	176	83	178	3.5%	1.02 [0.82, 1.28]
HPV-035: Cervarix vs. Al(OH)3 (f, 18-35; 7)	94	300	75	300	2.6%	1.25 [0.97, 1.62]
**Subtotal (95% CI)**		**507**		**508**	**7.5%**	**1.09 [0.93, 1.27]**
Total events	198		178			
Heterogeneity: $Tau^2 = 0.00$; $Chi^2 = 1.85$, df = 2 (P = 0.40); $I^2 = 0\%$						
Test for overall effect: Z = 1.08 (P = 0.28)						
**12.28.4 General fatigue ('solicited'): Cervarix vs. Al(OH)3 (females, age 24-72; follow-up until 36 months)**						
HPV-015: Cervarix vs. Al(OH)3 (f, 24-72; 36)	1116	2882	916	2871	17.1%	1.21 [1.13, 1.30]
**Subtotal (95% CI)**		**2882**		**2871**	**17.1%**	**1.21 [1.13, 1.30]**
Total events	1116		916			
Heterogeneity: Not applicable						
Test for overall effect: Z = 5.39 (P < 0.00001)						
**12.28.5 General fatigue ('solicited'): Cervarix vs. Engerix: hepatitis B vaccine (females and males, age 9-16; follow-up until 72 months)**						
HPV-030: Cervarix vs. Engerix (f, 9-15; 12)	107	247	104	246	4.0%	1.02 [0.84, 1.26]
HPV-040: Cervarix vs. Engerix (f & m, 12-16; 72)	291	604	411	1028	10.2%	1.21 [1.08, 1.35]
**Subtotal (95% CI)**		**851**		**1274**	**14.2%**	**1.14 [0.98, 1.32]**
Total events	398		515			
Heterogeneity: $Tau^2 = 0.01$; $Chi^2 = 1.87$, df = 1 (P = 0.17); $I^2 = 46\%$						
Test for overall effect: Z = 1.66 (P = 0.10)						
**12.28.6 General fatigue ('solicited'): Cervarix vs. Engerix: hepatitis B vaccine (females, age 26-46; follow-up until 12 months)**						
HPV-069: Cervarix vs. Engerix (f, 26-46; 12)	100	606	68	606	2.2%	1.47 [1.10, 1.96]
**Subtotal (95% CI)**		**606**		**606**	**2.2%**	**1.47 [1.10, 1.96]**
Total events	100		68			
Heterogeneity: Not applicable						
Test for overall effect: Z = 2.64 (P = 0.008)						
**12.28.7 General fatigue ('solicited'): Cervarix vs. Havrix: hepatitis A vaccine (females, age 9-15; follow-up until 12 months)**						
HPV-013: Cervarix vs. Havrix (f, 10-14; 12)	499	1035	434	1032	12.5%	1.15 [1.04, 1.26]
HPV-033: Cervarix vs. Havrix (f, 9-15; 7)	59	160	52	161	2.0%	1.14 [0.84, 1.54]
**Subtotal (95% CI)**		**1195**		**1193**	**14.4%**	**1.15 [1.05, 1.26]**
Total events	558		486			
Heterogeneity: $Tau^2 = 0.00$; $Chi^2 = 0.00$, df = 1 (P = 0.98); $I^2 = 0\%$						
Test for overall effect: Z = 2.93 (P = 0.003)						
**12.28.8 General fatigue ('solicited'): Cervarix vs. Havrix: hepatitis A vaccine (females, age 15-25; follow-up until 48 months)**						
HPV-008: Cervarix vs. Havrix (f, 15-25; 48)	1771	9319	1652	9325	19.4%	1.07 [1.01, 1.14]
**Subtotal (95% CI)**		**9319**		**9325**	**19.4%**	**1.07 [1.01, 1.14]**
Total events	1771		1652			
Heterogeneity: Not applicable						
Test for overall effect: Z = 2.27 (P = 0.02)						
**12.28.9 General fatigue ('solicited'): Cervarix vs. Twinrix: hepatitis A and B vaccine (females, age 8-15; follow-up until 12 months)**						
HPV-029: Cervarix vs. Twinrix (f, 8-15; 12)	133	270	119	271	4.9%	1.12 [0.94, 1.34]
**Subtotal (95% CI)**		**270**		**271**	**4.9%**	**1.12 [0.94, 1.34]**
Total events	133		119			
Heterogeneity: Not applicable						
Test for overall effect: Z = 1.24 (P = 0.21)						
**12.28.10 General fatigue ('unsolicited'): Cervarix vs. Engerix: hepatitis B vaccine (females and males, age 9-16; follow-up until 72 months)**						
HPV-030: Cervarix vs. Engerix (f, 9-15; 12)	2	247	1	246	0.0%	1.99 [0.18, 21.82]
HPV-040: Cervarix vs. Engerix (f & m, 12-16; 72)	0	1908	2	3128	0.0%	0.33 [0.02, 6.82]
**Subtotal (95% CI)**		**0**		**0**		**Not estimable**
Total events	0		0			
Heterogeneity: Not applicable						
Test for overall effect: Not applicable						
**12.28.11 General fatigue ('systemic adverse events'): Gardasil vs. carrier solution (females and males, age 9-16; follow-up until 18 months)**						
V501-018: Gardasil vs. carrier solu. (f&m,9-16;18)	18	1184	7	597	0.3%	1.30 [0.54, 3.09]
**Subtotal (95% CI)**		**1184**		**597**	**0.3%**	**1.30 [0.54, 3.09]**
Total events	18		7			
Heterogeneity: Not applicable						
Test for overall effect: Z = 0.59 (P = 0.56)						
**12.28.12 General fatigue ('systemic adverse events'): Gardasil vs. AAHS (males, age 15-27; follow-up until 36 months)**						
V501-020: Gardasil vs. AAHS (m, 15-27; 36)	13	2032	19	2033	0.4%	0.68 [0.34, 1.38]
**Subtotal (95% CI)**		**2032**		**2033**	**0.4%**	**0.68 [0.34, 1.38]**
Total events	13		19			
Heterogeneity: Not applicable						
Test for overall effect: Z = 1.06 (P = 0.29)						
**12.28.13 General fatigue ('systemic adverse events'): Gardasil 9 vs. saline placebo (females, age 12-26; follow-up until 7 months)**						
V503-006: Gardasil 9 vs. saline (f, 12-26;7)	21	618	11	306	0.4%	0.95 [0.46, 1.94]
**Subtotal (95% CI)**		**618**		**306**	**0.4%**	**0.95 [0.46, 1.94]**
Total events	21		11			
Heterogeneity: Not applicable						
Test for overall effect: Z = 0.15 (P = 0.88)						
**12.28.14 General fatigue ('systemic adverse events'): HPV 16 vaccine vs. AAHS (females, age 16-25; follow-up until 48 months)**						
V501-005: HPV 16 vaccine vs. AAHS (f, 16-25; 48)	59	1204	64	1205	1.5%	0.92 [0.65, 1.30]
**Subtotal (95% CI)**		**1204**		**1205**	**1.5%**	**0.92 [0.65, 1.30]**
Total events	59		64			
Heterogeneity: Not applicable						
Test for overall effect: Z = 0.46 (P = 0.65)						
**Total (95% CI)**		**21751**		**21194**	**100.0%**	**1.13 [1.08, 1.18]**
Total events	4933		4489			
Heterogeneity: $Tau^2 = 0.00$; $Chi^2 = 21.72$, df = 17 (P = 0.20); $I^2 = 22\%$						
Test for overall effect: Z = 5.50 (P < 0.00001)						
Test for subgroup differences: $Chi^2 = 14.15$, df = 12 (P = 0.29), $I^2 = 15.2\%$						

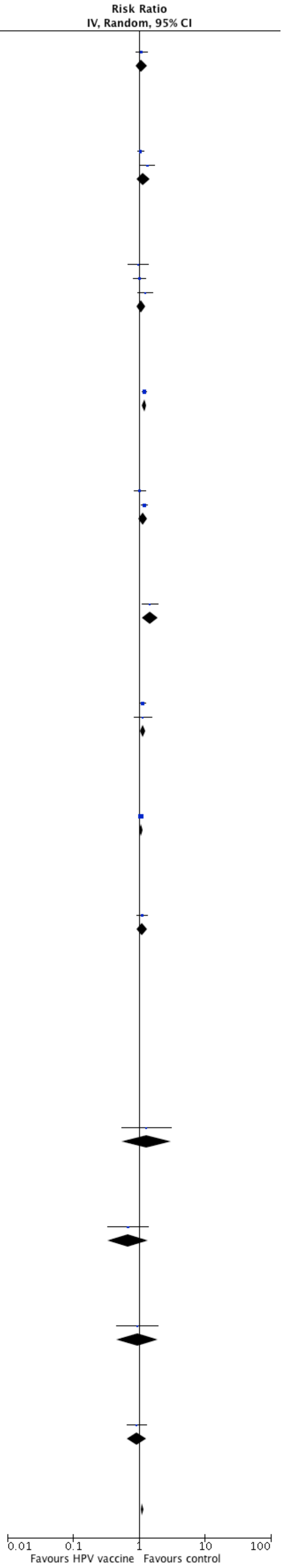

*1.18. Risk ratio for 'solicited' (GlaxoSmithKline): **1.14 [1.09, 1.19]**; risk ratio for 'unsolicited' (GlaxoSmithKline): 1.00 [0.15, 6.53]; risk ratio for 'systemic adverse events' (Merck Sharp & Dohme): 0.92 [0.70, 1.20]. To avoid double counting of participants in the total risk ratio estimate, we excluded the 'unsolicited' adverse events from total risk ratio estimate for studies that reported 'solicited' adverse events.

1.19. General harms most associated with the HPV vaccines (‘solicited’ and ‘unsolicited’) - ‘headache’: intention to treat analysis

Study or Subgroup	HPV vaccine Events	HPV vaccine Total	Control Events	Control Total	Weight	Risk Ratio IV, Random, 95% CI
**12.29.1 General headache ('solicited'): Cervarix vs. Al(OH)3 (females, age 9-17; follow-up until 12 months)**						
HPV-058: Cervarix vs. Al(OH)3 (f, 9-17; 12)	123	374	99	376	3.5%	1.25 [1.00, 1.56]
**Subtotal (95% CI)**		**374**		**376**	**3.5%**	**1.25 [1.00, 1.56]**
Total events	123		99			
Heterogeneity: Not applicable						
Test for overall effect: Z = 1.96 (P = 0.05)						
**12.29.2 General headache ('solicited'): Cervarix vs. Al(OH)3 (females, age 15-26; follow-up until 27 months)**						
HPV-001: Cervarix vs. Al(OH)3 (f, 15-26; 27)	331	560	329	553	10.2%	0.99 [0.90, 1.10]
HPV-038: Cervarix vs. Al(OH)3 (f, 15-25; 7)	73	149	30	76	1.8%	1.24 [0.90, 1.71]
**Subtotal (95% CI)**		**709**		**629**	**12.0%**	**1.05 [0.87, 1.27]**
Total events	404		359			
Heterogeneity: Tau² = 0.01; Chi² = 1.67, df = 1 (P = 0.20); I² = 40%						
Test for overall effect: Z = 0.51 (P = 0.61)						
**12.29.3 General headache ('solicited'): Cervarix vs. Al(OH)3 (females, age 18-35; follow-up until 12 months)**						
HPV-003: Cervarix vs. Al(OH)3 (f, 18-30; 12)	16	31	21	30	1.2%	0.74 [0.49, 1.12]
HPV-031: Cervarix vs. Al(OH)3 (f, 18-35; 7)	72	176	71	178	2.8%	1.03 [0.80, 1.32]
HPV-035: Cervarix vs. Al(OH)3 (f, 18-35; 7)	67	150	54	150	2.4%	1.24 [0.94, 1.64]
**Subtotal (95% CI)**		**357**		**358**	**6.4%**	**1.02 [0.79, 1.31]**
Total events	155		146			
Heterogeneity: Tau² = 0.03; Chi² = 4.22, df = 2 (P = 0.12); I² = 53%						
Test for overall effect: Z = 0.12 (P = 0.91)						
**12.29.4 General headache ('solicited'): Cervarix vs. Al(OH)3 (females, age 24-72; follow-up until 36 months)**						
HPV-015: Cervarix vs. Al(OH)3 (f, 24-72; 36)	1165	2882	1074	2871	13.6%	1.08 [1.01, 1.15]
**Subtotal (95% CI)**		**2882**		**2871**	**13.6%**	**1.08 [1.01, 1.15]**
Total events	1165		1074			
Heterogeneity: Not applicable						
Test for overall effect: Z = 2.34 (P = 0.02)						
**12.29.5 General headache ('solicited'): Cervarix vs. Engerix: hepatitis B vaccine (females and males, age 9-16; follow-up until 72 months)**						
HPV-030: Cervarix vs. Engerix (f, 9-15; 12)	131	247	129	246	5.4%	1.01 [0.86, 1.20]
HPV-040: Cervarix vs. Engerix (f & m, 12-16; 72)	261	604	371	1028	8.1%	1.20 [1.06, 1.35]
**Subtotal (95% CI)**		**851**		**1274**	**13.5%**	**1.11 [0.94, 1.31]**
Total events	392		500			
Heterogeneity: Tau² = 0.01; Chi² = 2.55, df = 1 (P = 0.11); I² = 61%						
Test for overall effect: Z = 1.26 (P = 0.21)						
**12.29.6 General headache ('solicited'): Cervarix vs. Engerix: hepatitis B vaccine (females, age 26-46; follow-up until 12 months)**						
HPV-069: Cervarix vs. Engerix (f, 26-46; 12)	71	606	46	606	1.6%	1.54 [1.08, 2.20]
**Subtotal (95% CI)**		**606**		**606**	**1.6%**	**1.54 [1.08, 2.20]**
Total events	71		46			
Heterogeneity: Not applicable						
Test for overall effect: Z = 2.41 (P = 0.02)						
**12.29.7 General headache ('solicited'): Cervarix vs. Havrix: hepatitis A vaccine (females, age 9-15; follow-up until 12 months)**						
HPV-013: Cervarix vs. Havrix (f, 10-14; 12)	516	1035	464	1032	10.8%	1.11 [1.01, 1.21]
HPV-033: Cervarix vs. Havrix (f, 9-15; 7)	44	160	53	161	1.7%	0.84 [0.60, 1.17]
**Subtotal (95% CI)**		**1195**		**1193**	**12.5%**	**1.01 [0.78, 1.31]**
Total events	560		517			
Heterogeneity: Tau² = 0.02; Chi² = 2.56, df = 1 (P = 0.11); I² = 61%						
Test for overall effect: Z = 0.07 (P = 0.94)						
**12.29.8 General headache ('solicited'): Cervarix vs. Havrix: hepatitis A vaccine (females, age 15-25; follow-up until 48 months)**						
HPV-008: Cervarix vs. Havrix (f, 15-25; 48)	1668	9319	1583	9325	13.9%	1.05 [0.99, 1.12]
**Subtotal (95% CI)**		**9319**		**9325**	**13.9%**	**1.05 [0.99, 1.12]**
Total events	1668		1583			
Heterogeneity: Not applicable						
Test for overall effect: Z = 1.66 (P = 0.10)						
**12.29.9 General headache ('solicited'): Cervarix vs. Twinrix: hepatitis A and B vaccine (females, age 8-15; follow-up until 12 months)**						
HPV-029: Cervarix vs. Twinrix (f, 8-15; 12)	124	270	110	271	4.3%	1.13 [0.93, 1.37]
**Subtotal (95% CI)**		**270**		**271**	**4.3%**	**1.13 [0.93, 1.37]**
Total events	124		110			
Heterogeneity: Not applicable						
Test for overall effect: Z = 1.25 (P = 0.21)						
**12.29.10 General headache ('unsolicited'): Cervarix vs. Al(OH)3 (females, age 9-17; follow-up until 12 months)**						
HPV-058: Cervarix vs. Al(OH)3 (f, 9-17; 12)	1	374	0	376	0.0%	3.02 [0.12, 73.80]
**Subtotal (95% CI)**		**0**		**0**		**Not estimable**
Total events	0		0			
Heterogeneity: Not applicable						
Test for overall effect: Not applicable						
**12.29.11 General headache ('unsolicited'): Cervarix vs. Al(OH)3 (females, age 15-25; follow-up until 7 months)**						
HPV-038: Cervarix vs. Al(OH)3 (f, 15-25; 7)	10	149	5	76	0.0%	1.02 [0.36, 2.88]
**Subtotal (95% CI)**		**0**		**0**		**Not estimable**
Total events	0		0			
Heterogeneity: Not applicable						
Test for overall effect: Not applicable						
**12.29.12 General headache ('unsolicited'): Cervarix vs. Al(OH)3 (females, age 18-35; follow-up until 7 months)**						
HPV-031: Cervarix vs. Al(OH)3 (f, 18-35; 7)	4	176	1	178	0.0%	4.05 [0.46, 35.84]
HPV-035: Cervarix vs. Al(OH)3 (f, 18-35; 7)	8	150	8	150	0.0%	1.00 [0.39, 2.59]
**Subtotal (95% CI)**		**0**		**0**		**Not estimable**
Total events	0		0			
Heterogeneity: Not applicable						
Test for overall effect: Not applicable						
**12.29.13 General headache ('unsolicited'): Cervarix vs. Engerix: hepatitis B vaccine (females and males, age 9-16; follow-up until 72 months)**						
HPV-030: Cervarix vs. Engerix (f, 9-15; 12)	24	247	11	246	0.0%	2.17 [1.09, 4.34]
HPV-040: Cervarix vs. Engerix (f & m, 12-16; 72)	26	1908	20	3128	0.0%	2.13 [1.19, 3.81]
**Subtotal (95% CI)**		**0**		**0**		**Not estimable**
Total events	0		0			
Heterogeneity: Not applicable						
Test for overall effect: Not applicable						
**12.29.14 General headache ('unsolicited'): Cervarix vs. Twinrix: hepatitis A and B vaccine (females, age 8-15; follow-up until 12 months)**						
HPV-029: Cervarix vs. Twinrix (f, 8-15; 12)	13	270	8	271	0.0%	1.63 [0.69, 3.87]
**Subtotal (95% CI)**		**0**		**0**		**Not estimable**
Total events	0		0			
Heterogeneity: Not applicable						
Test for overall effect: Not applicable						
**12.29.15 General headache ('systemic adverse events'): Gardasil vs. carrier solution (females and males, age 9-16; follow-up until 18 months)**						
V501-018: Gardasil vs. carrier solu. (f&m,9-16;18)	221	1184	110	597	3.9%	1.01 [0.82, 1.25]
**Subtotal (95% CI)**		**1184**		**597**	**3.9%**	**1.01 [0.82, 1.25]**
Total events	221		110			
Heterogeneity: Not applicable						
Test for overall effect: Z = 0.12 (P = 0.90)						
**12.29.16 General headache ('systemic adverse events'): Gardasil vs. AAHS (males, age 15-27; follow-up until 36 months)**						
V501-020: Gardasil vs. AAHS (m, 15-27; 36)	179	2032	207	2033	4.5%	0.87 [0.72, 1.05]
**Subtotal (95% CI)**		**2032**		**2033**	**4.5%**	**0.87 [0.72, 1.05]**
Total events	179		207			
Heterogeneity: Not applicable						
Test for overall effect: Z = 1.49 (P = 0.14)						
**12.29.17 General headache ('systemic adverse events'): HPV 16 vaccine vs. AAHS (females, age 16-25; follow-up until 48 months)**						
V501-005: HPV 16 vaccine vs. AAHS (f, 16-25; 48)	499	1204	495	1205	10.4%	1.01 [0.92, 1.11]
**Subtotal (95% CI)**		**1204**		**1205**	**10.4%**	**1.01 [0.92, 1.11]**
Total events	499		495			
Heterogeneity: Not applicable						
Test for overall effect: Z = 0.18 (P = 0.86)						
**Total (95% CI)**		**20983**		**20738**	**100.0%**	**1.06 [1.02, 1.11]**
Total events	5561		5246			
Heterogeneity: Tau² = 0.00; Chi² = 26.66, df = 16 (P = 0.05); I² = 40%						
Test for overall effect: Z = 2.62 (P = 0.009)						
Test for subgroup differences: Chi² = 13.39, df = 11 (P = 0.27), I² = 17.9%						

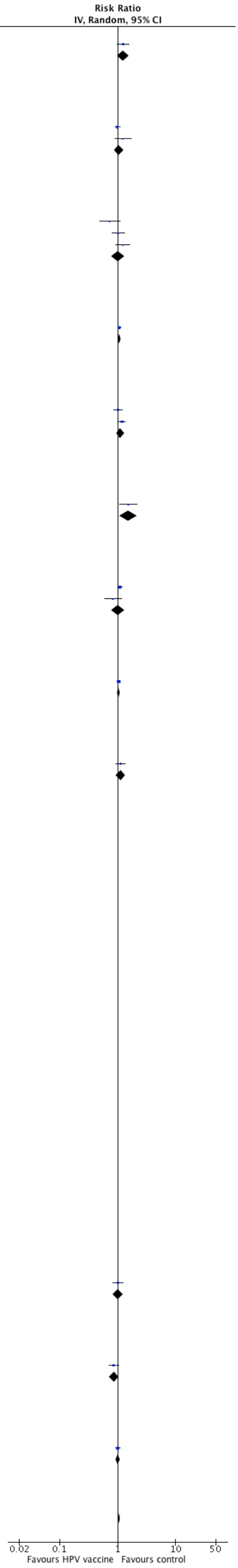

*1.19. Risk ratio for ‘solicited’ (GlaxoSmithKline): **1.08 [1.03, 1.14]**; risk ratio for ‘unsolicited’ (GlaxoSmithKline): **1.76 [1.26, 2.47]**; risk ratio for ‘systemic adverse events’ (Merck Sharp & Dohme): 0.98 [0.90, 1.07]. To avoid double counting of participants in the total risk ratio estimate, we excluded the ‘unsolicited’ adverse events from total risk ratio estimate for studies that reported ‘solicited’ adverse events.

1.20. General harms most associated with the HPV vaccines (‘solicited’ and ‘unsolicited’) - ‘myalgia’: intention to treat analysis

Study or Subgroup	HPV vaccine Events	HPV vaccine Total	Control Events	Control Total	Weight	Risk Ratio IV, Random, 95% CI
**12.30.1 General myalgia ('solicited'): Cervarix vs. Al(OH)3 (females, age 9-17; follow-up until 12 months)**						
HPV-058: Cervarix vs. Al(OH)3 (f, 9-17; 12)	110	374	93	376	7.9%	1.19 [0.94, 1.51]
**Subtotal (95% CI)**		**374**		**376**	**7.9%**	**1.19 [0.94, 1.51]**
Total events	110		93			
Heterogeneity: Not applicable						
Test for overall effect: Z = 1.44 (P = 0.15)						
**12.30.2 General myalgia ('solicited'): Cervarix vs. Al(OH)3 (females, age 15-25; follow-up until 7 months)**						
HPV-038: Cervarix vs. Al(OH)3 (f, 15-25; 7)	97	298	33	152	6.0%	1.50 [1.06, 2.11]
**Subtotal (95% CI)**		**298**		**152**	**6.0%**	**1.50 [1.06, 2.11]**
Total events	97		33			
Heterogeneity: Not applicable						
Test for overall effect: Z = 2.31 (P = 0.02)						
**12.30.3 General myalgia ('solicited'): Cervarix vs. Al(OH)3 (females, age 18-35; follow-up until 12 months)**						
HPV-003: Cervarix vs. Al(OH)3 (f, 18-30; 12)	1	31	0	30	0.2%	2.91 [0.12, 68.66]
HPV-031: Cervarix vs. Al(OH)3 (f, 18-35; 7)	10	176	11	178	1.9%	0.92 [0.40, 2.11]
HPV-035: Cervarix vs. Al(OH)3 (f, 18-35; 7)	88	150	61	150	7.9%	1.44 [1.14, 1.83]
**Subtotal (95% CI)**		**357**		**358**	**10.0%**	**1.40 [1.12, 1.76]**
Total events	99		72			
Heterogeneity: Tau^2 = 0.00; Chi^2 = 1.25, df = 2 (P = 0.53); I^2 = 0%						
Test for overall effect: Z = 2.92 (P = 0.003)						
**12.30.4 General myalgia ('solicited'): Cervarix vs. Al(OH)3 (females, age 24-72; follow-up until 36 months)**						
HPV-015: Cervarix vs. Al(OH)3 (f, 24-72; 36)	878	2882	622	2871	10.4%	1.41 [1.29, 1.54]
**Subtotal (95% CI)**		**2882**		**2871**	**10.4%**	**1.41 [1.29, 1.54]**
Total events	878		622			
Heterogeneity: Not applicable						
Test for overall effect: Z = 7.53 (P < 0.00001)						
**12.30.5 General myalgia ('solicited'): Cervarix vs. Engerix: hepatitis B vaccine (females and males, age 9-16; follow-up until 72 months)**						
HPV-030: Cervarix vs. Engerix (f, 9-15; 12)	55	247	53	246	6.2%	1.03 [0.74, 1.44]
HPV-040: Cervarix vs. Engerix (f & m, 12-16; 72)	321	604	250	1028	9.8%	2.19 [1.92, 2.49]
**Subtotal (95% CI)**		**851**		**1274**	**16.0%**	**1.53 [0.73, 3.18]**
Total events	376		303			
Heterogeneity: Tau^2 = 0.26; Chi^2 = 16.78, df = 1 (P < 0.0001); I^2 = 94%						
Test for overall effect: Z = 1.13 (P = 0.26)						
**12.30.6 General myalgia ('solicited'): Cervarix vs. Engerix: hepatitis B vaccine (females, age 26-46; follow-up until 12 months)**						
HPV-069: Cervarix vs. Engerix (f, 26-46; 12)	69	606	42	606	5.7%	1.64 [1.14, 2.37]
**Subtotal (95% CI)**		**606**		**606**	**5.7%**	**1.64 [1.14, 2.37]**
Total events	69		42			
Heterogeneity: Not applicable						
Test for overall effect: Z = 2.65 (P = 0.008)						
**12.30.7 General myalgia ('solicited'): Cervarix vs. Havrix: hepatitis A vaccine (females, age 9-15; follow-up until 12 months)**						
HPV-013: Cervarix vs. Havrix (f, 10-14; 12)	509	1035	340	1032	10.1%	1.49 [1.34, 1.66]
HPV-033: Cervarix vs. Havrix (f, 9-15; 7)	49	160	29	161	5.2%	1.70 [1.14, 2.55]
**Subtotal (95% CI)**		**1195**		**1193**	**15.3%**	**1.51 [1.36, 1.67]**
Total events	558		369			
Heterogeneity: Tau^2 = 0.00; Chi^2 = 0.37, df = 1 (P = 0.54); I^2 = 0%						
Test for overall effect: Z = 7.76 (P < 0.00001)						
**12.30.8 General myalgia ('solicited'): Cervarix vs. Havrix: hepatitis A vaccine (females, age 15-25; follow-up until 48 months)**						
HPV-008: Cervarix vs. Havrix (f, 15-25; 48)	1607	9319	1381	9325	10.6%	1.16 [1.09, 1.24]
**Subtotal (95% CI)**		**9319**		**9325**	**10.6%**	**1.16 [1.09, 1.24]**
Total events	1607		1381			
Heterogeneity: Not applicable						
Test for overall effect: Z = 4.52 (P < 0.00001)						
**12.30.9 General myalgia ('solicited'): Cervarix vs. Twinrix: hepatitis A and B vaccine (females, age 8-15; follow-up until 12 months)**						
HPV-029: Cervarix vs. Twinrix (f, 8-15; 12)	99	270	75	271	7.7%	1.32 [1.03, 1.70]
**Subtotal (95% CI)**		**270**		**271**	**7.7%**	**1.32 [1.03, 1.70]**
Total events	99		75			
Heterogeneity: Not applicable						
Test for overall effect: Z = 2.22 (P = 0.03)						
**12.30.10 General myalgia ('unsolicited'): Cervarix vs. Al(OH)3 (females, age 15-25; follow-up until 7 months)**						
HPV-038: Cervarix vs. Al(OH)3 (f, 15-25; 7)	1	149	0	76	0.0%	1.54 [0.06, 37.36]
**Subtotal (95% CI)**		**0**		**0**		**Not estimable**
Total events	0		0			
Heterogeneity: Not applicable						
Test for overall effect: Not applicable						
**12.30.11 General myalgia ('unsolicited'): Cervarix vs. Al(OH)3 (females, age 18-35; follow-up until 7 months)**						
HPV-031: Cervarix vs. Al(OH)3 (f, 18-35; 7)	0	176	1	178	0.0%	0.34 [0.01, 8.22]
**Subtotal (95% CI)**		**0**		**0**		**Not estimable**
Total events	0		0			
Heterogeneity: Not applicable						
Test for overall effect: Not applicable						
**12.30.12 General myalgia ('unsolicited'): Cervarix vs. Engerix: hepatitis B vaccine (females and males, age 12-16; follow-up until 72 months)**						
HPV-040: Cervarix vs. Engerix (f & m, 12-16; 72)	2	1908	0	3128	0.0%	8.20 [0.39, 170.61]
**Subtotal (95% CI)**		**0**		**0**		**Not estimable**
Total events	0		0			
Heterogeneity: Not applicable						
Test for overall effect: Not applicable						
**12.30.13 General myalgia ('unsolicited'): Cervarix vs. Havrix: hepatitis A vaccine (females, age 9-15; follow-up until 7 months)**						
HPV-033: Cervarix vs. Havrix (f, 9-15; 7)	0	160	1	161	0.2%	0.34 [0.01, 8.17]
**Subtotal (95% CI)**		**160**		**161**	**0.2%**	**0.34 [0.01, 8.17]**
Total events	0		1			
Heterogeneity: Not applicable						
Test for overall effect: Z = 0.67 (P = 0.50)						
**12.30.14 General myalgia ('systemic adverse events'): Gardasil vs. carrier solution (females and males, age 9-16; follow-up until 18 months)**						
V501-018: Gardasil vs. carrier solu. (f&m,9-16;18)	31	1184	10	597	2.5%	1.56 [0.77, 3.17]
**Subtotal (95% CI)**		**1184**		**597**	**2.5%**	**1.56 [0.77, 3.17]**
Total events	31		10			
Heterogeneity: Not applicable						
Test for overall effect: Z = 1.24 (P = 0.21)						
**12.30.15 General myalgia ('systemic adverse events'): Gardasil vs. AAHS (males, age 15-27; follow-up until 36 months)**						
V501-020: Gardasil vs. AAHS (m, 15-27; 36)	18	2032	11	2033	2.3%	1.64 [0.78, 3.46]
**Subtotal (95% CI)**		**2032**		**2033**	**2.3%**	**1.64 [0.78, 3.46]**
Total events	18		11			
Heterogeneity: Not applicable						
Test for overall effect: Z = 1.29 (P = 0.20)						
**12.30.16 General myalgia ('systemic adverse events'): Gardasil 9 vs. saline placebo (females, age 12-26; follow-up until 7 months)**						
V503-006: Gardasil 9 vs. saline (f, 12-26;7)	13	618	5	306	1.3%	1.29 [0.46, 3.58]
**Subtotal (95% CI)**		**618**		**306**	**1.3%**	**1.29 [0.46, 3.58]**
Total events	13		5			
Heterogeneity: Not applicable						
Test for overall effect: Z = 0.48 (P = 0.63)						
**12.30.17 General myalgia ('systemic adverse events'): HPV 16 vaccine vs. AAHS (females, age 16-25; follow-up until 48 months)**						
V501-005: HPV 16 vaccine vs. AAHS (f, 16-25; 48)	34	1204	30	1205	4.2%	1.13 [0.70, 1.84]
**Subtotal (95% CI)**		**1204**		**1205**	**4.2%**	**1.13 [0.70, 1.84]**
Total events	34		30			
Heterogeneity: Not applicable						
Test for overall effect: Z = 0.51 (P = 0.61)						
**Total (95% CI)**		**21350**		**20728**	**100.0%**	**1.41 [1.24, 1.60]**
Total events	3989		3047			
Heterogeneity: Tau^2 = 0.04; Chi^2 = 84.24, df = 17 (P < 0.00001); I^2 = 80%						
Test for overall effect: Z = 5.34 (P < 0.00001)						
Test for subgroup differences: Chi^2 = 26.48, df = 13 (P = 0.01), I^2 = 50.9%						

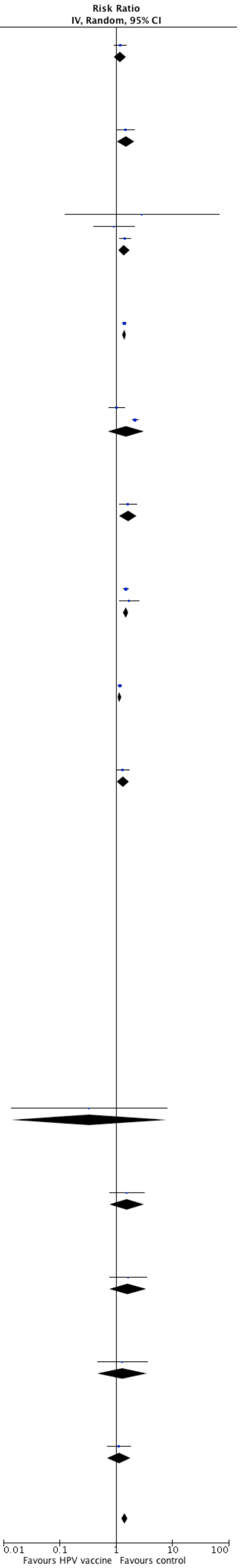

*1.20. Risk ratio for ‘solicited’ (GlaxoSmithKline): **1.42 [1.24, 1.63]**; risk ratio for ‘unsolicited’ (GlaxoSmithKline): 1.15 [0.24, 5.57]; risk ratio for ‘systemic adverse events’ (Merck Sharp & Dohme): 1.33 [0.95, 1.85]. To avoid double counting of participants in the total risk ratio estimate, we excluded the ‘unsolicited’ adverse events from total risk ratio estimate for studies that reported ‘solicited’ adverse events.

2. Trial register entries

2.1. All-cause mortality/deaths*: intention to treat analysis

Study or Subgroup	HPV vaccine Events	HPV vaccine Total	Control Events	Control Total	Weight	Risk Ratio IV, Random, 95% CI
**2.1.1 All-cause mortality/deaths: Cervarix vs. Al(OH)3 (females, age 24-72; follow-up until 36 months)**						
HPV-015: Cervarix vs. Al(OH)3 (f, 24-72; 36)	13	2882	5	2871	21.5%	2.59 [0.92, 7.26]
**Subtotal (95% CI)**		**2882**		**2871**	**21.5%**	**2.59 [0.92, 7.26]**
Total events	13		5			
Heterogeneity: Not applicable						
Test for overall effect: Z = 1.81 (P = 0.07)						
**2.1.2 All-cause mortality/deaths: Cervarix vs. Aimmugen: hepatitis A vaccine (females, age 19-25; follow-up until 7 months)**						
HPV-032: Cervarix vs. Aimmugen (f, 19-25; 7)	1	519	0	521	3.1%	3.01 [0.12, 73.76]
**Subtotal (95% CI)**		**519**		**521**	**3.1%**	**3.01 [0.12, 73.76]**
Total events	1		0			
Heterogeneity: Not applicable						
Test for overall effect: Z = 0.68 (P = 0.50)						
**2.1.3 All-cause mortality/deaths: Cervarix vs. Engerix: hepatitis B vaccine (females and males, age 9-16; follow-up until 72 months)**						
HPV-040: Cervarix vs. Engerix (f & m, 12-16; 72)	5	14837	5	17338	16.4%	1.17 [0.34, 4.04]
**Subtotal (95% CI)**		**14837**		**17338**	**16.4%**	**1.17 [0.34, 4.04]**
Total events	5		5			
Heterogeneity: Not applicable						
Test for overall effect: Z = 0.25 (P = 0.81)						
**2.1.4 All-cause mortality/deaths: Cervarix vs. Engerix: hepatitis B vaccine (females, age 26-46; follow-up until 12 months)**						
HPV-069: Cervarix vs. Engerix (f, 26-46; 12)	0	606	0	606		Not estimable
**Subtotal (95% CI)**		**606**		**606**		**Not estimable**
Total events	0		0			
Heterogeneity: Not applicable						
Test for overall effect: Not applicable						
**2.1.5 All-cause mortality/deaths: Cervarix vs. Havrix: hepatitis A vaccine (females, age 15-25; follow-up until 48 months)**						
HPV-008: Cervarix vs. Havrix (f, 15-25; 48)	0	9319	0	9325		Not estimable
**Subtotal (95% CI)**		**9319**		**9325**		**Not estimable**
Total events	0		0			
Heterogeneity: Not applicable						
Test for overall effect: Not applicable						
**2.1.6 All-cause mortality/deaths: Gardasil vs. AAHS (females, age 15-26; follow-up until 45 months)**						
V501-013: Gardasil vs. AAHS (f, 16-24; 45)	2	2723	2	2732	7.7%	1.00 [0.14, 7.12]
V501-015: Gardasil vs. AAHS (f, 15-26; 36)	7	6087	5	6080	18.5%	1.40 [0.44, 4.40]
**Subtotal (95% CI)**		**8810**		**8812**	**26.1%**	**1.28 [0.48, 3.46]**
Total events	9		7			
Heterogeneity: Tau² = 0.00; Chi² = 0.08, df = 1 (P = 0.77); I² = 0%						
Test for overall effect: Z = 0.50 (P = 0.62)						
**2.1.7 All-cause mortality/deaths: Gardasil vs. AAHS (females, age 21-46; follow-up until 48 months)**						
V501-019: Gardasil vs. AAHS (f, 21-46; 48)	8	1911	4	1908	17.3%	2.00 [0.60, 6.62]
**Subtotal (95% CI)**		**1911**		**1908**	**17.3%**	**2.00 [0.60, 6.62]**
Total events	8		4			
Heterogeneity: Not applicable						
Test for overall effect: Z = 1.13 (P = 0.26)						
**2.1.8 All-cause mortality/deaths: Gardasil vs. AAHS (males, age 15-27; follow-up until 36 months)**						
V501-020: Gardasil vs. AAHS (m, 15-27; 36)	3	2032	10	2033	15.5%	0.30 [0.08, 1.09]
**Subtotal (95% CI)**		**2032**		**2033**	**15.5%**	**0.30 [0.08, 1.09]**
Total events	3		10			
Heterogeneity: Not applicable						
Test for overall effect: Z = 1.83 (P = 0.07)						
**2.1.9 All-cause mortality/deaths: HPV 16 vaccine vs. AAHS (females, age 16-25; follow-up until 48 months)**						
V501-005: HPV 16 vaccine vs. AAHS (f, 16-25; 48)	0	1204	0	1205		Not estimable
**Subtotal (95% CI)**		**1204**		**1205**		**Not estimable**
Total events	0		0			
Heterogeneity: Not applicable						
Test for overall effect: Not applicable						
**Total (95% CI)**		**42120**		**44619**	**100.0%**	**1.30 [0.73, 2.30]**
Total events	39		31			
Heterogeneity: Tau² = 0.12; Chi² = 7.55, df = 6 (P = 0.27); I² = 21%						
Test for overall effect: Z = 0.89 (P = 0.37)						
Test for subgroup differences: Chi² = 7.47, df = 5 (P = 0.19), I² = 33.1%						

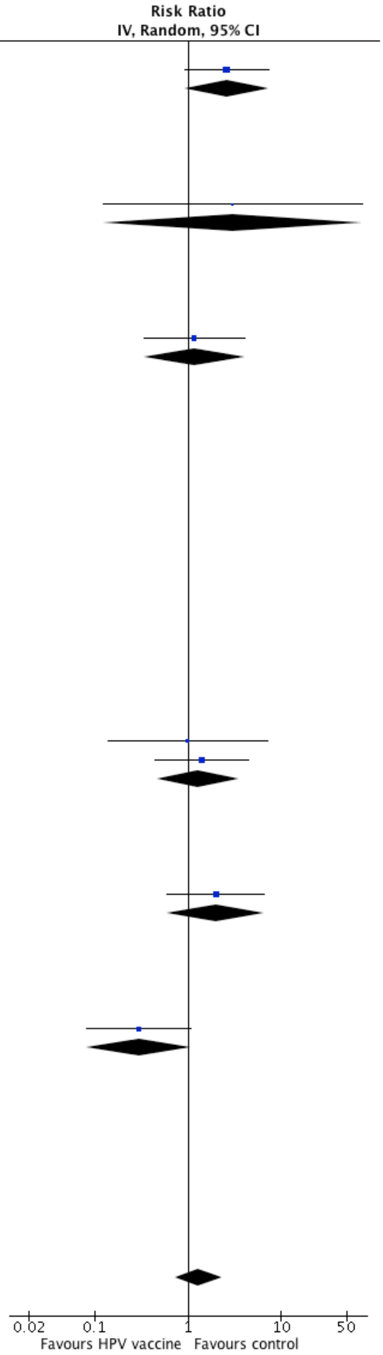

*2.1. Risk ratio for GlaxoSmithKline studies (i.e., HPV-0xx): 1.92 [0.89, 4.15]; risk ratio for Merck Sharp & Dohme studies (i.e., V50x-xxx): 0.98 [0.41, 2.33].

2.2. Mortality/deaths from HPV-related cancers (anal, cervical, oropharyngeal, penile, vaginal and vulvar) irrespective of HPV type: intention to treat analysis

Study or Subgroup	HPV vaccine Events	HPV vaccine Total	Control Events	Control Total	Weight	Risk Ratio IV, Random, 95% CI
**2.2.1 Cervical cancer: Cervarix (females, age 24–72; follow-up until 36 months)**						
HPV-015: Cervarix vs. Al(OH)3 (f, 24–72; 36)	0	2882	0	2871		Not estimable
**Subtotal (95% CI)**		**2882**		**2871**		**Not estimable**
Total events	0		0			
Heterogeneity: Not applicable						
Test for overall effect: Not applicable						
**2.2.2 Oropharyngeal cancer: Cervarix (females, age 24–72; follow-up until 36 months)**						
HPV-015: Cervarix vs. Al(OH)3 (f, 24–72; 36)	0	2882	0	2871		Not estimable
**Subtotal (95% CI)**		**2882**		**2871**		**Not estimable**
Total events	0		0			
Heterogeneity: Not applicable						
Test for overall effect: Not applicable						
**2.2.3 Oropharyngeal cancer: Gardasil (females, age 21–46; follow-up until 48 months)**						
V501-019: Gardasil vs. AAHS (f, 21–46; 48)	0	1911	0	1908		Not estimable
**Subtotal (95% CI)**		**1911**		**1908**		**Not estimable**
Total events	0		0			
Heterogeneity: Not applicable						
Test for overall effect: Not applicable						
**Total (95% CI)**		**7675**		**7650**		**Not estimable**
Total events	0		0			
Heterogeneity: Not applicable						
Test for overall effect: Not applicable						
Test for subgroup differences: Not applicable						

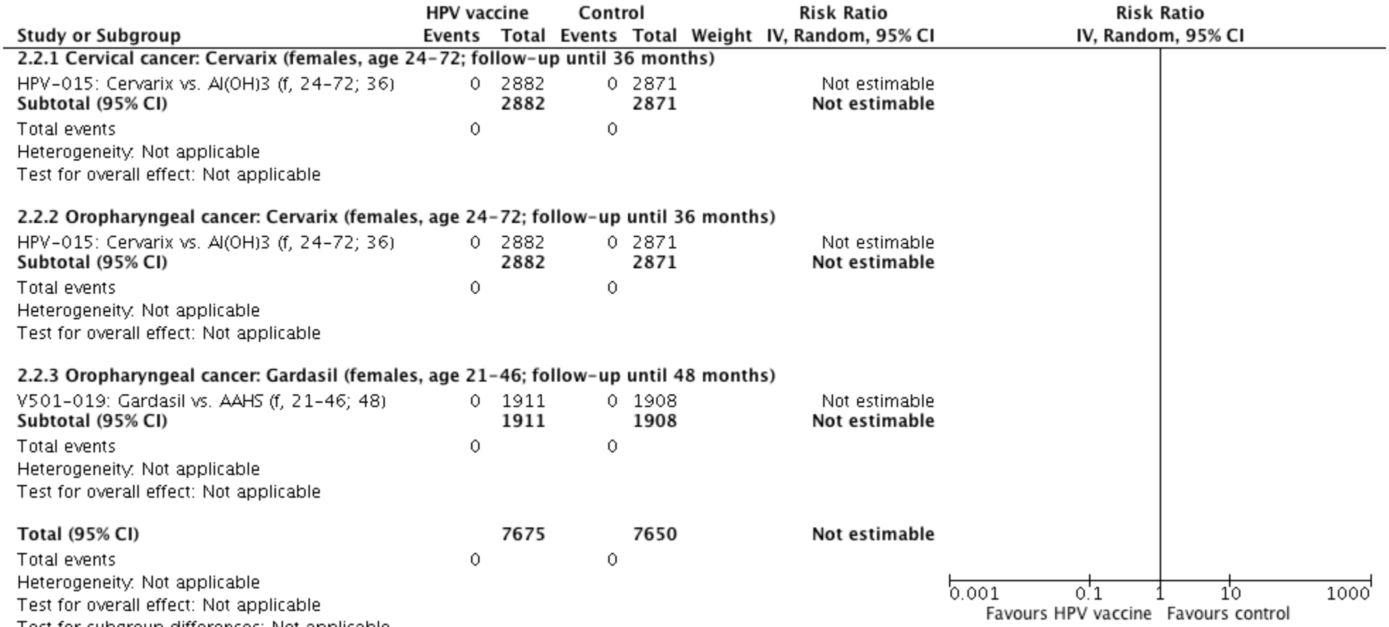

*2.2. Risk ratio for 'medically significant conditions' (GlaxoSmithKline): not applicable; risk ratio for 'new medical history' (Merck Sharp & Dohme): not applicable.

2.3. Incidence of HPV-related cancers (anal, cervical, oropharyngeal, penile, vaginal and vulvar) irrespective of HPV type: intention to treat analysis

Study or Subgroup	HPV vaccine Events	HPV vaccine Total	Control Events	Control Total	Weight	Risk Ratio IV, Random, 95% CI
**2.3.1 Cervical cancer: Cervarix (females, age 24-72; follow-up until 36 months)**						
HPV-015: Cervarix vs. Al(OH)3 (f, 24-72; 36)	0	2882	0	2871		Not estimable
**Subtotal (95% CI)**		**2882**		**2871**		**Not estimable**
Total events	0		0			
Heterogeneity: Not applicable						
Test for overall effect: Not applicable						
**2.3.2 Cervical cancer: Gardasil (females, age 21-46; follow-up until 48 months)**						
V501-019: Gardasil vs. AAHS (f, 21-46; 48)	0	1911	0	1908		Not estimable
**Subtotal (95% CI)**		**1911**		**1908**		**Not estimable**
Total events	0		0			
Heterogeneity: Not applicable						
Test for overall effect: Not applicable						
**2.3.3 Oropharyngeal cancer: Cervarix (females, age 24-72; follow-up until 36 months)**						
HPV-015: Cervarix vs. Al(OH)3 (f, 24-72; 36)	0	2882	0	2871		Not estimable
**Subtotal (95% CI)**		**2882**		**2871**		**Not estimable**
Total events	0		0			
Heterogeneity: Not applicable						
Test for overall effect: Not applicable						
**2.3.4 Oropharyngeal cancer: Gardasil (females, age 21-46; follow-up until 48 months)**						
V501-019: Gardasil vs. AAHS (f, 21-46; 48)	0	1911	0	1908		Not estimable
**Subtotal (95% CI)**		**1911**		**1908**		**Not estimable**
Total events	0		0			
Heterogeneity: Not applicable						
Test for overall effect: Not applicable						
**2.3.5 Vaginal cancer: Cervarix (females, age 24-72; follow-up until 36 months)**						
HPV-015: Cervarix vs. Al(OH)3 (f, 24-72; 36)	0	2882	0	2871		Not estimable
**Subtotal (95% CI)**		**2882**		**2871**		**Not estimable**
Total events	0		0			
Heterogeneity: Not applicable						
Test for overall effect: Not applicable						
**2.3.6 Vulvar cancer: Cervarix (females, age 15-25; follow-up until 48 months)**						
HPV-008: Cervarix vs. Havrix (f, 15-25; 48)	0	9319	0	9325		Not estimable
**Subtotal (95% CI)**		**9319**		**9325**		**Not estimable**
Total events	0		0			
Heterogeneity: Not applicable						
Test for overall effect: Not applicable						
**2.3.7 Vulvar cancer: Gardasil (females, age 15-26; follow-up until 45 months)**						
V501-013: Gardasil vs. AAHS (f, 16-24; 45)	0	2723	0	2732		Not estimable
**Subtotal (95% CI)**		**2723**		**2732**		**Not estimable**
Total events	0		0			
Heterogeneity: Not applicable						
Test for overall effect: Not applicable						
**Total (95% CI)**		**24510**		**24486**		**Not estimable**
Total events	0		0			
Heterogeneity: Not applicable						
Test for overall effect: Not applicable						
Test for subgroup differences: Not applicable						

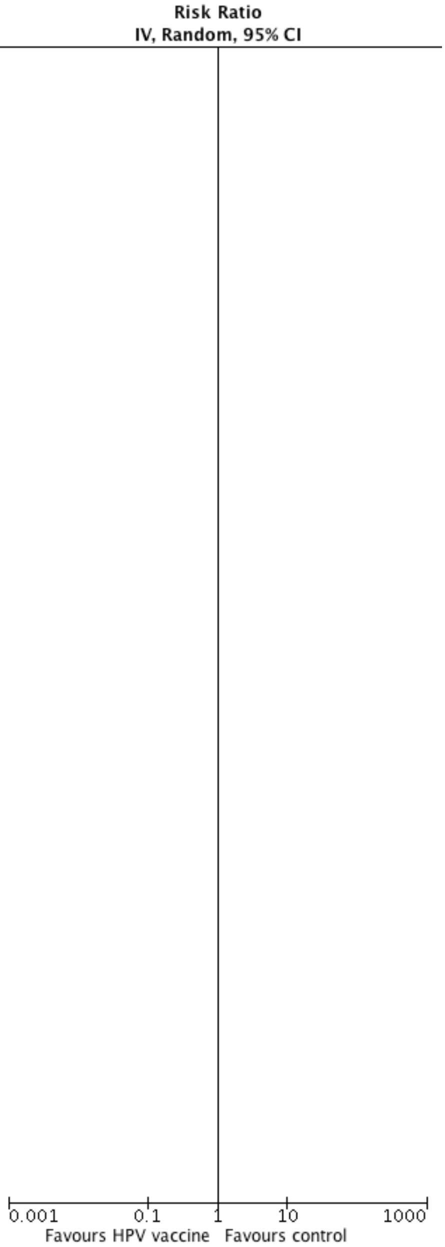

*2.3. Risk ratio for 'medically significant conditions' (GlaxoSmithKline): not applicable; risk ratio for 'new medical history' (Merck Sharp & Dohme): not applicable.

2.4. Incidence of HPV-related carcinoma in situ (anal intraepithelial neoplasia grade 3 [AIN3], cervical adenocarcinoma in situ [AIS], cervical intraepithelial neoplasia grade 3 [CIN3], penile intraepithelial neoplasia grade 3 [PIN3], vaginal intraepithelial neoplasia grade 3 [VIN3] and vulvar intraepithelial neoplasia grade 3 [VaIN3]) irrespective of HPV type: intention to treat analysis

	HPV vaccine		Control			Risk Ratio
**Study or Subgroup**	**Events**	**Total**	**Events**	**Total**	**Weight**	**IV, Random, 95% CI**
**2.4.1 AIS: Cervarix (females, age 15-25; follow-up until 48 months)**						
HPV-008: Cervarix vs. Havrix (f, 15-25; 48)	0	9319	0	9325		Not estimable
**Subtotal (95% CI)**		**9319**		**9325**		**Not estimable**
Total events	0		0			
Heterogeneity: Not applicable						
Test for overall effect: Not applicable						
**2.4.2 AIS: Gardasil (females, age 15-26; follow-up until 45 months)**						
V501-013: Gardasil vs. AAHS (f, 16-24; 45)	0	2723	0	2732		Not estimable
V501-015: Gardasil vs. AAHS (f, 15-26; 36)	0	6087	0	6080		Not estimable
**Subtotal (95% CI)**		**8810**		**8812**		**Not estimable**
Total events	0		0			
Heterogeneity: Not applicable						
Test for overall effect: Not applicable						
**2.4.3 AIS: Gardasil (females, age 21-46; follow-up until 48 months)**						
V501-019: Gardasil vs. AAHS (f, 21-46; 48)	0	1911	0	1908		Not estimable
**Subtotal (95% CI)**		**1911**		**1908**		**Not estimable**
Total events	0		0			
Heterogeneity: Not applicable						
Test for overall effect: Not applicable						
**2.4.4 CIN3: Cervarix (females, age 15-25; follow-up until 48 months)**						
HPV-008: Cervarix vs. Havrix (f, 15-25; 48)	0	9319	0	9325		Not estimable
**Subtotal (95% CI)**		**9319**		**9325**		**Not estimable**
Total events	0		0			
Heterogeneity: Not applicable						
Test for overall effect: Not applicable						
**2.4.5 CIN3: Gardasil (females, age 15-26; follow-up until 45 months)**						
V501-013: Gardasil vs. AAHS (f, 16-24; 45)	0	2723	0	2732		Not estimable
V501-015: Gardasil vs. AAHS (f, 15-26; 36)	0	6087	0	6080		Not estimable
**Subtotal (95% CI)**		**8810**		**8812**		**Not estimable**
Total events	0		0			
Heterogeneity: Not applicable						
Test for overall effect: Not applicable						
**2.4.6 CIN3: Gardasil (females, age 21-46; follow-up until 48 months)**						
V501-019: Gardasil vs. AAHS (f, 21-46; 48)	0	1911	0	1908		Not estimable
**Subtotal (95% CI)**		**1911**		**1908**		**Not estimable**
Total events	0		0			
Heterogeneity: Not applicable						
Test for overall effect: Not applicable						
**Total (95% CI)**		**40080**		**40090**		**Not estimable**
Total events	0		0			
Heterogeneity: Not applicable						
Test for overall effect: Not applicable						
Test for subgroup differences: Not applicable						

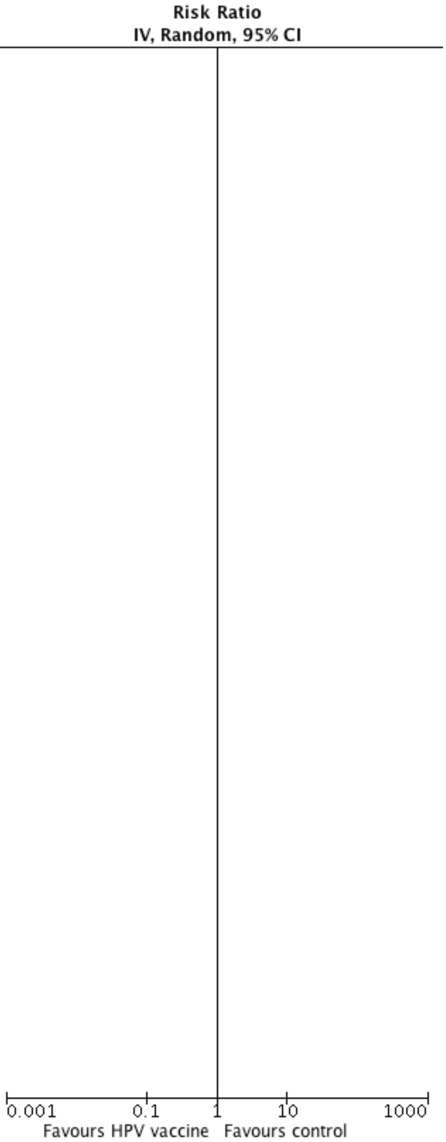

*2.4. Risk ratio for 'medically significant conditions' (GlaxoSmithKline): not applicable; risk ratio for 'new medical history' (Merck Sharp & Dohme): not applicable.

2.5. Incidence of HPV-related moderate intraepithelial neoplasia (anal intraepithelial neoplasia grade 2 [AIN2], cervical intraepithelial neoplasia grade 2 [CIN2], penile intraepithelial neoplasia grade 2 [PIN2], vaginal intraepithelial neoplasia grade 2 [VIN2] and vulvar intraepithelial neoplasia grade 2 [VaIN2]) irrespective of HPV type: intention to treat analysis

	HPV vaccine		Control			Risk Ratio
**Study or Subgroup**	**Events**	**Total**	**Events**	**Total**	**Weight**	**IV, Random, 95% CI**
**2.5.1 CIN2: Cervarix (females, age 15-25; follow-up until 48 months)**						
HPV-008: Cervarix vs. Havrix (f, 15-25; 48)	0	9319	0	9325		Not estimable
**Subtotal (95% CI)**		**9319**		**9325**		**Not estimable**
Total events	0		0			
Heterogeneity: Not applicable						
Test for overall effect: Not applicable						
**2.5.2 CIN2: Gardasil (females, age 15-26; follow-up until 45 months)**						
V501-013: Gardasil vs. AAHS (f, 16-24; 45)	0	2723	0	2732		Not estimable
V501-015: Gardasil vs. AAHS (f, 15-26; 36)	0	6087	0	6080		Not estimable
**Subtotal (95% CI)**		**8810**		**8812**		**Not estimable**
Total events	0		0			
Heterogeneity: Not applicable						
Test for overall effect: Not applicable						
**2.5.3 CIN2: Gardasil (females, age 21-46; follow-up until 48 months)**						
V501-019: Gardasil vs. AAHS (f, 21-46; 48)	0	1911	0	1908		Not estimable
**Subtotal (95% CI)**		**1911**		**1908**		**Not estimable**
Total events	0		0			
Heterogeneity: Not applicable						
Test for overall effect: Not applicable						
**Total (95% CI)**		**20040**		**20045**		**Not estimable**
Total events	0		0			
Heterogeneity: Not applicable						
Test for overall effect: Not applicable						
Test for subgroup differences: Not applicable						

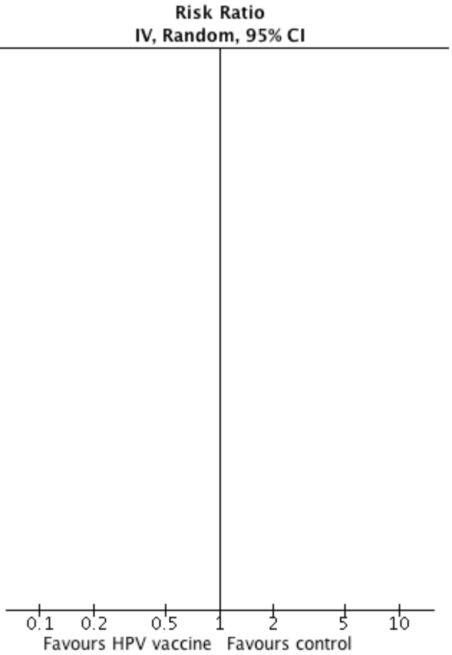

*2.5. Risk ratio for 'medically significant conditions' (GlaxoSmithKline): not applicable; risk ratio for 'new medical history' (Merck Sharp & Dohme): not applicable.

2.6. Incidence of HPV-related moderate intraepithelial neoplasia or worse (AIN2$^+$, CIN2$^+$, PIN2$^+$, VIN2$^+$, VaIN2$^+$) irrespective of HPV type: intention to treat analysis

Study or Subgroup	HPV vaccine Events	HPV vaccine Total	Control Events	Control Total	Weight	Risk Ratio IV, Random, 95% CI
**2.6.1 CIN2+: Cervarix (females, age 15-25; follow-up until 48 months)**						
HPV-008: Cervarix vs. Havrix (f, 15-25; 48)	0	9319	0	9325		Not estimable
**Subtotal (95% CI)**		**9319**		**9325**		**Not estimable**
Total events	0		0			
Heterogeneity: Not applicable						
Test for overall effect: Not applicable						
**2.6.2 CIN2+: Cervarix (females, age 22-29; follow-up until 12 months)**						
HPV-063: Cervarix vs. Aimmugen (f, 22-29; 12)	0	464	0	463		Not estimable
**Subtotal (95% CI)**		**464**		**463**		**Not estimable**
Total events	0		0			
Heterogeneity: Not applicable						
Test for overall effect: Not applicable						
**2.6.3 CIN2+: Cervarix (females, age 24-72; follow-up until 36 months)**						
HPV-015: Cervarix vs. Al(OH)3 (f, 24-72; 36)	0	2882	0	2871		Not estimable
**Subtotal (95% CI)**		**2882**		**2871**		**Not estimable**
Total events	0		0			
Heterogeneity: Not applicable						
Test for overall effect: Not applicable						
**2.6.4 CIN2+: Gardasil (females, age 15-26; follow-up until 45 months)**						
V501-013: Gardasil vs. AAHS (f, 16-24; 45)	0	2723	0	2732		Not estimable
V501-015: Gardasil vs. AAHS (f, 15-26; 36)	0	6087	0	6080		Not estimable
**Subtotal (95% CI)**		**8810**		**8812**		**Not estimable**
Total events	0		0			
Heterogeneity: Not applicable						
Test for overall effect: Not applicable						
**2.6.5 CIN2+: Gardasil (females, age 21-46; follow-up until 48 months)**						
V501-019: Gardasil vs. AAHS (f, 21-46; 48)	0	1911	0	1908		Not estimable
**Subtotal (95% CI)**		**1911**		**1908**		**Not estimable**
Total events	0		0			
Heterogeneity: Not applicable						
Test for overall effect: Not applicable						
**2.6.6 PIN2+: Gardasil (males, age 15-27; follow-up until 36 months)**						
V501-020: Gardasil vs. AAHS (m, 15-27; 36)	0	2032	0	2033		Not estimable
**Subtotal (95% CI)**		**2032**		**2033**		**Not estimable**
Total events	0		0			
Heterogeneity: Not applicable						
Test for overall effect: Not applicable						
**2.6.7 VIN2+: Gardasil (females, age 15-26; follow-up until 45 months)**						
V501-013: Gardasil vs. AAHS (f, 16-24; 45)	0	2723	0	2732		Not estimable
V501-015: Gardasil vs. AAHS (f, 15-26; 36)	0	6087	0	6080		Not estimable
**Subtotal (95% CI)**		**8810**		**8812**		**Not estimable**
Total events	0		0			
Heterogeneity: Not applicable						
Test for overall effect: Not applicable						
**2.6.8 VaIN2+: Gardasil (females, age 15-26; follow-up until 45 months)**						
V501-013: Gardasil vs. AAHS (f, 16-24; 45)	0	2723	0	2732		Not estimable
V501-015: Gardasil vs. AAHS (f, 15-26; 36)	0	6087	0	6080		Not estimable
**Subtotal (95% CI)**		**8810**		**8812**		**Not estimable**
Total events	0		0			
Heterogeneity: Not applicable						
Test for overall effect: Not applicable						
**2.6.9 VaIN2+: Gardasil (females, age 21-46; follow-up until 48 months)**						
V501-019: Gardasil vs. AAHS (f, 21-46; 48)	0	1911	0	1908		Not estimable
**Subtotal (95% CI)**		**1911**		**1908**		**Not estimable**
Total events	0		0			
Heterogeneity: Not applicable						
Test for overall effect: Not applicable						
**2.6.10 VIN2+ or VaIN2+: Cervarix (females, age 15-25; follow-up until 48 months)**						
HPV-008: Cervarix vs. Havrix (f, 15-25; 48)	0	9319	0	9325		Not estimable
**Subtotal (95% CI)**		**9319**		**9325**		**Not estimable**
Total events	0		0			
Heterogeneity: Not applicable						
Test for overall effect: Not applicable						
**Total (95% CI)**		**54268**		**54269**		**Not estimable**
Total events	0		0			
Heterogeneity: Not applicable						
Test for overall effect: Not applicable						
Test for subgroup differences: Not applicable						

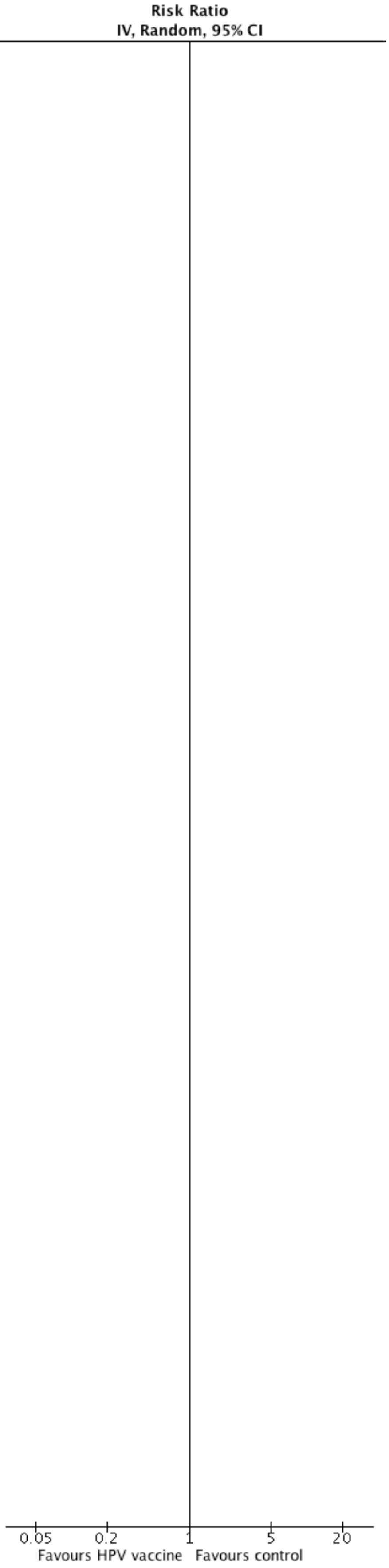

*2.6. Risk ratio for 'medically significant conditions' (GlaxoSmithKline): not applicable; risk ratio for 'new medical history' (Merck Sharp & Dohme): not applicable.

2.7. Number of treatment procedures (both surgical and non-surgical treatment) due to HPV-related diseases irrespective of HPV type: intention to treat analysis

Study or Subgroup	HPV vaccine Events	HPV vaccine Total	Control Events	Control Total	Weight	Risk Ratio IV, Random, 95% CI
**2.7.1 Treatment procedures: Cervarix (females, age 15-25; follow-up until 48 months)**						
HPV-008: Cervarix vs. Havrix (f, 15-25; 48)	0	9319	0	9325		Not estimable
**Subtotal (95% CI)**		**9319**		**9325**		**Not estimable**
Total events	0		0			
Heterogeneity: Not applicable						
Test for overall effect: Not applicable						
**2.7.2 Treatment procedures: Cervarix (females, age 24-72; follow-up until 36 months)**						
HPV-015: Cervarix vs. Al(OH)3 (f, 24-72; 36)	76	2882	84	2871	100.0%	0.90 [0.66, 1.22]
**Subtotal (95% CI)**		**2882**		**2871**	**100.0%**	**0.90 [0.66, 1.22]**
Total events	76		84			
Heterogeneity: Not applicable						
Test for overall effect: Z = 0.67 (P = 0.51)						
**2.7.3 Treatment procedures: Gardasil (females, age 15-26; follow-up until 45 months)**						
V501-013: Gardasil vs. AAHS (f, 16-24; 45)	0	2723	0	2732		Not estimable
V501-015: Gardasil vs. AAHS (f, 15-26; 36)	0	6087	0	6080		Not estimable
**Subtotal (95% CI)**		**8810**		**8812**		**Not estimable**
Total events	0		0			
Heterogeneity: Not applicable						
Test for overall effect: Not applicable						
**2.7.4 Treatment procedures: Gardasil (females, age 21-46; follow-up until 48 months)**						
V501-019: Gardasil vs. AAHS (f, 21-46; 48)	0	1911	0	1908		Not estimable
**Subtotal (95% CI)**		**1911**		**1908**		**Not estimable**
Total events	0		0			
Heterogeneity: Not applicable						
Test for overall effect: Not applicable						
**2.7.5 Treatment procedures: Gardasil (males, age 15-27; follow-up until 36 months)**						
V501-020: Gardasil vs. AAHS (m, 15-27; 36)	0	2032	0	2033		Not estimable
**Subtotal (95% CI)**		**2032**		**2033**		**Not estimable**
Total events	0		0			
Heterogeneity: Not applicable						
Test for overall effect: Not applicable						
**Total (95% CI)**		**24954**		**24949**	**100.0%**	**0.90 [0.66, 1.22]**
Total events	76		84			
Heterogeneity: Not applicable						
Test for overall effect: Z = 0.67 (P = 0.51)						
Test for subgroup differences: Not applicable						

Risk Ratio
IV, Random, 95% CI

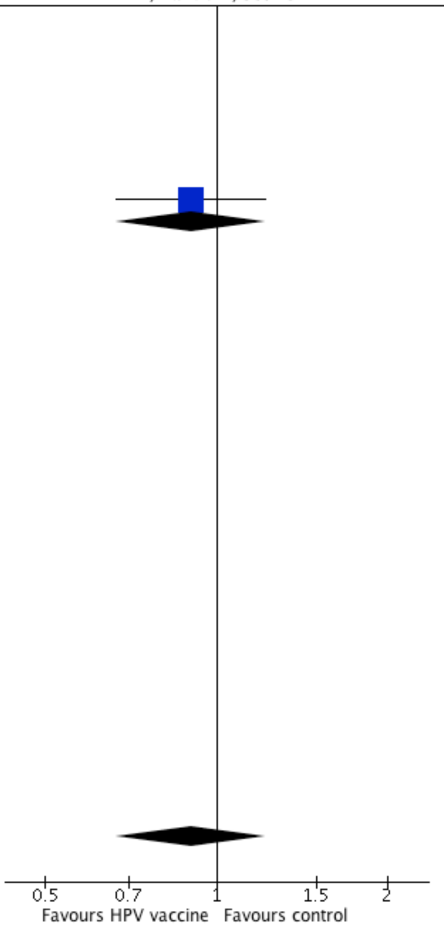

*2.7. Risk ratio for GlaxoSmithKline studies (i.e., HPV-0xx): 0.90 [0.66, 1.22]; risk ratio for Merck Sharp & Dohme studies (i.e., V50x-xxx): not applicable.

2.8. Fatal harms*: intention to treat analysis

Study or Subgroup	HPV vaccine Events	HPV vaccine Total	Control Events	Control Total	Weight	Risk Ratio IV, Random, 95% CI
**2.8.1 Fatal harms: Cervarix vs. Al(OH)3 (females, age 24-72; follow-up until 36 months)**						
HPV-015: Cervarix vs. Al(OH)3 (f, 24-72; 36)	13	2882	5	2871	21.5%	2.59 [0.92, 7.26]
**Subtotal (95% CI)**		**2882**		**2871**	**21.5%**	**2.59 [0.92, 7.26]**
Total events	13		5			
Heterogeneity: Not applicable						
Test for overall effect: Z = 1.81 (P = 0.07)						
**2.8.2 Fatal harms: Cervarix vs. Aimmugen: hepatitis A vaccine (females, age 19-25; follow-up until 7 months)**						
HPV-032: Cervarix vs. Aimmugen (f, 19-25; 7)	1	519	0	521	3.1%	3.01 [0.12, 73.76]
**Subtotal (95% CI)**		**519**		**521**	**3.1%**	**3.01 [0.12, 73.76]**
Total events	1		0			
Heterogeneity: Not applicable						
Test for overall effect: Z = 0.68 (P = 0.50)						
**2.8.3 Fatal harms: Cervarix vs. Engerix: hepatitis B vaccine (females, age 26-46; follow-up until 12 months)**						
HPV-069: Cervarix vs. Engerix (f, 26-46; 12)	0	606	0	606		Not estimable
**Subtotal (95% CI)**		**606**		**606**		**Not estimable**
Total events	0		0			
Heterogeneity: Not applicable						
Test for overall effect: Not applicable						
**2.8.4 Fatal harms: Cervarix vs. Engerix: hepatitis B vaccine (females and males, age 9-16; follow-up until 72 months)**						
HPV-040: Cervarix vs. Engerix (f & m, 12-16; 72)	5	14837	5	17338	16.4%	1.17 [0.34, 4.04]
**Subtotal (95% CI)**		**14837**		**17338**	**16.4%**	**1.17 [0.34, 4.04]**
Total events	5		5			
Heterogeneity: Not applicable						
Test for overall effect: Z = 0.25 (P = 0.81)						
**2.8.5 Fatal harms: Gardasil vs. AAHS (females, age 15-26; follow-up until 45 months)**						
V501-013: Gardasil vs. AAHS (f, 16-24; 45)	2	2723	2	2732	7.7%	1.00 [0.14, 7.12]
V501-015: Gardasil vs. AAHS (f, 15-26; 36)	7	6087	5	6080	18.5%	1.40 [0.44, 4.40]
**Subtotal (95% CI)**		**8810**		**8812**	**26.1%**	**1.28 [0.48, 3.46]**
Total events	9		7			
Heterogeneity: $Tau^2 = 0.00$; $Chi^2 = 0.08$, df = 1 (P = 0.77); $I^2 = 0\%$						
Test for overall effect: Z = 0.50 (P = 0.62)						
**2.8.6 Fatal harms: Gardasil vs. AAHS (females, age 21-46; follow-up until 48 months)**						
V501-019: Gardasil vs. AAHS (f, 21-46; 48)	8	1911	4	1908	17.3%	2.00 [0.60, 6.62]
**Subtotal (95% CI)**		**1911**		**1908**	**17.3%**	**2.00 [0.60, 6.62]**
Total events	8		4			
Heterogeneity: Not applicable						
Test for overall effect: Z = 1.13 (P = 0.26)						
**2.8.7 Fatal harms: Gardasil vs. AAHS (males, age 15-27; follow-up until 36 months)**						
V501-020: Gardasil vs. AAHS (m, 15-27; 36)	3	2032	10	2033	15.5%	0.30 [0.08, 1.09]
**Subtotal (95% CI)**		**2032**		**2033**	**15.5%**	**0.30 [0.08, 1.09]**
Total events	3		10			
Heterogeneity: Not applicable						
Test for overall effect: Z = 1.83 (P = 0.07)						
**2.8.8 Fatal harms: HPV 16 vaccine vs. AAHS (females, age 16-25; follow-up until 48 months)**						
V501-005: HPV 16 vaccine vs. AAHS (f, 16-25; 48)	0	1204	0	1205		Not estimable
**Subtotal (95% CI)**		**1204**		**1205**		**Not estimable**
Total events	0		0			
Heterogeneity: Not applicable						
Test for overall effect: Not applicable						
**Total (95% CI)**		**32801**		**35294**	**100.0%**	**1.30 [0.73, 2.30]**
Total events	39		31			
Heterogeneity: $Tau^2 = 0.12$; $Chi^2 = 7.55$, df = 6 (P = 0.27); $I^2 = 21\%$						
Test for overall effect: Z = 0.89 (P = 0.37)						
Test for subgroup differences: $Chi^2 = 7.47$, df = 5 (P = 0.19), $I^2 = 33.1\%$						

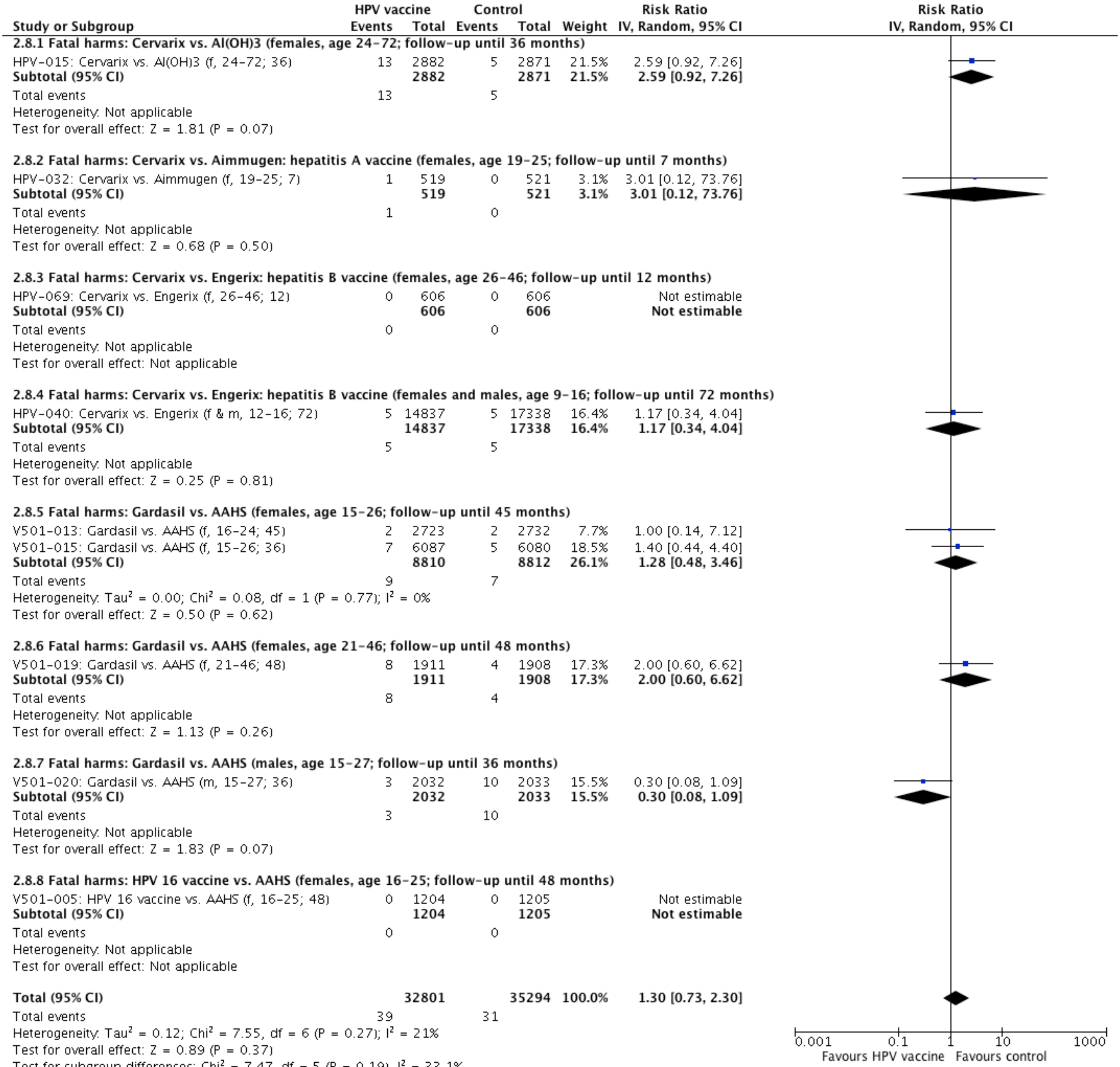

*2.8. Risk ratio for GlaxoSmithKline studies (i.e., HPV-0xx): 1.92 [0.89, 4.15]; risk ratio for Merck Sharp & Dohme studies (i.e., V50x-xxx): 0.98 [0.41, 2.33].

2.9. Serious harms*: intention to treat analysis

Study or Subgroup	HPV vaccine Events	HPV vaccine Total	Control Events	Control Total	Weight	Risk Ratio IV, Random, 95% CI
**2.9.1 Serious harms: Cervarix vs. Al(OH)3 (females, age 9-17; follow-up until 12 months)**						
HPV-058: Cervarix vs. Al(OH)3 (f, 9-17; 12)	5	374	3	376	0.3%	1.68 [0.40, 6.96]
**Subtotal (95% CI)**		**374**		**376**	**0.3%**	**1.68 [0.40, 6.96]**
Total events	5		3			
Heterogeneity: Not applicable						
Test for overall effect: Z = 0.71 (P = 0.48)						
**2.9.2 Serious harms: Cervarix vs. Al(OH)3 (females, age 15-25; follow-up until 7 months)**						
HPV-038: Cervarix vs. Al(OH)3 (f, 15-25; 7)	2	149	1	76	0.1%	1.02 [0.09, 11.07]
**Subtotal (95% CI)**		**149**		**76**	**0.1%**	**1.02 [0.09, 11.07]**
Total events	2		1			
Heterogeneity: Not applicable						
Test for overall effect: Z = 0.02 (P = 0.99)						
**2.9.3 Serious harms: Cervarix vs. Al(OH)3 (females, age 18-35; follow-up until 12 months)**						
HPV-003: Cervarix vs. Al(OH)3 (f, 18-30; 12)	0	31	0	30		Not estimable
HPV-023: Cervarix vs. Al(OH)3 (f, 21-32; 36)	20	223	11	213	1.0%	1.74 [0.85, 3.54]
HPV-031: Cervarix vs. Al(OH)3 (f, 18-35; 7)	2	176	4	178	0.2%	0.51 [0.09, 2.73]
HPV-035: Cervarix vs. Al(OH)3 (f, 18-35; 7)	0	150	0	150		Not estimable
**Subtotal (95% CI)**		**580**		**571**	**1.2%**	**1.20 [0.40, 3.63]**
Total events	22		15			
Heterogeneity: $Tau^2 = 0.33$; $Chi^2 = 1.75$, df = 1 (P = 0.19); $I^2 = 43\%$						
Test for overall effect: Z = 0.32 (P = 0.75)						
**2.9.4 Serious harms: Cervarix vs. Al(OH)3 (females, age 24-72; follow-up until 36 months)**						
HPV-015: Cervarix vs. Al(OH)3 (f, 24-72; 36)	291	2882	269	2871	21.0%	1.08 [0.92, 1.26]
**Subtotal (95% CI)**		**2882**		**2871**	**21.0%**	**1.08 [0.92, 1.26]**
Total events	291		269			
Heterogeneity: Not applicable						
Test for overall effect: Z = 0.93 (P = 0.35)						
**2.9.5 Serious harms: Cervarix vs. Aimmugen: hepatitis A vaccine (females, age 22-29; follow-up until 12 months)**						
HPV-032: Cervarix vs. Aimmugen (f, 19-25; 7)	18	519	19	521	1.3%	0.95 [0.50, 1.79]
HPV-063: Cervarix vs. Aimmugen (f, 22-29; 12)	11	375	16	377	0.9%	0.69 [0.33, 1.47]
**Subtotal (95% CI)**		**894**		**898**	**2.2%**	**0.83 [0.51, 1.35]**
Total events	29		35			
Heterogeneity: $Tau^2 = 0.00$; $Chi^2 = 0.40$, df = 1 (P = 0.53); $I^2 = 0\%$						
Test for overall effect: Z = 0.74 (P = 0.46)						
**2.9.6 Serious harms: Cervarix vs. Engerix: hepatitis B vaccine (females and males, age 9-16; follow-up until 72 months)**						
HPV-030: Cervarix vs. Engerix (f, 9-15; 12)	2	247	1	246	0.1%	1.99 [0.18, 21.82]
HPV-040: Cervarix vs. Engerix (f & m, 12-16; 72)	58	2436	25	1267	2.4%	1.21 [0.76, 1.92]
**Subtotal (95% CI)**		**2683**		**1513**	**2.5%**	**1.23 [0.78, 1.94]**
Total events	60		26			
Heterogeneity: $Tau^2 = 0.00$; $Chi^2 = 0.16$, df = 1 (P = 0.69); $I^2 = 0\%$						
Test for overall effect: Z = 0.89 (P = 0.38)						
**2.9.7 Serious harms: Cervarix vs. Engerix: hepatitis B vaccine (females, age 26-46; follow-up until 12 months)**						
HPV-069: Cervarix vs. Engerix (f, 26-46; 12)	3	606	3	606	0.2%	1.00 [0.20, 4.93]
**Subtotal (95% CI)**		**606**		**606**	**0.2%**	**1.00 [0.20, 4.93]**
Total events	3		3			
Heterogeneity: Not applicable						
Test for overall effect: Z = 0.00 (P = 1.00)						
**2.9.8 Serious harms: Cervarix vs. Havrix: hepatitis A vaccine (females, age 9-15; follow-up until 12 months)**						
HPV-013: Cervarix vs. Havrix (f, 10-14; 12)	0	1035	0	1032		Not estimable
HPV-033: Cervarix vs. Havrix (f, 9-15; 7)	0	160	0	161		Not estimable
**Subtotal (95% CI)**		**1195**		**1193**		**Not estimable**
Total events	0		0			
Heterogeneity: Not applicable						
Test for overall effect: Not applicable						
**2.9.9 Serious harms: Cervarix vs. Havrix: hepatitis A vaccine (females, age 15-25; follow-up until 48 months)**						
HPV-008: Cervarix vs. Havrix (f, 15-25; 48)	829	9319	835	9325	61.8%	0.99 [0.91, 1.09]
**Subtotal (95% CI)**		**9319**		**9325**	**61.8%**	**0.99 [0.91, 1.09]**
Total events	829		835			
Heterogeneity: Not applicable						
Test for overall effect: Z = 0.14 (P = 0.89)						
**2.9.10 Serious harms: Cervarix vs. Twinrix: hepatitis A and B vaccine (females, age 8-15; follow-up until 12 months)**						
HPV-029: Cervarix vs. Twinrix (f, 8-15; 12)	4	270	4	271	0.3%	1.00 [0.25, 3.97]
**Subtotal (95% CI)**		**270**		**271**	**0.3%**	**1.00 [0.25, 3.97]**
Total events	4		4			
Heterogeneity: Not applicable						
Test for overall effect: Z = 0.01 (P = 1.00)						
**2.9.11 Serious harms: Gardasil vs. carrier solution (females, age 9-16; follow-up until 18 months)**						
V501-018: Gardasil vs. carrier solu. (f&m,9-16;18)	6	1184	0	597	0.1%	6.56 [0.37, 116.26]
**Subtotal (95% CI)**		**1184**		**597**	**0.1%**	**6.56 [0.37, 116.26]**
Total events	6		0			
Heterogeneity: Not applicable						
Test for overall effect: Z = 1.28 (P = 0.20)						
**2.9.12 Serious harms: Gardasil vs. AAHS (females, age 15-26; follow-up until 45 months)**						
V501-013: Gardasil vs. AAHS (f, 16-24; 45)	50	2723	45	2732	3.3%	1.11 [0.75, 1.66]
V501-015: Gardasil vs. AAHS (f, 15-26; 36)	46	6087	56	6080	3.4%	0.82 [0.56, 1.21]
**Subtotal (95% CI)**		**8810**		**8812**	**6.7%**	**0.95 [0.71, 1.29]**
Total events	96		101			
Heterogeneity: $Tau^2 = 0.01$; $Chi^2 = 1.16$, df = 1 (P = 0.28); $I^2 = 14\%$						
Test for overall effect: Z = 0.31 (P = 0.75)						
**2.9.13 Serious harms: Gardasil vs. AAHS (females, age 21-46; follow-up until 48 months)**						
V501-019: Gardasil vs. AAHS (f, 21-46; 48)	21	1911	23	1908	1.5%	0.91 [0.51, 1.64]
**Subtotal (95% CI)**		**1911**		**1908**	**1.5%**	**0.91 [0.51, 1.64]**
Total events	21		23			
Heterogeneity: Not applicable						
Test for overall effect: Z = 0.31 (P = 0.76)						
**2.9.14 Serious harms: Gardasil vs. AAHS (males, age 15-27; follow-up until 36 months)**						
V501-020: Gardasil vs. AAHS (m, 15-27; 36)	8	2032	11	2033	0.6%	0.73 [0.29, 1.81]
**Subtotal (95% CI)**		**2032**		**2033**	**0.6%**	**0.73 [0.29, 1.81]**
Total events	8		11			
Heterogeneity: Not applicable						
Test for overall effect: Z = 0.69 (P = 0.49)						
**2.9.15 Serious harms: Gardasil 9 vs. saline placebo (females, age 12-26; follow-up until 7 months)**						
V503-006: Gardasil 9 vs. saline (f, 12-26;7)	3	618	3	306	0.2%	0.50 [0.10, 2.44]
**Subtotal (95% CI)**		**618**		**306**	**0.2%**	**0.50 [0.10, 2.44]**
Total events	3		3			
Heterogeneity: Not applicable						
Test for overall effect: Z = 0.86 (P = 0.39)						
**2.9.16 Serious harms: HPV 16 vaccine vs. AAHS (females, age 16-25; follow-up until 48 months)**						
V501-005: HPV 16 vaccine vs. AAHS (f, 16-25; 48)	19	1204	20	1205	1.3%	0.95 [0.51, 1.77]
**Subtotal (95% CI)**		**1204**		**1205**	**1.3%**	**0.95 [0.51, 1.77]**
Total events	19		20			
Heterogeneity: Not applicable						
Test for overall effect: Z = 0.16 (P = 0.87)						
**Total (95% CI)**		**34711**		**32561**	**100.0%**	**1.01 [0.94, 1.09]**
Total events	1398		1349			
Heterogeneity: $Tau^2 = 0.00$; $Chi^2 = 10.40$, df = 18 (P = 0.92); $I^2 = 0\%$						
Test for overall effect: Z = 0.30 (P = 0.77)						
Test for subgroup differences: $Chi^2 = 5.86$, df = 14 (P = 0.97), $I^2 = 0\%$						

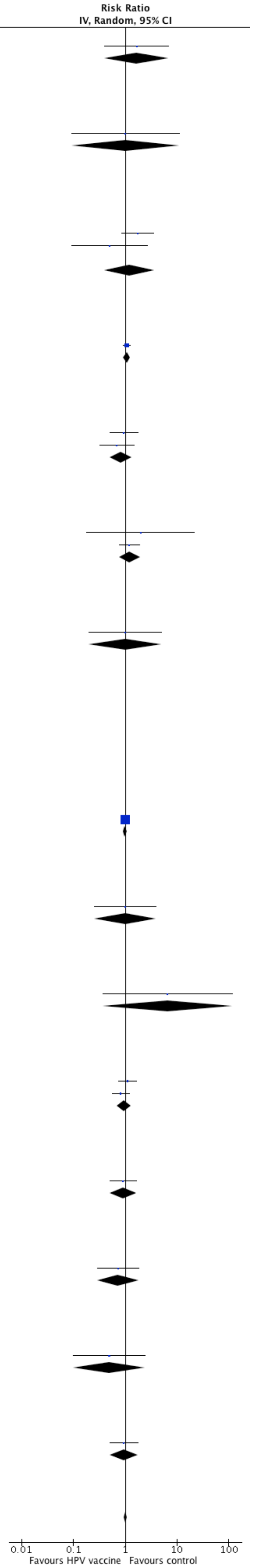

*2.9. Risk ratio for GlaxoSmithKline studies (i.e., HPV-0xx): 1.02 [0.95, 1.10]; risk ratio for Merck Sharp & Dohme studies (i.e., V50x-xxx): 0.93 [0.74, 1.16].

2.10. Serious harms judged as ‘definitely associated’* with chronic regional pain syndrome (CRPS): intention to treat analysis

Study or Subgroup	HPV vaccine Events	HPV vaccine Total	Control Events	Control Total	Weight	Risk Ratio IV, Random, 95% CI
**2.10.1 Serious harms 'definitely associated' with CRPS: Cervarix vs. Al(OH)3 (females, age 18-35; follow-up until 7 months)**						
HPV-035: Cervarix vs. Al(OH)3 (f, 18-35; 7)	0	150	0	150		Not estimable
**Subtotal (95% CI)**		**150**		**150**		**Not estimable**
Total events	0		0			
Heterogeneity: Not applicable						
Test for overall effect: Not applicable						
**2.10.2 Serious harms 'definitely associated' with CRPS: Cervarix vs. Al(OH)3 (females, age 24-72; follow-up until 36 months)**						
HPV-015: Cervarix vs. Al(OH)3 (f, 24-72; 36)	15	2882	9	2871	16.5%	1.66 [0.73, 3.79]
**Subtotal (95% CI)**		**2882**		**2871**	**16.5%**	**1.66 [0.73, 3.79]**
Total events	15		9			
Heterogeneity: Not applicable						
Test for overall effect: Z = 1.20 (P = 0.23)						
**2.10.3 Serious harms 'definitely associated' with CRPS: Cervarix vs. Aimmugen: hepatitis A vaccine (females, age 22-29; follow-up until 12 months)**						
HPV-063: Cervarix vs. Aimmugen (f, 22-29; 12)	0	375	1	377	1.1%	0.34 [0.01, 8.20]
**Subtotal (95% CI)**		**375**		**377**	**1.1%**	**0.34 [0.01, 8.20]**
Total events	0		1			
Heterogeneity: Not applicable						
Test for overall effect: Z = 0.67 (P = 0.50)						
**2.10.4 Serious harms 'definitely associated' with CRPS: Cervarix vs. Engerix: hepatitis B vaccine (females and males, age 9-16; follow-up until 72 months)**						
HPV-040: Cervarix vs. Engerix (f & m, 12-16; 72)	2	2436	0	1267	1.2%	2.60 [0.12, 54.15]
**Subtotal (95% CI)**		**2436**		**1267**	**1.2%**	**2.60 [0.12, 54.15]**
Total events	2		0			
Heterogeneity: Not applicable						
Test for overall effect: Z = 0.62 (P = 0.54)						
**2.10.5 Serious harms 'definitely associated' with CRPS: Cervarix vs. Havrix: hepatitis A vaccine (females, age 10-14; follow-up until 12 months)**						
HPV-013: Cervarix vs. Havrix (f, 10-14; 12)	0	1035	0	1032		Not estimable
**Subtotal (95% CI)**		**1035**		**1032**		**Not estimable**
Total events	0		0			
Heterogeneity: Not applicable						
Test for overall effect: Not applicable						
**2.10.6 Serious harms 'definitely associated' with CRPS: Cervarix vs. Havrix: hepatitis A vaccine (females, age 15-25; follow-up until 48 months)**						
HPV-008: Cervarix vs. Havrix (f, 15-25; 48)	59	9319	39	9325	69.2%	1.51 [1.01, 2.27]
**Subtotal (95% CI)**		**9319**		**9325**	**69.2%**	**1.51 [1.01, 2.27]**
Total events	59		39			
Heterogeneity: Not applicable						
Test for overall effect: Z = 2.01 (P = 0.04)						
**2.10.7 Serious harms 'definitely associated' with CRPS: Gardasil vs. carrier solution (females and males, age 9-16; follow-up until 18 months)**						
V501-018: Gardasil vs. carrier solu. (f&m,9-16;18)	1	1184	0	597	1.1%	1.51 [0.06, 37.11]
**Subtotal (95% CI)**		**1184**		**597**	**1.1%**	**1.51 [0.06, 37.11]**
Total events	1		0			
Heterogeneity: Not applicable						
Test for overall effect: Z = 0.25 (P = 0.80)						
**2.10.8 Serious harms 'definitely associated' with CRPS: Gardasil vs. AAHS (females, age 15-26; follow-up until 45 months)**						
V501-013: Gardasil vs. AAHS (f, 16-24; 45)	1	2723	2	2732	2.0%	0.50 [0.05, 5.53]
V501-015: Gardasil vs. AAHS (f, 15-26; 36)	4	6087	3	6080	5.0%	1.33 [0.30, 5.95]
**Subtotal (95% CI)**		**8810**		**8812**	**7.0%**	**1.01 [0.28, 3.61]**
Total events	5		5			
Heterogeneity: $Tau^2 = 0.00$; $Chi^2 = 0.46$, df = 1 (P = 0.50); $I^2 = 0\%$						
Test for overall effect: Z = 0.02 (P = 0.98)						
**2.10.9 Serious harms 'definitely associated' with CRPS: Gardasil vs. AAHS (females, age 21-46; follow-up until 48 months)**						
V501-019: Gardasil vs. AAHS (f, 21-46; 48)	4	1911	0	1908	1.3%	8.99 [0.48, 166.79]
**Subtotal (95% CI)**		**1911**		**1908**	**1.3%**	**8.99 [0.48, 166.79]**
Total events	4		0			
Heterogeneity: Not applicable						
Test for overall effect: Z = 1.47 (P = 0.14)						
**2.10.10 Serious harms 'definitely associated' with CRPS: Gardasil vs. AAHS (males, age 15-27; follow-up until 36 months)**						
V501-020: Gardasil vs. AAHS (m, 15-27; 36)	1	2032	0	2033	1.1%	3.00 [0.12, 73.64]
**Subtotal (95% CI)**		**2032**		**2033**	**1.1%**	**3.00 [0.12, 73.64]**
Total events	1		0			
Heterogeneity: Not applicable						
Test for overall effect: Z = 0.67 (P = 0.50)						
**2.10.11 Serious harms 'definitely associated' with CRPS: HPV 16 vaccine vs. AAHS (females, age 16-25; follow-up until 48 months)**						
V501-005: HPV 16 vaccine vs. AAHS (f, 16-25; 48)	1	1204	1	1205	1.5%	1.00 [0.06, 15.98]
**Subtotal (95% CI)**		**1204**		**1205**	**1.5%**	**1.00 [0.06, 15.98]**
Total events	1		1			
Heterogeneity: Not applicable						
Test for overall effect: Z = 0.00 (P = 1.00)						
**Total (95% CI)**		**31338**		**29577**	**100.0%**	**1.52 [1.08, 2.12]**
Total events	88		55			
Heterogeneity: $Tau^2 = 0.00$; $Chi^2 = 3.56$, df = 9 (P = 0.94); $I^2 = 0\%$						
Test for overall effect: Z = 2.43 (P = 0.01)						
Test for subgroup differences: $Chi^2 = 3.10$, df = 8 (P = 0.93), $I^2 = 0\%$						

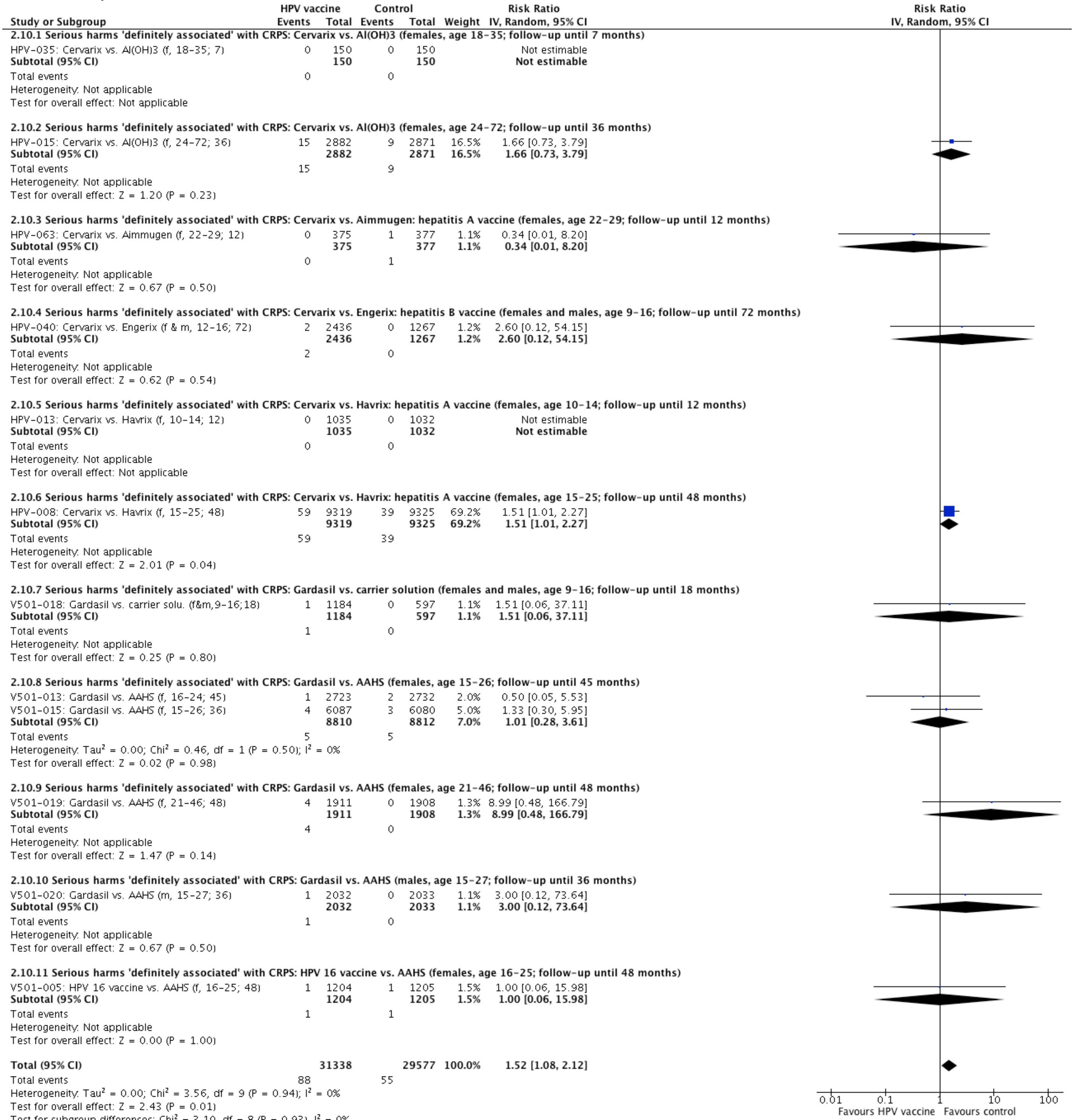

*2.10. Risk ratio for GlaxoSmithKline studies (i.e., HPV-0xx): **1.52 [1.07, 2.18]**; risk ratio for Merck Sharp & Dohme studies (i.e., V50x-xxx): 1.48 [0.56, 3.89]. We asked a physician with clinical expertise in CRPS to assess the reported MedDRA preferred terms as 'definitely,' 'probably,' 'probably not' or 'definitely not' associated with CRPS. We sent an Excel sheet to the physician with all the reported MedDRA terms. The physician was blinded, as the Excel sheet contained no outcome data. When the physician had assessed all the MedDRA terms, we synthesized the data for those MedDRA terms that the physician judged 'definitely' associated with CRPS and compared it to the reported serious harms.

2.11. Serious harms judged as ‘definitely associated’* with postural orthostatic tachycardia syndrome (POTS): intention to treat analysis

Study or Subgroup	HPV vaccine Events	HPV vaccine Total	Control Events	Control Total	Weight	Risk Ratio IV, Random, 95% CI
**2.11.1 Serious harms 'definitely associated' with POTS: Cervarix vs. Al(OH)3 (females, age 18-35; follow-up until 7 months)**						
HPV-035: Cervarix vs. Al(OH)3 (f, 18-35; 7)	0	150	0	150		Not estimable
**Subtotal (95% CI)**		**150**		**150**		**Not estimable**
Total events	0		0			
Heterogeneity: Not applicable						
Test for overall effect: Not applicable						
**2.11.2 Serious harms 'definitely associated' with POTS: Cervarix vs. Al(OH)3 (females, age 24-72; follow-up until 36 months)**						
HPV-015: Cervarix vs. Al(OH)3 (f, 24-72; 36)	9	2882	5	2871	19.7%	1.79 [0.60, 5.34]
**Subtotal (95% CI)**		**2882**		**2871**	**19.7%**	**1.79 [0.60, 5.34]**
Total events	9		5			
Heterogeneity: Not applicable						
Test for overall effect: Z = 1.05 (P = 0.29)						
**2.11.3 Serious harms 'definitely associated' with POTS: Cervarix vs. Aimmugen: hepatitis A vaccine (females, age 22-29; follow-up until 12 months)**						
HPV-063: Cervarix vs. Aimmugen (f, 22-29; 12)	0	375	1	377	2.3%	0.34 [0.01, 8.20]
**Subtotal (95% CI)**		**375**		**377**	**2.3%**	**0.34 [0.01, 8.20]**
Total events	0		1			
Heterogeneity: Not applicable						
Test for overall effect: Z = 0.67 (P = 0.50)						
**2.11.4 Serious harms 'definitely associated' with POTS: Cervarix vs. Engerix: hepatitis B vaccine (females and males, age 9-16; follow-up until 72 months)**						
HPV-040: Cervarix vs. Engerix (f & m, 12-16; 72)	2	2436	0	1267	2.5%	2.60 [0.12, 54.15]
**Subtotal (95% CI)**		**2436**		**1267**	**2.5%**	**2.60 [0.12, 54.15]**
Total events	2		0			
Heterogeneity: Not applicable						
Test for overall effect: Z = 0.62 (P = 0.54)						
**2.11.5 Serious harms 'definitely associated' with POTS: Cervarix vs. Havrix: hepatitis A vaccine (females, age 10-14; follow-up until 12 months)**						
HPV-013: Cervarix vs. Havrix (f, 10-14; 12)	0	1035	0	1032		Not estimable
**Subtotal (95% CI)**		**1035**		**1032**		**Not estimable**
Total events	0		0			
Heterogeneity: Not applicable						
Test for overall effect: Not applicable						
**2.11.6 Serious harms 'definitely associated' with POTS: Cervarix vs. Havrix: hepatitis A vaccine (females, age 15-25; follow-up until 48 months)**						
HPV-008: Cervarix vs. Havrix (f, 15-25; 48)	28	9319	12	9325	51.4%	2.33 [1.19, 4.59]
**Subtotal (95% CI)**		**9319**		**9325**	**51.4%**	**2.33 [1.19, 4.59]**
Total events	28		12			
Heterogeneity: Not applicable						
Test for overall effect: Z = 2.46 (P = 0.01)						
**2.11.7 Serious harms 'definitely associated' with POTS: Gardasil vs. AAHS (females, age 15-26; follow-up until 45 months)**						
V501-013: Gardasil vs. AAHS (f, 16-24; 45)	3	2723	3	2732	9.2%	1.00 [0.20, 4.97]
V501-015: Gardasil vs. AAHS (f, 15-26; 36)	3	6087	1	6080	4.6%	3.00 [0.31, 28.80]
**Subtotal (95% CI)**		**8810**		**8812**	**13.7%**	**1.44 [0.39, 5.33]**
Total events	6		4			
Heterogeneity: Tau² = 0.00; Chi² = 0.60, df = 1 (P = 0.44); I² = 0%						
Test for overall effect: Z = 0.55 (P = 0.58)						
**2.11.8 Serious harms 'definitely associated' with POTS: Gardasil vs. AAHS (females, age 21-46; follow-up until 48 months)**						
V501-019: Gardasil vs. AAHS (f, 21-46; 48)	4	1911	0	1908	2.7%	8.99 [0.48, 166.79]
**Subtotal (95% CI)**		**1911**		**1908**	**2.7%**	**8.99 [0.48, 166.79]**
Total events	4		0			
Heterogeneity: Not applicable						
Test for overall effect: Z = 1.47 (P = 0.14)						
**2.11.9 Serious harms 'definitely associated' with POTS: Gardasil vs. AAHS (males, age 15-27; follow-up until 36 months)**						
V501-020: Gardasil vs. AAHS (m, 15-27; 36)	1	2032	0	2033	2.3%	3.00 [0.12, 73.64]
**Subtotal (95% CI)**		**2032**		**2033**	**2.3%**	**3.00 [0.12, 73.64]**
Total events	1		0			
Heterogeneity: Not applicable						
Test for overall effect: Z = 0.67 (P = 0.50)						
**2.11.10 Serious harms 'definitely associated' with POTS: Gardasil 9 vs. saline placebo (females, age 12-26; follow-up until 7 months)**						
V503-006: Gardasil 9 vs. saline (f, 12-26;7)	1	618	0	306	2.3%	1.49 [0.06, 36.42]
**Subtotal (95% CI)**		**618**		**306**	**2.3%**	**1.49 [0.06, 36.42]**
Total events	1		0			
Heterogeneity: Not applicable						
Test for overall effect: Z = 0.24 (P = 0.81)						
**2.11.11 Serious harms 'definitely associated' with POTS: HPV 16 vaccine vs. AAHS (females, age 16-25; follow-up until 48 months)**						
V501-005: HPV 16 vaccine vs. AAHS (f, 16-25; 48)	1	1204	1	1205	3.1%	1.00 [0.06, 15.98]
**Subtotal (95% CI)**		**1204**		**1205**	**3.1%**	**1.00 [0.06, 15.98]**
Total events	1		1			
Heterogeneity: Not applicable						
Test for overall effect: Z = 0.00 (P = 1.00)						
**Total (95% CI)**		**30772**		**29286**	**100.0%**	**2.00 [1.23, 3.25]**
Total events	52		23			
Heterogeneity: Tau² = 0.00; Chi² = 3.66, df = 9 (P = 0.93); I² = 0%						
Test for overall effect: Z = 2.81 (P = 0.005)						
Test for subgroup differences: Chi² = 3.06, df = 8 (P = 0.93), I² = 0%						

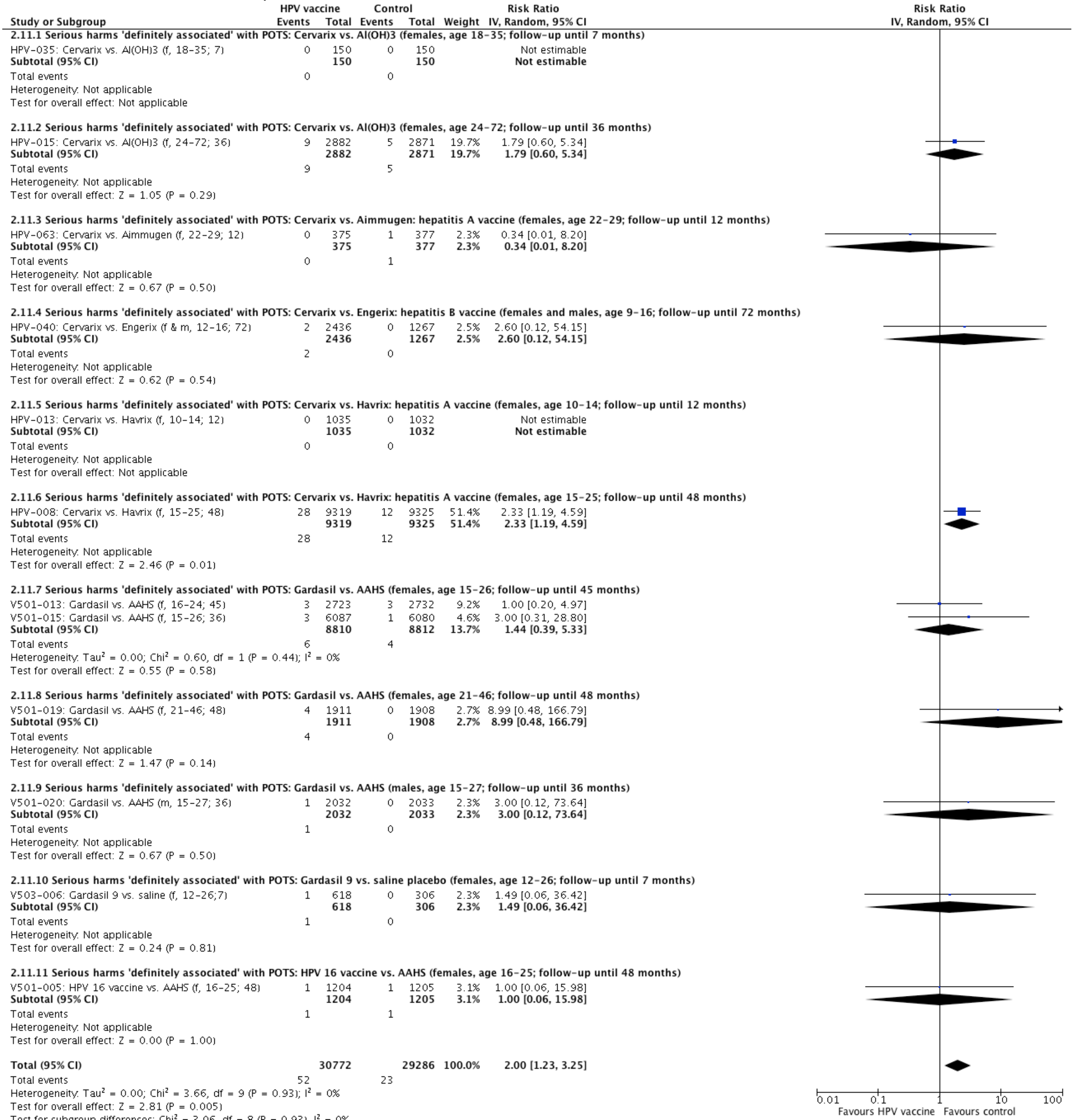

*2.11. Risk ratio for GlaxoSmithKline studies (i.e., HPV-0xx): **2.06 [1.18, 3.60]**; risk ratio for Merck Sharp & Dohme studies (i.e., V50x-xxx): 1.83 [0.68, 4.89]. We asked a physician with clinical expertise in POTS to assess the reported MedDRA preferred terms as 'definitely,' 'probably,' 'probably not' or 'definitely not' associated with POTS. We sent an Excel sheet to the physician with all the reported MedDRA terms. The physician was blinded, as the Excel sheet contained no outcome data. When the physician had assessed all the MedDRA terms, we synthesized the data for those MedDRA terms that the physician judged 'definitely' associated with POTS and compared it to the reported serious harms.

2.12. Serious harms reported within the MedDRA system organ class 'nervous system disorders (10029205)'*: intention to treat analysis

Study or Subgroup	HPV vaccine Events	HPV vaccine Total	Control Events	Control Total	Weight	Risk Ratio IV, Random, 95% CI
**2.12.1 Serious nervous system disorders: Cervarix vs. Al(OH)3 (females, age 18–35; follow-up until 12 months)**						
HPV-003: Cervarix vs. Al(OH)3 (f, 18–30; 12)	0	31	0	30		Not estimable
HPV-035: Cervarix vs. Al(OH)3 (f, 18–35; 7)	0	150	0	150		Not estimable
**Subtotal (95% CI)**		**181**		**180**		**Not estimable**
Total events	0		0			
Heterogeneity: Not applicable						
Test for overall effect: Not applicable						
**2.12.2 Serious nervous system disorders: Cervarix vs. Al(OH)3 (females, age 24–72; follow-up until 36 months)**						
HPV-015: Cervarix vs. Al(OH)3 (f, 24–72; 36)	15	2882	11	2871	23.9%	1.36 [0.63, 2.95]
**Subtotal (95% CI)**		**2882**		**2871**	**23.9%**	**1.36 [0.63, 2.95]**
Total events	15		11			
Heterogeneity: Not applicable						
Test for overall effect: Z = 0.77 (P = 0.44)						
**2.12.3 Serious nervous system disorders: Cervarix vs. Aimmugen: hepatitis A vaccine (females, age 22–29; follow-up until 12 months)**						
HPV-032: Cervarix vs. Aimmugen (f, 19–25; 7)	0	519	1	521	1.4%	0.33 [0.01, 8.20]
HPV-063: Cervarix vs. Aimmugen (f, 22–29; 12)	1	375	1	377	1.9%	1.01 [0.06, 16.01]
**Subtotal (95% CI)**		**894**		**898**	**3.3%**	**0.63 [0.08, 5.09]**
Total events	1		2			
Heterogeneity: Tau² = 0.00; Chi² = 0.26, df = 1 (P = 0.61); I² = 0%						
Test for overall effect: Z = 0.44 (P = 0.66)						
**2.12.4 Serious nervous system disorders: Cervarix vs. Engerix: hepatitis B vaccine (females and males, age 12–16; follow-up until 72 months)**						
HPV-040: Cervarix vs. Engerix (f & m, 12–16; 72)	2	2436	0	1267	1.6%	2.60 [0.12, 54.15]
**Subtotal (95% CI)**		**2436**		**1267**	**1.6%**	**2.60 [0.12, 54.15]**
Total events	2		0			
Heterogeneity: Not applicable						
Test for overall effect: Z = 0.62 (P = 0.54)						
**2.12.5 Serious nervous system disorders: Cervarix vs. Engerix: hepatitis B vaccine (females, age 26–46; follow-up until 12 months)**						
HPV-069: Cervarix vs. Engerix (f, 26–46; 12)	1	606	0	606	1.4%	3.00 [0.12, 73.50]
**Subtotal (95% CI)**		**606**		**606**	**1.4%**	**3.00 [0.12, 73.50]**
Total events	1		0			
Heterogeneity: Not applicable						
Test for overall effect: Z = 0.67 (P = 0.50)						
**2.12.6 Serious nervous system disorders: Cervarix vs. Havrix: hepatitis A vaccine (females, age 10–14; follow-up until 12 months)**						
HPV-013: Cervarix vs. Havrix (f, 10–14; 12)	0	1035	0	1032		Not estimable
**Subtotal (95% CI)**		**1035**		**1032**		**Not estimable**
Total events	0		0			
Heterogeneity: Not applicable						
Test for overall effect: Not applicable						
**2.12.7 Serious nervous system disorders: Cervarix vs. Havrix: hepatitis A vaccine (females, age 15–25; follow-up until 48 months)**						
HPV-008: Cervarix vs. Havrix (f, 15–25; 48)	39	9319	25	9325	57.3%	1.56 [0.95, 2.58]
**Subtotal (95% CI)**		**9319**		**9325**	**57.3%**	**1.56 [0.95, 2.58]**
Total events	39		25			
Heterogeneity: Not applicable						
Test for overall effect: Z = 1.74 (P = 0.08)						
**2.12.8 Serious nervous system disorders: Gardasil vs. AAHS (females, age 15–26; follow-up until 45 months)**						
V501-013: Gardasil vs. AAHS (f, 16–24; 45)	1	2723	5	2732	3.1%	0.20 [0.02, 1.72]
V501-015: Gardasil vs. AAHS (f, 15–26; 36)	4	6087	1	6080	3.0%	4.00 [0.45, 35.74]
**Subtotal (95% CI)**		**8810**		**8812**	**6.1%**	**0.89 [0.05, 16.65]**
Total events	5		6			
Heterogeneity: Tau² = 3.25; Chi² = 3.65, df = 1 (P = 0.06); I² = 73%						
Test for overall effect: Z = 0.08 (P = 0.94)						
**2.12.9 Serious nervous system disorders: Gardasil vs. AAHS (females, age 21–46; follow-up until 48 months)**						
V501-019: Gardasil vs. AAHS (f, 21–46; 48)	3	1911	0	1908	1.6%	6.99 [0.36, 135.21]
**Subtotal (95% CI)**		**1911**		**1908**	**1.6%**	**6.99 [0.36, 135.21]**
Total events	3		0			
Heterogeneity: Not applicable						
Test for overall effect: Z = 1.29 (P = 0.20)						
**2.12.10 Serious nervous system disorders: Gardasil vs. AAHS (males, age 15–27; follow-up until 36 months)**						
V501-020: Gardasil vs. AAHS (m, 15–27; 36)	1	2032	0	2033	1.4%	3.00 [0.12, 73.64]
**Subtotal (95% CI)**		**2032**		**2033**	**1.4%**	**3.00 [0.12, 73.64]**
Total events	1		0			
Heterogeneity: Not applicable						
Test for overall effect: Z = 0.67 (P = 0.50)						
**2.12.11 Serious nervous system disorders: Gardasil 9 vs. saline placebo (females, age 12–26; follow-up until 7 months)**						
V503-006: Gardasil 9 vs. saline (f, 12–26;7)	1	618	1	306	1.9%	0.50 [0.03, 7.89]
**Subtotal (95% CI)**		**618**		**306**	**1.9%**	**0.50 [0.03, 7.89]**
Total events	1		1			
Heterogeneity: Not applicable						
Test for overall effect: Z = 0.50 (P = 0.62)						
**2.12.12 Serious nervous system disorders: HPV 16 vaccine vs. AAHS (females, age 16–25; follow-up until 48 months)**						
V501-005: HPV 16 vaccine vs. AAHS (f, 16–25; 48)	1	1204	0	1205	1.4%	3.00 [0.12, 73.63]
**Subtotal (95% CI)**		**1204**		**1205**	**1.4%**	**3.00 [0.12, 73.63]**
Total events	1		0			
Heterogeneity: Not applicable						
Test for overall effect: Z = 0.67 (P = 0.50)						
**Total (95% CI)**		**31928**		**30443**	**100.0%**	**1.47 [1.01, 2.15]**
Total events	69		45			
Heterogeneity: Tau² = 0.00; Chi² = 7.46, df = 11 (P = 0.76); I² = 0%						
Test for overall effect: Z = 1.99 (P = 0.05)						
Test for subgroup differences: Chi² = 3.20, df = 9 (P = 0.96), I² = 0%						

Risk Ratio IV, Random, 95% CI

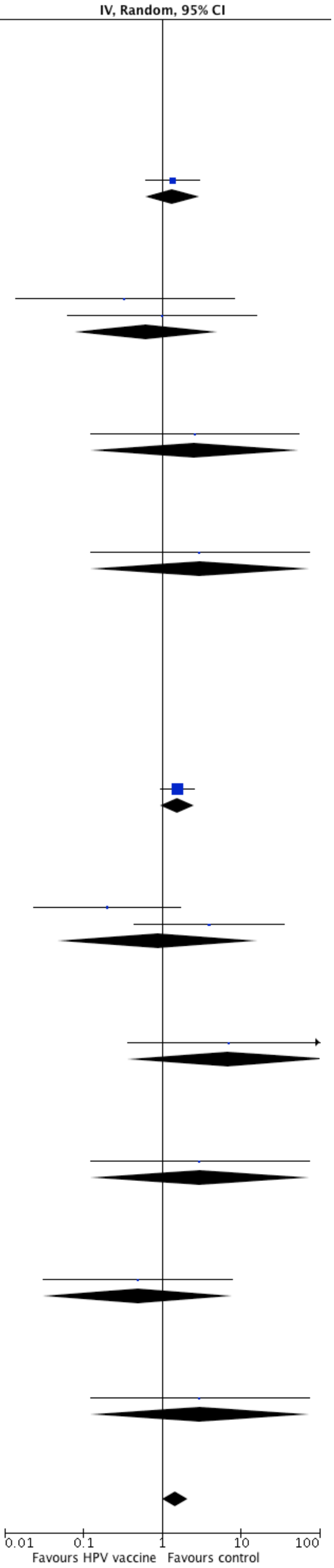

*2.12. Risk ratio for GlaxoSmithKline studies (i.e., HPV-0xx): 1.48 [0.99, 2.22]; risk ratio for Merck Sharp & Dohme studies (i.e., V50x-xxx): 1.45 [0.43, 4.82].

2.13. New onset diseases ('medically significant conditions' and 'new medical history'*): intention to treat analysis

Study or Subgroup	HPV vaccine Events	HPV vaccine Total	Control Events	Control Total	Weight	Risk Ratio IV, Random, 95% CI
**2.13.1 New onset diseases ('medically significant conditions'): Cervarix vs. Al(OH)3 (females, age 9-17; follow-up until 12 months)**						
HPV-058: Cervarix vs. Al(OH)3 (f, 9-17; 12)	14	374	11	376	0.9%	1.28 [0.59, 2.78]
**Subtotal (95% CI)**		**374**		**376**	**0.9%**	**1.28 [0.59, 2.78]**
Total events	14		11			
Heterogeneity: Not applicable						
Test for overall effect: Z = 0.62 (P = 0.53)						
**2.13.2 New onset diseases ('medically significant conditions'): Cervarix vs. Al(OH)3 (females, age 15-25; follow-up until 7 months)**						
HPV-038: Cervarix vs. Al(OH)3 (f, 15-25; 7)	34	149	10	76	1.3%	1.73 [0.91, 3.32]
**Subtotal (95% CI)**		**149**		**76**	**1.3%**	**1.73 [0.91, 3.32]**
Total events	34		10			
Heterogeneity: Not applicable						
Test for overall effect: Z = 1.66 (P = 0.10)						
**2.13.3 New onset diseases ('medically significant conditions'): Cervarix vs. Al(OH)3 (females, age 18-35; follow-up until 7 months)**						
HPV-023: Cervarix vs. Al(OH)3 (f, 21-32; 36)	60	223	38	213	3.8%	1.51 [1.05, 2.16]
HPV-031: Cervarix vs. Al(OH)3 (f, 18-35; 7)	13	176	24	178	1.3%	0.55 [0.29, 1.04]
HPV-035: Cervarix vs. Al(OH)3 (f, 18-35; 7)	0	150	0	150		Not estimable
**Subtotal (95% CI)**		**549**		**541**	**5.1%**	**0.94 [0.35, 2.54]**
Total events	73		62			
Heterogeneity: Tau² = 0.44; Chi² = 7.27, df = 1 (P = 0.007); I² = 86%						
Test for overall effect: Z = 0.12 (P = 0.91)						
**2.13.4 New onset diseases ('medically significant conditions'): Cervarix vs. Al(OH)3 (females, age 24-72; follow-up until 36 months)**						
HPV-015: Cervarix vs. Al(OH)3 (f, 24-72; 36)	1170	2882	1140	2871	34.8%	1.02 [0.96, 1.09]
**Subtotal (95% CI)**		**2882**		**2871**	**34.8%**	**1.02 [0.96, 1.09]**
Total events	1170		1140			
Heterogeneity: Not applicable						
Test for overall effect: Z = 0.69 (P = 0.49)						
**2.13.5 New onset diseases ('medically significant conditions'): Cervarix vs. Aimmugen: hepatitis A vaccine (females, age 22-29; follow-up until 12 months)**						
HPV-032: Cervarix vs. Aimmugen (f, 19-25; 7)	91	519	107	521	7.3%	0.85 [0.66, 1.10]
HPV-063: Cervarix vs. Aimmugen (f, 22-29; 12)	11	375	15	377	0.9%	0.74 [0.34, 1.58]
**Subtotal (95% CI)**		**894**		**898**	**8.2%**	**0.84 [0.66, 1.07]**
Total events	102		122			
Heterogeneity: Tau² = 0.00; Chi² = 0.13, df = 1 (P = 0.72); I² = 0%						
Test for overall effect: Z = 1.41 (P = 0.16)						
**2.13.6 New onset diseases ('medically significant conditions'): Cervarix vs. Engerix: hepatitis B vaccine (females and males, age 9-15; follow-up until 12 months)**						
HPV-030: Cervarix vs. Engerix (f, 9-15; 12)	28	247	22	246	1.9%	1.27 [0.75, 2.15]
HPV-040: Cervarix vs. Engerix (f & m, 12-16; 72)	47	643	76	1047	4.1%	1.01 [0.71, 1.43]
**Subtotal (95% CI)**		**890**		**1293**	**5.9%**	**1.08 [0.81, 1.45]**
Total events	75		98			
Heterogeneity: Tau² = 0.00; Chi² = 0.50, df = 1 (P = 0.48); I² = 0%						
Test for overall effect: Z = 0.52 (P = 0.61)						
**2.13.7 New onset diseases ('medically significant conditions'): Cervarix vs. Engerix: hepatitis B vaccine (females, age 26-46; follow-up until 12 months)**						
HPV-069: Cervarix vs. Engerix (f, 26-46; 12)	5	606	7	606	0.4%	0.71 [0.23, 2.24]
**Subtotal (95% CI)**		**606**		**606**	**0.4%**	**0.71 [0.23, 2.24]**
Total events	5		7			
Heterogeneity: Not applicable						
Test for overall effect: Z = 0.58 (P = 0.56)						
**2.13.8 New onset diseases ('medically significant conditions'): Cervarix vs. Havrix: hepatitis A vaccine (females, age 9-15; follow-up until 12 months)**						
HPV-013: Cervarix vs. Havrix (f, 10-14; 12)	0	1035	0	1032		Not estimable
HPV-033: Cervarix vs. Havrix (f, 9-15; 7)	0	160	0	161		Not estimable
**Subtotal (95% CI)**		**1195**		**1193**		**Not estimable**
Total events	0		0			
Heterogeneity: Not applicable						
Test for overall effect: Not applicable						
**2.13.9 New onset diseases ('medically significant conditions'): Cervarix vs. Havrix: hepatitis A vaccine (females, age 15-25; follow-up until 48 months)**						
HPV-008: Cervarix vs. Havrix (f, 15-25; 48)	3378	9319	3298	9325	41.4%	1.02 [0.99, 1.07]
**Subtotal (95% CI)**		**9319**		**9325**	**41.4%**	**1.02 [0.99, 1.07]**
Total events	3378		3298			
Heterogeneity: Not applicable						
Test for overall effect: Z = 1.25 (P = 0.21)						
**2.13.10 New onset diseases ('medically significant conditions'): Cervarix vs. Twinrix: hepatitis A and B vaccine (females, age 8-15; follow-up until 12 months)**						
HPV-029: Cervarix vs. Twinrix (f, 8-15; 12)	23	270	31	271	2.0%	0.74 [0.45, 1.24]
**Subtotal (95% CI)**		**270**		**271**	**2.0%**	**0.74 [0.45, 1.24]**
Total events	23		31			
Heterogeneity: Not applicable						
Test for overall effect: Z = 1.13 (P = 0.26)						
**2.13.11 New onset diseases ('new medical history'): Gardasil vs. carrier solution (females and males, age 9-16; follow-up until 18 months)**						
V501-018: Gardasil vs. carrier solu. (f&m,9-16;18)	0	1184	0	597		Not estimable
**Subtotal (95% CI)**		**1184**		**597**		**Not estimable**
Total events	0		0			
Heterogeneity: Not applicable						
Test for overall effect: Not applicable						
**2.13.12 New onset diseases ('new medical history'): Gardasil vs. AAHS (females, age 15-26; follow-up until 45 months)**						
V501-013: Gardasil vs. AAHS (f, 16-24; 45)	0	2723	0	2732		Not estimable
V501-015: Gardasil vs. AAHS (f, 15-26; 36)	0	6087	0	6080		Not estimable
**Subtotal (95% CI)**		**8810**		**8812**		**Not estimable**
Total events	0		0			
Heterogeneity: Not applicable						
Test for overall effect: Not applicable						
**2.13.13 New onset diseases ('new medical history'): Gardasil vs. AAHS (females, age 21-46; follow-up until 48 months)**						
V501-019: Gardasil vs. AAHS (f, 21-46; 48)	0	1911	0	1908		Not estimable
**Subtotal (95% CI)**		**1911**		**1908**		**Not estimable**
Total events	0		0			
Heterogeneity: Not applicable						
Test for overall effect: Not applicable						
**2.13.14 New onset diseases ('new medical history'): Gardasil vs. AAHS (males, age 15-27; follow-up until 36 months)**						
V501-020: Gardasil vs. AAHS (m, 15-27; 36)	0	2032	0	2032		Not estimable
**Subtotal (95% CI)**		**2032**		**2032**		**Not estimable**
Total events	0		0			
Heterogeneity: Not applicable						
Test for overall effect: Not applicable						
**2.13.15 New onset diseases ('new medical history'): Gardasil 9 vs. saline placebo (females, age 12-26; follow-up until 7 months)**						
V503-006: Gardasil 9 vs. saline (f, 12-26;7)	0	618	0	306		Not estimable
**Subtotal (95% CI)**		**618**		**306**		**Not estimable**
Total events	0		0			
Heterogeneity: Not applicable						
Test for overall effect: Not applicable						
**2.13.16 New onset diseases ('new medical history'): HPV 16 vaccine vs. AAHS (females, age 16-25; follow-up until 48 months)**						
V501-005: HPV 16 vaccine vs. AAHS (f, 16-25; 48)	0	1204	0	1205		Not estimable
**Subtotal (95% CI)**		**1204**		**1205**		**Not estimable**
Total events	0		0			
Heterogeneity: Not applicable						
Test for overall effect: Not applicable						
**Total (95% CI)**		**32887**		**32310**	**100.0%**	**1.02 [0.95, 1.10]**
Total events	4874		4779			
Heterogeneity: Tau² = 0.00; Chi² = 16.14, df = 11 (P = 0.14); I² = 32%						
Test for overall effect: Z = 0.49 (P = 0.63)						
Test for subgroup differences: Chi² = 7.44, df = 8 (P = 0.49), I² = 0%						

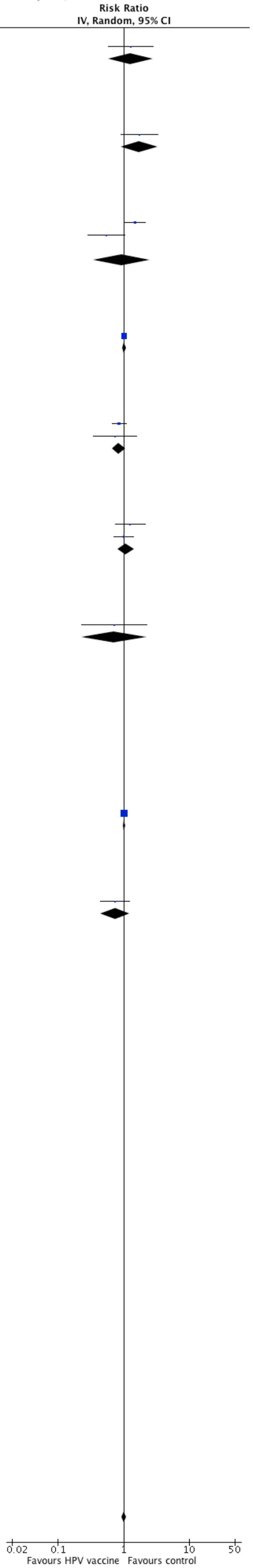

*2.13. Risk ratio for 'medically significant conditions' (GlaxoSmithKline): 1.02 [0.95, 1.10]; risk ratio for 'new medical history' (Merck Sharp & Dohme): not applicable.

2.14. New onset diseases most associated with the HPV vaccines (‘medically significant conditions’*) - ‘back pain’: intention to treat analysis

Study or Subgroup	HPV vaccine Events	HPV vaccine Total	Control Events	Control Total	Weight	Risk Ratio IV, Random, 95% CI
**2.14.1 New onset back pain ('medically significant conditions'): Cervarix vs. Al(OH)3 (females, age 18-35; follow-up until 12 months)**						
HPV-031: Cervarix vs. Al(OH)3 (f, 18-35; 7)	0	176	0	178		Not estimable
HPV-035: Cervarix vs. Al(OH)3 (f, 18-35; 7)	0	150	0	150		Not estimable
**Subtotal (95% CI)**		**326**		**328**		**Not estimable**
Total events	0		0			
Heterogeneity: Not applicable						
Test for overall effect: Not applicable						
**2.14.2 New onset back pain ('medically significant conditions'): Cervarix vs. Al(OH)3 (females, age 24-72; follow-up until 36 months)**						
HPV-015: Cervarix vs. Al(OH)3 (f, 24-72; 36)	0	2882	0	2871		Not estimable
**Subtotal (95% CI)**		**2882**		**2871**		**Not estimable**
Total events	0		0			
Heterogeneity: Not applicable						
Test for overall effect: Not applicable						
**2.14.3 New onset back pain ('medically significant conditions'): Cervarix vs. Aimmugen: hepatitis A vaccine (females, age 22-29; follow-up until 12 months)**						
HPV-063: Cervarix vs. Aimmugen (f, 22-29; 12)	0	375	0	377		Not estimable
**Subtotal (95% CI)**		**375**		**377**		**Not estimable**
Total events	0		0			
Heterogeneity: Not applicable						
Test for overall effect: Not applicable						
**2.14.4 New onset back pain ('medically significant conditions'): Cervarix vs. Havrix: hepatitis A vaccine (females, age 10-14; follow-up until 12 months)**						
HPV-013: Cervarix vs. Havrix (f, 10-14; 12)	0	1035	0	1032		Not estimable
**Subtotal (95% CI)**		**1035**		**1032**		**Not estimable**
Total events	0		0			
Heterogeneity: Not applicable						
Test for overall effect: Not applicable						
**2.14.5 New onset back pain ('medically significant conditions'): Cervarix vs. Havrix: hepatitis A vaccine (females, age 15-25; follow-up until 48 months)**						
HPV-008: Cervarix vs. Havrix (f, 15-25; 48)	0	9319	0	9325		Not estimable
**Subtotal (95% CI)**		**9319**		**9325**		**Not estimable**
Total events	0		0			
Heterogeneity: Not applicable						
Test for overall effect: Not applicable						
**2.14.6 New onset back pain ('new medical history'): Gardasil vs. carrier solution (females and males, age 9-16; follow-up until 18 months)**						
V501-018: Gardasil vs. carrier solu. (f&m,9-16;18)	0	1184	0	597		Not estimable
**Subtotal (95% CI)**		**1184**		**597**		**Not estimable**
Total events	0		0			
Heterogeneity: Not applicable						
Test for overall effect: Not applicable						
**2.14.7 New onset back pain ('new medical history'): Gardasil vs. AAHS (females, age 15-26; follow-up until 45 months)**						
V501-013: Gardasil vs. AAHS (f, 16-24; 45)	68	2723	63	2732	100.0%	1.08 [0.77, 1.52]
V501-015: Gardasil vs. AAHS (f, 15-26; 36)	0	6087	0	6080		Not estimable
**Subtotal (95% CI)**		**8810**		**8812**	**100.0%**	**1.08 [0.77, 1.52]**
Total events	68		63			
Heterogeneity: Not applicable						
Test for overall effect: Z = 0.46 (P = 0.64)						
**2.14.8 New onset back pain ('new medical history'): Gardasil vs. AAHS (females, age 21-46; follow-up until 48 months)**						
V501-019: Gardasil vs. AAHS (f, 21-46; 48)	0	1911	0	1908		Not estimable
**Subtotal (95% CI)**		**1911**		**1908**		**Not estimable**
Total events	0		0			
Heterogeneity: Not applicable						
Test for overall effect: Not applicable						
**2.14.9 New onset back pain ('new medical history'): Gardasil vs. AAHS (males, age 15-27; follow-up until 36 months)**						
V501-020: Gardasil vs. AAHS (m, 15-27; 36)	0	2032	0	2033		Not estimable
**Subtotal (95% CI)**		**2032**		**2033**		**Not estimable**
Total events	0		0			
Heterogeneity: Not applicable						
Test for overall effect: Not applicable						
**2.14.10 New onset back pain ('new medical history'): Gardasil 9 vs. saline placebo (females, age 12-26; follow-up until 7 months)**						
V503-006: Gardasil 9 vs. saline (f, 12-26;7)	0	618	0	306		Not estimable
**Subtotal (95% CI)**		**618**		**306**		**Not estimable**
Total events	0		0			
Heterogeneity: Not applicable						
Test for overall effect: Not applicable						
**2.14.11 New onset back pain ('new medical history'): HPV 16 vaccine vs. AAHS (females, age 16-25; follow-up until 48 months)**						
V501-005: HPV 16 vaccine vs. AAHS (f, 16-25; 48)	0	1204	0	1205		Not estimable
**Subtotal (95% CI)**		**1204**		**1205**		**Not estimable**
Total events	0		0			
Heterogeneity: Not applicable						
Test for overall effect: Not applicable						
**Total (95% CI)**		**29696**		**28794**	**100.0%**	**1.08 [0.77, 1.52]**
Total events	68		63			
Heterogeneity: Not applicable						
Test for overall effect: Z = 0.46 (P = 0.64)						
Test for subgroup differences: Not applicable						

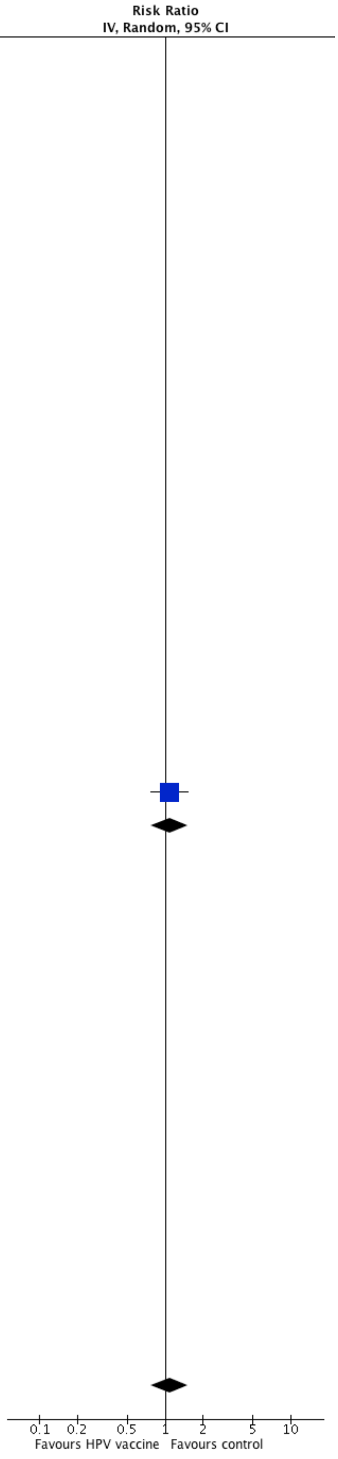

*2.14. Risk ratio for 'medically significant conditions’ (GlaxoSmithKline): not applicable; risk ratio for ‘new medical history’ (Merck Sharp & Dohme): 1.08 [0.77, 1.52].

2.15. New onset diseases most inversely associated with the HPV vaccines ('new medical history') - 'vaginal infection': intention to treat analysis

Study or Subgroup	HPV vaccine Events	HPV vaccine Total	Control Events	Control Total	Weight	Risk Ratio IV, Random, 95% CI
**2.15.1 New onset vaginal infection ('new medical history'): Gardasil vs. carrier solution (females and males, age 9-16; follow-up until 18 months)**						
V501-018: Gardasil vs. carrier solu. (f&m,9-16;18)	0	1184	0	597		Not estimable
**Subtotal (95% CI)**		**1184**		**597**		**Not estimable**
Total events	0		0			
Heterogeneity: Not applicable						
Test for overall effect: Not applicable						
**2.15.2 New onset vaginal infection ('new medical history'): Gardasil vs. AAHS (females, age 15-26; follow-up until 45 months)**						
V501-013: Gardasil vs. AAHS (f, 16-24; 45)	0	2723	0	2732		Not estimable
V501-015: Gardasil vs. AAHS (f, 15-26; 36)	0	6087	0	6080		Not estimable
**Subtotal (95% CI)**		**8810**		**8812**		**Not estimable**
Total events	0		0			
Heterogeneity: Not applicable						
Test for overall effect: Not applicable						
**2.15.3 New onset vaginal infection ('new medical history'): Gardasil vs. AAHS (females, age 21-46; follow-up until 48 months)**						
V501-019: Gardasil vs. AAHS (f, 21-46; 48)	0	1911	0	1908		Not estimable
**Subtotal (95% CI)**		**1911**		**1908**		**Not estimable**
Total events	0		0			
Heterogeneity: Not applicable						
Test for overall effect: Not applicable						
**2.15.4 New onset vaginal infection ('new medical history'): Gardasil 9 vs. saline placebo (females, age 12-26; follow-up until 7 months)**						
V503-006: Gardasil 9 vs. saline (f, 12-26;7)	0	618	0	306		Not estimable
**Subtotal (95% CI)**		**618**		**306**		**Not estimable**
Total events	0		0			
Heterogeneity: Not applicable						
Test for overall effect: Not applicable						
**Total (95% CI)**		**12523**		**11623**		**Not estimable**
Total events	0		0			
Heterogeneity: Not applicable						
Test for overall effect: Not applicable						
Test for subgroup differences: Not applicable						

Risk Ratio
IV, Random, 95% CI

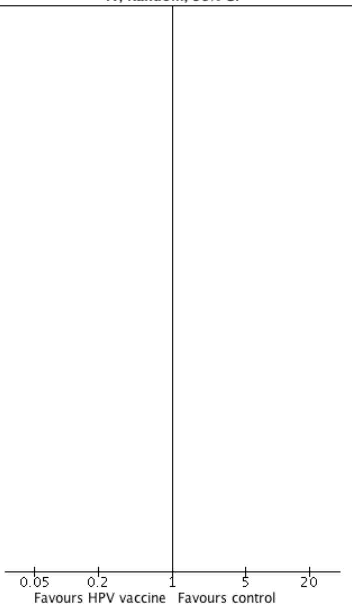

*2.15. Risk ratio for 'medically significant conditions' (GlaxoSmithKline): not applicable; risk ratio for 'new medical history' (Merck Sharp & Dohme): not applicable.

2.16. New onset diseases ('medically significant conditions' and 'new medical history') reported within the MedDRA system organ class ‘vascular disorders (10047065)': intention to treat analysis

Study or Subgroup	HPV vaccine Events	HPV vaccine Total	Control Events	Control Total	Weight	Risk Ratio IV, Random, 95% CI
**2.16.1 New onset vascular disorders ('medically significant conditions'): Cervarix vs. Al(OH)3 (females, age 24-72; follow-up until 36 months)**						
HPV-015: Cervarix vs. Al(OH)3 (f, 24-72; 36)	0	2882	0	2871		Not estimable
**Subtotal (95% CI)**		**2882**		**2871**		**Not estimable**
Total events	0		0			
Heterogeneity: Not applicable						
Test for overall effect: Not applicable						
**2.16.2 New onset vascular disorders ('medically significant conditions'): Cervarix vs. Aimmugen: hepatitis A vaccine (females, age 22-29; follow-up until 12 months)**						
HPV-063: Cervarix vs. Aimmugen (f, 22-29; 12)	0	375	0	377		Not estimable
**Subtotal (95% CI)**		**375**		**377**		**Not estimable**
Total events	0		0			
Heterogeneity: Not applicable						
Test for overall effect: Not applicable						
**2.16.3 New onset vascular disorders ('medically significant conditions'): Cervarix vs. Havrix: hepatitis A vaccine (females, age 10-14; follow-up until 12 months)**						
HPV-013: Cervarix vs. Havrix (f, 10-14; 12)	0	1035	0	1032		Not estimable
**Subtotal (95% CI)**		**1035**		**1032**		**Not estimable**
Total events	0		0			
Heterogeneity: Not applicable						
Test for overall effect: Not applicable						
**2.16.4 New onset vascular disorders ('medically significant conditions'): Cervarix vs. Havrix: hepatitis A vaccine (females, age 15-25; follow-up until 48 months)**						
HPV-008: Cervarix vs. Havrix (f, 15-25; 48)	0	9319	0	9325		Not estimable
**Subtotal (95% CI)**		**9319**		**9325**		**Not estimable**
Total events	0		0			
Heterogeneity: Not applicable						
Test for overall effect: Not applicable						
**2.16.5 New onset vascular disorders ('medically significant conditions'): Cervarix vs. Twinrix: hepatitis A and B vaccine (females, age 8-15; follow-up until 12 months)**						
HPV-029: Cervarix vs. Twinrix (f, 8-15; 12)	0	270	0	271		Not estimable
**Subtotal (95% CI)**		**270**		**271**		**Not estimable**
Total events	0		0			
Heterogeneity: Not applicable						
Test for overall effect: Not applicable						
**2.16.6 New onset vascular disorders ('new medical history'): Gardasil vs. carrier solution (females and males, age 9-16; follow-up until 18 months)**						
V501-018: Gardasil vs. carrier solu. (f&m,9-16;18)	0	1184	0	597		Not estimable
**Subtotal (95% CI)**		**1184**		**597**		**Not estimable**
Total events	0		0			
Heterogeneity: Not applicable						
Test for overall effect: Not applicable						
**2.16.7 New onset vascular disorders ('new medical history'): Gardasil vs. AAHS (females, age 15-26; follow-up until 45 months)**						
V501-013: Gardasil vs. AAHS (f, 16-24; 45)	0	2723	0	2732		Not estimable
V501-015: Gardasil vs. AAHS (f, 15-26; 36)	0	6087	0	6080		Not estimable
**Subtotal (95% CI)**		**8810**		**8812**		**Not estimable**
Total events	0		0			
Heterogeneity: Not applicable						
Test for overall effect: Not applicable						
**2.16.8 New onset vascular disorders ('new medical history'): Gardasil vs. AAHS (females, age 21-46; follow-up until 48 months)**						
V501-019: Gardasil vs. AAHS (f, 21-46; 48)	0	1911	0	1908		Not estimable
**Subtotal (95% CI)**		**1911**		**1908**		**Not estimable**
Total events	0		0			
Heterogeneity: Not applicable						
Test for overall effect: Not applicable						
**2.16.9 New onset vascular disorders ('new medical history'): Gardasil vs. AAHS (males, age 15-27; follow-up until 36 months)**						
V501-020: Gardasil vs. AAHS (m, 15-27; 36)	0	2032	0	2033		Not estimable
**Subtotal (95% CI)**		**2032**		**2033**		**Not estimable**
Total events	0		0			
Heterogeneity: Not applicable						
Test for overall effect: Not applicable						
**2.16.10 New onset vascular disorders ('new medical history'): HPV 16 vaccine vs. AAHS (females, age 16-25; follow-up until 48 months)**						
V501-005: HPV 16 vaccine vs. AAHS (f, 16-25; 48)	0	1204	0	1205		Not estimable
**Subtotal (95% CI)**		**1204**		**1205**		**Not estimable**
Total events	0		0			
Heterogeneity: Not applicable						
Test for overall effect: Not applicable						
**Total (95% CI)**		**29022**		**28431**		**Not estimable**
Total events	0		0			
Heterogeneity: Not applicable						
Test for overall effect: Not applicable						
Test for subgroup differences: Not applicable						

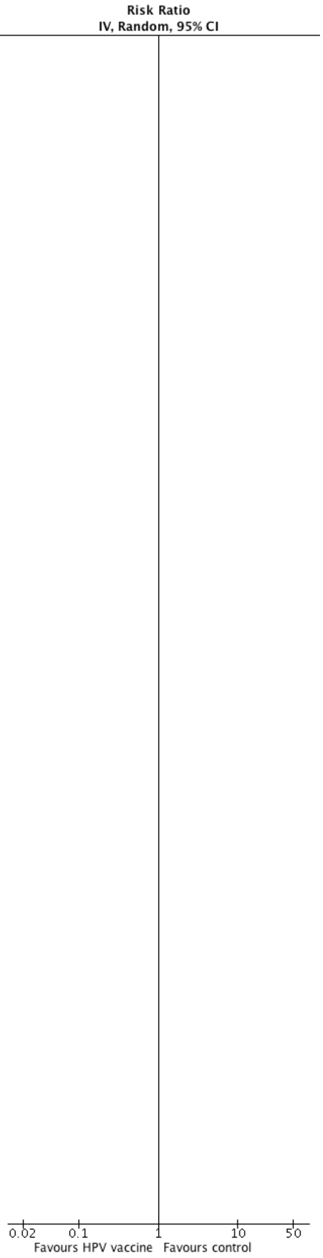

*2.16. Risk ratio for 'medically significant conditions’ (GlaxoSmithKline): not applicable; risk ratio for ‘new medical history’ (Merck Sharp & Dohme): not applicable.

2.17. General harms (‘solicited,’ ‘unsolicited’ and ‘systemic adverse events’*): intention to treat analysis

Study or Subgroup	HPV vaccine Events	HPV vaccine Total	Control Events	Control Total	Weight	Risk Ratio IV, Random, 95% CI
**2.17.1 General harms (‘unsolicited’): Cervarix vs. Al(OH)3 (females, age 9-17; follow-up until 12 months)**						
HPV-058: Cervarix vs. Al(OH)3 (f, 9-17; 12)	139	374	125	376	8.9%	1.12 [0.92, 1.36]
**Subtotal (95% CI)**		**374**		**376**	**8.9%**	**1.12 [0.92, 1.36]**
Total events	139		125			
Heterogeneity: Not applicable						
Test for overall effect: Z = 1.12 (P = 0.26)						
**2.17.2 General harms (‘unsolicited’): Cervarix vs. Al(OH)3 (females, age 15-26; follow-up until 27 months)**						
HPV-001: Cervarix vs. Al(OH)3 (f, 15-26; 27)	0	560	0	553		Not estimable
HPV-038: Cervarix vs. Al(OH)3 (f, 15-25; 7)	66	149	19	76	2.5%	1.77 [1.15, 2.72]
**Subtotal (95% CI)**		**709**		**629**	**2.5%**	**1.77 [1.15, 2.72]**
Total events	66		19			
Heterogeneity: Not applicable						
Test for overall effect: Z = 2.61 (P = 0.009)						
**2.17.3 General harms (‘unsolicited’): Cervarix vs. Al(OH)3 (females, age 18-35; follow-up until 12 months)**						
HPV-003: Cervarix vs. Al(OH)3 (f, 18-30; 12)	0	31	0	30		Not estimable
HPV-031: Cervarix vs. Al(OH)3 (f, 18-35; 7)	20	176	26	178	1.6%	0.78 [0.45, 1.34]
HPV-035: Cervarix vs. Al(OH)3 (f, 18-35; 7)	0	150	0	150		Not estimable
**Subtotal (95% CI)**		**357**		**358**	**1.6%**	**0.78 [0.45, 1.34]**
Total events	20		26			
Heterogeneity: Not applicable						
Test for overall effect: Z = 0.90 (P = 0.37)						
**2.17.4 General harms (‘unsolicited’): Cervarix vs. Al(OH)3 (females, age 24-72; follow-up until 36 months)**						
HPV-015: Cervarix vs. Al(OH)3 (f, 24-72; 36)	1153	2882	1164	2871	22.6%	0.99 [0.93, 1.05]
**Subtotal (95% CI)**		**2882**		**2871**	**22.6%**	**0.99 [0.93, 1.05]**
Total events	1153		1164			
Heterogeneity: Not applicable						
Test for overall effect: Z = 0.41 (P = 0.68)						
**2.17.5 General harms (‘unsolicited’): Cervarix vs. Aimmugen (females, age 19-25; follow-up until 7 months)**						
HPV-032: Cervarix vs. Aimmugen (f, 19-25; 7)	294	519	266	521	16.2%	1.11 [0.99, 1.24]
**Subtotal (95% CI)**		**519**		**521**	**16.2%**	**1.11 [0.99, 1.24]**
Total events	294		266			
Heterogeneity: Not applicable						
Test for overall effect: Z = 1.81 (P = 0.07)						
**2.17.6 General harms (‘unsolicited’): Cervarix vs. Engerix: hepatitis B vaccine (females and males, age 12-16; follow-up until 72 months)**						
HPV-030: Cervarix vs. Engerix (f, 9-15; 12)	99	247	99	246	7.8%	1.00 [0.80, 1.24]
HPV-040: Cervarix vs. Engerix (f & m, 12-16; 72)	157	643	202	1047	9.6%	1.27 [1.05, 1.52]
**Subtotal (95% CI)**		**890**		**1293**	**17.4%**	**1.13 [0.89, 1.43]**
Total events	256		301			
Heterogeneity: $Tau^2 = 0.02$; $Chi^2 = 2.75$, df = 1 (P = 0.10); $I^2 = 64\%$						
Test for overall effect: Z = 1.02 (P = 0.31)						
**2.17.7 General harms (‘unsolicited’): Cervarix vs. Engerix: hepatitis B vaccine (females, age 26-46; follow-up until 12 months)**						
HPV-069: Cervarix vs. Engerix (f, 26-46; 12)	32	606	36	606	2.2%	0.89 [0.56, 1.41]
**Subtotal (95% CI)**		**606**		**606**	**2.2%**	**0.89 [0.56, 1.41]**
Total events	32		36			
Heterogeneity: Not applicable						
Test for overall effect: Z = 0.50 (P = 0.62)						
**2.17.8 General harms (‘unsolicited’): Cervarix vs. Havrix: hepatitis A vaccine (females, age 9-15; follow-up until 12 months)**						
HPV-013: Cervarix vs. Havrix (f, 10-14; 12)	0	1035	0	1032		Not estimable
HPV-033: Cervarix vs. Havrix (f, 9-15; 7)	0	160	0	161		Not estimable
**Subtotal (95% CI)**		**1195**		**1193**		**Not estimable**
Total events	0		0			
Heterogeneity: Not applicable						
Test for overall effect: Not applicable						
**2.17.9 General harms (‘unsolicited’): Cervarix vs. Havrix: hepatitis A vaccine (females, age 15-25; follow-up until 48 months)**						
HPV-008: Cervarix vs. Havrix (f, 15-25; 48)	1466	9319	1448	9325	22.1%	1.01 [0.95, 1.08]
**Subtotal (95% CI)**		**9319**		**9325**	**22.1%**	**1.01 [0.95, 1.08]**
Total events	1466		1448			
Heterogeneity: Not applicable						
Test for overall effect: Z = 0.38 (P = 0.70)						
**2.17.10 General harms (‘unsolicited’): Cervarix vs. Twinrix: hepatitis A and B vaccine (females, age 8-15; follow-up until 12 months)**						
HPV-029: Cervarix vs. Twinrix (f, 8-15; 12)	96	270	83	271	6.6%	1.16 [0.91, 1.48]
**Subtotal (95% CI)**		**270**		**271**	**6.6%**	**1.16 [0.91, 1.48]**
Total events	96		83			
Heterogeneity: Not applicable						
Test for overall effect: Z = 1.22 (P = 0.22)						
**2.17.11 General harms (‘systemic adverse events’): Gardasil vs. carrier solution (females and males, age 9-16; follow-up until 18 months)**						
V501-018: Gardasil vs. carrier solu. (f&m,9-16;18)	0	1184	0	597		Not estimable
**Subtotal (95% CI)**		**1184**		**597**		**Not estimable**
Total events	0		0			
Heterogeneity: Not applicable						
Test for overall effect: Not applicable						
**2.17.12 General harms (‘systemic adverse events’): Gardasil vs. AAHS (females, age 15-26; follow-up until 45 months)**						
V501-013: Gardasil vs. AAHS (f, 16-24; 45)	0	2723	0	2732		Not estimable
V501-015: Gardasil vs. AAHS (f, 15-26; 36)	0	6087	0	6080		Not estimable
**Subtotal (95% CI)**		**8810**		**8812**		**Not estimable**
Total events	0		0			
Heterogeneity: Not applicable						
Test for overall effect: Not applicable						
**2.17.13 General harms (‘systemic adverse events’): Gardasil vs. AAHS (females, age 21-46; follow-up until 48 months)**						
V501-019: Gardasil vs. AAHS (f, 21-46; 48)	0	1911	0	1908		Not estimable
**Subtotal (95% CI)**		**1911**		**1908**		**Not estimable**
Total events	0		0			
Heterogeneity: Not applicable						
Test for overall effect: Not applicable						
**2.17.14 General harms (‘systemic adverse events’): Gardasil vs. AAHS (males, age 15-27; follow-up until 36 months)**						
V501-020: Gardasil vs. AAHS (m, 15-27; 36)	0	2032	0	2033		Not estimable
**Subtotal (95% CI)**		**2032**		**2033**		**Not estimable**
Total events	0		0			
Heterogeneity: Not applicable						
Test for overall effect: Not applicable						
**2.17.15 General harms (‘systemic adverse events’): Gardasil 9 vs. saline placebo (females, age 12-26; follow-up until 7 months)**						
V503-006: Gardasil 9 vs. saline (f, 12-26;7)	0	618	0	306		Not estimable
**Subtotal (95% CI)**		**618**		**306**		**Not estimable**
Total events	0		0			
Heterogeneity: Not applicable						
Test for overall effect: Not applicable						
**2.17.16 General harms (‘systemic adverse events’): HPV 16 vaccine vs. AAHS (females, age 16-25; follow-up until 48 months)**						
V501-005: HPV 16 vaccine vs. AAHS (f, 16-25; 48)	0	1204	0	1205		Not estimable
**Subtotal (95% CI)**		**1204**		**1205**		**Not estimable**
Total events	0		0			
Heterogeneity: Not applicable						
Test for overall effect: Not applicable						
**Total (95% CI)**		**32880**		**32304**	**100.0%**	**1.07 [1.00, 1.15]**
Total events	3522		3468			
Heterogeneity: $Tau^2 = 0.00$; $Chi^2 = 17.75$, df = 9 (P = 0.04); $I^2 = 49\%$						
Test for overall effect: Z = 1.84 (P = 0.07)						
Test for subgroup differences: $Chi^2 = 13.46$, df = 8 (P = 0.10), $I^2 = 40.6\%$						

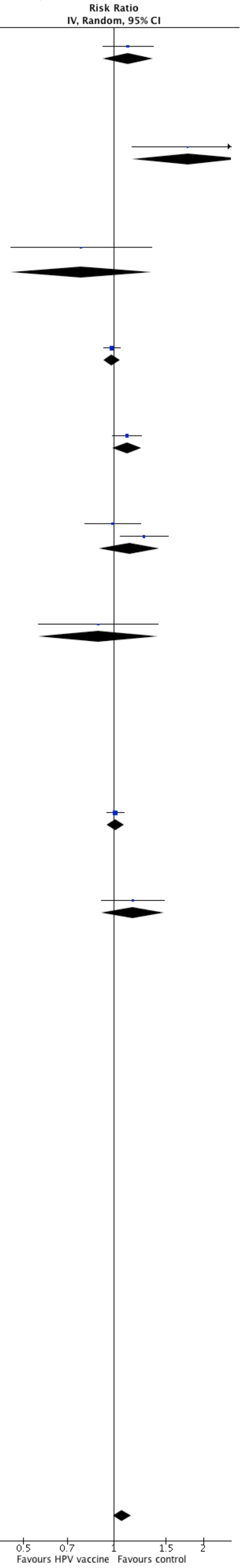

*2.17. Risk ratio for ‘solicited’ (GlaxoSmithKline): **1.07 [1.00, 1.15]**; risk ratio for ‘systemic adverse events’ (Merck Sharp & Dohme): not applicable.

2.18. General harms most associated with the HPV vaccines (‘solicited,’ ‘unsolicited’ and ‘systemic adverse events’*) - ‘fatigue’: intention to treat analysis

Study or Subgroup	HPV vaccine Events	HPV vaccine Total	Control Events	Control Total	Weight	Risk Ratio IV, Random, 95% CI
**2.18.1 General fatigue ('solicited'): Cervarix vs. Al(OH)3 (females, age 9-17; follow-up until 12 months)**						
HPV-058: Cervarix vs. Al(OH)3 (f, 9-17; 12)	137	374	126	376	5.9%	1.09 [0.90, 1.33]
**Subtotal (95% CI)**		**374**		**376**	**5.9%**	**1.09 [0.90, 1.33]**
Total events	137		126			
Heterogeneity: Not applicable						
Test for overall effect: Z = 0.89 (P = 0.37)						
**2.18.2 General fatigue ('solicited'): Cervarix vs. Al(OH)3 (females, age 15-26; follow-up until 27 months)**						
HPV-001: Cervarix vs. Al(OH)3 (f, 15-26; 27)	0	560	0	553		Not estimable
HPV-038: Cervarix vs. Al(OH)3 (f, 15-25; 7)	103	149	39	76	4.1%	1.35 [1.06, 1.72]
**Subtotal (95% CI)**		**709**		**629**	**4.1%**	**1.35 [1.06, 1.72]**
Total events	103		39			
Heterogeneity: Not applicable						
Test for overall effect: Z = 2.39 (P = 0.02)						
**2.18.3 General fatigue ('solicited'): Cervarix vs. Al(OH)3 (females, age 18-35; follow-up until 12 months)**						
HPV-003: Cervarix vs. Al(OH)3 (f, 18-30; 12)	0	31	0	30		Not estimable
HPV-031: Cervarix vs. Al(OH)3 (f, 18-35; 7)	84	176	83	178	4.8%	1.02 [0.82, 1.28]
HPV-035: Cervarix vs. Al(OH)3 (f, 18-35; 7)	0	150	0	150		Not estimable
**Subtotal (95% CI)**		**357**		**358**	**4.8%**	**1.02 [0.82, 1.28]**
Total events	84		83			
Heterogeneity: Not applicable						
Test for overall effect: Z = 0.21 (P = 0.84)						
**2.18.4 General fatigue ('solicited'): Cervarix vs. Al(OH)3 (females, age 24-72; follow-up until 36 months)**						
HPV-015: Cervarix vs. Al(OH)3 (f, 24-72; 36)	1116	2882	916	2871	19.1%	1.21 [1.13, 1.30]
**Subtotal (95% CI)**		**2882**		**2871**	**19.1%**	**1.21 [1.13, 1.30]**
Total events	1116		916			
Heterogeneity: Not applicable						
Test for overall effect: Z = 5.39 (P < 0.00001)						
**2.18.5 General fatigue ('solicited'): Cervarix vs. Aimmugen: hepatitis A vaccine (females, age 19-29; follow-up until 12 months)**						
HPV-032: Cervarix vs. Aimmugen (f, 19-25; 7)	341	519	300	521	14.8%	1.14 [1.04, 1.26]
**Subtotal (95% CI)**		**519**		**521**	**14.8%**	**1.14 [1.04, 1.26]**
Total events	341		300			
Heterogeneity: Not applicable						
Test for overall effect: Z = 2.68 (P = 0.007)						
**2.18.6 General fatigue ('solicited'): Cervarix vs. Engerix: hepatitis B vaccine (females and males, age 9-16; follow-up until 72 months)**						
HPV-030: Cervarix vs. Engerix (f, 9-15; 12)	107	247	104	246	5.5%	1.02 [0.84, 1.26]
HPV-040: Cervarix vs. Engerix (f & m, 12-16; 72)	291	643	411	1047	12.4%	1.15 [1.03, 1.29]
**Subtotal (95% CI)**		**890**		**1293**	**17.9%**	**1.12 [1.02, 1.24]**
Total events	398		515			
Heterogeneity: Tau² = 0.00; Chi² = 0.98, df = 1 (P = 0.32); I² = 0%						
Test for overall effect: Z = 2.26 (P = 0.02)						
**2.18.7 General fatigue ('solicited'): Cervarix vs. Engerix: hepatitis B vaccine (females, age 26-46; follow-up until 12 months)**						
HPV-069: Cervarix vs. Engerix (f, 26-46; 12)	100	606	68	606	3.1%	1.47 [1.10, 1.96]
**Subtotal (95% CI)**		**606**		**606**	**3.1%**	**1.47 [1.10, 1.96]**
Total events	100		68			
Heterogeneity: Not applicable						
Test for overall effect: Z = 2.64 (P = 0.008)						
**2.18.8 General fatigue ('solicited'): Cervarix vs. Havrix: hepatitis A vaccine (females, age 9-15; follow-up until 12 months)**						
HPV-013: Cervarix vs. Havrix (f, 10-14; 12)	0	1035	0	1032		Not estimable
HPV-033: Cervarix vs. Havrix (f, 9-15; 7)	0	160	0	161		Not estimable
**Subtotal (95% CI)**		**1195**		**1193**		**Not estimable**
Total events	0		0			
Heterogeneity: Not applicable						
Test for overall effect: Not applicable						
**2.18.9 General fatigue ('solicited'): Cervarix vs. Havrix: hepatitis A vaccine (females, age 15-25; follow-up until 48 months)**						
HPV-008: Cervarix vs. Havrix (f, 15-25; 48)	1771	9319	1652	9325	20.9%	1.07 [1.01, 1.14]
**Subtotal (95% CI)**		**9319**		**9325**	**20.9%**	**1.07 [1.01, 1.14]**
Total events	1771		1652			
Heterogeneity: Not applicable						
Test for overall effect: Z = 2.27 (P = 0.02)						
**2.18.10 General fatigue ('solicited'): Cervarix vs. Twinrix: hepatitis A and B vaccine (females, age 8-15; follow-up until 12 months)**						
HPV-029: Cervarix vs. Twinrix (f, 8-15; 12)	133	270	119	271	6.6%	1.12 [0.94, 1.34]
**Subtotal (95% CI)**		**270**		**271**	**6.6%**	**1.12 [0.94, 1.34]**
Total events	133		119			
Heterogeneity: Not applicable						
Test for overall effect: Z = 1.24 (P = 0.21)						
**2.18.11 General fatigue ('unsolicited'): Cervarix vs. Engerix: hepatitis B vaccine (females and males, age 9-16; follow-up until 72 months)**						
HPV-030: Cervarix vs. Engerix (f, 9-15; 12)	0	247	0	246		Not estimable
HPV-040: Cervarix vs. Engerix (f & m, 12-16; 72)	0	1908	0	3128		Not estimable
**Subtotal (95% CI)**		**2155**		**3374**		**Not estimable**
Total events	0		0			
Heterogeneity: Not applicable						
Test for overall effect: Not applicable						
**2.18.12 General fatigue ('systemic adverse events'): Gardasil vs. carrier solution (females and males, age 9-16; follow-up until 18 months)**						
V501-018: Gardasil vs. carrier solu. (f&m,9-16;18)	0	1184	0	597		Not estimable
**Subtotal (95% CI)**		**1184**		**597**		**Not estimable**
Total events	0		0			
Heterogeneity: Not applicable						
Test for overall effect: Not applicable						
**2.18.13 General fatigue ('systemic adverse events'): Gardasil vs. AAHS (males, age 15-27; follow-up until 36 months)**						
V501-020: Gardasil vs. AAHS (m, 15-27; 36)	13	2032	19	2033	0.6%	0.68 [0.34, 1.38]
**Subtotal (95% CI)**		**2032**		**2033**	**0.6%**	**0.68 [0.34, 1.38]**
Total events	13		19			
Heterogeneity: Not applicable						
Test for overall effect: Z = 1.06 (P = 0.29)						
**2.18.14 General fatigue ('systemic adverse events'): Gardasil 9 vs. saline placebo (females, age 12-26; follow-up until 7 months)**						
V503-006: Gardasil 9 vs. saline (f, 12-26;7)	0	618	0	306		Not estimable
**Subtotal (95% CI)**		**618**		**306**		**Not estimable**
Total events	0		0			
Heterogeneity: Not applicable						
Test for overall effect: Not applicable						
**2.18.15 General fatigue ('systemic adverse events'): HPV 16 vaccine vs. AAHS (females, age 16-25; follow-up until 48 months)**						
V501-005: HPV 16 vaccine vs. AAHS (f, 16-25; 48)	59	1204	64	1205	2.2%	0.92 [0.65, 1.30]
**Subtotal (95% CI)**		**1204**		**1205**	**2.2%**	**0.92 [0.65, 1.30]**
Total events	59		64			
Heterogeneity: Not applicable						
Test for overall effect: Z = 0.46 (P = 0.65)						
**Total (95% CI)**		**24314**		**24958**	**100.0%**	**1.13 [1.07, 1.19]**
Total events	4255		3901			
Heterogeneity: Tau² = 0.00; Chi² = 17.23, df = 11 (P = 0.10); I² = 36%						
Test for overall effect: Z = 4.58 (P < 0.00001)						
Test for subgroup differences: Chi² = 16.25, df = 10 (P = 0.09), I² = 38.5%						

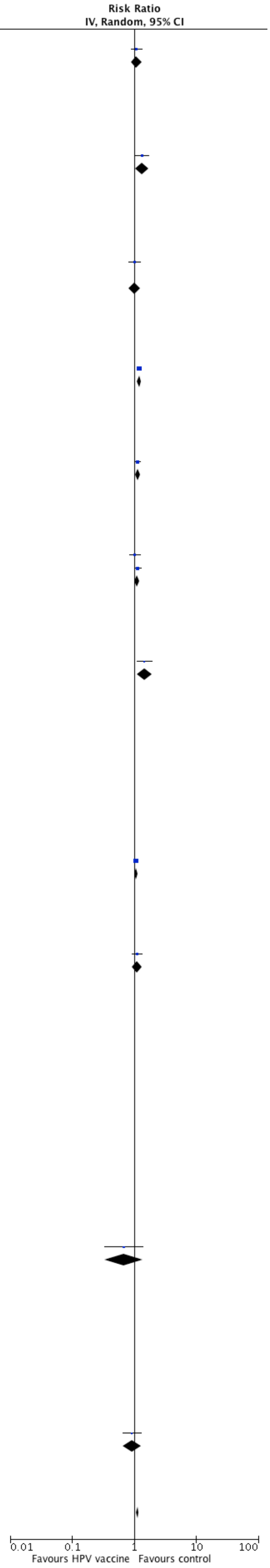

*2.18. Risk ratio for ‘solicited’ (GlaxoSmithKline): **1.14 [1.08, 1.20]**; risk ratio for ‘unsolicited’ (GlaxoSmithKline): not applicable; risk ratio for ‘systemic adverse events’ (Merck Sharp & Dohme): 0.87 [0.64, 1.19]. To avoid double counting of participants in the total risk ratio estimate, we excluded the ‘unsolicited’ adverse events from total risk ratio estimate for studies that reported ‘solicited’ adverse events.

2.19. General harms most associated with the HPV vaccines (‘solicited,’ ‘unsolicited’ and ‘systemic adverse events’*) - ‘headache’: intention to treat analysis

Study or Subgroup	HPV vaccine Events	HPV vaccine Total	Control Events	Control Total	Weight	Risk Ratio IV, Random, 95% CI
**2.19.1 General headache ('solicited'): Cervarix vs. Al(OH)3 (females, age 9-17; follow-up until 12 months)**						
HPV-058: Cervarix vs. Al(OH)3 (f, 9-17; 12)	123	374	99	376	3.4%	1.25 [1.00, 1.56]
**Subtotal (95% CI)**		**374**		**376**	**3.4%**	**1.25 [1.00, 1.56]**
Total events	123		99			
Heterogeneity: Not applicable						
Test for overall effect: Z = 1.96 (P = 0.05)						
**2.19.2 General headache ('solicited'): Cervarix vs. Al(OH)3 (females, age 15-26; follow-up until 27 months)**						
HPV-001: Cervarix vs. Al(OH)3 (f, 15-26; 27)	0	560	0	553		Not estimable
HPV-038: Cervarix vs. Al(OH)3 (f, 15-25; 7)	73	149	30	76	1.7%	1.24 [0.90, 1.71]
**Subtotal (95% CI)**		**709**		**629**	**1.7%**	**1.24 [0.90, 1.71]**
Total events	73		30			
Heterogeneity: Not applicable						
Test for overall effect: Z = 1.31 (P = 0.19)						
**2.19.3 General headache ('solicited'): Cervarix vs. Al(OH)3 (females, age 18-35; follow-up until 12 months)**						
HPV-003: Cervarix vs. Al(OH)3 (f, 18-30; 12)	0	31	0	30		Not estimable
HPV-031: Cervarix vs. Al(OH)3 (f, 18-35; 7)	72	176	71	178	2.7%	1.03 [0.80, 1.32]
HPV-035: Cervarix vs. Al(OH)3 (f, 18-35; 7)	0	150	0	150		Not estimable
**Subtotal (95% CI)**		**357**		**358**	**2.7%**	**1.03 [0.80, 1.32]**
Total events	72		71			
Heterogeneity: Not applicable						
Test for overall effect: Z = 0.20 (P = 0.84)						
**2.19.4 General headache ('solicited'): Cervarix vs. Al(OH)3 (females, age 24-72; follow-up until 36 months)**						
HPV-015: Cervarix vs. Al(OH)3 (f, 24-72; 36)	1165	2882	1074	2871	19.3%	1.08 [1.01, 1.15]
**Subtotal (95% CI)**		**2882**		**2871**	**19.3%**	**1.08 [1.01, 1.15]**
Total events	1165		1074			
Heterogeneity: Not applicable						
Test for overall effect: Z = 2.34 (P = 0.02)						
**2.19.5 General headache ('solicited'): Cervarix vs. Aimmugen: hepatitis A vaccine (females, age 19-29; follow-up until 12 months)**						
HPV-032: Cervarix vs. Aimmugen (f, 19-25; 7)	250	519	222	521	8.0%	1.13 [0.99, 1.29]
**Subtotal (95% CI)**		**519**		**521**	**8.0%**	**1.13 [0.99, 1.29]**
Total events	250		222			
Heterogeneity: Not applicable						
Test for overall effect: Z = 1.80 (P = 0.07)						
**2.19.6 General headache ('solicited'): Cervarix vs. Engerix: hepatitis B vaccine (females and males, age 9-16; follow-up until 72 months)**						
HPV-030: Cervarix vs. Engerix (f, 9-15; 12)	131	247	129	246	5.6%	1.01 [0.86, 1.20]
HPV-040: Cervarix vs. Engerix (f & m, 12-16; 72)	261	643	371	1047	8.9%	1.15 [1.01, 1.30]
**Subtotal (95% CI)**		**890**		**1293**	**14.5%**	**1.09 [0.97, 1.23]**
Total events	392		500			
Heterogeneity: $Tau^2 = 0.00$; $Chi^2 = 1.37$, df = 1 (P = 0.24); $I^2 = 27\%$						
Test for overall effect: Z = 1.42 (P = 0.15)						
**2.19.7 General headache ('solicited'): Cervarix vs. Engerix: hepatitis B vaccine (females, age 26-46; follow-up until 12 months)**						
HPV-069: Cervarix vs. Engerix (f, 26-46; 12)	71	606	46	606	1.4%	1.54 [1.08, 2.20]
**Subtotal (95% CI)**		**606**		**606**	**1.4%**	**1.54 [1.08, 2.20]**
Total events	71		46			
Heterogeneity: Not applicable						
Test for overall effect: Z = 2.41 (P = 0.02)						
**2.19.8 General headache ('solicited'): Cervarix vs. Havrix: hepatitis A vaccine (females, age 9-15; follow-up until 12 months)**						
HPV-013: Cervarix vs. Havrix (f, 10-14; 12)	0	1035	0	1032		Not estimable
HPV-033: Cervarix vs. Havrix (f, 9-15; 7)	0	160	0	161		Not estimable
**Subtotal (95% CI)**		**1195**		**1193**		**Not estimable**
Total events	0		0			
Heterogeneity: Not applicable						
Test for overall effect: Not applicable						
**2.19.9 General headache ('solicited'): Cervarix vs. Havrix: hepatitis A vaccine (females, age 15-25; follow-up until 48 months)**						
HPV-008: Cervarix vs. Havrix (f, 15-25; 48)	1668	9319	1583	9325	19.9%	1.05 [0.99, 1.12]
**Subtotal (95% CI)**		**9319**		**9325**	**19.9%**	**1.05 [0.99, 1.12]**
Total events	1668		1583			
Heterogeneity: Not applicable						
Test for overall effect: Z = 1.66 (P = 0.10)						
**2.19.10 General headache ('solicited'): Cervarix vs. Twinrix: hepatitis A and B vaccine (females, age 8-15; follow-up until 12 months)**						
HPV-029: Cervarix vs. Twinrix (f, 8-15; 12)	124	270	110	271	4.3%	1.13 [0.93, 1.37]
**Subtotal (95% CI)**		**270**		**271**	**4.3%**	**1.13 [0.93, 1.37]**
Total events	124		110			
Heterogeneity: Not applicable						
Test for overall effect: Z = 1.25 (P = 0.21)						
**2.19.11 General headache ('unsolicited'): Cervarix vs. Al(OH)3 (females, age 9-17; follow-up until 12 months)**						
HPV-058: Cervarix vs. Al(OH)3 (f, 9-17; 12)	0	374	0	376		Not estimable
**Subtotal (95% CI)**		**0**		**0**		**Not estimable**
Total events	0		0			
Heterogeneity: Not applicable						
Test for overall effect: Not applicable						
**2.19.12 General headache ('unsolicited'): Cervarix vs. Al(OH)3 (females, age 15-25; follow-up until 7 months)**						
HPV-038: Cervarix vs. Al(OH)3 (f, 15-25; 7)	9	149	4	76	0.0%	1.15 [0.37, 3.61]
**Subtotal (95% CI)**		**0**		**0**		**Not estimable**
Total events	0		0			
Heterogeneity: Not applicable						
Test for overall effect: Not applicable						
**2.19.13 General headache ('unsolicited'): Cervarix vs. Al(OH)3 (females, age 18-35; follow-up until 7 months)**						
HPV-031: Cervarix vs. Al(OH)3 (f, 18-35; 7)	0	176	0	178		Not estimable
HPV-035: Cervarix vs. Al(OH)3 (f, 18-35; 7)	0	150	0	150		Not estimable
**Subtotal (95% CI)**		**0**		**0**		**Not estimable**
Total events	0		0			
Heterogeneity: Not applicable						
Test for overall effect: Not applicable						
**2.19.14 General headache (unsolicited'): Cervarix vs. Aimmugen: hepatitis A vaccine (females, age 19-29; follow-up until 12 months)**						
HPV-032: Cervarix vs. Aimmugen (f, 19-25; 7)	19	519	27	521	0.0%	0.71 [0.40, 1.25]
**Subtotal (95% CI)**		**0**		**0**		**Not estimable**
Total events	0		0			
Heterogeneity: Not applicable						
Test for overall effect: Not applicable						
**2.19.15 General headache ('unsolicited'): Cervarix vs. Engerix: hepatitis B vaccine (females and males, age 9-16; follow-up until 72 months)**						
HPV-030: Cervarix vs. Engerix (f, 9-15; 12)	0	247	0	246		Not estimable
HPV-040: Cervarix vs. Engerix (f & m, 12-16; 72)	0	1908	0	3128		Not estimable
**Subtotal (95% CI)**		**0**		**0**		**Not estimable**
Total events	0		0			
Heterogeneity: Not applicable						
Test for overall effect: Not applicable						
**2.19.16 General headache ('unsolicited'): Cervarix vs. Havrix: hepatitis A vaccine (females, age 15-25; follow-up until 48 months)**						
HPV-008: Cervarix vs. Havrix (f, 15-25; 48)	228	9319	250	9325	0.0%	0.91 [0.76, 1.09]
**Subtotal (95% CI)**		**0**		**0**		**Not estimable**
Total events	0		0			
Heterogeneity: Not applicable						
Test for overall effect: Not applicable						
**2.19.17 General headache ('unsolicited'): Cervarix vs. Twinrix: hepatitis A and B vaccine (females, age 8-15; follow-up until 12 months)**						
HPV-029: Cervarix vs. Twinrix (f, 8-15; 12)	0	270	0	271		Not estimable
**Subtotal (95% CI)**		**0**		**0**		**Not estimable**
Total events	0		0			
Heterogeneity: Not applicable						
Test for overall effect: Not applicable						
**2.19.18 General headache ('systemic adverse events'): Gardasil vs. carrier solution (females and males, age 9-16; follow-up until 18 months)**						
V501-018: Gardasil vs. carrier solu. (f&m,9-16;18)	0	1184	0	597		Not estimable
**Subtotal (95% CI)**		**1184**		**597**		**Not estimable**
Total events	0		0			
Heterogeneity: Not applicable						
Test for overall effect: Not applicable						
**2.19.19 General headache ('systemic adverse events'): Gardasil vs. AAHS (males, age 15-27; follow-up until 36 months)**						
V501-020: Gardasil vs. AAHS (m, 15-27; 36)	179	2032	207	2033	4.4%	0.87 [0.72, 1.05]
**Subtotal (95% CI)**		**2032**		**2033**	**4.4%**	**0.87 [0.72, 1.05]**
Total events	179		207			
Heterogeneity: Not applicable						
Test for overall effect: Z = 1.49 (P = 0.14)						
**2.19.20 General headache ('systemic adverse events'): Gardasil 9 vs. saline placebo (females, age 12-26; follow-up until 7 months)**						
V503-006: Gardasil 9 vs. saline (f, 12-26;7)	318	618	150	306	7.6%	1.05 [0.91, 1.20]
**Subtotal (95% CI)**		**618**		**306**	**7.6%**	**1.05 [0.91, 1.20]**
Total events	318		150			
Heterogeneity: Not applicable						
Test for overall effect: Z = 0.69 (P = 0.49)						
**2.19.21 General headache ('systemic adverse events'): HPV 16 vaccine vs. AAHS (females, age 16-25; follow-up until 48 months)**						
V501-005: HPV 16 vaccine vs. AAHS (f, 16-25; 48)	499	1204	495	1205	12.8%	1.01 [0.92, 1.11]
**Subtotal (95% CI)**		**1204**		**1205**	**12.8%**	**1.01 [0.92, 1.11]**
Total events	499		495			
Heterogeneity: Not applicable						
Test for overall effect: Z = 0.18 (P = 0.86)						
**Total (95% CI)**		**22159**		**21584**	**100.0%**	**1.07 [1.03, 1.12]**
Total events	4934		4587			
Heterogeneity: $Tau^2 = 0.00$; $Chi^2 = 16.08$, df = 12 (P = 0.19); $I^2 = 25\%$						
Test for overall effect: Z = 3.18 (P = 0.001)						
Test for subgroup differences: $Chi^2 = 14.57$, df = 11 (P = 0.20), $I^2 = 24.5\%$						

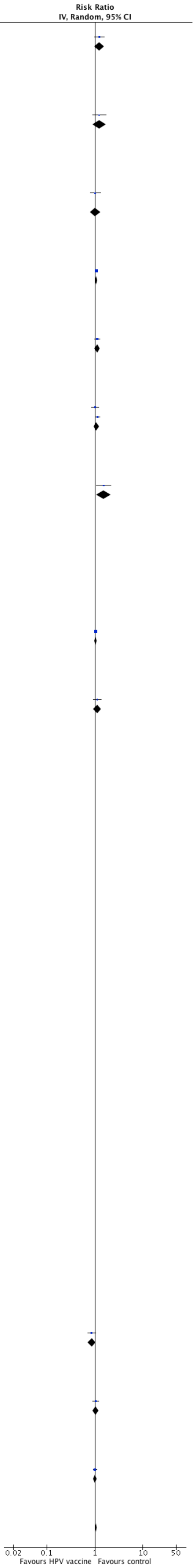

*2.19. Risk ratio for ‘solicited’ (GlaxoSmithKline): **1.09 [1.05, 1.13]**; risk ratio for ‘unsolicited’ (GlaxoSmithKline): 0.90 [0.76, 1.06]; risk ratio for ‘systemic adverse events’ (Merck Sharp & Dohme): 0.99 [0.91, 1.09]. To avoid double counting of participants in the total risk ratio estimate, we excluded the ‘unsolicited’ adverse events from total risk ratio estimate for studies that reported ‘solicited’ adverse events.

2.20. General harms most associated with the HPV vaccines (‘solicited,’ ‘unsolicited’ and ‘systemic adverse events’*) - ‘myalgia’: intention to treat analysis

Study or Subgroup	HPV vaccine Events	HPV vaccine Total	Control Events	Control Total	Weight	Risk Ratio IV, Random, 95% CI
**2.20.1 General myalgia ('solicited'): Cervarix vs. Al(OH)3 (females, age 9-17; follow-up until 12 months)**						
HPV-058: Cervarix vs. Al(OH)3 (f, 9-17; 12)	110	374	93	376	10.5%	1.19 [0.94, 1.51]
**Subtotal (95% CI)**		**374**		**376**	**10.5%**	**1.19 [0.94, 1.51]**
Total events	110		93			
Heterogeneity: Not applicable						
Test for overall effect: Z = 1.44 (P = 0.15)						
**2.20.2 General myalgia ('solicited'): Cervarix vs. Al(OH)3 (females, age 15-25; follow-up until 7 months)**						
HPV-038: Cervarix vs. Al(OH)3 (f, 15-25; 7)	97	149	33	76	9.7%	1.50 [1.13, 1.99]
**Subtotal (95% CI)**		**149**		**76**	**9.7%**	**1.50 [1.13, 1.99]**
Total events	97		33			
Heterogeneity: Not applicable						
Test for overall effect: Z = 2.81 (P = 0.005)						
**2.20.3 General myalgia ('solicited'): Cervarix vs. Al(OH)3 (females, age 18-35; follow-up until 12 months)**						
HPV-003: Cervarix vs. Al(OH)3 (f, 18-30; 12)	0	31	0	30		Not estimable
HPV-031: Cervarix vs. Al(OH)3 (f, 18-35; 7)	10	176	11	178	3.2%	0.92 [0.40, 2.11]
HPV-035: Cervarix vs. Al(OH)3 (f, 18-35; 7)	0	150	0	150		Not estimable
**Subtotal (95% CI)**		**357**		**358**	**3.2%**	**0.92 [0.40, 2.11]**
Total events	10		11			
Heterogeneity: Not applicable						
Test for overall effect: Z = 0.20 (P = 0.84)						
**2.20.4 General myalgia ('solicited'): Cervarix vs. Al(OH)3 (females, age 24-72; follow-up until 36 months)**						
HPV-015: Cervarix vs. Al(OH)3 (f, 24-72; 36)	878	2882	622	2871	12.7%	1.41 [1.29, 1.54]
**Subtotal (95% CI)**		**2882**		**2871**	**12.7%**	**1.41 [1.29, 1.54]**
Total events	878		622			
Heterogeneity: Not applicable						
Test for overall effect: Z = 7.53 (P < 0.00001)						
**2.20.5 General myalgia ('solicited'): Cervarix vs. Aimmugen: hepatitis A vaccine (females, age 19-29; follow-up until 12 months)**						
HPV-032: Cervarix vs. Aimmugen (f, 19-25; 7)	262	519	128	521	11.6%	2.05 [1.73, 2.44]
**Subtotal (95% CI)**		**519**		**521**	**11.6%**	**2.05 [1.73, 2.44]**
Total events	262		128			
Heterogeneity: Not applicable						
Test for overall effect: Z = 8.16 (P < 0.00001)						
**2.20.6 General myalgia ('solicited'): Cervarix vs. Engerix: hepatitis B vaccine (females and males, age 9-16; follow-up until 72 months)**						
HPV-030: Cervarix vs. Engerix (f, 9-15; 12)	55	247	53	246	8.8%	1.03 [0.74, 1.44]
HPV-040: Cervarix vs. Engerix (f & m, 12-16; 72)	321	643	250	1047	12.2%	2.09 [1.83, 2.39]
**Subtotal (95% CI)**		**890**		**1293**	**20.9%**	**1.50 [0.75, 2.98]**
Total events	376		303			
Heterogeneity: $Tau^2 = 0.23$; $Chi^2 = 14.80$, df = 1 (P = 0.0001); $I^2 = 93\%$						
Test for overall effect: Z = 1.14 (P = 0.25)						
**2.20.7 General myalgia ('solicited'): Cervarix vs. Engerix: hepatitis B vaccine (females, age 26-46; follow-up until 12 months)**						
HPV-069: Cervarix vs. Engerix (f, 26-46; 12)	69	606	42	606	8.2%	1.64 [1.14, 2.37]
**Subtotal (95% CI)**		**606**		**606**	**8.2%**	**1.64 [1.14, 2.37]**
Total events	69		42			
Heterogeneity: Not applicable						
Test for overall effect: Z = 2.65 (P = 0.008)						
**2.20.8 General myalgia ('solicited'): Cervarix vs. Havrix: hepatitis A vaccine (females, age 9-15; follow-up until 12 months)**						
HPV-013: Cervarix vs. Havrix (f, 10-14; 12)	0	1035	0	1032		Not estimable
HPV-033: Cervarix vs. Havrix (f, 9-15; 7)	0	160	0	161		Not estimable
**Subtotal (95% CI)**		**1195**		**1193**		**Not estimable**
Total events	0		0			
Heterogeneity: Not applicable						
Test for overall effect: Not applicable						
**2.20.9 General myalgia ('solicited'): Cervarix vs. Havrix: hepatitis A vaccine (females, age 15-25; follow-up until 48 months)**						
HPV-008: Cervarix vs. Havrix (f, 15-25; 48)	1607	9319	1381	9325	12.9%	1.16 [1.09, 1.24]
**Subtotal (95% CI)**		**9319**		**9325**	**12.9%**	**1.16 [1.09, 1.24]**
Total events	1607		1381			
Heterogeneity: Not applicable						
Test for overall effect: Z = 4.52 (P < 0.00001)						
**2.20.10 General myalgia ('solicited'): Cervarix vs. Twinrix: hepatitis A and B vaccine (females, age 8-15; follow-up until 12 months)**						
HPV-029: Cervarix vs. Twinrix (f, 8-15; 12)	99	270	75	271	10.3%	1.32 [1.03, 1.70]
**Subtotal (95% CI)**		**270**		**271**	**10.3%**	**1.32 [1.03, 1.70]**
Total events	99		75			
Heterogeneity: Not applicable						
Test for overall effect: Z = 2.22 (P = 0.03)						
**2.20.11 General myalgia ('unsolicited'): Cervarix vs. Al(OH)3 (females, age 15-25; follow-up until 7 months)**						
HPV-038: Cervarix vs. Al(OH)3 (f, 15-25; 7)	0	149	0	76		Not estimable
**Subtotal (95% CI)**		**149**		**76**		**Not estimable**
Total events	0		0			
Heterogeneity: Not applicable						
Test for overall effect: Not applicable						
**2.20.12 General myalgia ('unsolicited'): Cervarix vs. Al(OH)3 (females, age 18-35; follow-up until 7 months)**						
HPV-031: Cervarix vs. Al(OH)3 (f, 18-35; 7)	0	176	0	178		Not estimable
**Subtotal (95% CI)**		**176**		**178**		**Not estimable**
Total events	0		0			
Heterogeneity: Not applicable						
Test for overall effect: Not applicable						
**2.20.13 General myalgia ('unsolicited'): Cervarix vs. Engerix: hepatitis B vaccine (females and males, age 12-16; follow-up until 72 months)**						
HPV-040: Cervarix vs. Engerix (f & m, 12-16; 72)	0	1908	0	3128		Not estimable
**Subtotal (95% CI)**		**1908**		**3128**		**Not estimable**
Total events	0		0			
Heterogeneity: Not applicable						
Test for overall effect: Not applicable						
**2.20.14 General myalgia ('unsolicited'): Cervarix vs. Havrix: hepatitis A vaccine (females, age 9-15; follow-up until 7 months)**						
HPV-033: Cervarix vs. Havrix (f, 9-15; 7)	0	160	0	161		Not estimable
**Subtotal (95% CI)**		**160**		**161**		**Not estimable**
Total events	0		0			
Heterogeneity: Not applicable						
Test for overall effect: Not applicable						
**2.20.15 General myalgia ('systemic adverse events'): Gardasil vs. carrier solution (females and males, age 9-16; follow-up until 18 months)**						
V501-018: Gardasil vs. carrier solu. (f&m,9-16;18)	0	1184	0	597		Not estimable
**Subtotal (95% CI)**		**1184**		**597**		**Not estimable**
Total events	0		0			
Heterogeneity: Not applicable						
Test for overall effect: Not applicable						
**2.20.16 General myalgia ('systemic adverse events'): Gardasil vs. AAHS (males, age 15-27; follow-up until 36 months)**						
V501-020: Gardasil vs. AAHS (m, 15-27; 36)	0	2032	0	2033		Not estimable
**Subtotal (95% CI)**		**2032**		**2033**		**Not estimable**
Total events	0		0			
Heterogeneity: Not applicable						
Test for overall effect: Not applicable						
**2.20.17 General myalgia ('systemic adverse events'): Gardasil 9 vs. saline placebo (females, age 12-26; follow-up until 7 months)**						
V503-006: Gardasil 9 vs. saline (f, 12-26;7)	0	618	0	306		Not estimable
**Subtotal (95% CI)**		**618**		**306**		**Not estimable**
Total events	0		0			
Heterogeneity: Not applicable						
Test for overall effect: Not applicable						
**2.20.18 General myalgia ('systemic adverse events'): HPV 16 vaccine vs. AAHS (females, age 16-25; follow-up until 48 months)**						
V501-005: HPV 16 vaccine vs. AAHS (f, 16-25; 48)	0	1204	0	1205		Not estimable
**Subtotal (95% CI)**		**1204**		**1205**		**Not estimable**
Total events	0		0			
Heterogeneity: Not applicable						
Test for overall effect: Not applicable						
**Total (95% CI)**		**23992**		**24574**	**100.0%**	**1.44 [1.21, 1.71]**
Total events	3508		2688			
Heterogeneity: $Tau^2 = 0.06$; $Chi^2 = 90.02$, df = 9 (P < 0.00001); $I^2 = 90\%$						
Test for overall effect: Z = 4.18 (P < 0.0001)						
Test for subgroup differences: $Chi^2 = 44.56$, df = 8 (P < 0.00001), $I^2 = 82.0\%$						

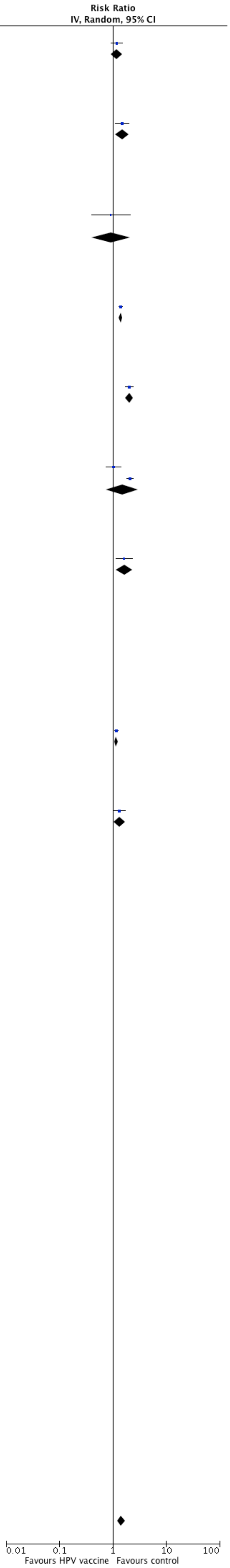

*2.20. Risk ratio for ‘solicited’ (GlaxoSmithKline): **1.37 [1.31, 1.43]**; risk ratio for ‘unsolicited’ (GlaxoSmithKline): not applicable; risk ratio for ‘systemic adverse events’ (Merck Sharp & Dohme): not applicable. To avoid double counting of participants in the total risk ratio estimate, we excluded the ‘unsolicited’ adverse events from total risk ratio estimate for studies that reported ‘solicited’ adverse events.

3. Journal publications

3.1. All-cause mortality/deaths*: intention to treat analysis

Study or Subgroup	HPV vaccine Events	HPV vaccine Total	Control Events	Control Total	Weight	Risk Ratio IV, Random, 95% CI
**3.1.1 All-cause mortality/deaths: Cervarix vs. Al(OH)3 (females, age 24-72; follow-up until 36 months)**						
HPV-015: Cervarix vs. Al(OH)3 (f, 24-72; 36)	14	2882	3	2871	20.5%	4.65 [1.34, 16.16]
**Subtotal (95% CI)**		**2882**		**2871**	**20.5%**	**4.65 [1.34, 16.16]**
Total events	14		3			
Heterogeneity: Not applicable						
Test for overall effect: Z = 2.42 (P = 0.02)						
**3.1.2 All-cause mortality/deaths: Cervarix vs. Aimmugen: hepatitis A vaccine (females, age 19-25; follow-up until 7 months)**						
HPV-032: Cervarix vs. Aimmugen (f, 19-25; 7)	0	519	0	521		Not estimable
**Subtotal (95% CI)**		**519**		**521**		**Not estimable**
Total events	0		0			
Heterogeneity: Not applicable						
Test for overall effect: Not applicable						
**3.1.3 All-cause mortality/deaths: Cervarix vs. Engerix: hepatitis B vaccine (females, age 26-46; follow-up until 12 months)**						
HPV-069: Cervarix vs. Engerix (f, 26-46; 12)	0	606	0	606		Not estimable
**Subtotal (95% CI)**		**606**		**606**		**Not estimable**
Total events	0		0			
Heterogeneity: Not applicable						
Test for overall effect: Not applicable						
**3.1.4 All-cause mortality/deaths: Cervarix vs. Havrix: hepatitis A vaccine (females, age 15-25; follow-up until 48 months)**						
HPV-008: Cervarix vs. Havrix (f, 15-25; 48)	9	9319	8	9325	25.1%	1.13 [0.43, 2.92]
**Subtotal (95% CI)**		**9319**		**9325**	**25.1%**	**1.13 [0.43, 2.92]**
Total events	9		8			
Heterogeneity: Not applicable						
Test for overall effect: Z = 0.24 (P = 0.81)						
**3.1.5 All-cause mortality/deaths: Gardasil vs. AAHS (females, age 15-26; follow-up until 45 months)**						
V501-013: Gardasil vs. AAHS (f, 16-24; 45)	2	2723	2	2732	12.4%	1.00 [0.14, 7.12]
V501-015: Gardasil vs. AAHS (f, 15-26; 36)	7	6087	5	6080	22.0%	1.40 [0.44, 4.40]
**Subtotal (95% CI)**		**8810**		**8812**	**34.4%**	**1.28 [0.48, 3.46]**
Total events	9		7			
Heterogeneity: Tau² = 0.00; Chi² = 0.08, df = 1 (P = 0.77); I² = 0%						
Test for overall effect: Z = 0.50 (P = 0.62)						
**3.1.6 All-cause mortality/deaths: Gardasil vs. AAHS (females, age 21-46; follow-up until 48 months)**						
V501-019: Gardasil vs. AAHS (f, 21-46; 48)	0	1911	0	1908		Not estimable
**Subtotal (95% CI)**		**1911**		**1908**		**Not estimable**
Total events	0		0			
Heterogeneity: Not applicable						
Test for overall effect: Not applicable						
**3.1.7 All-cause mortality/deaths: Gardasil vs. AAHS (males, age 15-27; follow-up until 36 months)**						
V501-020: Gardasil vs. AAHS (m, 15-27; 36)	3	2032	10	2033	19.9%	0.30 [0.08, 1.09]
**Subtotal (95% CI)**		**2032**		**2033**	**19.9%**	**0.30 [0.08, 1.09]**
Total events	3		10			
Heterogeneity: Not applicable						
Test for overall effect: Z = 1.83 (P = 0.07)						
**3.1.8 All-cause mortality/deaths: HPV 16 vaccine vs. AAHS (females, age 16-25; follow-up until 48 months)**						
V501-005: HPV 16 vaccine vs. AAHS (f, 16-25; 48)	0	1204	0	1205		Not estimable
**Subtotal (95% CI)**		**1204**		**1205**		**Not estimable**
Total events	0		0			
Heterogeneity: Not applicable						
Test for overall effect: Not applicable						
**Total (95% CI)**		**27283**		**27281**	**100.0%**	**1.20 [0.51, 2.80]**
Total events	35		28			
Heterogeneity: Tau² = 0.51; Chi² = 9.10, df = 4 (P = 0.06); I² = 56%						
Test for overall effect: Z = 0.41 (P = 0.68)						
Test for subgroup differences: Chi² = 9.02, df = 3 (P = 0.03), I² = 66.7%						

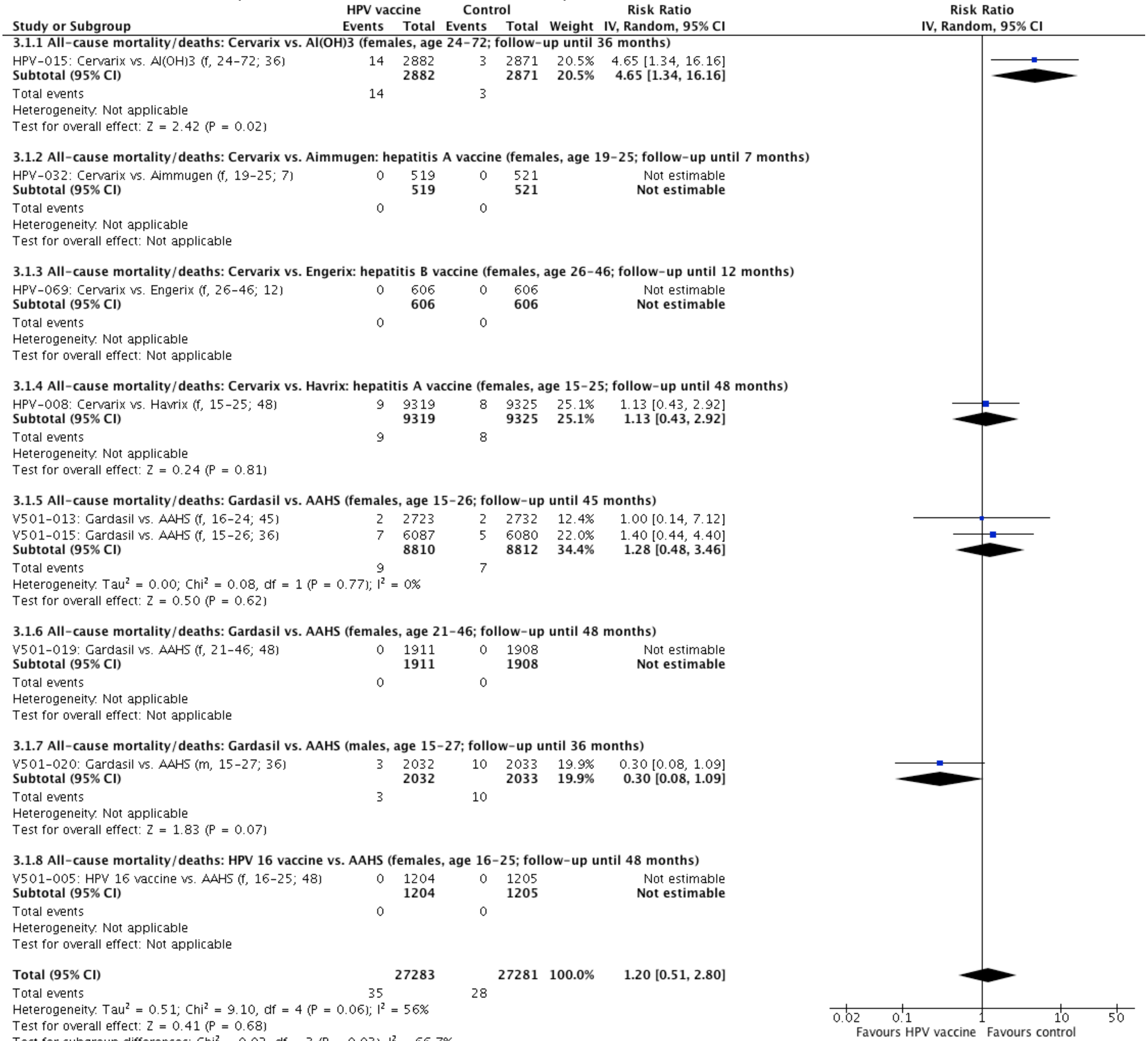

*3.1. Risk ratio for GlaxoSmithKline studies (i.e., HPV-0xx): 2.16 [0.54, 8.61]; risk ratio for Merck Sharp & Dohme studies (i.e., V50x-xxx): 0.74 [0.27, 2.05].

3.2. Mortality/deaths from HPV-related cancers (anal, cervical, oropharyngeal, penile, vaginal and vulvar) irrespective of HPV type: intention to treat analysis

Study or Subgroup	HPV vaccine Events	HPV vaccine Total	Control Events	Control Total	Weight	Risk Ratio IV, Random, 95% CI
**3.2.1 Cervical cancer: Cervarix (females, age 24-72; follow-up until 36 months)**						
HPV-015: Cervarix vs. Al(OH)3 (f, 24-72; 36)	0	2882	0	2871		Not estimable
**Subtotal (95% CI)**		**2882**		**2871**		**Not estimable**
Total events	0		0			
Heterogeneity: Not applicable						
Test for overall effect: Not applicable						
**3.2.2 Oropharyngeal cancer: Cervarix (females, age 24-72; follow-up until 36 months)**						
HPV-015: Cervarix vs. Al(OH)3 (f, 24-72; 36)	0	2882	0	2871		Not estimable
**Subtotal (95% CI)**		**2882**		**2871**		**Not estimable**
Total events	0		0			
Heterogeneity: Not applicable						
Test for overall effect: Not applicable						
**3.2.3 Oropharyngeal cancer: Gardasil (females, age 21-46; follow-up until 48 months)**						
V501-019: Gardasil vs. AAHS (f, 21-46; 48)	0	1911	0	1908		Not estimable
**Subtotal (95% CI)**		**1911**		**1908**		**Not estimable**
Total events	0		0			
Heterogeneity: Not applicable						
Test for overall effect: Not applicable						
**Total (95% CI)**		**7675**		**7650**		**Not estimable**
Total events	0		0			
Heterogeneity: Not applicable						
Test for overall effect: Not applicable						
Test for subgroup differences: Not applicable						

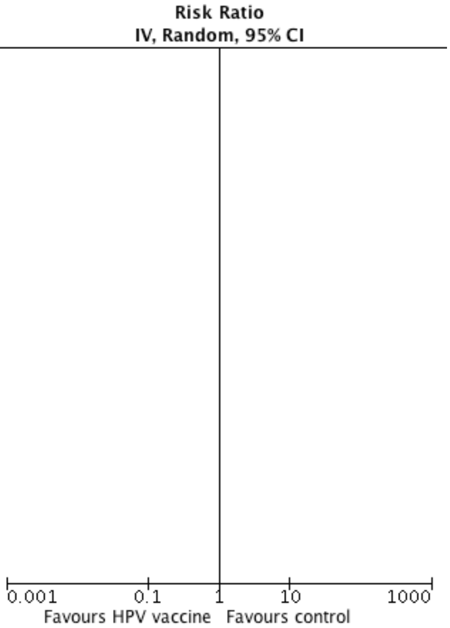

*3.2. Risk ratio for 'medically significant conditions' (GlaxoSmithKline): not applicable; risk ratio for 'new medical history' (Merck Sharp & Dohme): not applicable.

3.3. Incidence of HPV-related cancers (anal, cervical, oropharyngeal, penile, vaginal and vulvar) irrespective of HPV type*: intention to treat analysis

Study or Subgroup	HPV vaccine Events	HPV vaccine Total	Control Events	Control Total	Weight	Risk Ratio IV, Random, 95% CI
**3.3.1 Cervical cancer: Cervarix (females, age 24-72; follow-up until 36 months)**						
HPV-015: Cervarix vs. Al(OH)3 (f, 24-72; 36)	0	2882	0	2871		Not estimable
**Subtotal (95% CI)**		**2882**		**2871**		**Not estimable**
Total events	0		0			
Heterogeneity: Not applicable						
Test for overall effect: Not applicable						
**3.3.2 Cervical cancer: Gardasil (females, age 21-46; follow-up until 48 months)**						
V501-019: Gardasil vs. AAHS (f, 21-46; 48)	0	1911	0	1908		Not estimable
**Subtotal (95% CI)**		**1911**		**1908**		**Not estimable**
Total events	0		0			
Heterogeneity: Not applicable						
Test for overall effect: Not applicable						
**3.3.3 Oropharyngeal cancer: Cervarix (females, age 24-72; follow-up until 36 months)**						
HPV-015: Cervarix vs. Al(OH)3 (f, 24-72; 36)	0	2882	0	2871		Not estimable
**Subtotal (95% CI)**		**2882**		**2871**		**Not estimable**
Total events	0		0			
Heterogeneity: Not applicable						
Test for overall effect: Not applicable						
**3.3.4 Oropharyngeal cancer: Gardasil (females, age 21-46; follow-up until 48 months)**						
V501-019: Gardasil vs. AAHS (f, 21-46; 48)	0	1911	0	1908		Not estimable
**Subtotal (95% CI)**		**1911**		**1908**		**Not estimable**
Total events	0		0			
Heterogeneity: Not applicable						
Test for overall effect: Not applicable						
**3.3.5 Vaginal cancer: Cervarix (females, age 24-72; follow-up until 36 months)**						
HPV-015: Cervarix vs. Al(OH)3 (f, 24-72; 36)	0	2882	0	2871		Not estimable
**Subtotal (95% CI)**		**2882**		**2871**		**Not estimable**
Total events	0		0			
Heterogeneity: Not applicable						
Test for overall effect: Not applicable						
**3.3.6 Vulvar cancer: Cervarix (females, age 15-25; follow-up until 48 months)**						
HPV-008: Cervarix vs. Havrix (f, 15-25; 48)	0	9319	0	9325		Not estimable
**Subtotal (95% CI)**		**9319**		**9325**		**Not estimable**
Total events	0		0			
Heterogeneity: Not applicable						
Test for overall effect: Not applicable						
**3.3.7 Vulvar cancer: Gardasil (females, age 15-26; follow-up until 45 months)**						
V501-013: Gardasil vs. AAHS (f, 16-24; 45)	1	2723	0	2732	100.0%	3.01 [0.12, 73.85]
**Subtotal (95% CI)**		**2723**		**2732**	**100.0%**	**3.01 [0.12, 73.85]**
Total events	1		0			
Heterogeneity: Not applicable						
Test for overall effect: Z = 0.67 (P = 0.50)						
**Total (95% CI)**		**24510**		**24486**	**100.0%**	**3.01 [0.12, 73.85]**
Total events	1		0			
Heterogeneity: Not applicable						
Test for overall effect: Z = 0.67 (P = 0.50)						
Test for subgroup differences: Not applicable						

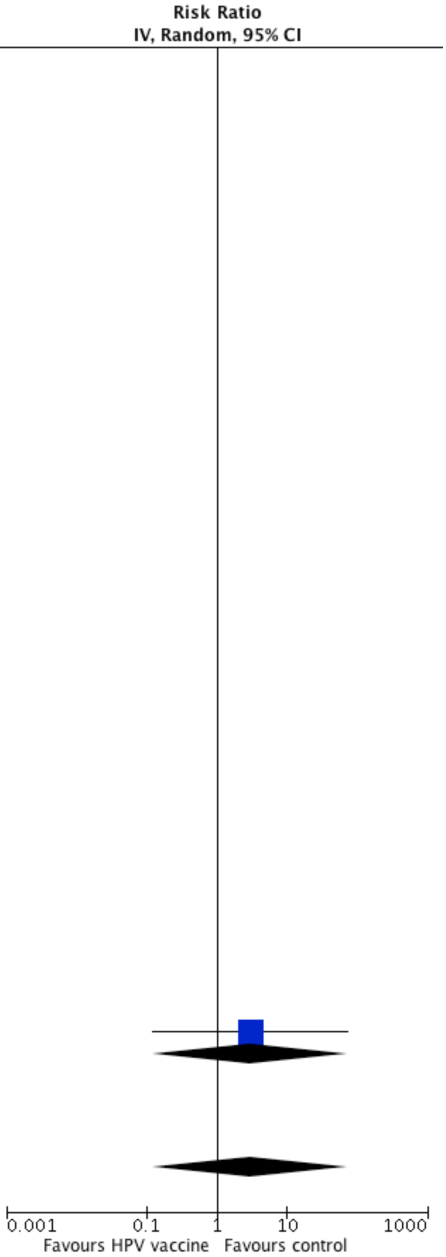

*3.3. Risk ratio for GlaxoSmithKline studies (i.e., HPV-0xx): not applicable; risk ratio for Merck Sharp & Dohme studies (i.e., V50x-xxx): 3.01 [0.12, 73.85].

3.4. Incidence of HPV-related carcinoma in situ (anal intraepithelial neoplasia grade 3 [AIN3], cervical adenocarcinoma in situ [AIS], cervical intraepithelial neoplasia grade 3 [CIN3], penile intraepithelial neoplasia grade 3 [PIN3], vaginal intraepithelial neoplasia grade 3 [VIN3] and vulvar intraepithelial neoplasia grade 3 [VaIN3]) irrespective of HPV type*: intention to treat analysis

	HPV vaccine		Control			Risk Ratio
**Study or Subgroup**	**Events**	**Total**	**Events**	**Total**	**Weight**	**IV, Random, 95% CI**
**3.4.1 AIS: Cervarix (females, age 15-25; follow-up until 48 months)**						
HPV-008: Cervarix vs. Havrix (f, 15-25; 48)	0	9319	0	9325		Not estimable
**Subtotal (95% CI)**		**9319**		**9325**		**Not estimable**
Total events	0		0			
Heterogeneity: Not applicable						
Test for overall effect: Not applicable						
**3.4.2 AIS: Gardasil (females, age 15-26; follow-up until 45 months)**						
V501-013: Gardasil vs. AAHS (f, 16-24; 45)	1	2723	6	2732	2.4%	0.17 [0.02, 1.39]
V501-015: Gardasil vs. AAHS (f, 15-26; 36)	5	6087	8	6080	7.8%	0.62 [0.20, 1.91]
**Subtotal (95% CI)**		**8810**		**8812**	**10.2%**	**0.44 [0.14, 1.38]**
Total events	6		14			
Heterogeneity: $Tau^2 = 0.12$; $Chi^2 = 1.16$, df = 1 (P = 0.28); $I^2 = 14\%$						
Test for overall effect: Z = 1.41 (P = 0.16)						
**3.4.3 AIS: Gardasil (females, age 21-46; follow-up until 48 months)**						
V501-019: Gardasil vs. AAHS (f, 21-46; 48)	0	1911	0	1908		Not estimable
**Subtotal (95% CI)**		**1911**		**1908**		**Not estimable**
Total events	0		0			
Heterogeneity: Not applicable						
Test for overall effect: Not applicable						
**3.4.4 CIN3: Cervarix (females, age 15-25; follow-up until 48 months)**						
HPV-008: Cervarix vs. Havrix (f, 15-25; 48)	0	9319	0	9325		Not estimable
**Subtotal (95% CI)**		**9319**		**9325**		**Not estimable**
Total events	0		0			
Heterogeneity: Not applicable						
Test for overall effect: Not applicable						
**3.4.5 CIN3: Gardasil (females, age 15-26; follow-up until 45 months)**						
V501-013: Gardasil vs. AAHS (f, 16-24; 45)	79	2723	72	2732	40.7%	1.10 [0.80, 1.51]
V501-015: Gardasil vs. AAHS (f, 15-26; 36)	127	6087	161	6080	49.1%	0.79 [0.63, 0.99]
**Subtotal (95% CI)**		**8810**		**8812**	**89.8%**	**0.91 [0.66, 1.27]**
Total events	206		233			
Heterogeneity: $Tau^2 = 0.04$; $Chi^2 = 2.83$, df = 1 (P = 0.09); $I^2 = 65\%$						
Test for overall effect: Z = 0.54 (P = 0.59)						
**3.4.6 CIN3: Gardasil (females, age 21-46; follow-up until 48 months)**						
V501-019: Gardasil vs. AAHS (f, 21-46; 48)	0	1911	0	1908		Not estimable
**Subtotal (95% CI)**		**1911**		**1908**		**Not estimable**
Total events	0		0			
Heterogeneity: Not applicable						
Test for overall effect: Not applicable						
**Total (95% CI)**		**40080**		**40090**	**100.0%**	**0.85 [0.61, 1.19]**
Total events	212		247			
Heterogeneity: $Tau^2 = 0.04$; $Chi^2 = 5.53$, df = 3 (P = 0.14); $I^2 = 46\%$						
Test for overall effect: Z = 0.93 (P = 0.35)						
Test for subgroup differences: $Chi^2 = 1.45$, df = 1 (P = 0.23), $I^2 = 30.9\%$						

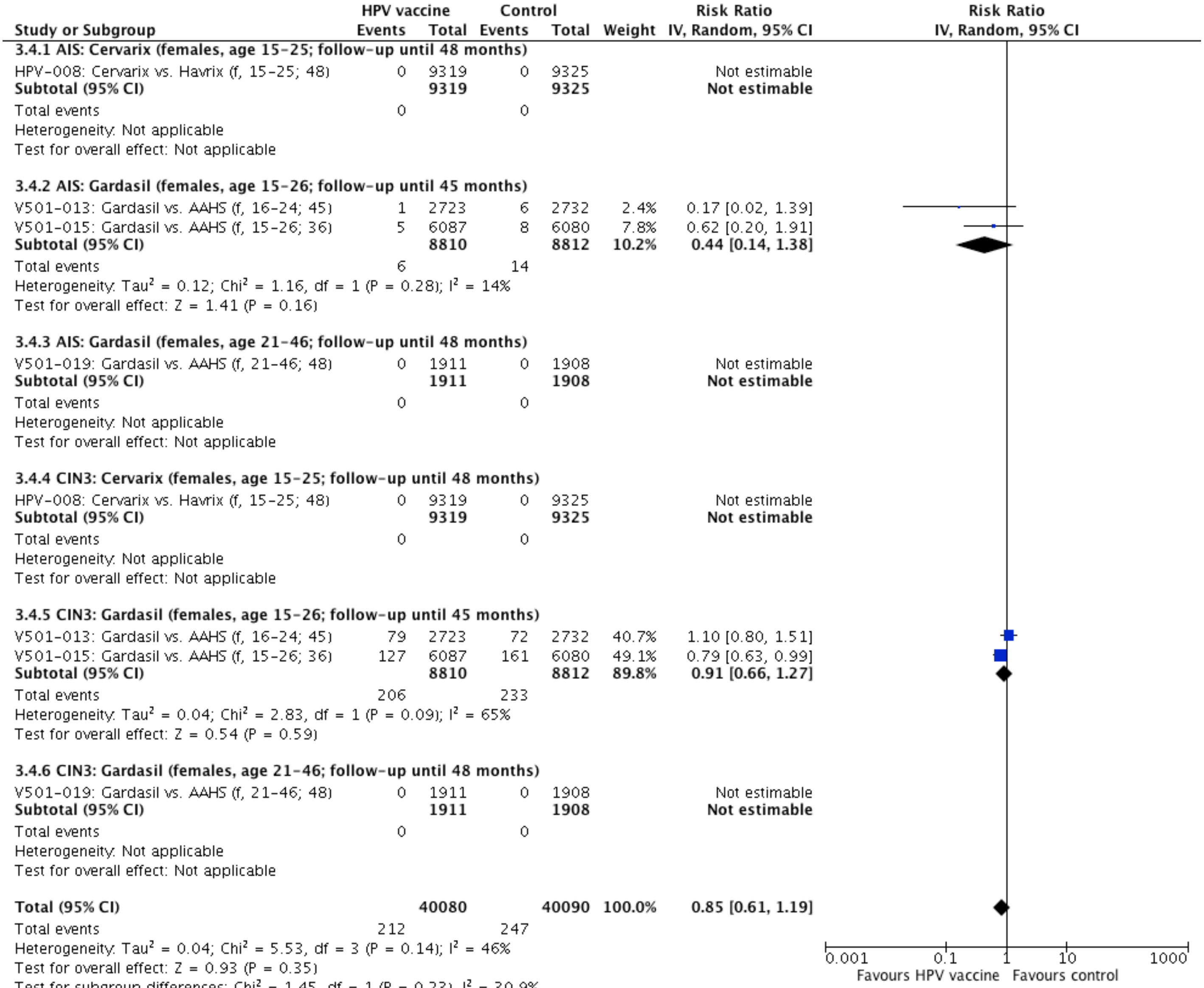

*3.4. Risk ratio for GlaxoSmithKline studies (i.e., HPV-0xx): not applicable; risk ratio for Merck Sharp & Dohme studies (i.e., V50x-xxx): 0.85 [0.61, 1.19].

3.5. Incidence of HPV-related moderate intraepithelial neoplasia (anal intraepithelial neoplasia grade 2 [AIN2], cervical intraepithelial neoplasia grade 2 [CIN2], penile intraepithelial neoplasia grade 2 [PIN2], vaginal intraepithelial neoplasia grade 2 [VIN2] and vulvar intraepithelial neoplasia grade 2 [VaIN2]) irrespective of HPV type*: intention to treat analysis

Study or Subgroup	HPV vaccine Events	HPV vaccine Total	Control Events	Control Total	Weight	Risk Ratio IV, Random, 95% CI
**3.5.1 CIN2: Cervarix (females, age 15-25; follow-up until 48 months)**						
HPV-008: Cervarix vs. Havrix (f, 15-25; 48)	0	9319	0	9325		Not estimable
**Subtotal (95% CI)**		**9319**		**9325**		**Not estimable**
Total events	0		0			
Heterogeneity: Not applicable						
Test for overall effect: Not applicable						
**3.5.2 CIN2: Gardasil (females, age 15-26; follow-up until 45 months)**						
V501-013: Gardasil vs. AAHS (f, 16-24; 45)	102	2723	116	2732	39.6%	0.88 [0.68, 1.14]
V501-015: Gardasil vs. AAHS (f, 15-26; 36)	149	6087	192	6080	60.4%	0.78 [0.63, 0.96]
**Subtotal (95% CI)**		**8810**		**8812**	**100.0%**	**0.82 [0.69, 0.96]**
Total events	251		308			
Heterogeneity: $Tau^2 = 0.00$; $Chi^2 = 0.57$, df = 1 (P = 0.45); $I^2 = 0\%$						
Test for overall effect: Z = 2.43 (P = 0.02)						
**3.5.3 CIN2: Gardasil (females, age 21-46; follow-up until 48 months)**						
V501-019: Gardasil vs. AAHS (f, 21-46; 48)	0	1911	0	1908		Not estimable
**Subtotal (95% CI)**		**1911**		**1908**		**Not estimable**
Total events	0		0			
Heterogeneity: Not applicable						
Test for overall effect: Not applicable						
**Total (95% CI)**		**20040**		**20045**	**100.0%**	**0.82 [0.69, 0.96]**
Total events	251		308			
Heterogeneity: $Tau^2 = 0.00$; $Chi^2 = 0.57$, df = 1 (P = 0.45); $I^2 = 0\%$						
Test for overall effect: Z = 2.43 (P = 0.02)						
Test for subgroup differences: Not applicable						

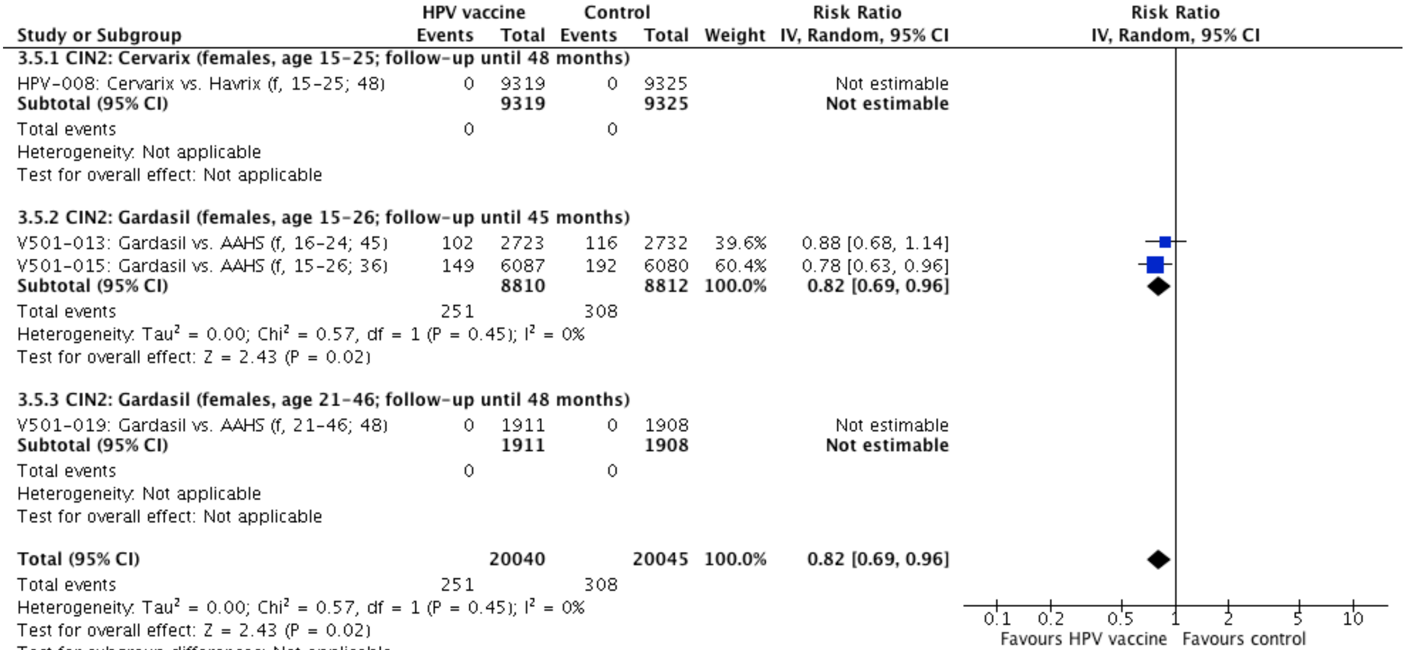

*3.5. Risk ratio for GlaxoSmithKline studies (i.e., HPV-0xx): not applicable; risk ratio for Merck Sharp & Dohme studies (i.e., V50x-xxx): 0.82 [0.69, 0.96]. There were no reports of AIN2, PIN2, VIN2 or VaIN2 irrespective of HPV type.

3.6. Incidence of HPV-related moderate intraepithelial neoplasia or worse (AIN2⁺, CIN2⁺, PIN2⁺, VIN2⁺, VaIN2⁺) irrespective of HPV type*: intention to treat analysis

Study or Subgroup	HPV vaccine Events	HPV vaccine Total	Control Events	Control Total	Weight	Risk Ratio IV, Random, 95% CI
**3.6.1 CIN2+: Cervarix (females, age 15-25; follow-up until 48 months)**						
HPV-008: Cervarix vs. Havrix (f, 15-25; 48)	224	9319	322	9325	29.2%	0.70 [0.59, 0.82]
**Subtotal (95% CI)**		**9319**		**9325**	**29.2%**	**0.70 [0.59, 0.82]**
Total events	224		322			
Heterogeneity: Not applicable						
Test for overall effect: Z = 4.22 (P < 0.0001)						
**3.6.2 CIN2+: Cervarix (females, age 22-29; follow-up until 12 months)**						
HPV-063: Cervarix vs. Aimmugen (f, 22-29; 12)	19	375	41	377	8.4%	0.47 [0.28, 0.79]
**Subtotal (95% CI)**		**375**		**377**	**8.4%**	**0.47 [0.28, 0.79]**
Total events	19		41			
Heterogeneity: Not applicable						
Test for overall effect: Z = 2.85 (P = 0.004)						
**3.6.3 CIN2+: Cervarix (females, age 24-72; follow-up until 36 months)**						
HPV-015: Cervarix vs. Al(OH)3 (f, 24-72; 36)	0	2882	0	2871		Not estimable
**Subtotal (95% CI)**		**2882**		**2871**		**Not estimable**
Total events	0		0			
Heterogeneity: Not applicable						
Test for overall effect: Not applicable						
**3.6.4 CIN2+: Gardasil (females, age 15-26; follow-up until 45 months)**						
V501-013: Gardasil vs. AAHS (f, 16-24; 45)	183	2723	194	2732	26.6%	0.95 [0.78, 1.15]
V501-015: Gardasil vs. AAHS (f, 15-26; 36)	219	6087	266	6080	28.5%	0.82 [0.69, 0.98]
**Subtotal (95% CI)**		**8810**		**8812**	**55.1%**	**0.88 [0.76, 1.01]**
Total events	402		460			
Heterogeneity: Tau² = 0.00; Chi² = 1.10, df = 1 (P = 0.29); I² = 9%						
Test for overall effect: Z = 1.89 (P = 0.06)						
**3.6.5 CIN2+: Gardasil (females, age 21-46; follow-up until 48 months)**						
V501-019: Gardasil vs. AAHS (f, 21-46; 48)	0	1911	0	1908		Not estimable
**Subtotal (95% CI)**		**1911**		**1908**		**Not estimable**
Total events	0		0			
Heterogeneity: Not applicable						
Test for overall effect: Not applicable						
**3.6.6 PIN2+: Gardasil (males, age 15-27; follow-up until 36 months)**						
V501-020: Gardasil vs. AAHS (m, 15-27; 36)	3	2032	2	2033	0.9%	1.50 [0.25, 8.97]
**Subtotal (95% CI)**		**2032**		**2033**	**0.9%**	**1.50 [0.25, 8.97]**
Total events	3		2			
Heterogeneity: Not applicable						
Test for overall effect: Z = 0.44 (P = 0.66)						
**3.6.7 VIN2+ or VaIN2+: Gardasil (females, age 15-26; follow-up until 45 months)**						
V501-013: Gardasil vs. AAHS (f, 16-24; 45)	17	2723	23	2732	6.3%	0.74 [0.40, 1.38]
V501-015: Gardasil vs. AAHS (f, 15-26; 36)	0	6087	0	6080		Not estimable
**Subtotal (95% CI)**		**8810**		**8812**	**6.3%**	**0.74 [0.40, 1.38]**
Total events	17		23			
Heterogeneity: Not applicable						
Test for overall effect: Z = 0.94 (P = 0.35)						
**3.6.8 VaIN2+: Gardasil (females, age 15-26; follow-up until 45 months)**						
V501-013: Gardasil vs. AAHS (f, 16-24; 45)	0	2723	0	2732		Not estimable
V501-015: Gardasil vs. AAHS (f, 15-26; 36)	0	6087	0	6080		Not estimable
**Subtotal (95% CI)**		**8810**		**8812**		**Not estimable**
Total events	0		0			
Heterogeneity: Not applicable						
Test for overall effect: Not applicable						
**3.6.9 VaIN2+: Gardasil (females, age 21-46; follow-up until 48 months)**						
V501-019: Gardasil vs. AAHS (f, 21-46; 48)	0	1911	0	1908		Not estimable
**Subtotal (95% CI)**		**1911**		**1908**		**Not estimable**
Total events	0		0			
Heterogeneity: Not applicable						
Test for overall effect: Not applicable						
**3.6.10 VIN2+ or VaIN2+: Cervarix (females, age 15-25; follow-up until 48 months)**						
HPV-008: Cervarix vs. Havrix (f, 15-25; 48)	0	9319	0	9325		Not estimable
**Subtotal (95% CI)**		**9319**		**9325**		**Not estimable**
Total events	0		0			
Heterogeneity: Not applicable						
Test for overall effect: Not applicable						
**Total (95% CI)**		**54179**		**54183**	**100.0%**	**0.77 [0.65, 0.92]**
Total events	665		848			
Heterogeneity: Tau² = 0.02; Chi² = 10.10, df = 5 (P = 0.07); I² = 50%						
Test for overall effect: Z = 2.93 (P = 0.003)						
Test for subgroup differences: Chi² = 8.77, df = 4 (P = 0.07), I² = 54.4%						

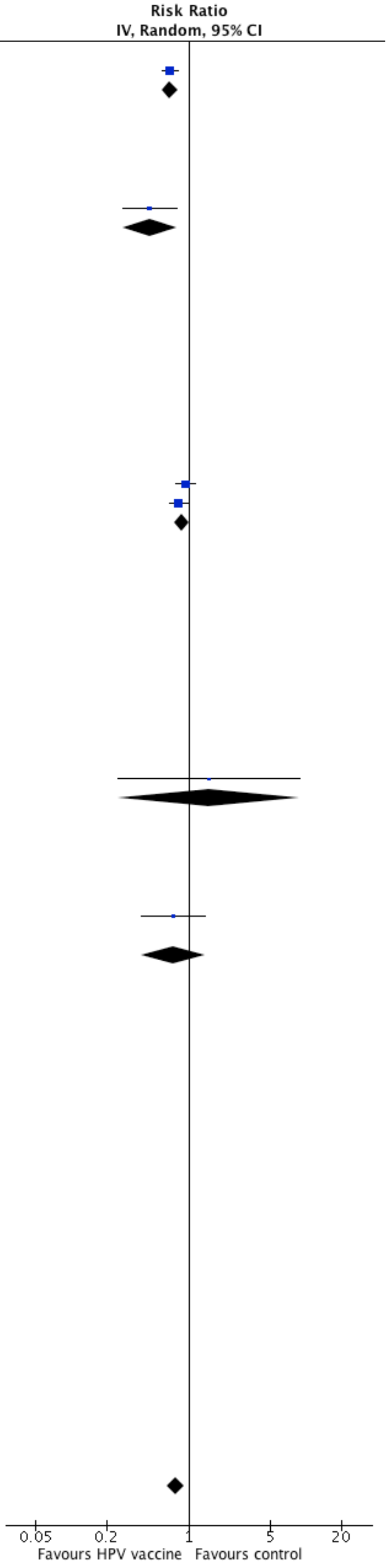

*3.6. Risk ratio for GlaxoSmithKline studies (i.e., HPV-0xx): 0.62 [0.43, 0.89]; risk ratio for Merck Sharp & Dohme studies (i.e., V50x-xxx): 0.87 [0.77, 0.99].

3.7. Number of treatment procedures (both surgical and non-surgical treatment) due to HPV-related diseases irrespective of HPV type*: intention to treat analysis

Study or Subgroup	HPV vaccine Events	HPV vaccine Total	Control Events	Control Total	Weight	Risk Ratio IV, Random, 95% CI
**3.7.1 Treatment procedures: Cervarix (females, age 15-25; follow-up until 48 months)**						
HPV-008: Cervarix vs. Havrix (f, 15-25; 48)	180	9319	240	9325	100.0%	0.75 [0.62, 0.91]
**Subtotal (95% CI)**		**9319**		**9325**	**100.0%**	**0.75 [0.62, 0.91]**
Total events	180		240			
Heterogeneity: Not applicable						
Test for overall effect: Z = 2.94 (P = 0.003)						
**3.7.2 Treatment procedures: Cervarix (females, age 24-72; follow-up until 36 months)**						
HPV-015: Cervarix vs. Al(OH)3 (f, 24-72; 36)	0	2882	0	2871		Not estimable
**Subtotal (95% CI)**		**2882**		**2871**		**Not estimable**
Total events	0		0			
Heterogeneity: Not applicable						
Test for overall effect: Not applicable						
**3.7.3 Treatment procedures: Gardasil (females, age 15-26; follow-up until 45 months)**						
V501-013: Gardasil vs. AAHS (f, 16-24; 45)	0	2723	0	2732		Not estimable
V501-015: Gardasil vs. AAHS (f, 15-26; 36)	0	6087	0	6080		Not estimable
**Subtotal (95% CI)**		**8810**		**8812**		**Not estimable**
Total events	0		0			
Heterogeneity: Not applicable						
Test for overall effect: Not applicable						
**3.7.4 Treatment procedures: Gardasil (females, age 21-46; follow-up until 48 months)**						
V501-019: Gardasil vs. AAHS (f, 21-46; 48)	0	1911	0	1908		Not estimable
**Subtotal (95% CI)**		**1911**		**1908**		**Not estimable**
Total events	0		0			
Heterogeneity: Not applicable						
Test for overall effect: Not applicable						
**3.7.5 Treatment procedures: Gardasil (males, age 15-27; follow-up until 36 months)**						
V501-020: Gardasil vs. AAHS (m, 15-27; 36)	0	2032	0	2033		Not estimable
**Subtotal (95% CI)**		**2032**		**2033**		**Not estimable**
Total events	0		0			
Heterogeneity: Not applicable						
Test for overall effect: Not applicable						
**Total (95% CI)**		**24954**		**24949**	**100.0%**	**0.75 [0.62, 0.91]**
Total events	180		240			
Heterogeneity: Not applicable						
Test for overall effect: Z = 2.94 (P = 0.003)						
Test for subgroup differences: Not applicable						

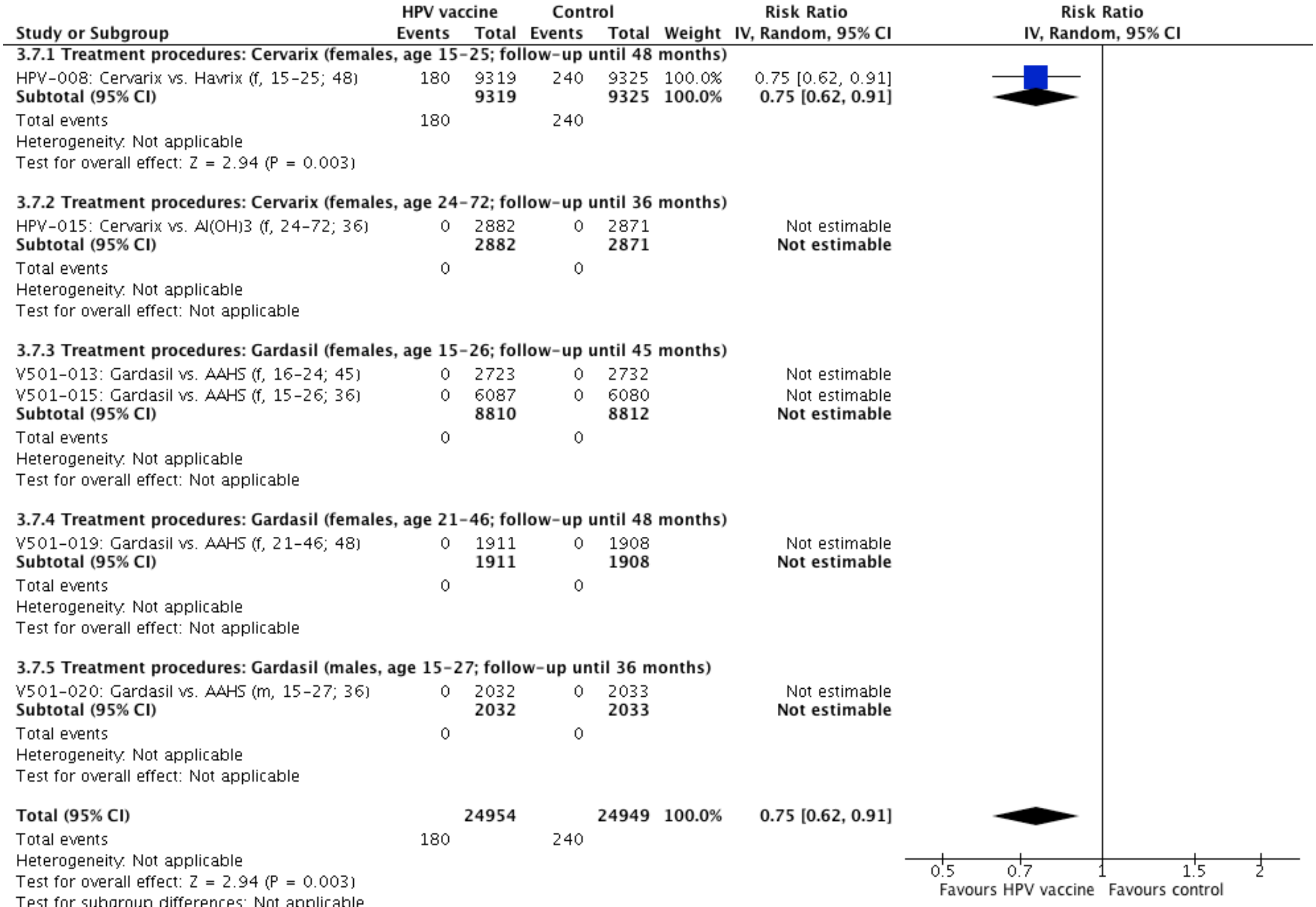

*3.7. Risk ratio for GlaxoSmithKline studies (i.e., HPV-0xx): **0.75 [0.62, 0.91]**; risk ratio for Merck Sharp & Dohme studies (i.e., V50x-xxx): not applicable.
`

3.8. Fatal harms*: intention to treat analysis

Study or Subgroup	HPV vaccine Events	HPV vaccine Total	Control Events	Control Total	Weight	Risk Ratio IV, Random, 95% CI
**3.8.1 Fatal harms: Cervarix vs. Al(OH)3 (females, age 24-72; follow-up until 36 months)**						
HPV-015: Cervarix vs. Al(OH)3 (f, 24-72; 36)	14	2882	3	2871	20.5%	4.65 [1.34, 16.16]
**Subtotal (95% CI)**		**2882**		**2871**	**20.5%**	**4.65 [1.34, 16.16]**
Total events	14		3			
Heterogeneity: Not applicable						
Test for overall effect: Z = 2.42 (P = 0.02)						
**3.8.2 Fatal harms: Cervarix vs. Aimmugen: hepatitis A vaccine (females, age 19-25; follow-up until 7 months)**						
HPV-032: Cervarix vs. Aimmugen (f, 19-25; 7)	0	519	0	521		Not estimable
HPV-063: Cervarix vs. Aimmugen (f, 22-29; 12)	0	375	0	377		Not estimable
**Subtotal (95% CI)**		**894**		**898**		**Not estimable**
Total events	0		0			
Heterogeneity: Not applicable						
Test for overall effect: Not applicable						
**3.8.3 Fatal harms: Cervarix vs. Engerix: hepatitis B vaccine (females, age 26-46; follow-up until 12 months)**						
HPV-069: Cervarix vs. Engerix (f, 26-46; 12)	0	606	0	606		Not estimable
**Subtotal (95% CI)**		**606**		**606**		**Not estimable**
Total events	0		0			
Heterogeneity: Not applicable						
Test for overall effect: Not applicable						
**3.8.4 Fatal harms: Cervarix vs. Havrix: hepatitis A vaccine (females, age 15-25; follow-up until 48 months)**						
HPV-008: Cervarix vs. Havrix (f, 15-25; 48)	9	9319	8	9325	25.1%	1.13 [0.43, 2.92]
**Subtotal (95% CI)**		**9319**		**9325**	**25.1%**	**1.13 [0.43, 2.92]**
Total events	9		8			
Heterogeneity: Not applicable						
Test for overall effect: Z = 0.24 (P = 0.81)						
**3.8.5 Fatal harms: Gardasil vs. AAHS (females, age 15-26; follow-up until 45 months)**						
V501-013: Gardasil vs. AAHS (f, 16-24; 45)	2	2723	2	2732	12.4%	1.00 [0.14, 7.12]
V501-015: Gardasil vs. AAHS (f, 15-26; 36)	7	6087	5	6080	22.0%	1.40 [0.44, 4.40]
**Subtotal (95% CI)**		**8810**		**8812**	**34.4%**	**1.28 [0.48, 3.46]**
Total events	9		7			
Heterogeneity: $Tau^2 = 0.00$; $Chi^2 = 0.08$, df = 1 (P = 0.77); $I^2 = 0\%$						
Test for overall effect: Z = 0.50 (P = 0.62)						
**3.8.6 Fatal harms: Gardasil vs. AAHS (females, age 21-46; follow-up until 48 months)**						
V501-019: Gardasil vs. AAHS (f, 21-46; 48)	0	1911	0	1908		Not estimable
**Subtotal (95% CI)**		**1911**		**1908**		**Not estimable**
Total events	0		0			
Heterogeneity: Not applicable						
Test for overall effect: Not applicable						
**3.8.7 Fatal harms: Gardasil vs. AAHS (males, age 15-27; follow-up until 36 months)**						
V501-020: Gardasil vs. AAHS (m, 15-27; 36)	3	2032	10	2033	19.9%	0.30 [0.08, 1.09]
**Subtotal (95% CI)**		**2032**		**2033**	**19.9%**	**0.30 [0.08, 1.09]**
Total events	3		10			
Heterogeneity: Not applicable						
Test for overall effect: Z = 1.83 (P = 0.07)						
**3.8.8 Fatal harms: HPV 16 vaccine vs. AAHS (females, age 16-25; follow-up until 48 months)**						
V501-005: HPV 16 vaccine vs. AAHS (f, 16-25; 48)	0	1204	0	1205		Not estimable
**Subtotal (95% CI)**		**1204**		**1205**		**Not estimable**
Total events	0		0			
Heterogeneity: Not applicable						
Test for overall effect: Not applicable						
**Total (95% CI)**		**27658**		**27658**	**100.0%**	**1.20 [0.51, 2.80]**
Total events	35		28			
Heterogeneity: $Tau^2 = 0.51$; $Chi^2 = 9.10$, df = 4 (P = 0.06); $I^2 = 56\%$						
Test for overall effect: Z = 0.41 (P = 0.68)						
Test for subgroup differences: $Chi^2 = 9.02$, df = 3 (P = 0.03), $I^2 = 66.7\%$						

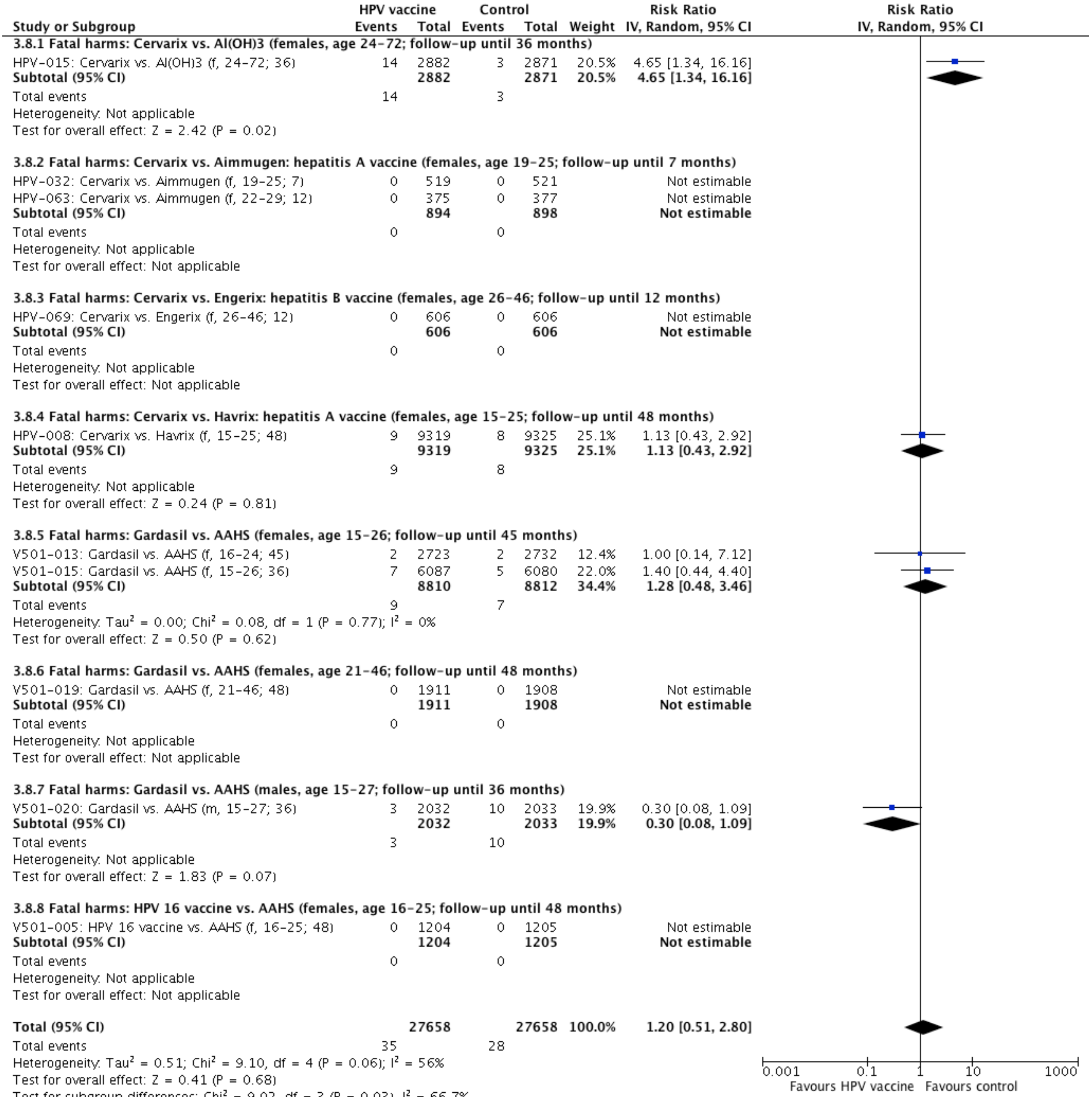

*3.8. Risk ratio for GlaxoSmithKline studies (i.e., HPV-0xx): 2.16 [0.54, 8.61]; risk ratio for Merck Sharp & Dohme studies (i.e., V50x-xxx): 0.74 [0.27, 2.05].

3.9. Serious harms*: intention to treat analysis

Study or Subgroup	HPV vaccine Events	HPV vaccine Total	Control Events	Control Total	Weight	Risk Ratio IV, Random, 95% CI
**3.9.1 Serious harms: Cervarix vs. Al(OH)3 (females, age 9-17; follow-up until 12 months)**						
HPV-058: Cervarix vs. Al(OH)3 (f, 9-17; 12)	5	374	2	376	0.2%	2.51 [0.49, 12.87]
**Subtotal (95% CI)**		**374**		**376**	**0.2%**	**2.51 [0.49, 12.87]**
Total events	5		2			
Heterogeneity: Not applicable						
Test for overall effect: Z = 1.11 (P = 0.27)						
**3.9.2 Serious harms: Cervarix vs. Al(OH)3 (females, age 15-25; follow-up until 7 months)**						
HPV-001: Cervarix vs. Al(OH)3 (f, 15-26; 27)	22	560	19	553	1.6%	1.14 [0.63, 2.09]
HPV-038: Cervarix vs. Al(OH)3 (f, 15-25; 7)	2	149	1	76	0.1%	1.02 [0.09, 11.07]
**Subtotal (95% CI)**		**709**		**629**	**1.7%**	**1.14 [0.63, 2.04]**
Total events	24		20			
Heterogeneity: Tau² = 0.00; Chi² = 0.01, df = 1 (P = 0.93); I² = 0%						
Test for overall effect: Z = 0.43 (P = 0.67)						
**3.9.3 Serious harms: Cervarix vs. Al(OH)3 (females, age 18-35; follow-up until 12 months)**						
HPV-003: Cervarix vs. Al(OH)3 (f, 18-30; 12)	0	31	0	30		Not estimable
HPV-023: Cervarix vs. Al(OH)3 (f, 21-32; 36)	20	222	11	211	1.2%	1.73 [0.85, 3.52]
HPV-031: Cervarix vs. Al(OH)3 (f, 18-35; 7)	2	176	4	178	0.2%	0.51 [0.09, 2.73]
HPV-035: Cervarix vs. Al(OH)3 (f, 18-35; 7)	3	150	1	150	0.1%	3.00 [0.32, 28.52]
**Subtotal (95% CI)**		**579**		**569**	**1.5%**	**1.49 [0.75, 2.97]**
Total events	25		16			
Heterogeneity: Tau² = 0.03; Chi² = 2.11, df = 2 (P = 0.35); I² = 5%						
Test for overall effect: Z = 1.14 (P = 0.25)						
**3.9.4 Serious harms: Cervarix vs. Al(OH)3 (females, age 24-72; follow-up until 36 months)**						
HPV-015: Cervarix vs. Al(OH)3 (f, 24-72; 36)	285	2882	267	2871	23.1%	1.06 [0.91, 1.25]
**Subtotal (95% CI)**		**2882**		**2871**	**23.1%**	**1.06 [0.91, 1.25]**
Total events	285		267			
Heterogeneity: Not applicable						
Test for overall effect: Z = 0.76 (P = 0.45)						
**3.9.5 Serious harms: Cervarix vs. Aimmugen: hepatitis A vaccine (females, age 22-29; follow-up until 12 months)**						
HPV-032: Cervarix vs. Aimmugen (f, 19-25; 7)	18	519	19	521	1.5%	0.95 [0.50, 1.79]
HPV-063: Cervarix vs. Aimmugen (f, 22-29; 12)	26	375	34	377	2.4%	0.77 [0.47, 1.26]
**Subtotal (95% CI)**		**894**		**898**	**3.9%**	**0.83 [0.57, 1.23]**
Total events	44		53			
Heterogeneity: Tau² = 0.00; Chi² = 0.27, df = 1 (P = 0.60); I² = 0%						
Test for overall effect: Z = 0.93 (P = 0.35)						
**3.9.6 Serious harms: Cervarix vs. Engerix: hepatitis B vaccine (females and males, age 9-16; follow-up until 72 months)**						
HPV-030: Cervarix vs. Engerix (f, 9-15; 12)	2	247	1	246	0.1%	1.99 [0.18, 21.82]
HPV-040: Cervarix vs. Engerix (f & m, 12-16; 72)	8	643	21	1047	0.9%	0.62 [0.28, 1.39]
**Subtotal (95% CI)**		**890**		**1293**	**1.0%**	**0.70 [0.32, 1.50]**
Total events	10		22			
Heterogeneity: Tau² = 0.00; Chi² = 0.82, df = 1 (P = 0.37); I² = 0%						
Test for overall effect: Z = 0.92 (P = 0.36)						
**3.9.7 Serious harms: Cervarix vs. Engerix: hepatitis B vaccine (females, age 26-46; follow-up until 12 months)**						
HPV-069: Cervarix vs. Engerix (f, 26-46; 12)	3	606	3	606	0.2%	1.00 [0.20, 4.93]
**Subtotal (95% CI)**		**606**		**606**	**0.2%**	**1.00 [0.20, 4.93]**
Total events	3		3			
Heterogeneity: Not applicable						
Test for overall effect: Z = 0.00 (P = 1.00)						
**3.9.8 Serious harms: Cervarix vs. Havrix: hepatitis A vaccine (females, age 9-15; follow-up until 12 months)**						
HPV-013: Cervarix vs. Havrix (f, 10-14; 12)	24	1035	23	1032	1.8%	1.04 [0.59, 1.83]
HPV-033: Cervarix vs. Havrix (f, 9-15; 7)	0	160	1	161	0.1%	0.34 [0.01, 8.17]
**Subtotal (95% CI)**		**1195**		**1193**	**1.9%**	**1.01 [0.58, 1.75]**
Total events	24		24			
Heterogeneity: Tau² = 0.00; Chi² = 0.47, df = 1 (P = 0.49); I² = 0%						
Test for overall effect: Z = 0.02 (P = 0.99)						
**3.9.9 Serious harms: Cervarix vs. Havrix: hepatitis A vaccine (females, age 15-25; follow-up until 48 months)**						
HPV-008: Cervarix vs. Havrix (f, 15-25; 48)	701	9319	699	9325	57.3%	1.00 [0.91, 1.11]
**Subtotal (95% CI)**		**9319**		**9325**	**57.3%**	**1.00 [0.91, 1.11]**
Total events	701		699			
Heterogeneity: Not applicable						
Test for overall effect: Z = 0.07 (P = 0.95)						
**3.9.10 Serious harms: Cervarix vs. Twinrix: hepatitis A and B vaccine (females, age 8-15; follow-up until 12 months)**						
HPV-029: Cervarix vs. Twinrix (f, 8-15; 12)	4	270	5	271	0.3%	0.80 [0.22, 2.96]
**Subtotal (95% CI)**		**270**		**271**	**0.3%**	**0.80 [0.22, 2.96]**
Total events	4		5			
Heterogeneity: Not applicable						
Test for overall effect: Z = 0.33 (P = 0.74)						
**3.9.11 Serious harms: Gardasil vs. carrier solution (females, age 9-16; follow-up until 18 months)**						
V501-018: Gardasil vs. carrier solu. (f&m,9-16;18)	5	1184	0	597	0.1%	5.55 [0.31, 100.22]
**Subtotal (95% CI)**		**1184**		**597**	**0.1%**	**5.55 [0.31, 100.22]**
Total events	5		0			
Heterogeneity: Not applicable						
Test for overall effect: Z = 1.16 (P = 0.25)						
**3.9.12 Serious harms: Gardasil vs. AAHS (females, age 15-26; follow-up until 45 months)**						
V501-013: Gardasil vs. AAHS (f, 16-24; 45)	48	2723	45	2732	3.6%	1.07 [0.72, 1.60]
V501-015: Gardasil vs. AAHS (f, 15-26; 36)	45	6087	54	6080	3.7%	0.83 [0.56, 1.23]
**Subtotal (95% CI)**		**8810**		**8812**	**7.3%**	**0.94 [0.71, 1.25]**
Total events	93		99			
Heterogeneity: Tau² = 0.00; Chi² = 0.76, df = 1 (P = 0.38); I² = 0%						
Test for overall effect: Z = 0.42 (P = 0.67)						
**3.9.13 Serious harms: Gardasil vs. AAHS (females, age 21-46; follow-up until 48 months)**						
V501-019: Gardasil vs. AAHS (f, 21-46; 48)	3	1911	7	1908	0.3%	0.43 [0.11, 1.65]
**Subtotal (95% CI)**		**1911**		**1908**	**0.3%**	**0.43 [0.11, 1.65]**
Total events	3		7			
Heterogeneity: Not applicable						
Test for overall effect: Z = 1.23 (P = 0.22)						
**3.9.14 Serious harms: Gardasil vs. AAHS (males, age 15-27; follow-up until 36 months)**						
V501-020: Gardasil vs. AAHS (m, 15-27; 36)	8	2032	11	2033	0.7%	0.73 [0.29, 1.81]
**Subtotal (95% CI)**		**2032**		**2033**	**0.7%**	**0.73 [0.29, 1.81]**
Total events	8		11			
Heterogeneity: Not applicable						
Test for overall effect: Z = 0.69 (P = 0.49)						
**3.9.15 Serious harms: Gardasil 9 vs. saline placebo (females, age 12-26; follow-up until 7 months)**						
V503-006: Gardasil 9 vs. saline (f, 12-26;7)	3	618	3	306	0.2%	0.50 [0.10, 2.44]
**Subtotal (95% CI)**		**618**		**306**	**0.2%**	**0.50 [0.10, 2.44]**
Total events	3		3			
Heterogeneity: Not applicable						
Test for overall effect: Z = 0.86 (P = 0.39)						
**3.9.16 Serious harms: HPV 16 vaccine vs. AAHS (females, age 16-25; follow-up until 48 months)**						
V501-005: HPV 16 vaccine vs. AAHS (f, 16-25; 48)	4	1204	3	1205	0.3%	1.33 [0.30, 5.95]
**Subtotal (95% CI)**		**1204**		**1205**	**0.3%**	**1.33 [0.30, 5.95]**
Total events	4		3			
Heterogeneity: Not applicable						
Test for overall effect: Z = 0.38 (P = 0.71)						
**Total (95% CI)**		**33477**		**32892**	**100.0%**	**1.01 [0.93, 1.09]**
Total events	1241		1234			
Heterogeneity: Tau² = 0.00; Chi² = 14.32, df = 22 (P = 0.89); I² = 0%						
Test for overall effect: Z = 0.16 (P = 0.87)						
Test for subgroup differences: Chi² = 9.48, df = 15 (P = 0.85), I² = 0%						

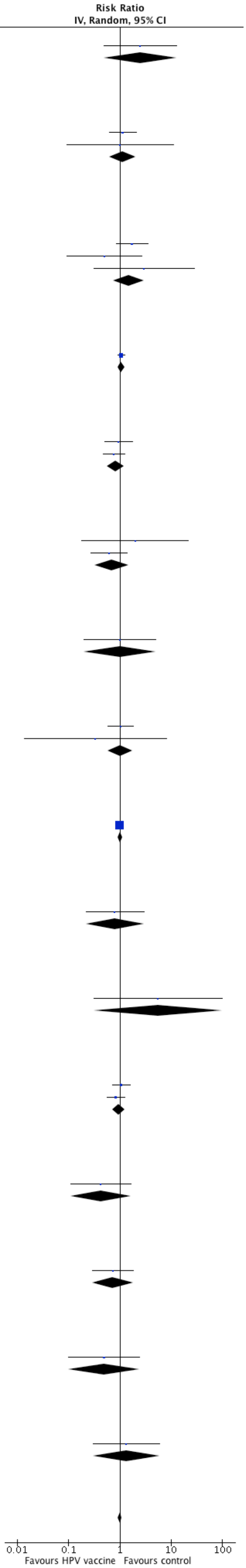

*3.9. Risk ratio for GlaxoSmithKline studies (i.e., HPV-0xx): 0.90 [0.70, 1.17]; risk ratio for Merck Sharp & Dohme studies (i.e., V50x-xxx): 1.02 [0.94, 1.10].

3.10. Serious harms judged as 'definitely associated'* with chronic regional pain syndrome (CRPS): intention to treat analysis

Study or Subgroup	HPV vaccine Events	HPV vaccine Total	Control Events	Control Total	Weight	Risk Ratio IV, Random, 95% CI
**3.10.1 Serious harms 'definitely associated' with CRPS: Cervarix vs. Al(OH)3 (females, age 18-35; follow-up until 7 months)**						
HPV-035: Cervarix vs. Al(OH)3 (f, 18-35; 7)	4	150	0	150	17.5%	9.00 [0.49, 165.71]
**Subtotal (95% CI)**		**150**		**150**	**17.5%**	**9.00 [0.49, 165.71]**
Total events	4		0			
Heterogeneity: Not applicable						
Test for overall effect: Z = 1.48 (P = 0.14)						
**3.10.2 Serious harms 'definitely associated' with CRPS: Cervarix vs. Al(OH)3 (females, age 24-72; follow-up until 36 months)**						
HPV-015: Cervarix vs. Al(OH)3 (f, 24-72; 36)	0	2882	0	2871		Not estimable
**Subtotal (95% CI)**		**2882**		**2871**		**Not estimable**
Total events	0		0			
Heterogeneity: Not applicable						
Test for overall effect: Not applicable						
**3.10.3 Serious harms 'definitely associated' with CRPS: Cervarix vs. Aimmugen: hepatitis A vaccine (females, age 22-29; follow-up until 12 months)**						
HPV-063: Cervarix vs. Aimmugen (f, 22-29; 12)	0	375	0	377		Not estimable
**Subtotal (95% CI)**		**375**		**377**		**Not estimable**
Total events	0		0			
Heterogeneity: Not applicable						
Test for overall effect: Not applicable						
**3.10.4 Serious harms 'definitely associated' with CRPS: Cervarix vs. Engerix: hepatitis B vaccine (females and males, age 9-16; follow-up until 72 months)**						
HPV-040: Cervarix vs. Engerix (f & m, 12-16; 72)	0	2436	0	1267		Not estimable
**Subtotal (95% CI)**		**2436**		**1267**		**Not estimable**
Total events	0		0			
Heterogeneity: Not applicable						
Test for overall effect: Not applicable						
**3.10.5 Serious harms 'definitely associated' with CRPS: Cervarix vs. Havrix: hepatitis A vaccine (females, age 10-14; follow-up until 12 months)**						
HPV-013: Cervarix vs. Havrix (f, 10-14; 12)	2	1035	2	1032	38.8%	1.00 [0.14, 7.07]
**Subtotal (95% CI)**		**1035**		**1032**	**38.8%**	**1.00 [0.14, 7.07]**
Total events	2		2			
Heterogeneity: Not applicable						
Test for overall effect: Z = 0.00 (P = 1.00)						
**3.10.6 Serious harms 'definitely associated' with CRPS: Cervarix vs. Havrix: hepatitis A vaccine (females, age 15-25; follow-up until 48 months)**						
HPV-008: Cervarix vs. Havrix (f, 15-25; 48)	0	9319	0	9325		Not estimable
**Subtotal (95% CI)**		**9319**		**9325**		**Not estimable**
Total events	0		0			
Heterogeneity: Not applicable						
Test for overall effect: Not applicable						
**3.10.7 Serious harms 'definitely associated' with CRPS: Gardasil vs. carrier solution (females and males, age 9-16; follow-up until 18 months)**						
V501-018: Gardasil vs. carrier solu. (f&m,9-16;18)	1	1184	0	597	14.5%	1.51 [0.06, 37.11]
**Subtotal (95% CI)**		**1184**		**597**	**14.5%**	**1.51 [0.06, 37.11]**
Total events	1		0			
Heterogeneity: Not applicable						
Test for overall effect: Z = 0.25 (P = 0.80)						
**3.10.8 Serious harms 'definitely associated' with CRPS: Gardasil vs. AAHS (females, age 15-26; follow-up until 45 months)**						
V501-013: Gardasil vs. AAHS (f, 16-24; 45)	0	2723	0	2732		Not estimable
V501-015: Gardasil vs. AAHS (f, 15-26; 36)	0	6087	0	6080		Not estimable
**Subtotal (95% CI)**		**8810**		**8812**		**Not estimable**
Total events	0		0			
Heterogeneity: Not applicable						
Test for overall effect: Not applicable						
**3.10.9 Serious harms 'definitely associated' with CRPS: Gardasil vs. AAHS (females, age 21-46; follow-up until 48 months)**						
V501-019: Gardasil vs. AAHS (f, 21-46; 48)	0	1911	0	1908		Not estimable
**Subtotal (95% CI)**		**1911**		**1908**		**Not estimable**
Total events	0		0			
Heterogeneity: Not applicable						
Test for overall effect: Not applicable						
**3.10.10 Serious harms 'definitely associated' with CRPS: Gardasil vs. AAHS (males, age 15-27; follow-up until 36 months)**						
V501-020: Gardasil vs. AAHS (m, 15-27; 36)	1	2032	0	2033	14.5%	3.00 [0.12, 73.64]
**Subtotal (95% CI)**		**2032**		**2033**	**14.5%**	**3.00 [0.12, 73.64]**
Total events	1		0			
Heterogeneity: Not applicable						
Test for overall effect: Z = 0.67 (P = 0.50)						
**3.10.11 Serious harms 'definitely associated' with CRPS: Gardasil 9 vs. saline placebo (females, age 12-26; follow-up until 7 months)**						
V503-006: Gardasil 9 vs. saline (f, 12-26;7)	1	618	0	306	14.6%	1.49 [0.06, 36.42]
**Subtotal (95% CI)**		**618**		**306**	**14.6%**	**1.49 [0.06, 36.42]**
Total events	1		0			
Heterogeneity: Not applicable						
Test for overall effect: Z = 0.24 (P = 0.81)						
**3.10.12 Serious harms 'definitely associated' with CRPS: HPV 16 vaccine vs. AAHS (females, age 16-25; follow-up until 48 months)**						
V501-005: HPV 16 vaccine vs. AAHS (f, 16-25; 48)	0	1204	0	1205		Not estimable
**Subtotal (95% CI)**		**1204**		**1205**		**Not estimable**
Total events	0		0			
Heterogeneity: Not applicable						
Test for overall effect: Not applicable						
**Total (95% CI)**		**31956**		**29883**	**100.0%**	**1.94 [0.57, 6.57]**
Total events	9		2			
Heterogeneity: $Tau^2 = 0.00$; $Chi^2 = 1.63$, df = 4 (P = 0.80); $I^2 = 0\%$						
Test for overall effect: Z = 1.06 (P = 0.29)						
Test for subgroup differences: $Chi^2 = 1.63$, df = 4 (P = 0.80), $I^2 = 0\%$						

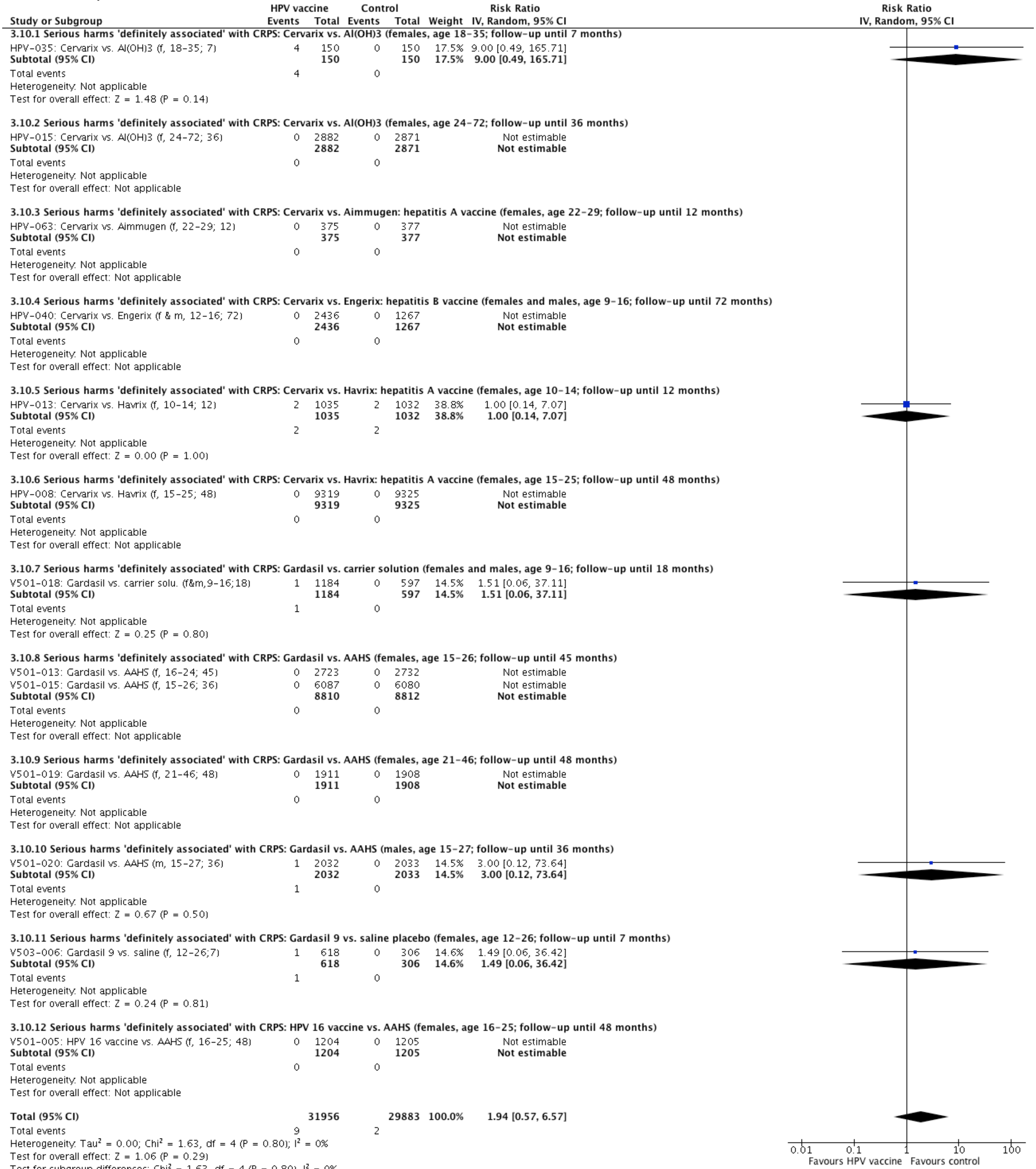

*3.10. Risk ratio for GlaxoSmithKline studies (i.e., HPV-0xx): 2.27 [0.28, 18.35]; risk ratio for Merck Sharp & Dohme studies (i.e., V50x-xxx): 1.89 [0.30, 11.99]. We asked a physician with clinical expertise in CRPS to assess the reported MedDRA preferred terms as 'definitely,' 'probably,' 'probably not' or 'definitely not' associated with CRPS. We sent an Excel sheet to the physician with all the reported MedDRA terms. The physician was blinded, as the Excel sheet contained no outcome data. When the physician had assessed all the MedDRA terms, we synthesized the data for those MedDRA terms that the physician judged 'definitely' associated with CRPS and compared it to the reported serious harms.

3.11. Serious harms judged as 'definitely associated'* with postural orthostatic tachycardia syndrome (POTS): intention to treat analysis

Study or Subgroup	HPV vaccine Events	HPV vaccine Total	Control Events	Control Total	Weight	Risk Ratio IV, Random, 95% CI
**3.11.1 Serious harms 'definitely associated' with POTS: Cervarix vs. Al(OH)3 (females, age 18-35; follow-up until 7 months)**						
HPV-035: Cervarix vs. Al(OH)3 (f, 18-35; 7)	3	150	0	150	22.2%	7.00 [0.36, 134.36]
**Subtotal (95% CI)**		**150**		**150**	**22.2%**	**7.00 [0.36, 134.36]**
Total events	3		0			
Heterogeneity: Not applicable						
Test for overall effect: Z = 1.29 (P = 0.20)						
**3.11.2 Serious harms 'definitely associated' with POTS: Cervarix vs. Al(OH)3 (females, age 24-72; follow-up until 36 months)**						
HPV-015: Cervarix vs. Al(OH)3 (f, 24-72; 36)	0	2882	0	2871		Not estimable
**Subtotal (95% CI)**		**2882**		**2871**		**Not estimable**
Total events	0		0			
Heterogeneity: Not applicable						
Test for overall effect: Not applicable						
**3.11.3 Serious harms 'definitely associated' with POTS: Cervarix vs. Aimmugen: hepatitis A vaccine (females, age 22-29; follow-up until 12 months)**						
HPV-063: Cervarix vs. Aimmugen (f, 22-29; 12)	0	375	0	377		Not estimable
**Subtotal (95% CI)**		**375**		**377**		**Not estimable**
Total events	0		0			
Heterogeneity: Not applicable						
Test for overall effect: Not applicable						
**3.11.4 Serious harms 'definitely associated' with POTS: Cervarix vs. Engerix: hepatitis B vaccine (females and males, age 9-16; follow-up until 72 months)**						
HPV-040: Cervarix vs. Engerix (f & m, 12-16; 72)	0	2436	0	1267		Not estimable
**Subtotal (95% CI)**		**2436**		**1267**		**Not estimable**
Total events	0		0			
Heterogeneity: Not applicable						
Test for overall effect: Not applicable						
**3.11.5 Serious harms 'definitely associated' with POTS: Cervarix vs. Havrix: hepatitis A vaccine (females, age 10-14; follow-up until 12 months)**						
HPV-013: Cervarix vs. Havrix (f, 10-14; 12)	0	1035	2	1032	21.0%	0.20 [0.01, 4.15]
**Subtotal (95% CI)**		**1035**		**1032**	**21.0%**	**0.20 [0.01, 4.15]**
Total events	0		2			
Heterogeneity: Not applicable						
Test for overall effect: Z = 1.04 (P = 0.30)						
**3.11.6 Serious harms 'definitely associated' with POTS: Cervarix vs. Havrix: hepatitis A vaccine (females, age 15-25; follow-up until 48 months)**						
HPV-008: Cervarix vs. Havrix (f, 15-25; 48)	0	9319	0	9325		Not estimable
**Subtotal (95% CI)**		**9319**		**9325**		**Not estimable**
Total events	0		0			
Heterogeneity: Not applicable						
Test for overall effect: Not applicable						
**3.11.7 Serious harms 'definitely associated' with POTS: Cervarix vs. Twinrix: hepatitis A and B vaccine (females, age 8-15; follow-up until 12 months)**						
HPV-029: Cervarix vs. Twinrix (f, 8-15; 12)	1	270	0	271	19.0%	3.01 [0.12, 73.59]
**Subtotal (95% CI)**		**270**		**271**	**19.0%**	**3.01 [0.12, 73.59]**
Total events	1		0			
Heterogeneity: Not applicable						
Test for overall effect: Z = 0.68 (P = 0.50)						
**3.11.8 Serious harms 'definitely associated' with POTS: Gardasil vs. AAHS (females, age 15-26; follow-up until 45 months)**						
V501-013: Gardasil vs. AAHS (f, 16-24; 45)	0	2723	0	2732		Not estimable
V501-015: Gardasil vs. AAHS (f, 15-26; 36)	0	6087	0	6080		Not estimable
**Subtotal (95% CI)**		**8810**		**8812**		**Not estimable**
Total events	0		0			
Heterogeneity: Not applicable						
Test for overall effect: Not applicable						
**3.11.9 Serious harms 'definitely associated' with POTS: Gardasil vs. AAHS (females, age 21-46; follow-up until 48 months)**						
V501-019: Gardasil vs. AAHS (f, 21-46; 48)	0	1911	0	1908		Not estimable
**Subtotal (95% CI)**		**1911**		**1908**		**Not estimable**
Total events	0		0			
Heterogeneity: Not applicable						
Test for overall effect: Not applicable						
**3.11.10 Serious harms 'definitely associated' with POTS: Gardasil vs. AAHS (males, age 15-27; follow-up until 36 months)**						
V501-020: Gardasil vs. AAHS (m, 15-27; 36)	1	2032	0	2033	18.9%	3.00 [0.12, 73.64]
**Subtotal (95% CI)**		**2032**		**2033**	**18.9%**	**3.00 [0.12, 73.64]**
Total events	1		0			
Heterogeneity: Not applicable						
Test for overall effect: Z = 0.67 (P = 0.50)						
**3.11.11 Serious harms 'definitely associated' with POTS: Gardasil 9 vs. saline placebo (females, age 12-26; follow-up until 7 months)**						
V503-006: Gardasil 9 vs. saline (f, 12-26;7)	1	618	0	306	18.9%	1.49 [0.06, 36.42]
**Subtotal (95% CI)**		**618**		**306**	**18.9%**	**1.49 [0.06, 36.42]**
Total events	1		0			
Heterogeneity: Not applicable						
Test for overall effect: Z = 0.24 (P = 0.81)						
**3.11.12 Serious harms 'definitely associated' with POTS: HPV 16 vaccine vs. AAHS (females, age 16-25; follow-up until 48 months)**						
V501-005: HPV 16 vaccine vs. AAHS (f, 16-25; 48)	0	1204	0	1205		Not estimable
**Subtotal (95% CI)**		**1204**		**1205**		**Not estimable**
Total events	0		0			
Heterogeneity: Not applicable						
Test for overall effect: Not applicable						
**Total (95% CI)**		**31042**		**29557**	**100.0%**	**1.79 [0.45, 7.22]**
Total events	6		2			
Heterogeneity: $Tau^2 = 0.00$; $Chi^2 = 3.04$, df = 4 (P = 0.55); $I^2 = 0\%$						
Test for overall effect: Z = 0.82 (P = 0.41)						
Test for subgroup differences: $Chi^2 = 3.04$, df = 4 (P = 0.55), $I^2 = 0\%$						

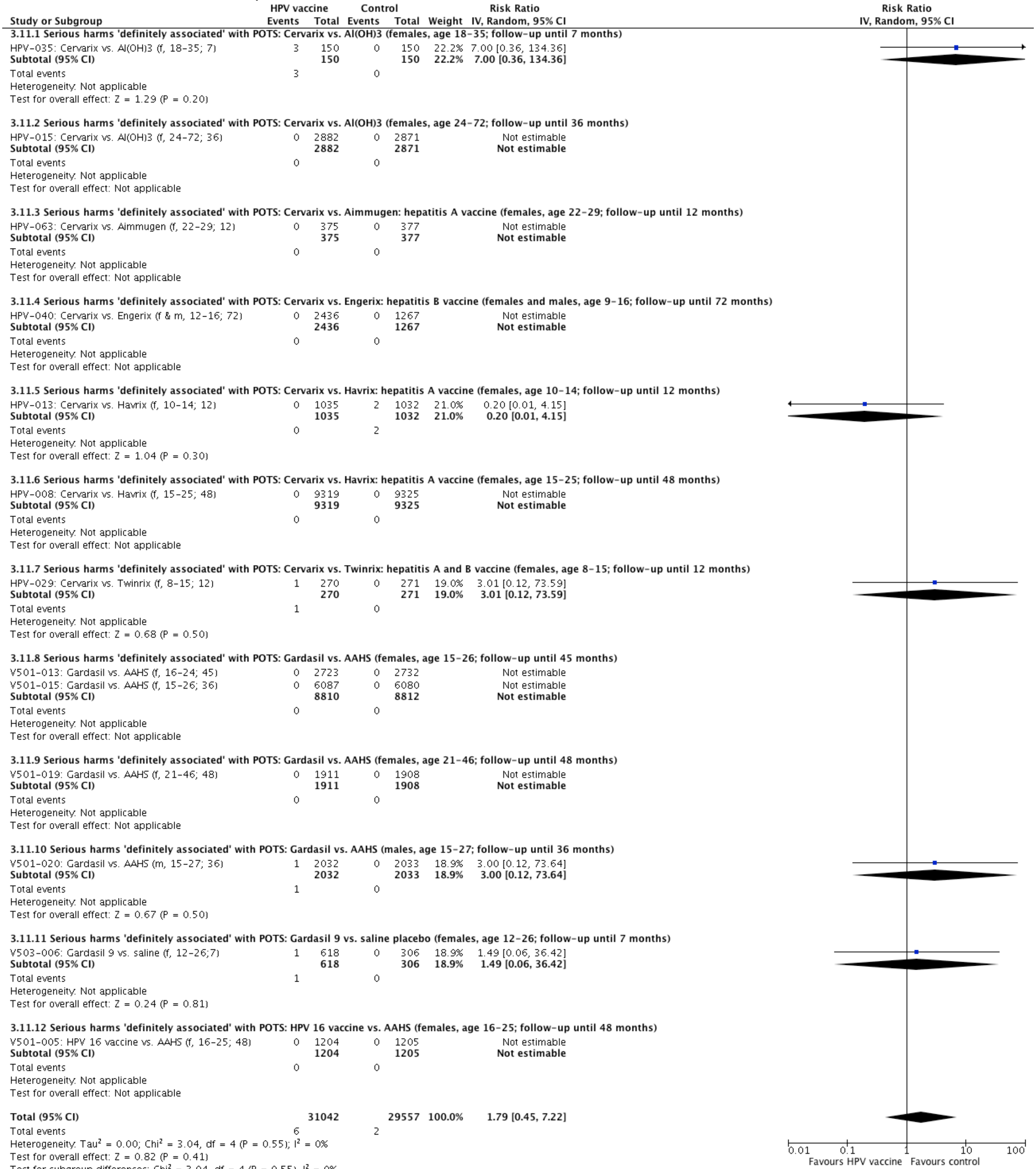

*3.11. Risk ratio for GlaxoSmithKline studies (i.e., HPV-0xx): 1.62 [0.19, 13.68]; risk ratio for Merck Sharp & Dohme studies (i.e., V50x-xxx): 2.11 [0.22, 20.29]. We asked a physician with clinical expertise in POTS to assess the reported MedDRA preferred terms as 'definitely,' 'probably,' 'probably not' or 'definitely not' associated with POTS. We sent an Excel sheet to the physician with all the reported MedDRA terms. The physician was blinded, as the Excel sheet contained no outcome data. When the physician had assessed all the MedDRA terms, we synthesized the data for those MedDRA terms that the physician judged 'definitely' associated with POTS and compared it to the reported serious harms.

3.12. Serious harms reported within the MedDRA system organ class 'nervous system disorders (10029205)'*: intention to treat analysis

Study or Subgroup	HPV vaccine Events	HPV vaccine Total	Control Events	Control Total	Weight	Risk Ratio IV, Random, 95% CI
**3.12.1 Serious nervous system disorders: Cervarix vs. Al(OH)3 (females, age 18-35; follow-up until 12 months)**						
HPV-003: Cervarix vs. Al(OH)3 (f, 18-30; 12)	0	31	0	30		Not estimable
HPV-035: Cervarix vs. Al(OH)3 (f, 18-35; 7)	2	150	0	150	11.0%	5.00 [0.24, 103.28]
**Subtotal (95% CI)**		**181**		**180**	**11.0%**	**5.00 [0.24, 103.28]**
Total events	2		0			
Heterogeneity: Not applicable						
Test for overall effect: Z = 1.04 (P = 0.30)						
**3.12.2 Serious nervous system disorders: Cervarix vs. Al(OH)3 (females, age 24-72; follow-up until 36 months)**						
HPV-015: Cervarix vs. Al(OH)3 (f, 24-72; 36)	0	2882	0	2871		Not estimable
**Subtotal (95% CI)**		**2882**		**2871**		**Not estimable**
Total events	0		0			
Heterogeneity: Not applicable						
Test for overall effect: Not applicable						
**3.12.3 Serious nervous system disorders: Cervarix vs. Aimmugen: hepatitis A vaccine (females, age 22-29; follow-up until 12 months)**						
HPV-063: Cervarix vs. Aimmugen (f, 22-29; 12)	0	375	0	377		Not estimable
**Subtotal (95% CI)**		**375**		**377**		**Not estimable**
Total events	0		0			
Heterogeneity: Not applicable						
Test for overall effect: Not applicable						
**3.12.4 Serious nervous system disorders: Cervarix vs. Engerix: hepatitis B vaccine (females and males, age 12-16; follow-up until 72 months)**						
HPV-040: Cervarix vs. Engerix (f & m, 12-16; 72)	0	2436	0	1267		Not estimable
**Subtotal (95% CI)**		**2436**		**1267**		**Not estimable**
Total events	0		0			
Heterogeneity: Not applicable						
Test for overall effect: Not applicable						
**3.12.5 Serious nervous system disorders: Cervarix vs. Engerix: hepatitis B vaccine (females, age 26-46; follow-up until 12 months)**						
HPV-069: Cervarix vs. Engerix (f, 26-46; 12)	0	606	0	606		Not estimable
**Subtotal (95% CI)**		**606**		**606**		**Not estimable**
Total events	0		0			
Heterogeneity: Not applicable						
Test for overall effect: Not applicable						
**3.12.6 Serious nervous system disorders: Cervarix vs. Havrix: hepatitis A vaccine (females, age 10-14; follow-up until 12 months)**						
HPV-013: Cervarix vs. Havrix (f, 10-14; 12)	1	1035	1	1032	13.1%	1.00 [0.06, 15.92]
**Subtotal (95% CI)**		**1035**		**1032**	**13.1%**	**1.00 [0.06, 15.92]**
Total events	1		1			
Heterogeneity: Not applicable						
Test for overall effect: Z = 0.00 (P = 1.00)						
**3.12.7 Serious nervous system disorders: Cervarix vs. Havrix: hepatitis A vaccine (females, age 15-25; follow-up until 48 months)**						
HPV-008: Cervarix vs. Havrix (f, 15-25; 48)	0	9319	0	9325		Not estimable
**Subtotal (95% CI)**		**9319**		**9325**		**Not estimable**
Total events	0		0			
Heterogeneity: Not applicable						
Test for overall effect: Not applicable						
**3.12.8 Serious nervous system disorders: Gardasil vs. AAHS (females, age 15-26; follow-up until 45 months)**						
V501-013: Gardasil vs. AAHS (f, 16-24; 45)	1	2723	4	2732	21.0%	0.25 [0.03, 2.24]
V501-015: Gardasil vs. AAHS (f, 15-26; 36)	4	6087	1	6080	21.0%	4.00 [0.45, 35.74]
**Subtotal (95% CI)**		**8810**		**8812**	**42.0%**	**1.00 [0.07, 15.09]**
Total events	5		5			
Heterogeneity: Tau² = 2.58; Chi² = 3.07, df = 1 (P = 0.08); I² = 67%						
Test for overall effect: Z = 0.00 (P = 1.00)						
**3.12.9 Serious nervous system disorders: Gardasil vs. AAHS (females, age 21-46; follow-up until 48 months)**						
V501-019: Gardasil vs. AAHS (f, 21-46; 48)	2	1911	0	1908	10.9%	4.99 [0.24, 103.91]
**Subtotal (95% CI)**		**1911**		**1908**	**10.9%**	**4.99 [0.24, 103.91]**
Total events	2		0			
Heterogeneity: Not applicable						
Test for overall effect: Z = 1.04 (P = 0.30)						
**3.12.10 Serious nervous system disorders: Gardasil vs. AAHS (males, age 15-27; follow-up until 36 months)**						
V501-020: Gardasil vs. AAHS (m, 15-27; 36)	1	2032	0	2033	9.8%	3.00 [0.12, 73.64]
**Subtotal (95% CI)**		**2032**		**2033**	**9.8%**	**3.00 [0.12, 73.64]**
Total events	1		0			
Heterogeneity: Not applicable						
Test for overall effect: Z = 0.67 (P = 0.50)						
**3.12.11 Serious nervous system disorders: Gardasil 9 vs. saline placebo (females, age 12-26; follow-up until 7 months)**						
V503-006: Gardasil 9 vs. saline (f, 12-26;7)	1	618	1	306	13.1%	0.50 [0.03, 7.89]
**Subtotal (95% CI)**		**618**		**306**	**13.1%**	**0.50 [0.03, 7.89]**
Total events	1		1			
Heterogeneity: Not applicable						
Test for overall effect: Z = 0.50 (P = 0.62)						
**3.12.12 Serious nervous system disorders: HPV 16 vaccine vs. AAHS (females, age 16-25; follow-up until 48 months)**						
V501-005: HPV 16 vaccine vs. AAHS (f, 16-25; 48)	0	1204	0	1205		Not estimable
**Subtotal (95% CI)**		**1204**		**1205**		**Not estimable**
Total events	0		0			
Heterogeneity: Not applicable						
Test for overall effect: Not applicable						
**Total (95% CI)**		**31409**		**29922**	**100.0%**	**1.45 [0.53, 3.94]**
Total events	12		7			
Heterogeneity: Tau² = 0.00; Chi² = 5.41, df = 6 (P = 0.49); I² = 0%						
Test for overall effect: Z = 0.72 (P = 0.47)						
Test for subgroup differences: Chi² = 2.14, df = 5 (P = 0.83), I² = 0%						

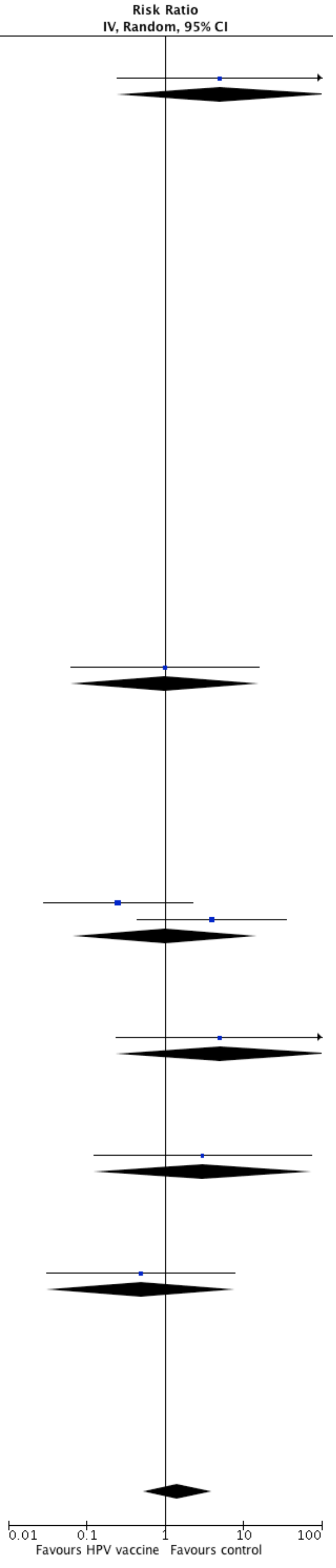

*3.12. Risk ratio for GlaxoSmithKline studies (i.e., HPV-0xx): 2.08 [0.27, 16.05]; risk ratio for Merck Sharp & Dohme studies (i.e., V50x-xxx): 1.31 [0.37, 4.60].

3.13. New onset diseases ('medically significant conditions' and 'new medical history'*): intention to treat analysis

Study or Subgroup	HPV vaccine Events	HPV vaccine Total	Control Events	Control Total	Weight	Risk Ratio IV, Random, 95% CI
**3.13.1 New onset diseases ('medically significant conditions'): Cervarix vs. Al(OH)3 (females, age 9-17; follow-up until 12 months)**						
HPV-058: Cervarix vs. Al(OH)3 (f, 9-17; 12)	14	374	11	376	1.2%	1.28 [0.59, 2.78]
**Subtotal (95% CI)**		**374**		**376**	**1.2%**	**1.28 [0.59, 2.78]**
Total events	14		11			
Heterogeneity: Not applicable						
Test for overall effect: Z = 0.62 (P = 0.53)						
**3.13.2 New onset diseases ('medically significant conditions'): Cervarix vs. Al(OH)3 (females, age 15-25; follow-up until 7 months)**						
HPV-038: Cervarix vs. Al(OH)3 (f, 15-25; 7)	34	149	10	76	1.6%	1.73 [0.91, 3.32]
**Subtotal (95% CI)**		**149**		**76**	**1.6%**	**1.73 [0.91, 3.32]**
Total events	34		10			
Heterogeneity: Not applicable						
Test for overall effect: Z = 1.66 (P = 0.10)						
**3.13.3 New onset diseases ('medically significant conditions'): Cervarix vs. Al(OH)3 (females, age 18-35; follow-up until 7 months)**						
HPV-023: Cervarix vs. Al(OH)3 (f, 21-32; 36)	60	222	38	211	4.7%	1.50 [1.05, 2.15]
HPV-031: Cervarix vs. Al(OH)3 (f, 18-35; 7)	13	176	24	178	1.7%	0.55 [0.29, 1.04]
HPV-035: Cervarix vs. Al(OH)3 (f, 18-35; 7)	42	150	24	150	3.2%	1.75 [1.12, 2.74]
**Subtotal (95% CI)**		**548**		**539**	**9.6%**	**1.19 [0.66, 2.13]**
Total events	115		86			
Heterogeneity: Tau² = 0.20; Chi² = 9.20, df = 2 (P = 0.01); I² = 78%						
Test for overall effect: Z = 0.58 (P = 0.56)						
**3.13.4 New onset diseases ('medically significant conditions'): Cervarix vs. Al(OH)3 (females, age 24-72; follow-up until 36 months)**						
HPV-015: Cervarix vs. Al(OH)3 (f, 24-72; 36)	1169	2882	1136	2871	23.8%	1.03 [0.96, 1.09]
**Subtotal (95% CI)**		**2882**		**2871**	**23.8%**	**1.03 [0.96, 1.09]**
Total events	1169		1136			
Heterogeneity: Not applicable						
Test for overall effect: Z = 0.77 (P = 0.44)						
**3.13.5 New onset diseases ('medically significant conditions'): Cervarix vs. Aimmugen: hepatitis A vaccine (females, age 22-29; follow-up until 12 months)**						
HPV-032: Cervarix vs. Aimmugen (f, 19-25; 7)	91	519	107	521	8.1%	0.85 [0.66, 1.10]
HPV-063: Cervarix vs. Aimmugen (f, 22-29; 12)	98	375	115	377	9.3%	0.86 [0.68, 1.08]
**Subtotal (95% CI)**		**894**		**898**	**17.4%**	**0.86 [0.72, 1.01]**
Total events	189		222			
Heterogeneity: Tau² = 0.00; Chi² = 0.00, df = 1 (P = 0.98); I² = 0%						
Test for overall effect: Z = 1.81 (P = 0.07)						
**3.13.6 New onset diseases ('medically significant conditions'): Cervarix vs. Engerix: hepatitis B vaccine (females and males, age 9-15; follow-up until 12 months)**						
HPV-030: Cervarix vs. Engerix (f, 9-15; 12)	30	247	23	246	2.5%	1.30 [0.78, 2.17]
HPV-040: Cervarix vs. Engerix (f & m, 12-16; 72)	47	643	76	1047	4.9%	1.01 [0.71, 1.43]
**Subtotal (95% CI)**		**890**		**1293**	**7.4%**	**1.09 [0.82, 1.46]**
Total events	77		99			
Heterogeneity: Tau² = 0.00; Chi² = 0.64, df = 1 (P = 0.42); I² = 0%						
Test for overall effect: Z = 0.59 (P = 0.55)						
**3.13.7 New onset diseases ('medically significant conditions'): Cervarix vs. Engerix: hepatitis B vaccine (females, age 26-46; follow-up until 12 months)**						
HPV-069: Cervarix vs. Engerix (f, 26-46; 12)	5	606	7	606	0.6%	0.71 [0.23, 2.24]
**Subtotal (95% CI)**		**606**		**606**	**0.6%**	**0.71 [0.23, 2.24]**
Total events	5		7			
Heterogeneity: Not applicable						
Test for overall effect: Z = 0.58 (P = 0.56)						
**3.13.8 New onset diseases ('medically significant conditions'): Cervarix vs. Havrix: hepatitis A vaccine (females, age 9-15; follow-up until 12 months)**						
HPV-013: Cervarix vs. Havrix (f, 10-14; 12)	166	1035	195	1032	11.8%	0.85 [0.70, 1.02]
HPV-033: Cervarix vs. Havrix (f, 9-15; 7)	11	160	10	161	1.0%	1.11 [0.48, 2.53]
**Subtotal (95% CI)**		**1195**		**1193**	**12.8%**	**0.86 [0.72, 1.03]**
Total events	177		205			
Heterogeneity: Tau² = 0.00; Chi² = 0.38, df = 1 (P = 0.54); I² = 0%						
Test for overall effect: Z = 1.61 (P = 0.11)						
**3.13.9 New onset diseases ('medically significant conditions'): Cervarix vs. Havrix: hepatitis A vaccine (females, age 15-25; follow-up until 48 months)**						
HPV-008: Cervarix vs. Havrix (f, 15-25; 48)	2960	9319	3025	9325	25.6%	0.98 [0.94, 1.02]
**Subtotal (95% CI)**		**9319**		**9325**	**25.6%**	**0.98 [0.94, 1.02]**
Total events	2960		3025			
Heterogeneity: Not applicable						
Test for overall effect: Z = 0.99 (P = 0.32)						
**3.13.10 New onset diseases ('medically significant conditions'): Cervarix vs. Twinrix: hepatitis A and B vaccine (females, age 8-15; follow-up until 12 months)**						
HPV-029: Cervarix vs. Twinrix (f, 8-15; 12)	0	270	0	271		Not estimable
**Subtotal (95% CI)**		**270**		**271**		**Not estimable**
Total events	0		0			
Heterogeneity: Not applicable						
Test for overall effect: Not applicable						
**3.13.11 New onset diseases ('new medical history'): Gardasil vs. carrier solution (females and males, age 9-16; follow-up until 18 months)**						
V501-018: Gardasil vs. carrier solu. (f&m,9-16;18)	0	1184	0	597		Not estimable
**Subtotal (95% CI)**		**1184**		**597**		**Not estimable**
Total events	0		0			
Heterogeneity: Not applicable						
Test for overall effect: Not applicable						
**3.13.12 New onset diseases ('new medical history'): Gardasil vs. AAHS (females, age 15-26; follow-up until 45 months)**						
V501-013: Gardasil vs. AAHS (f, 16-24; 45)	0	2723	0	2732		Not estimable
V501-015: Gardasil vs. AAHS (f, 15-26; 36)	0	6087	0	6080		Not estimable
**Subtotal (95% CI)**		**8810**		**8812**		**Not estimable**
Total events	0		0			
Heterogeneity: Not applicable						
Test for overall effect: Not applicable						
**3.13.13 New onset diseases ('new medical history'): Gardasil vs. AAHS (females, age 21-46; follow-up until 48 months)**						
V501-019: Gardasil vs. AAHS (f, 21-46; 48)	0	1911	0	1908		Not estimable
**Subtotal (95% CI)**		**1911**		**1908**		**Not estimable**
Total events	0		0			
Heterogeneity: Not applicable						
Test for overall effect: Not applicable						
**3.13.14 New onset diseases ('new medical history'): Gardasil vs. AAHS (males, age 15-27; follow-up until 36 months)**						
V501-020: Gardasil vs. AAHS (m, 15-27; 36)	0	2032	0	2032		Not estimable
**Subtotal (95% CI)**		**2032**		**2032**		**Not estimable**
Total events	0		0			
Heterogeneity: Not applicable						
Test for overall effect: Not applicable						
**3.13.15 New onset diseases ('new medical history'): Gardasil 9 vs. saline placebo (females, age 12-26; follow-up until 7 months)**						
V503-006: Gardasil 9 vs. saline (f, 12-26;7)	0	618	0	306		Not estimable
**Subtotal (95% CI)**		**618**		**306**		**Not estimable**
Total events	0		0			
Heterogeneity: Not applicable						
Test for overall effect: Not applicable						
**3.13.16 New onset diseases ('new medical history'): HPV 16 vaccine vs. AAHS (females, age 16-25; follow-up until 48 months)**						
V501-005: HPV 16 vaccine vs. AAHS (f, 16-25; 48)	0	1204	0	1205		Not estimable
**Subtotal (95% CI)**		**1204**		**1205**		**Not estimable**
Total events	0		0			
Heterogeneity: Not applicable						
Test for overall effect: Not applicable						
**Total (95% CI)**		**32886**		**32308**	**100.0%**	**1.00 [0.92, 1.09]**
Total events	4740		4801			
Heterogeneity: Tau² = 0.01; Chi² = 26.25, df = 13 (P = 0.02); I² = 50%						
Test for overall effect: Z = 0.02 (P = 0.98)						
Test for subgroup differences: Chi² = 10.93, df = 8 (P = 0.21), I² = 26.8%						

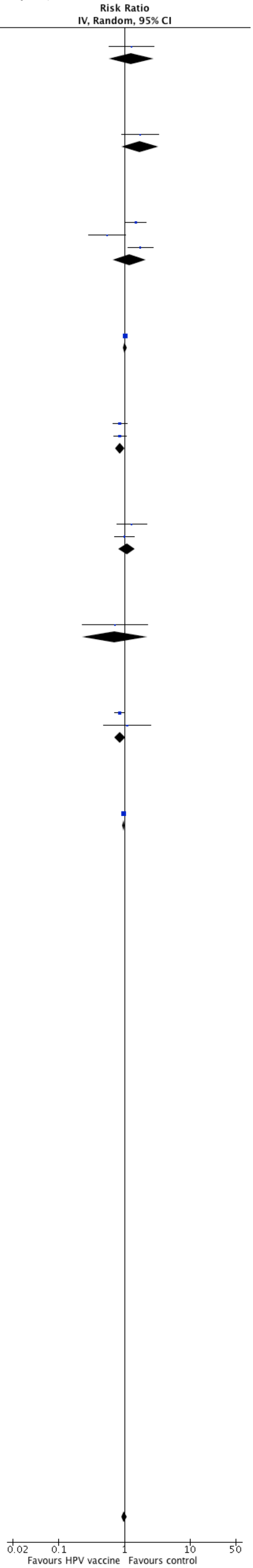

*3.13. Risk ratio for 'medically significant conditions' (GlaxoSmithKline): 1.00 [0.92, 1.09]; risk ratio for 'new medical history' (Merck Sharp & Dohme): not applicable.

3.14. New onset diseases most associated with the HPV vaccines (‘medically significant conditions’) - ‘back pain’: intention to treat analysis

Study or Subgroup	HPV vaccine Events	HPV vaccine Total	Control Events	Control Total	Weight	Risk Ratio IV, Random, 95% CI
**3.14.1 New onset back pain ('medically significant conditions'): Cervarix vs. Al(OH)3 (females, age 18-35; follow-up until 12 months)**						
HPV-031: Cervarix vs. Al(OH)3 (f, 18-35; 7)	0	176	0	178		Not estimable
HPV-035: Cervarix vs. Al(OH)3 (f, 18-35; 7)	0	150	0	150		Not estimable
**Subtotal (95% CI)**		**326**		**328**		**Not estimable**
Total events	0		0			
Heterogeneity: Not applicable						
Test for overall effect: Not applicable						
**3.14.2 New onset back pain ('medically significant conditions'): Cervarix vs. Al(OH)3 (females, age 24-72; follow-up until 36 months)**						
HPV-015: Cervarix vs. Al(OH)3 (f, 24-72; 36)	0	2882	0	2871		Not estimable
**Subtotal (95% CI)**		**2882**		**2871**		**Not estimable**
Total events	0		0			
Heterogeneity: Not applicable						
Test for overall effect: Not applicable						
**3.14.3 New onset back pain ('medically significant conditions'): Cervarix vs. Aimmugen: hepatitis A vaccine (females, age 22-29; follow-up until 12 months)**						
HPV-063: Cervarix vs. Aimmugen (f, 22-29; 12)	0	375	0	377		Not estimable
**Subtotal (95% CI)**		**375**		**377**		**Not estimable**
Total events	0		0			
Heterogeneity: Not applicable						
Test for overall effect: Not applicable						
**3.14.4 New onset back pain ('medically significant conditions'): Cervarix vs. Havrix: hepatitis A vaccine (females, age 10-14; follow-up until 12 months)**						
HPV-013: Cervarix vs. Havrix (f, 10-14; 12)	0	1035	0	1032		Not estimable
**Subtotal (95% CI)**		**1035**		**1032**		**Not estimable**
Total events	0		0			
Heterogeneity: Not applicable						
Test for overall effect: Not applicable						
**3.14.5 New onset back pain ('medically significant conditions'): Cervarix vs. Havrix: hepatitis A vaccine (females, age 15-25; follow-up until 48 months)**						
HPV-008: Cervarix vs. Havrix (f, 15-25; 48)	0	9319	0	9325		Not estimable
**Subtotal (95% CI)**		**9319**		**9325**		**Not estimable**
Total events	0		0			
Heterogeneity: Not applicable						
Test for overall effect: Not applicable						
**3.14.6 New onset back pain ('new medical history'): Gardasil vs. carrier solution (females and males, age 9-16; follow-up until 18 months)**						
V501-018: Gardasil vs. carrier solu. (f&m,9-16;18)	0	1184	0	597		Not estimable
**Subtotal (95% CI)**		**1184**		**597**		**Not estimable**
Total events	0		0			
Heterogeneity: Not applicable						
Test for overall effect: Not applicable						
**3.14.7 New onset back pain ('new medical history'): Gardasil vs. AAHS (females, age 15-26; follow-up until 45 months)**						
V501-013: Gardasil vs. AAHS (f, 16-24; 45)	0	2723	0	2732		Not estimable
V501-015: Gardasil vs. AAHS (f, 15-26; 36)	0	6087	0	6080		Not estimable
**Subtotal (95% CI)**		**8810**		**8812**		**Not estimable**
Total events	0		0			
Heterogeneity: Not applicable						
Test for overall effect: Not applicable						
**3.14.8 New onset back pain ('new medical history'): Gardasil vs. AAHS (females, age 21-46; follow-up until 48 months)**						
V501-019: Gardasil vs. AAHS (f, 21-46; 48)	0	1911	0	1908		Not estimable
**Subtotal (95% CI)**		**1911**		**1908**		**Not estimable**
Total events	0		0			
Heterogeneity: Not applicable						
Test for overall effect: Not applicable						
**3.14.9 New onset back pain ('new medical history'): Gardasil vs. AAHS (males, age 15-27; follow-up until 36 months)**						
V501-020: Gardasil vs. AAHS (m, 15-27; 36)	0	2032	0	2033		Not estimable
**Subtotal (95% CI)**		**2032**		**2033**		**Not estimable**
Total events	0		0			
Heterogeneity: Not applicable						
Test for overall effect: Not applicable						
**3.14.10 New onset back pain ('new medical history'): Gardasil 9 vs. saline placebo (females, age 12-26; follow-up until 7 months)**						
V503-006: Gardasil 9 vs. saline (f, 12-26;7)	0	618	0	306		Not estimable
**Subtotal (95% CI)**		**618**		**306**		**Not estimable**
Total events	0		0			
Heterogeneity: Not applicable						
Test for overall effect: Not applicable						
**3.14.11 New onset back pain ('new medical history'): HPV 16 vaccine vs. AAHS (females, age 16-25; follow-up until 48 months)**						
V501-005: HPV 16 vaccine vs. AAHS (f, 16-25; 48)	0	1204	0	1205		Not estimable
**Subtotal (95% CI)**		**1204**		**1205**		**Not estimable**
Total events	0		0			
Heterogeneity: Not applicable						
Test for overall effect: Not applicable						
**Total (95% CI)**		**29696**		**28794**		**Not estimable**
Total events	0		0			
Heterogeneity: Not applicable						
Test for overall effect: Not applicable						
Test for subgroup differences: Not applicable						

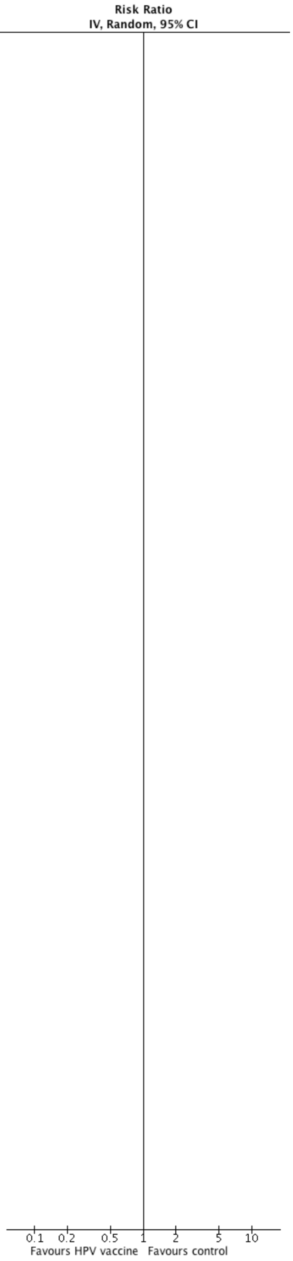

*3.14. Risk ratio for 'medically significant conditions’ (GlaxoSmithKline): not applicable; risk ratio for ‘new medical history’ (Merck Sharp & Dohme): not applicable.

3.15. New onset diseases most inversely associated with the HPV vaccines (‘new medical history’) - ‘vaginal infection’: intention to treat analysis

Study or Subgroup	HPV vaccine Events	HPV vaccine Total	Control Events	Control Total	Weight	Risk Ratio IV, Random, 95% CI
**3.15.1 New onset vaginal infection ('new medical history'): Gardasil vs. carrier solution (females and males, age 9-16; follow-up until 18 months)**						
V501-018: Gardasil vs. carrier solu. (f&m,9-16;18)	0	1184	0	597		Not estimable
**Subtotal (95% CI)**		**1184**		**597**		**Not estimable**
Total events	0		0			
Heterogeneity: Not applicable						
Test for overall effect: Not applicable						
**3.15.2 New onset vaginal infection ('new medical history'): Gardasil vs. AAHS (females, age 15-26; follow-up until 45 months)**						
V501-013: Gardasil vs. AAHS (f, 16-24; 45)	0	2723	0	2732		Not estimable
V501-015: Gardasil vs. AAHS (f, 15-26; 36)	0	6087	0	6080		Not estimable
**Subtotal (95% CI)**		**8810**		**8812**		**Not estimable**
Total events	0		0			
Heterogeneity: Not applicable						
Test for overall effect: Not applicable						
**3.15.3 New onset vaginal infection ('new medical history'): Gardasil vs. AAHS (females, age 21-46; follow-up until 48 months)**						
V501-019: Gardasil vs. AAHS (f, 21-46; 48)	0	1911	0	1908		Not estimable
**Subtotal (95% CI)**		**1911**		**1908**		**Not estimable**
Total events	0		0			
Heterogeneity: Not applicable						
Test for overall effect: Not applicable						
**3.15.4 New onset vaginal infection ('new medical history'): Gardasil 9 vs. saline placebo (females, age 12-26; follow-up until 7 months)**						
V503-006: Gardasil 9 vs. saline (f, 12-26;7)	0	618	0	306		Not estimable
**Subtotal (95% CI)**		**618**		**306**		**Not estimable**
Total events	0		0			
Heterogeneity: Not applicable						
Test for overall effect: Not applicable						
**Total (95% CI)**		**12523**		**11623**		**Not estimable**
Total events	0		0			
Heterogeneity: Not applicable						
Test for overall effect: Not applicable						
Test for subgroup differences: Not applicable						

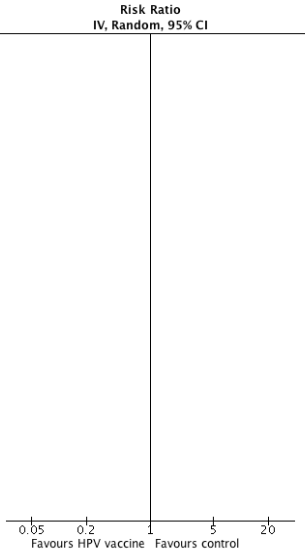

*3.15. Risk ratio for 'medically significant conditions’ (GlaxoSmithKline): not applicable; risk ratio for ‘new medical history’ (Merck Sharp & Dohme): not applicable.

3.16. New onset diseases ('medically significant conditions' and 'new medical history') reported within the MedDRA system organ class ‘vascular disorders (10047065)': intention to treat analysis

Study or Subgroup	HPV vaccine Events	HPV vaccine Total	Control Events	Control Total	Weight	Risk Ratio IV, Random, 95% CI
**3.16.1 New onset vascular disorders ('medically significant conditions'): Cervarix vs. Al(OH)3 (females, age 24-72; follow-up until 36 months)**						
HPV-015: Cervarix vs. Al(OH)3 (f, 24-72; 36)	0	2882	0	2871		Not estimable
**Subtotal (95% CI)**		**2882**		**2871**		**Not estimable**
Total events	0		0			
Heterogeneity: Not applicable						
Test for overall effect: Not applicable						
**3.16.2 New onset vascular disorders ('medically significant conditions'): Cervarix vs. Aimmugen: hepatitis A vaccine (females, age 22-29; follow-up until 12 months)**						
HPV-063: Cervarix vs. Aimmugen (f, 22-29; 12)	0	375	0	377		Not estimable
**Subtotal (95% CI)**		**375**		**377**		**Not estimable**
Total events	0		0			
Heterogeneity: Not applicable						
Test for overall effect: Not applicable						
**3.16.3 New onset vascular disorders ('medically significant conditions'): Cervarix vs. Havrix: hepatitis A vaccine (females, age 10-14; follow-up until 12 months)**						
HPV-013: Cervarix vs. Havrix (f, 10-14; 12)	0	1035	0	1032		Not estimable
**Subtotal (95% CI)**		**1035**		**1032**		**Not estimable**
Total events	0		0			
Heterogeneity: Not applicable						
Test for overall effect: Not applicable						
**3.16.4 New onset vascular disorders ('medically significant conditions'): Cervarix vs. Havrix: hepatitis A vaccine (females, age 15-25; follow-up until 48 months)**						
HPV-008: Cervarix vs. Havrix (f, 15-25; 48)	0	9319	0	9325		Not estimable
**Subtotal (95% CI)**		**9319**		**9325**		**Not estimable**
Total events	0		0			
Heterogeneity: Not applicable						
Test for overall effect: Not applicable						
**3.16.5 New onset vascular disorders ('medically significant conditions'): Cervarix vs. Twinrix: hepatitis A and B vaccine (females, age 8-15; follow-up until 12 months)**						
HPV-029: Cervarix vs. Twinrix (f, 8-15; 12)	0	270	0	271		Not estimable
**Subtotal (95% CI)**		**270**		**271**		**Not estimable**
Total events	0		0			
Heterogeneity: Not applicable						
Test for overall effect: Not applicable						
**3.16.6 New onset vascular disorders ('new medical history'): Gardasil vs. carrier solution (females and males, age 9-16; follow-up until 18 months)**						
V501-018: Gardasil vs. carrier solu. (f&m,9-16;18)	0	1184	0	597		Not estimable
**Subtotal (95% CI)**		**1184**		**597**		**Not estimable**
Total events	0		0			
Heterogeneity: Not applicable						
Test for overall effect: Not applicable						
**3.16.7 New onset vascular disorders ('new medical history'): Gardasil vs. AAHS (females, age 15-26; follow-up until 45 months)**						
V501-013: Gardasil vs. AAHS (f, 16-24; 45)	0	2723	0	2732		Not estimable
V501-015: Gardasil vs. AAHS (f, 15-26; 36)	0	6087	0	6080		Not estimable
**Subtotal (95% CI)**		**8810**		**8812**		**Not estimable**
Total events	0		0			
Heterogeneity: Not applicable						
Test for overall effect: Not applicable						
**3.16.8 New onset vascular disorders ('new medical history'): Gardasil vs. AAHS (females, age 21-46; follow-up until 48 months)**						
V501-019: Gardasil vs. AAHS (f, 21-46; 48)	0	1911	0	1908		Not estimable
**Subtotal (95% CI)**		**1911**		**1908**		**Not estimable**
Total events	0		0			
Heterogeneity: Not applicable						
Test for overall effect: Not applicable						
**3.16.9 New onset vascular disorders ('new medical history'): Gardasil vs. AAHS (males, age 15-27; follow-up until 36 months)**						
V501-020: Gardasil vs. AAHS (m, 15-27; 36)	0	2032	0	2033		Not estimable
**Subtotal (95% CI)**		**2032**		**2033**		**Not estimable**
Total events	0		0			
Heterogeneity: Not applicable						
Test for overall effect: Not applicable						
**3.16.10 New onset vascular disorders ('new medical history'): HPV 16 vaccine vs. AAHS (females, age 16-25; follow-up until 48 months)**						
V501-005: HPV 16 vaccine vs. AAHS (f, 16-25; 48)	0	1204	0	1205		Not estimable
**Subtotal (95% CI)**		**1204**		**1205**		**Not estimable**
Total events	0		0			
Heterogeneity: Not applicable						
Test for overall effect: Not applicable						
**Total (95% CI)**		**29022**		**28431**		**Not estimable**
Total events	0		0			
Heterogeneity: Not applicable						
Test for overall effect: Not applicable						
Test for subgroup differences: Not applicable						

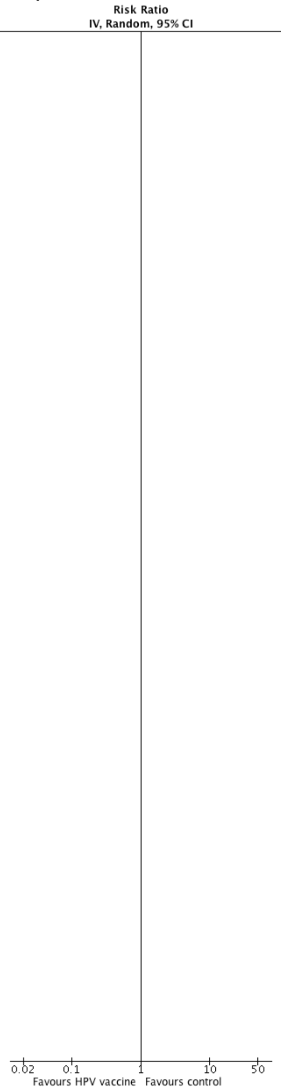

*3.16. Risk ratio for 'medically significant conditions’ (GlaxoSmithKline): not applicable; risk ratio for ‘new medical history’ (Merck Sharp & Dohme): not applicable.

3.17. General harms (‘solicited,’ ‘unsolicited’ and ‘systemic adverse events’*): intention to treat analysis

Study or Subgroup	HPV vaccine Events	HPV vaccine Total	Control Events	Control Total	Weight	Risk Ratio IV, Random, 95% CI
**3.17.1 General harms ('solicited' and 'unsolicited'): Cervarix vs. Al(OH)3 (females, age 9-17; follow-up until 12 months)**						
HPV-058: Cervarix vs. Al(OH)3 (f, 9-17; 12)	139	374	125	376	3.8%	1.12 [0.92, 1.36]
**Subtotal (95% CI)**		**374**		**376**	**3.8%**	**1.12 [0.92, 1.36]**
Total events	139		125			
Heterogeneity: Not applicable						
Test for overall effect: Z = 1.12 (P = 0.26)						
**3.17.2 General harms ('solicited' and 'unsolicited'): Cervarix vs. Al(OH)3 (females, age 15-26; follow-up until 27 months)**						
HPV-001: Cervarix vs. Al(OH)3 (f, 15-26; 27)	458	560	462	553	10.5%	0.98 [0.93, 1.03]
HPV-038: Cervarix vs. Al(OH)3 (f, 15-25; 7)	89	149	22	76	1.3%	2.06 [1.42, 3.01]
**Subtotal (95% CI)**		**709**		**629**	**11.8%**	**1.39 [0.67, 2.88]**
Total events	547		484			
Heterogeneity: Tau² = 0.26; Chi² = 14.80, df = 1 (P = 0.0001); I² = 93%						
Test for overall effect: Z = 0.88 (P = 0.38)						
**3.17.3 General harms ('solicited' and 'unsolicited'): Cervarix vs. Al(OH)3 (females, age 18-35; follow-up until 12 months)**						
HPV-003: Cervarix vs. Al(OH)3 (f, 18-30; 12)	0	31	0	30		Not estimable
HPV-031: Cervarix vs. Al(OH)3 (f, 18-35; 7)	0	176	0	178		Not estimable
HPV-035: Cervarix vs. Al(OH)3 (f, 18-35; 7)	104	150	85	150	4.4%	1.22 [1.03, 1.46]
**Subtotal (95% CI)**		**357**		**358**	**4.4%**	**1.22 [1.03, 1.46]**
Total events	104		85			
Heterogeneity: Not applicable						
Test for overall effect: Z = 2.25 (P = 0.02)						
**3.17.4 General harms ('solicited' and 'unsolicited'): Cervarix vs. Al(OH)3 (females, age 24-72; follow-up until 36 months)**						
HPV-015: Cervarix vs. Al(OH)3 (f, 24-72; 36)	1878	2882	1659	2871	11.2%	1.13 [1.08, 1.18]
**Subtotal (95% CI)**		**2882**		**2871**	**11.2%**	**1.13 [1.08, 1.18]**
Total events	1878		1659			
Heterogeneity: Not applicable						
Test for overall effect: Z = 5.73 (P < 0.00001)						
**3.17.5 General harms ('solicited' and 'unsolicited'): Cervarix vs. Engerix: hepatitis B vaccine (females and males, age 12-16; follow-up until 72 months)**						
HPV-030: Cervarix vs. Engerix (f, 9-15; 12)	135	247	130	246	4.8%	1.03 [0.88, 1.22]
HPV-040: Cervarix vs. Engerix (f & m, 12-16; 72)	157	604	202	1028	4.2%	1.32 [1.10, 1.59]
**Subtotal (95% CI)**		**851**		**1274**	**9.0%**	**1.17 [0.92, 1.48]**
Total events	292		332			
Heterogeneity: Tau² = 0.02; Chi² = 3.86, df = 1 (P = 0.05); I² = 74%						
Test for overall effect: Z = 1.25 (P = 0.21)						
**3.17.6 General harms ('solicited' and 'unsolicited'): Cervarix vs. Engerix: hepatitis B vaccine (females, age 26-46; follow-up until 12 months)**						
HPV-069: Cervarix vs. Engerix (f, 26-46; 12)	32	606	36	606	0.9%	0.89 [0.56, 1.41]
**Subtotal (95% CI)**		**606**		**606**	**0.9%**	**0.89 [0.56, 1.41]**
Total events	32		36			
Heterogeneity: Not applicable						
Test for overall effect: Z = 0.50 (P = 0.62)						
**3.17.7 General harms ('solicited' and 'unsolicited'): Cervarix vs. Havrix: hepatitis A vaccine (females, age 9-15; follow-up until 12 months)**						
HPV-013: Cervarix vs. Havrix (f, 10-14; 12)	0	1035	0	1032		Not estimable
HPV-033: Cervarix vs. Havrix (f, 9-15; 7)	0	160	0	161		Not estimable
**Subtotal (95% CI)**		**1195**		**1193**		**Not estimable**
Total events	0		0			
Heterogeneity: Not applicable						
Test for overall effect: Not applicable						
**3.17.8 General harms ('solicited' and 'unsolicited'): Cervarix vs. Havrix: hepatitis A vaccine (females, age 15-25; follow-up until 48 months)**						
HPV-008: Cervarix vs. Havrix (f, 15-25; 48)	0	9319	0	9325		Not estimable
**Subtotal (95% CI)**		**9319**		**9325**		**Not estimable**
Total events	0		0			
Heterogeneity: Not applicable						
Test for overall effect: Not applicable						
**3.17.9 General harms ('solicited' and 'unsolicited'): Cervarix vs. Twinrix: hepatitis A and B vaccine (females, age 8-15; follow-up until 12 months)**						
HPV-029: Cervarix vs. Twinrix (f, 8-15; 12)	0	270	0	271		Not estimable
**Subtotal (95% CI)**		**270**		**271**		**Not estimable**
Total events	0		0			
Heterogeneity: Not applicable						
Test for overall effect: Not applicable						
**3.17.10 General harms ('systemic adverse events'): Gardasil vs. carrier solution (females and males, age 9-16; follow-up until 18 months)**						
V501-018: Gardasil vs. carrier solu. (f&m,9-16;18)	541	1184	260	597	7.2%	1.05 [0.94, 1.17]
**Subtotal (95% CI)**		**1184**		**597**	**7.2%**	**1.05 [0.94, 1.17]**
Total events	541		260			
Heterogeneity: Not applicable						
Test for overall effect: Z = 0.85 (P = 0.39)						
**3.17.11 General harms ('systemic adverse events'): Gardasil vs. AAHS (females, age 15-26; follow-up until 45 months)**						
V501-013: Gardasil vs. AAHS (f, 16-24; 45)	1745	2723	1701	2732	11.2%	1.03 [0.99, 1.07]
V501-015: Gardasil vs. AAHS (f, 15-26; 36)	275	6087	268	6080	4.8%	1.02 [0.87, 1.21]
**Subtotal (95% CI)**		**8810**		**8812**	**16.0%**	**1.03 [0.99, 1.07]**
Total events	2020		1969			
Heterogeneity: Tau² = 0.00; Chi² = 0.00, df = 1 (P = 0.96); I² = 0%						
Test for overall effect: Z = 1.42 (P = 0.15)						
**3.17.12 General harms ('systemic adverse events'): Gardasil vs. AAHS (females, age 21-46; follow-up until 48 months)**						
V501-019: Gardasil vs. AAHS (f, 21-46; 48)	1118	1911	1131	1908	10.5%	0.99 [0.94, 1.04]
**Subtotal (95% CI)**		**1911**		**1908**	**10.5%**	**0.99 [0.94, 1.04]**
Total events	1118		1131			
Heterogeneity: Not applicable						
Test for overall effect: Z = 0.49 (P = 0.63)						
**3.17.13 General harms ('systemic adverse events'): Gardasil vs. AAHS (males, age 15-27; follow-up until 36 months)**						
V501-020: Gardasil vs. AAHS (m, 15-27; 36)	616	2032	622	2033	8.2%	0.99 [0.90, 1.09]
**Subtotal (95% CI)**		**2032**		**2033**	**8.2%**	**0.99 [0.90, 1.09]**
Total events	616		622			
Heterogeneity: Not applicable						
Test for overall effect: Z = 0.19 (P = 0.85)						
**3.17.14 General harms ('systemic adverse events'): Gardasil 9 vs. saline placebo (females, age 12-26; follow-up until 7 months)**						
V503-006: Gardasil 9 vs. saline (f, 12-26;7)	363	618	170	306	6.7%	1.06 [0.94, 1.19]
**Subtotal (95% CI)**		**618**		**306**	**6.7%**	**1.06 [0.94, 1.19]**
Total events	363		170			
Heterogeneity: Not applicable						
Test for overall effect: Z = 0.91 (P = 0.36)						
**3.17.15 General harms ('systemic adverse events'): HPV 16 vaccine vs. AAHS (females, age 16-25; follow-up until 48 months)**						
V501-005: HPV 16 vaccine vs. AAHS (f, 16-25; 48)	807	1204	824	1205	10.4%	0.98 [0.93, 1.04]
**Subtotal (95% CI)**		**1204**		**1205**	**10.4%**	**0.98 [0.93, 1.04]**
Total events	807		824			
Heterogeneity: Not applicable						
Test for overall effect: Z = 0.71 (P = 0.48)						
**Total (95% CI)**		**32322**		**31764**	**100.0%**	**1.05 [1.01, 1.10]**
Total events	8457		7697			
Heterogeneity: Tau² = 0.00; Chi² = 53.00, df = 14 (P < 0.00001); I² = 74%						
Test for overall effect: Z = 2.28 (P = 0.02)						
Test for subgroup differences: Chi² = 30.09, df = 11 (P = 0.002), I² = 63.4%						

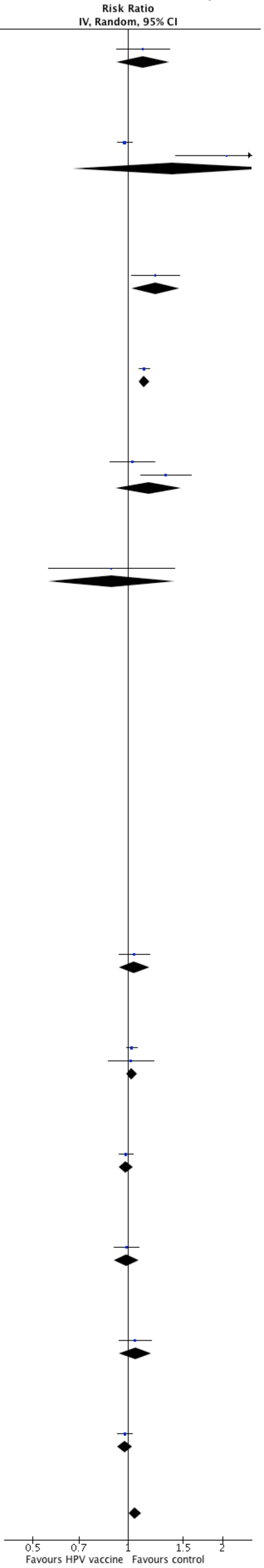

*3.17. Risk ratio for ‘solicited’ (GlaxoSmithKline): **1.14 [1.03, 1.26]**; risk ratio for ‘unsolicited’ (GlaxoSmithKline): 1.01 [0.98, 1.03]; risk ratio for ‘systemic adverse events’ (Merck Sharp & Dohme): 1.01 [0.97, 1.04]. To avoid double counting of participants in the total risk ratio estimate, we only included ‘solicited’ adverse events if the journal publication also had reported ‘unsolicited’ adverse events.

3.18. General harms most associated with the HPV vaccines (‘solicited,’ ‘unsolicited’ and ‘systemic adverse events’*) - ‘fatigue’: intention to treat analysis

Study or Subgroup	HPV vaccine Events	HPV vaccine Total	Control Events	Control Total	Weight	Risk Ratio IV, Random, 95% CI
**3.18.1 General fatigue ('solicited'): Cervarix vs. Al(OH)3 (females, age 9-17; follow-up until 12 months)**						
HPV-058: Cervarix vs. Al(OH)3 (f, 9-17; 12)	0	374	0	376		Not estimable
**Subtotal (95% CI)**		**374**		**376**		**Not estimable**
Total events	0		0			
Heterogeneity: Not applicable						
Test for overall effect: Not applicable						
**3.18.2 General fatigue ('solicited'): Cervarix vs. Al(OH)3 (females, age 15-26; follow-up until 27 months)**						
HPV-001: Cervarix vs. Al(OH)3 (f, 15-26; 27)	308	560	289	553	14.4%	1.05 [0.94, 1.17]
HPV-038: Cervarix vs. Al(OH)3 (f, 15-25; 7)	73	149	25	76	5.1%	1.49 [1.04, 2.14]
**Subtotal (95% CI)**		**709**		**629**	**19.5%**	**1.20 [0.86, 1.66]**
Total events	381		314			
Heterogeneity: $Tau^2 = 0.04$; $Chi^2 = 3.26$, df = 1 (P = 0.07); $I^2 = 69\%$						
Test for overall effect: Z = 1.08 (P = 0.28)						
**3.18.3 General fatigue ('solicited'): Cervarix vs. Al(OH)3 (females, age 18-35; follow-up until 12 months)**						
HPV-003: Cervarix vs. Al(OH)3 (f, 18-30; 12)	0	31	0	30		Not estimable
HPV-031: Cervarix vs. Al(OH)3 (f, 18-35; 7)	0	176	0	178		Not estimable
HPV-035: Cervarix vs. Al(OH)3 (f, 18-35; 7)	66	150	47	150	6.6%	1.40 [1.04, 1.89]
**Subtotal (95% CI)**		**357**		**358**	**6.6%**	**1.40 [1.04, 1.89]**
Total events	66		47			
Heterogeneity: Not applicable						
Test for overall effect: Z = 2.23 (P = 0.03)						
**3.18.4 General fatigue ('solicited'): Cervarix vs. Al(OH)3 (females, age 24-72; follow-up until 36 months)**						
HPV-015: Cervarix vs. Al(OH)3 (f, 24-72; 36)	0	2882	0	1871		Not estimable
**Subtotal (95% CI)**		**2882**		**1871**		**Not estimable**
Total events	0		0			
Heterogeneity: Not applicable						
Test for overall effect: Not applicable						
**3.18.5 General fatigue ('solicited'): Cervarix vs. Engerix: hepatitis B vaccine (females and males, age 9-16; follow-up until 72 months)**						
HPV-030: Cervarix vs. Engerix (f, 9-15; 12)	187	247	187	246	14.9%	1.00 [0.90, 1.10]
HPV-040: Cervarix vs. Engerix (f & m, 12-16; 72)	496	1593	596	2771	14.7%	1.45 [1.31, 1.60]
**Subtotal (95% CI)**		**1840**		**3017**	**29.6%**	**1.20 [0.83, 1.73]**
Total events	683		783			
Heterogeneity: $Tau^2 = 0.07$; $Chi^2 = 26.45$, df = 1 (P < 0.00001); $I^2 = 96\%$						
Test for overall effect: Z = 0.98 (P = 0.33)						
**3.18.6 General fatigue ('solicited'): Cervarix vs. Engerix: hepatitis B vaccine (females, age 26-46; follow-up until 12 months)**						
HPV-069: Cervarix vs. Engerix (f, 26-46; 12)	0	606	0	606		Not estimable
**Subtotal (95% CI)**		**606**		**606**		**Not estimable**
Total events	0		0			
Heterogeneity: Not applicable						
Test for overall effect: Not applicable						
**3.18.7 General fatigue ('solicited'): Cervarix vs. Havrix: hepatitis A vaccine (females, age 9-15; follow-up until 12 months)**						
HPV-013: Cervarix vs. Havrix (f, 10-14; 12)	893	1035	752	1032	17.0%	1.18 [1.13, 1.24]
HPV-033: Cervarix vs. Havrix (f, 9-15; 7)	90	160	87	161	10.2%	1.04 [0.85, 1.27]
**Subtotal (95% CI)**		**1195**		**1193**	**27.2%**	**1.15 [1.04, 1.28]**
Total events	983		839			
Heterogeneity: $Tau^2 = 0.00$; $Chi^2 = 1.56$, df = 1 (P = 0.21); $I^2 = 36\%$						
Test for overall effect: Z = 2.71 (P = 0.007)						
**3.18.8 General fatigue ('solicited'): Cervarix vs. Havrix: hepatitis A vaccine (females, age 15-25; follow-up until 48 months)**						
HPV-008: Cervarix vs. Havrix (f, 15-25; 48)	0	9319	0	9325		Not estimable
**Subtotal (95% CI)**		**9319**		**9325**		**Not estimable**
Total events	0		0			
Heterogeneity: Not applicable						
Test for overall effect: Not applicable						
**3.18.9 General fatigue ('solicited'): Cervarix vs. Twinrix: hepatitis A and B vaccine (females, age 8-15; follow-up until 12 months)**						
HPV-029: Cervarix vs. Twinrix (f, 8-15; 12)	217	270	208	271	15.4%	1.05 [0.96, 1.14]
**Subtotal (95% CI)**		**270**		**271**	**15.4%**	**1.05 [0.96, 1.14]**
Total events	217		208			
Heterogeneity: Not applicable						
Test for overall effect: Z = 1.02 (P = 0.31)						
**3.18.10 General fatigue ('unsolicited'): Cervarix vs. Engerix: hepatitis B vaccine (females and males, age 9-16; follow-up until 72 months)**						
HPV-030: Cervarix vs. Engerix (f, 9-15; 12)	0	247	0	246		Not estimable
HPV-040: Cervarix vs. Engerix (f & m, 12-16; 72)	0	1593	0	2771		Not estimable
**Subtotal (95% CI)**		**1840**		**3017**		**Not estimable**
Total events	0		0			
Heterogeneity: Not applicable						
Test for overall effect: Not applicable						
**3.18.11 General fatigue ('systemic adverse events'): Gardasil vs. carrier solution (females and males, age 9-16; follow-up until 18 months)**						
V501-018: Gardasil vs. carrier solu. (f&m,9-16;18)	0	1184	0	597		Not estimable
**Subtotal (95% CI)**		**1184**		**597**		**Not estimable**
Total events	0		0			
Heterogeneity: Not applicable						
Test for overall effect: Not applicable						
**3.18.12 General fatigue ('systemic adverse events'): Gardasil vs. AAHS (males, age 15-27; follow-up until 36 months)**						
V501-020: Gardasil vs. AAHS (m, 15-27; 36)	13	2032	19	2033	1.7%	0.68 [0.34, 1.38]
**Subtotal (95% CI)**		**2032**		**2033**	**1.7%**	**0.68 [0.34, 1.38]**
Total events	13		19			
Heterogeneity: Not applicable						
Test for overall effect: Z = 1.06 (P = 0.29)						
**3.18.13 General fatigue ('systemic adverse events'): Gardasil 9 vs. saline placebo (females, age 12-26; follow-up until 7 months)**						
V503-006: Gardasil 9 vs. saline (f, 12-26;7)	0	618	0	306		Not estimable
**Subtotal (95% CI)**		**618**		**306**		**Not estimable**
Total events	0		0			
Heterogeneity: Not applicable						
Test for overall effect: Not applicable						
**3.18.14 General fatigue ('systemic adverse events'): HPV 16 vaccine vs. AAHS (females, age 16-25; follow-up until 48 months)**						
V501-005: HPV 16 vaccine vs. AAHS (f, 16-25; 48)	0	1204	0	1205		Not estimable
**Subtotal (95% CI)**		**1204**		**1205**		**Not estimable**
Total events	0		0			
Heterogeneity: Not applicable						
Test for overall effect: Not applicable						
**Total (95% CI)**		**24430**		**24804**	**100.0%**	**1.15 [1.04, 1.26]**
Total events	2343		2210			
Heterogeneity: $Tau^2 = 0.01$; $Chi^2 = 42.84$, df = 8 (P < 0.00001); $I^2 = 81\%$						
Test for overall effect: Z = 2.78 (P = 0.005)						
Test for subgroup differences: $Chi^2 = 6.77$, df = 5 (P = 0.24), $I^2 = 26.2\%$						

Risk Ratio
IV, Random, 95% CI

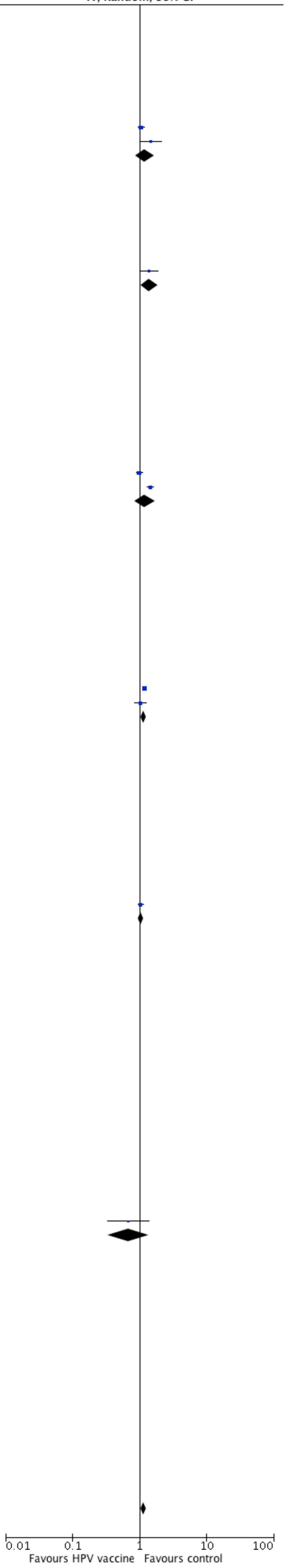

*3.18. Risk ratio for ‘solicited’ (GlaxoSmithKline): **1.16 [1.05, 1.28]**; risk ratio for ‘unsolicited’ (GlaxoSmithKline): not applicable; risk ratio for ‘systemic adverse events’ (Merck Sharp & Dohme): 0.68 [0.34, 1.38]. To avoid double counting of participants in the total risk ratio estimate, we excluded the ‘unsolicited’ adverse events from total risk ratio estimate for studies that reported ‘solicited’ adverse events.

3.19. General harms most associated with the HPV vaccines (‘solicited,’ ‘unsolicited’ and ‘systemic adverse events’*) - ‘headache’: intention to treat analysis

Study or Subgroup	HPV vaccine Events	HPV vaccine Total	Control Events	Control Total	Weight	Risk Ratio IV, Random, 95% CI
**3.19.1 General headache ('solicited'): Cervarix vs. Al(OH)3 (females, age 9-17; follow-up until 12 months)**						
HPV-058: Cervarix vs. Al(OH)3 (f, 9-17; 12)	0	374	0	376		Not estimable
**Subtotal (95% CI)**		**374**		**376**		**Not estimable**
Total events	0		0			
Heterogeneity: Not applicable						
Test for overall effect: Not applicable						
**3.19.2 General headache ('solicited'): Cervarix vs. Al(OH)3 (females, age 15-26; follow-up until 27 months)**						
HPV-001: Cervarix vs. Al(OH)3 (f, 15-26; 27)	331	560	329	553	14.9%	0.99 [0.90, 1.10]
HPV-038: Cervarix vs. Al(OH)3 (f, 15-25; 7)	44	149	16	76	1.8%	1.40 [0.85, 2.32]
**Subtotal (95% CI)**		**709**		**629**	**16.8%**	**1.08 [0.81, 1.44]**
Total events	375		345			
Heterogeneity: Tau^2 = 0.03; Chi^2 = 1.75, df = 1 (P = 0.19); I^2 = 43%						
Test for overall effect: Z = 0.51 (P = 0.61)						
**3.19.3 General headache ('solicited'): Cervarix vs. Al(OH)3 (females, age 18-35; follow-up until 12 months)**						
HPV-003: Cervarix vs. Al(OH)3 (f, 18-30; 12)	0	31	0	30		Not estimable
HPV-031: Cervarix vs. Al(OH)3 (f, 18-35; 7)	0	176	0	178		Not estimable
HPV-035: Cervarix vs. Al(OH)3 (f, 18-35; 7)	0	150	0	150		Not estimable
**Subtotal (95% CI)**		**357**		**358**		**Not estimable**
Total events	0		0			
Heterogeneity: Not applicable						
Test for overall effect: Not applicable						
**3.19.4 General headache ('solicited'): Cervarix vs. Al(OH)3 (females, age 24-72; follow-up until 36 months)**						
HPV-015: Cervarix vs. Al(OH)3 (f, 24-72; 36)	0	2882	0	2871		Not estimable
**Subtotal (95% CI)**		**2882**		**2871**		**Not estimable**
Total events	0		0			
Heterogeneity: Not applicable						
Test for overall effect: Not applicable						
**3.19.5 General headache ('solicited'): Cervarix vs. Engerix: hepatitis B vaccine (females and males, age 9-16; follow-up until 72 months)**						
HPV-030: Cervarix vs. Engerix (f, 9-15; 12)	226	247	210	246	17.7%	1.07 [1.01, 1.14]
HPV-040: Cervarix vs. Engerix (f & m, 12-16; 72)	381	1593	521	2771	13.3%	1.27 [1.13, 1.43]
**Subtotal (95% CI)**		**1840**		**3017**	**31.0%**	**1.16 [0.98, 1.37]**
Total events	607		731			
Heterogeneity: Tau^2 = 0.01; Chi^2 = 6.34, df = 1 (P = 0.01); I^2 = 84%						
Test for overall effect: Z = 1.73 (P = 0.08)						
**3.19.6 General headache ('solicited'): Cervarix vs. Engerix: hepatitis B vaccine (females, age 26-46; follow-up until 12 months)**						
HPV-069: Cervarix vs. Engerix (f, 26-46; 12)	0	606	0	606		Not estimable
**Subtotal (95% CI)**		**606**		**606**		**Not estimable**
Total events	0		0			
Heterogeneity: Not applicable						
Test for overall effect: Not applicable						
**3.19.7 General headache ('solicited'): Cervarix vs. Havrix: hepatitis A vaccine (females, age 9-15; follow-up until 12 months)**						
HPV-013: Cervarix vs. Havrix (f, 10-14; 12)	891	1035	778	1032	19.3%	1.14 [1.09, 1.19]
HPV-033: Cervarix vs. Havrix (f, 9-15; 7)	62	160	76	161	5.7%	0.82 [0.64, 1.06]
**Subtotal (95% CI)**		**1195**		**1193**	**25.0%**	**0.99 [0.72, 1.37]**
Total events	953		854			
Heterogeneity: Tau^2 = 0.05; Chi^2 = 6.30, df = 1 (P = 0.01); I^2 = 84%						
Test for overall effect: Z = 0.05 (P = 0.96)						
**3.19.8 General headache ('solicited'): Cervarix vs. Havrix: hepatitis A vaccine (females, age 15-25; follow-up until 48 months)**						
HPV-008: Cervarix vs. Havrix (f, 15-25; 48)	0	9319	0	9325		Not estimable
**Subtotal (95% CI)**		**9319**		**9325**		**Not estimable**
Total events	0		0			
Heterogeneity: Not applicable						
Test for overall effect: Not applicable						
**3.19.9 General headache ('solicited'): Cervarix vs. Twinrix: hepatitis A and B vaccine (females, age 8-15; follow-up until 12 months)**						
HPV-029: Cervarix vs. Twinrix (f, 8-15; 12)	210	270	180	271	14.2%	1.17 [1.05, 1.30]
**Subtotal (95% CI)**		**270**		**271**	**14.2%**	**1.17 [1.05, 1.30]**
Total events	210		180			
Heterogeneity: Not applicable						
Test for overall effect: Z = 2.92 (P = 0.004)						
**3.19.10 General headache ('unsolicited'): Cervarix vs. Al(OH)3 (females, age 9-17; follow-up until 12 months)**						
HPV-058: Cervarix vs. Al(OH)3 (f, 9-17; 12)	0	374	0	376		Not estimable
**Subtotal (95% CI)**		**0**		**0**		**Not estimable**
Total events	0		0			
Heterogeneity: Not applicable						
Test for overall effect: Not applicable						
**3.19.11 General headache ('unsolicited'): Cervarix vs. Al(OH)3 (females, age 15-25; follow-up until 7 months)**						
HPV-038: Cervarix vs. Al(OH)3 (f, 15-25; 7)	0	149	0	76		Not estimable
**Subtotal (95% CI)**		**0**		**0**		**Not estimable**
Total events	0		0			
Heterogeneity: Not applicable						
Test for overall effect: Not applicable						
**3.19.12 General headache ('unsolicited'): Cervarix vs. Al(OH)3 (females, age 18-35; follow-up until 7 months)**						
HPV-031: Cervarix vs. Al(OH)3 (f, 18-35; 7)	0	176	0	178		Not estimable
HPV-035: Cervarix vs. Al(OH)3 (f, 18-35; 7)	0	150	0	150		Not estimable
**Subtotal (95% CI)**		**0**		**0**		**Not estimable**
Total events	0		0			
Heterogeneity: Not applicable						
Test for overall effect: Not applicable						
**3.19.13 General headache ('unsolicited'): Cervarix vs. Engerix: hepatitis B vaccine (females and males, age 9-16; follow-up until 72 months)**						
HPV-030: Cervarix vs. Engerix (f, 9-15; 12)	0	247	0	246		Not estimable
HPV-040: Cervarix vs. Engerix (f & m, 12-16; 72)	23	1593	16	2771	0.0%	2.50 [1.33, 4.72]
**Subtotal (95% CI)**		**0**		**0**		**Not estimable**
Total events	0		0			
Heterogeneity: Not applicable						
Test for overall effect: Not applicable						
**3.19.14 General headache ('unsolicited'): Cervarix vs. Twinrix: hepatitis A and B vaccine (females, age 8-15; follow-up until 12 months)**						
HPV-029: Cervarix vs. Twinrix (f, 8-15; 12)	0	270	0	271		Not estimable
**Subtotal (95% CI)**		**0**		**0**		**Not estimable**
Total events	0		0			
Heterogeneity: Not applicable						
Test for overall effect: Not applicable						
**3.19.15 General headache ('systemic adverse events'): Gardasil vs. carrier solution (females and males, age 9-16; follow-up until 18 months)**						
V501-018: Gardasil vs. carrier solu. (f&m,9-16;18)	0	1184	0	597		Not estimable
**Subtotal (95% CI)**		**1184**		**597**		**Not estimable**
Total events	0		0			
Heterogeneity: Not applicable						
Test for overall effect: Not applicable						
**3.19.16 General headache ('systemic adverse events'): Gardasil vs. AAHS (males, age 15-27; follow-up until 36 months)**						
V501-020: Gardasil vs. AAHS (m, 15-27; 36)	179	2032	207	2033	8.3%	0.87 [0.72, 1.05]
**Subtotal (95% CI)**		**2032**		**2033**	**8.3%**	**0.87 [0.72, 1.05]**
Total events	179		207			
Heterogeneity: Not applicable						
Test for overall effect: Z = 1.49 (P = 0.14)						
**3.19.17 General headachec ('systemic adverse events'): Gardasil 9 vs. saline placebo (females, age 12-26; follow-up until 7 months)**						
V503-006: Gardasil 9 vs. saline (f, 12-26;7)	119	618	55	306	4.7%	1.07 [0.80, 1.43]
**Subtotal (95% CI)**		**618**		**306**	**4.7%**	**1.07 [0.80, 1.43]**
Total events	119		55			
Heterogeneity: Not applicable						
Test for overall effect: Z = 0.47 (P = 0.64)						
**3.19.18 General headache ('systemic adverse events'): HPV 16 vaccine vs. AAHS (females, age 16-25; follow-up until 48 months)**						
V501-005: HPV 16 vaccine vs. AAHS (f, 16-25; 48)	0	1204	0	1205		Not estimable
**Subtotal (95% CI)**		**1204**		**1205**		**Not estimable**
Total events	0		0			
Heterogeneity: Not applicable						
Test for overall effect: Not applicable						
**Total (95% CI)**		**22590**		**22787**	**100.0%**	**1.08 [1.01, 1.16]**
Total events	2443		2372			
Heterogeneity: Tau^2 = 0.01; Chi^2 = 26.92, df = 8 (P = 0.0007); I^2 = 70%						
Test for overall effect: Z = 2.13 (P = 0.03)						
Test for subgroup differences: Chi^2 = 8.27, df = 5 (P = 0.14), I^2 = 39.5%						

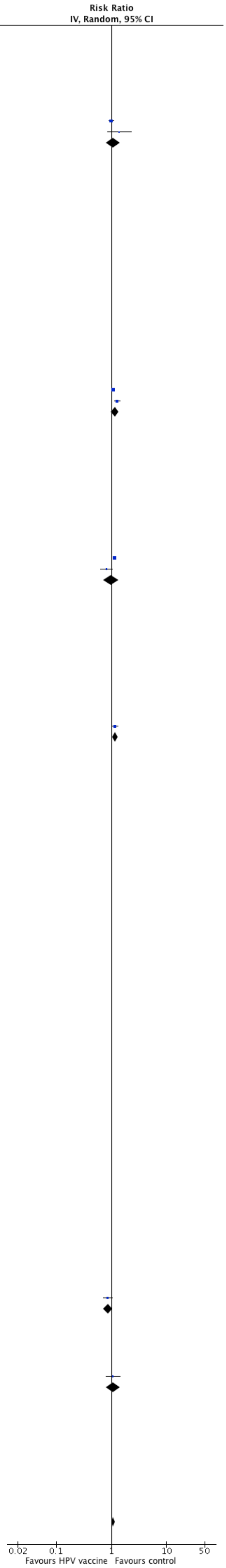

*3.19. Risk ratio for ‘solicited’ (GlaxoSmithKline): **1.11 [1.03, 1.19]**; risk ratio for ‘unsolicited’ (GlaxoSmithKline): **2.50 [1.33, 4.72]**; risk ratio for ‘systemic adverse events’ (Merck Sharp & Dohme): 0.94 [0.76, 1.14]. To avoid double counting of participants in the total risk ratio estimate, we excluded the ‘unsolicited’ adverse events from total risk ratio estimate for studies that reported ‘solicited’ adverse events.

3.20. General harms most associated with the HPV vaccines (‘solicited,’ ‘unsolicited’ and ‘systemic adverse events’*) - ‘myalgia’: intention to treat analysis

Study or Subgroup	HPV vaccine Events	HPV vaccine Total	Control Events	Control Total	Weight	Risk Ratio IV, Random, 95% CI
**3.20.1 General myalgia ('solicited'): Cervarix vs. Al(OH)3 (females, age 9-17; follow-up until 12 months)**						
HPV-058: Cervarix vs. Al(OH)3 (f, 9-17; 12)	0	374	0	376		Not estimable
**Subtotal (95% CI)**		**374**		**376**		**Not estimable**
Total events	0		0			
Heterogeneity: Not applicable						
Test for overall effect: Not applicable						
**3.20.2 General myalgia ('solicited'): Cervarix vs. Al(OH)3 (females, age 15-25; follow-up until 7 months)**						
HPV-038: Cervarix vs. Al(OH)3 (f, 15-25; 7)	66	149	20	76	11.1%	1.68 [1.11, 2.55]
**Subtotal (95% CI)**		**149**		**76**	**11.1%**	**1.68 [1.11, 2.55]**
Total events	66		20			
Heterogeneity: Not applicable						
Test for overall effect: Z = 2.45 (P = 0.01)						
**3.20.3 General myalgia ('solicited'): Cervarix vs. Al(OH)3 (females, age 18-35; follow-up until 12 months)**						
HPV-003: Cervarix vs. Al(OH)3 (f, 18-30; 12)	0	31	0	30		Not estimable
HPV-031: Cervarix vs. Al(OH)3 (f, 18-35; 7)	0	176	0	178		Not estimable
HPV-035: Cervarix vs. Al(OH)3 (f, 18-35; 7)	60	150	38	150	12.4%	1.58 [1.13, 2.21]
**Subtotal (95% CI)**		**357**		**358**	**12.4%**	**1.58 [1.13, 2.21]**
Total events	60		38			
Heterogeneity: Not applicable						
Test for overall effect: Z = 2.65 (P = 0.008)						
**3.20.4 General myalgia ('solicited'): Cervarix vs. Al(OH)3 (females, age 24-72; follow-up until 36 months)**						
HPV-015: Cervarix vs. Al(OH)3 (f, 24-72; 36)	0	2882	0	2871		Not estimable
**Subtotal (95% CI)**		**2882**		**2871**		**Not estimable**
Total events	0		0			
Heterogeneity: Not applicable						
Test for overall effect: Not applicable						
**3.20.5 General myalgia ('solicited'): Cervarix vs. Engerix: hepatitis B vaccine (females and males, age 9-16; follow-up until 72 months)**						
HPV-030: Cervarix vs. Engerix (f, 9-15; 12)	70	247	74	246	13.5%	0.94 [0.72, 1.24]
HPV-040: Cervarix vs. Engerix (f & m, 12-16; 72)	539	1593	347	2771	15.6%	2.70 [2.40, 3.05]
**Subtotal (95% CI)**		**1840**		**3017**	**29.1%**	**1.61 [0.57, 4.51]**
Total events	609		421			
Heterogeneity: $Tau^2 = 0.54$; $Chi^2 = 47.37$, df = 1 ($P < 0.00001$); $I^2 = 98\%$						
Test for overall effect: Z = 0.90 (P = 0.37)						
**3.20.6 General myalgia ('solicited'): Cervarix vs. Engerix: hepatitis B vaccine (females, age 26-46; follow-up until 12 months)**						
HPV-069: Cervarix vs. Engerix (f, 26-46; 12)	0	606	0	606		Not estimable
**Subtotal (95% CI)**		**606**		**606**		**Not estimable**
Total events	0		0			
Heterogeneity: Not applicable						
Test for overall effect: Not applicable						
**3.20.7 General myalgia ('solicited'): Cervarix vs. Havrix: hepatitis A vaccine (females, age 9-15; follow-up until 12 months)**						
HPV-013: Cervarix vs. Havrix (f, 10-14; 12)	890	1035	552	1032	16.1%	1.61 [1.51, 1.71]
HPV-033: Cervarix vs. Havrix (f, 9-15; 7)	79	160	40	161	12.9%	1.99 [1.46, 2.71]
**Subtotal (95% CI)**		**1195**		**1193**	**28.9%**	**1.69 [1.42, 2.01]**
Total events	969		592			
Heterogeneity: $Tau^2 = 0.01$; $Chi^2 = 1.72$, df = 1 (P = 0.19); $I^2 = 42\%$						
Test for overall effect: Z = 5.86 ($P < 0.00001$)						
**3.20.8 General myalgia ('solicited'): Cervarix vs. Havrix: hepatitis A vaccine (females, age 15-25; follow-up until 48 months)**						
HPV-008: Cervarix vs. Havrix (f, 15-25; 48)	0	9319	0	9325		Not estimable
**Subtotal (95% CI)**		**9319**		**9325**		**Not estimable**
Total events	0		0			
Heterogeneity: Not applicable						
Test for overall effect: Not applicable						
**3.20.9 General myalgia ('solicited'): Cervarix vs. Twinrix: hepatitis A and B vaccine (females, age 8-15; follow-up until 12 months)**						
HPV-029: Cervarix vs. Twinrix (f, 8-15; 12)	160	270	113	271	15.0%	1.42 [1.20, 1.69]
**Subtotal (95% CI)**		**270**		**271**	**15.0%**	**1.42 [1.20, 1.69]**
Total events	160		113			
Heterogeneity: Not applicable						
Test for overall effect: Z = 4.00 ($P < 0.0001$)						
**3.20.10 General myalgia ('unsolicited'): Cervarix vs. Al(OH)3 (females, age 15-25; follow-up until 7 months)**						
HPV-038: Cervarix vs. Al(OH)3 (f, 15-25; 7)	0	149	0	76		Not estimable
**Subtotal (95% CI)**		**149**		**76**		**Not estimable**
Total events	0		0			
Heterogeneity: Not applicable						
Test for overall effect: Not applicable						
**3.20.11 General myalgia ('unsolicited'): Cervarix vs. Al(OH)3 (females, age 18-35; follow-up until 7 months)**						
HPV-031: Cervarix vs. Al(OH)3 (f, 18-35; 7)	0	176	0	178		Not estimable
**Subtotal (95% CI)**		**176**		**178**		**Not estimable**
Total events	0		0			
Heterogeneity: Not applicable						
Test for overall effect: Not applicable						
**3.20.12 General myalgia ('unsolicited'): Cervarix vs. Engerix: hepatitis B vaccine (females and males, age 12-16; follow-up until 72 months)**						
HPV-040: Cervarix vs. Engerix (f & m, 12-16; 72)	0	1593	0	2771		Not estimable
**Subtotal (95% CI)**		**1593**		**2771**		**Not estimable**
Total events	0		0			
Heterogeneity: Not applicable						
Test for overall effect: Not applicable						
**3.20.13 General myalgia ('unsolicited'): Cervarix vs. Havrix: hepatitis A vaccine (females, age 9-15; follow-up until 7 months)**						
HPV-033: Cervarix vs. Havrix (f, 9-15; 7)	0	160	0	161		Not estimable
**Subtotal (95% CI)**		**160**		**161**		**Not estimable**
Total events	0		0			
Heterogeneity: Not applicable						
Test for overall effect: Not applicable						
**3.20.14 General myalgia ('systemic adverse events'): Gardasil vs. carrier solution (females and males, age 9-16; follow-up until 18 months)**						
V501-018: Gardasil vs. carrier solu. (f&m,9-16;18)	0	1184	0	597		Not estimable
**Subtotal (95% CI)**		**1184**		**597**		**Not estimable**
Total events	0		0			
Heterogeneity: Not applicable						
Test for overall effect: Not applicable						
**3.20.15 General myalgia ('systemic adverse events'): Gardasil vs. AAHS (females, age 15-26; follow-up until 45 months)**						
V501-015: Gardasil vs. AAHS (f, 15-26; 36)	4	6087	9	6080	3.4%	0.44 [0.14, 1.44]
**Subtotal (95% CI)**		**6087**		**6080**	**3.4%**	**0.44 [0.14, 1.44]**
Total events	4		9			
Heterogeneity: Not applicable						
Test for overall effect: Z = 1.35 (P = 0.18)						
**3.20.16 General myalgia ('systemic adverse events'): Gardasil vs. AAHS (males, age 15-27; follow-up until 36 months)**						
V501-020: Gardasil vs. AAHS (m, 15-27; 36)	0	2032	0	2033		Not estimable
**Subtotal (95% CI)**		**2032**		**2033**		**Not estimable**
Total events	0		0			
Heterogeneity: Not applicable						
Test for overall effect: Not applicable						
**3.20.17 General myalgia ('systemic adverse events'): Gardasil 9 vs. saline placebo (females, age 12-26; follow-up until 7 months)**						
V503-006: Gardasil 9 vs. saline (f, 12-26;7)	0	618	0	306		Not estimable
**Subtotal (95% CI)**		**618**		**306**		**Not estimable**
Total events	0		0			
Heterogeneity: Not applicable						
Test for overall effect: Not applicable						
**3.20.18 General myalgia ('systemic adverse events'): HPV 16 vaccine vs. AAHS (females, age 16-25; follow-up until 48 months)**						
V501-005: HPV 16 vaccine vs. AAHS (f, 16-25; 48)	0	1204	0	1205		Not estimable
**Subtotal (95% CI)**		**1204**		**1205**		**Not estimable**
Total events	0		0			
Heterogeneity: Not applicable						
Test for overall effect: Not applicable						
**Total (95% CI)**		**30195**		**31500**	**100.0%**	**1.57 [1.23, 2.01]**
Total events	1868		1193			
Heterogeneity: $Tau^2 = 0.10$; $Chi^2 = 88.28$, df = 7 ($P < 0.00001$); $I^2 = 92\%$						
Test for overall effect: Z = 3.60 (P = 0.0003)						
Test for subgroup differences: $Chi^2 = 6.39$, df = 5 (P = 0.27), $I^2 = 21.7\%$						

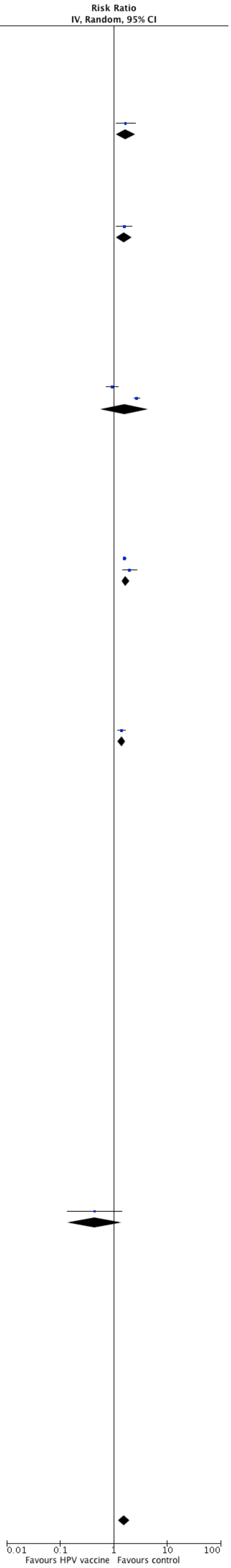

*3.20. Risk ratio for ‘solicited’ (GlaxoSmithKline): **1.64 [1.29, 2.10]**; risk ratio for ‘unsolicited’ (GlaxoSmithKline): not applicable; risk ratio for ‘systemic adverse events’ (Merck Sharp & Dohme): 0.44 [0.14, 1.44]. To avoid double counting of participants in the total risk ratio estimate, we excluded the ‘unsolicited’ adverse events from total risk ratio estimate for studies that reported ‘solicited’ adverse events.
